# Transcriptome analysis reveals manifold mechanisms of cyst development in ADPKD

**DOI:** 10.1186/s40246-016-0095-x

**Published:** 2016-11-21

**Authors:** Rita M. C. de Almeida, Sherry G. Clendenon, William G. Richards, Michael Boedigheimer, Michael Damore, Sandro Rossetti, Peter C. Harris, Britney-Shea Herbert, Wei Min Xu, Angela Wandinger-Ness, Heather H. Ward, James A. Glazier, Robert L. Bacallao

**Affiliations:** 1 Biocomplexity Institute and Department of Physics, Indiana University, Bloomington, IN 47405 USA; 2 Instituto de Física and Instituto Nacional de Ciência e Tecnologia, Universidade Federal do Rio Grande do Sul, 91501-970 Porto Alegre, RS Brazil; 3 Biocomplexity Institute and Department of Intelligent Systems Engineering, Indiana University, Bloomington, IN 47405 USA; 4 AMGEN Inc., One Amgen Center Drive, Thousand Oaks, CA 91320-1799 USA; 5 Division of Nephrology, Mayo Clinic, 200 First Street SW, Rochester, MN 55905 USA; 6 Department of Medical and Molecular Genetics, Indiana University School of Medicine, Indianapolis, IN 46202 USA; 7 Division of Nephrology, Department of Medicine, Richard Roudebush VAMC and Indiana University School of Medicine, Indianapolis, IN 46202 USA; 8 Department of Pathology MSC08-4640 and Cancer Research and Treatment Center, University of New Mexico Health Sciences Center, Albuquerque, NM 87131 USA; 9 Division of Nephrology, Department of Medicine, University of New Mexico Health Sciences Center, Albuquerque, NM 87131 USA

**Keywords:** Kidney, Transcriptogram, Cystic kidney disease, Autosomal dominant polycystic kidney disease, Bioinformatics, Pathway identification

## Abstract

**Background:**

Autosomal dominant polycystic kidney disease (ADPKD) causes progressive loss of renal function in adults as a consequence of the accumulation of cysts. ADPKD is the most common genetic cause of end-stage renal disease. Mutations in polycystin-1 occur in 87% of cases of ADPKD and mutations in polycystin-2 are found in 12% of ADPKD patients. The complexity of ADPKD has hampered efforts to identify the mechanisms underlying its pathogenesis. No current FDA (Federal Drug Administration)-approved therapies ameliorate ADPKD progression.

**Results:**

We used the de Almeida laboratory’s sensitive new transcriptogram method for whole-genome gene expression data analysis to analyze microarray data from cell lines developed from cell isolates of normal kidney and of both non-cystic nephrons and cysts from the kidney of a patient with ADPKD. We compared results obtained using standard Ingenuity Volcano plot analysis, Gene Set Enrichment Analysis (GSEA) and transcriptogram analysis. Transcriptogram analysis confirmed the findings of Ingenuity, GSEA, and published analysis of ADPKD kidney data and also identified multiple new expression changes in KEGG (Kyoto Encyclopedia of Genes and Genomes) pathways related to cell growth, cell death, genetic information processing, nucleotide metabolism, signal transduction, immune response, response to stimulus, cellular processes, ion homeostasis and transport and cofactors, vitamins, amino acids, energy, carbohydrates, drugs, lipids, and glycans. Transcriptogram analysis also provides significance metrics which allow us to prioritize further study of these pathways.

**Conclusions:**

Transcriptogram analysis identifies novel pathways altered in ADPKD, providing new avenues to identify both ADPKD’s mechanisms of pathogenesis and pharmaceutical targets to ameliorate the progression of the disease.

**Electronic supplementary material:**

The online version of this article (doi:10.1186/s40246-016-0095-x) contains supplementary material, which is available to authorized users.

## Background

Autosomal dominant polycystic kidney disease (ADPKD) is a monogenic disorder associated with cystic renal disease, liver cysts, cardiac valve abnormalities, and berry aneurysms [1]. Progressive accumulation of renal cysts in ADPKD culminates in kidney failure, usually during the fifth or sixth decades of life. With a disease incidence of 1 in 800, ADPKD is one of the most common human genetic disorders. Despite extensive research, no Federal Drug Administration (FDA)-approved treatments prevent or delay disease progression, limiting patient treatment options to dialysis or kidney transplantation [2].

Two gene loci are responsible for ADPKD; HmPKD1 (human polycystic kidney disease gene 1) and HmPKD2 (human polycystic kidney disease gene 2) located on chromosomes 16 and 4, respectively. The gene products of these loci are polycystin-1 (PC-1) and polycystin-2 (PC-2), proteins that form a heteromeric complex in primary cilium of renal cells [3–5]. Primary cilia are mechanically sensitive and serve as a flow sensor in renal tubular epithelia [6]. Due to the subcellular localization of polycystins, and the pleiotropic phenotypes associated with their mutations, Hildebrandt et al. called ADPKD the grandfather of ciliopathies [7].

Since ADPKD perturbs the Jnk/Stat (cJun N-terminal kinases/signal transducer and activator of transcription 1), MAPK (mitogen-activated protein kinase), mTOR (mammalian target of rapamycin), Wnt/Wgless (Wingless-related integration site/Wingless)and Erk (extracellular signal regulated kinase) kinase pathways [8], somatostatin analogues, vasopressin receptor antagonists, mTOR inhibitors, and statins have been tested for their ability to ameliorate the course of ADPKD. While mTOR inhibitors reduced cyst growth in murine polycystic kidney disease (PKD) models, they did not slow progression of renal impairment in human trials [9–11]. The FDA did not approve a recent vasopressin receptor antagonist, which inhibited cyst growth in several animal models of polycystic kidney disease, and slowed increases in total kidney volume and decreased estimated creatinine clearance in humans [12–14]. Because cyst formation in ADPKD begins in utero, drugs that can prevent initial cyst formation are not likely to be acceptable in a clinical setting [15, 16]. However, it is possible that the signaling pathways that initially drive cyst emergence are the unique entry points for treatment. We therefore concentrate on identifying the properties of ADPKD cells derived from normal nephrons and cysts from the same kidney to compare to kidney cells derived from an end-stage cystic kidney and normal human renal cortex. Our eventual goal would be to identify signaling pathways that provide a unique opening for targeted therapy in ADPKD.

The paucity of ADPKD drug candidates partially results from the difficulty in identifying pathways that promote cyst growth in renal cells but are not essential in other adult cell types. Standard gene-chip analysis of expressed mRNA shows that ADPKD perturbs Wnt/Wgless and Notch signaling [17, 18]. Decreased Notch signaling and mutated components in the Wnt pathway can cause renal cystic disease [18–20]. However, the Wnt/Wgless and Notch signaling pathways are essential to cell fate transitions and assignments in most cell types [21], so targeting these pathways would likely result in widespread and damaging bystander effects. Similarly, renal epithelial cells with an HmPKD1 mutation express many markers of immature cell differentiation, including increased proliferation rates, altered cell-ECM (extracellular matrix) adhesion, and increased apoptosis. However, most of these pathways are ubiquitous and thus not promising drug targets. To identify promising therapeutic targets, we need to compare expression patterns in cells from normal human renal cortex, normal nephrons in ADPKD patents, and in cysts from the same patients to identify pathways uniquely active in cystogenesis and not essential in other adult tissues. Identifying optimal drug targets also requires a coherent biological context that connects clusters of gene perturbations to the mechanisms of cyst formation, not just identification of individual perturbed genes in cystic ADPKD cells.

The de Almeida laboratory developed transcriptogram whole-genome gene expression data analysis to identify perturbed molecular networks and pathways more sensitively than conventional gene-chip analysis methods [22–24]. Transcriptogram analysis orders genes into a list using a Monte Carlo simulation that minimizes the distance within the list between associated gene products, so proximity in the list correlates with the degree of co-participation in biological processes. Calculating the average transcription level for genes within a moving window produces a transcriptogram profile which reveals any differentially expressed pathways. This robust statistical approach couples enhanced significance assessment with assessment of false discovery rates (FDRs). Transcriptogram analysis identifies rich sets of biological signaling interactions for later integrated hypothesis testing.

## Results and discussion

We generated three different telomerase-immortalized cell lines, derived from isolates of (i) a normal kidney of a 57-year-old male (designated as normal cells, NK), (ii) non-cystic tissue from the kidney of a 36-year-old male (designated as non-cystic ADPKD cells, NC-ADPKD), and (iii) cystic tissue from the same donor (designated as cystic ADPKD cells, C-ADPKD). The additional ADPKD cell line in this paper is a previously characterized telomerase immortalized cell line isolated from an end-stage polycystic kidney [25]. We refer to the expression patterns of the NK cells as normal. We refer to expression of a gene or pathway in ADPKD, NC-ADPKD, or C-ADPKD cells as higher-than-normal if the expression level in those cells is significantly greater than in NK cells. Conversely, we refer to expression of a gene or pathway in ADPKD, NC-ADPKD, or C-ADPKD cells as lower-than-normal if the expression level in those cells is significantly lower than in NK cells. Differences between the NC-ADPKD and NK cells are particularly significant because they may identify early changes permissive for cyst formation in morphologically normal kidney tissue. We obtained microarray data for genome-wide gene expression for all cell lines in triplicate. We then analyzed the resulting data using three different, complementary methods: single gene expression analysis using QIAGEN’s Ingenuity Pathway Analysis (IPA QIAGEN Redwood City), Gene Set Enrichment Analysis (GSEA) [26], and the transcriptogram method [22, 23, 27].

**Fig. 1:**
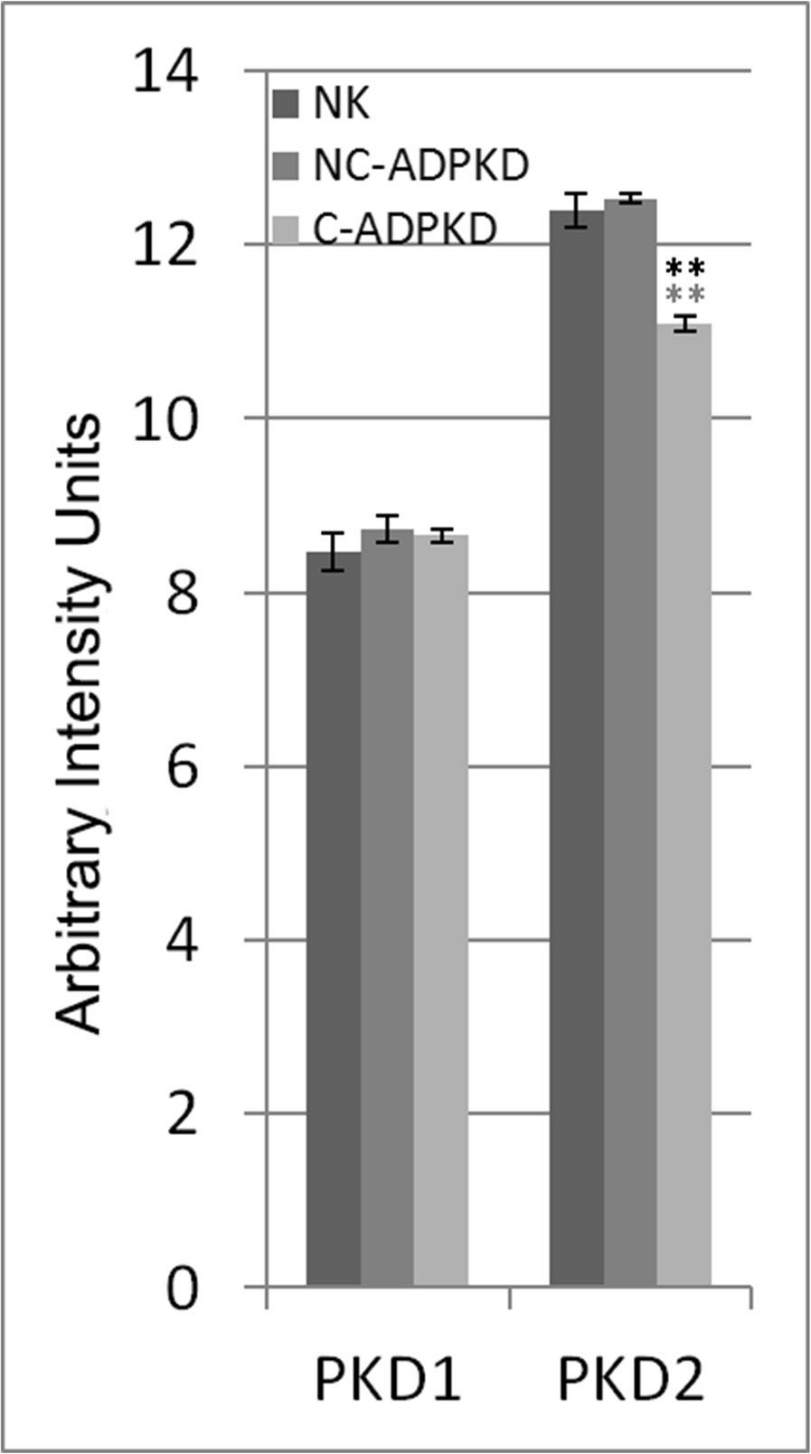
PKD1 and PKD2 gene expression in the NK, NC-ADPKD, and C-ADPKD cells. PKD1 expression is not different from normal in the NC-ADPKD and C-ADPKD cells. PKD2 expression is normal in the NC-ADPKD cells but significantly lower than normal in C-ADPKD cells. Bars show mean ± s.e., * * *p* < 0.01, * *p* < 0.05 (*black asterisk* is in comparison to NK, *gray asterisk* is in comparison to NC-ADPKD)

Mouse and human renal cells with aberrant polycystin-1 have impaired ciliary mechanosensation, and this impairment has been proposed as a cellular mechanism that promotes cystogenesis [28]. However, most cyst epithelia of human ADPKD kidneys express full-length polycystin-1 (PC-1) and polycystin-2 (PC-2) at normal or increased abundance [29–31]. Our PC-1 microarray data agrees with these observations (Fig. 1). Our microarray data show equal expression of PC-2 in NC-ADPKD and NK cells and reduced expression in C-ADPKD cells, a finding of uncertain significance.

### Volcano plots: single-gene analyses

**Fig. 2:**
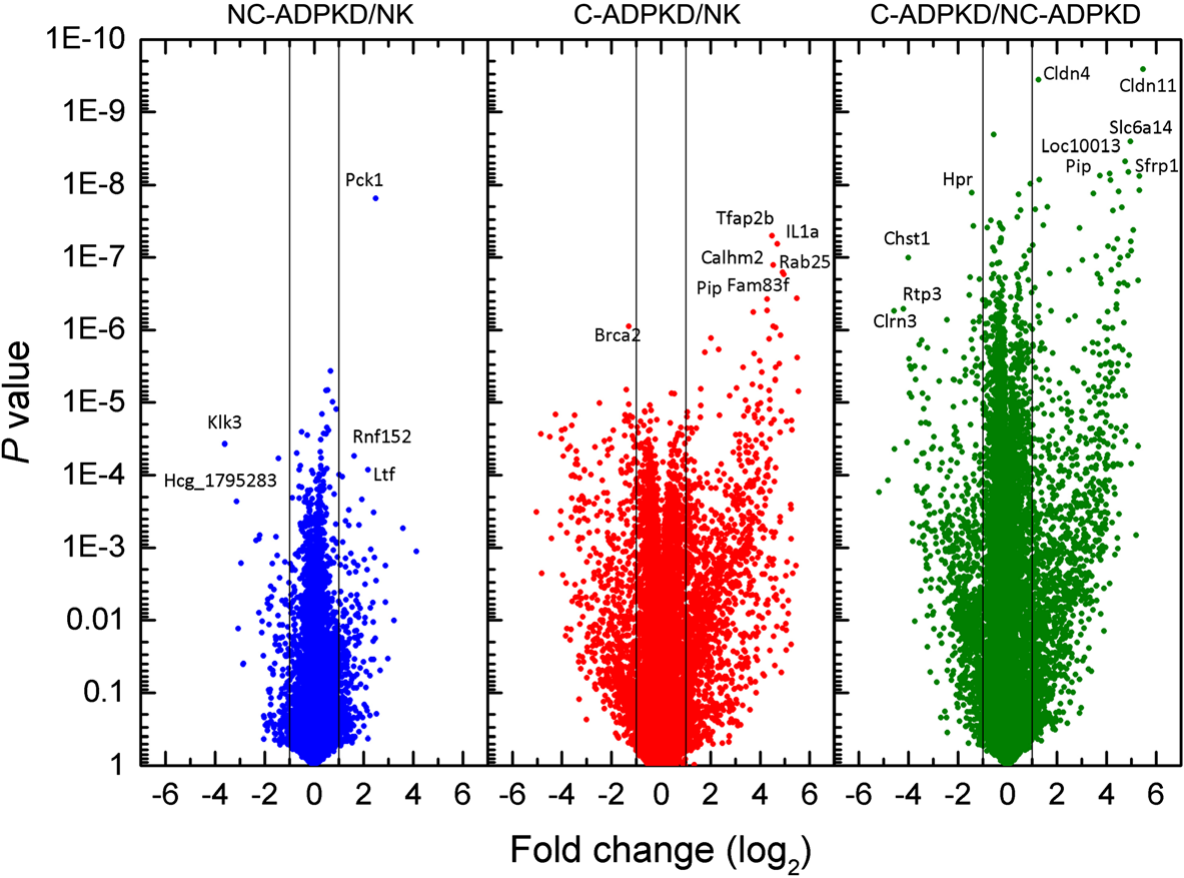
Volcano plot of single gene analysis. Plots of log *P* value versus log_2_ fold change for NC-ADPKD/NK, C-ADPKD/NK, and NC-ADPKD/C-ADPKD cells. The two *vertical lines* in each panel mark twofold change. *Labels* indicate representative genes with highly significant fold changes. See Additional file 1 for full lists of significantly changed genes

Ingenuity pathway analysis scores each gene independently to create a list of all genes which change significantly in expression between two states. We averaged gene expression levels over the replicates for each cell line after normalization and compared cell lines by calculating the fold change (FC) as the ratio between the averages. We also obtained *P* values for each gene expressed. We present the results as Volcano plots (Fig. 2). Setting a significance threshold of FC > 2 and *P* < 0.01, we found 80 genes with significantly altered expression levels between the NK and NC-ADPKD cells, 968 genes with significantly altered expression levels between the NK and C-ADPKD cells, and 1182 genes with significantly altered expression levels between the C-ADPKD and NC-ADPKD cells (Additional file 1). These long lists of differentially expressed genes, by themselves, do not provide immediate insights into the biological mechanisms of ADPKD.

### GSEA: Gene Set Enrichment Analyses

GSEA determines whether an a priori defined set of genes shows statistically significant, concordant differences between two biological states [26, 32, 33]. GSEA evaluates genome-wide expression profiles from cells belonging to two classes. We performed GSEA on the three pairs: NK versus NC-ADPKD cells, NK versus C-ADPKD cells, and NC-ADPKD versus C-ADPKD cells to identify differential expression in Kyoto Encyclopedia of Genes and Genomes (KEGG) pathways and in Gene Ontology: Biological Process (GO) terms. We set a significance threshold for the gene set permutation FDR **<**0.05. Table 1 summarizes the GSEA results and Table **2** lists all significantly altered KEGG pathway and GO term gene sets.

**Table 1:** GSEA analysis summary

GSEA summary for NC-ADPKD/NK						
Upregulated in		# gene sets	FDR < 0.25	FDR < 0.05	*P* < 0.05	*P* < 0.01
NK	KEGG	162	15	**5**	11	7
	GOBP	467	0	0	6	1
NC-ADPKD	KEGG	162	0	0	20	3
	GOBP	467	10	0	41	18
GSEA summary for C-ADPKD/NK						
Upregulated in		# gene sets	FDR < 0.25	FDR < 0.05	*P* < 0.05	*P* < 0.01
NK	KEGG	162	2	0	8	0
	GOBP	467	0	0	11	2
C-ADPKD	KEGG	162	15	**3**	17	12
	GOBP	467	1	**1**	46	13
GSEA summary for NC-ADPKD/C-ADPKD						
Upregulated in		# gene sets	FDR < 0.25	FDR < 0.05	*P* < 0.05	*P* < 0.01
NC-ADPKD	KEGG	162	11	0	10	4
	GOBP	467	17	0	24	13
C-ADPKD	KEGG	162	11	**4**	18	10
	GOBP	467	3	**1**	42	15

Numbers of gene sets that reach significance in comparisons of two GSEA classes, FDR < 0.05, are bold. *FDR* false discovery rate, *P* nominal *P* value, as described by Subramanian et al. 2005 [26]. GSEA expresses all differences as upregulation to minimize class bias due to the intrinsic asymmetry of the method

**Table 2:** KEGG pathways and GO terms identified as differentially expressed by GSEA analysis

	Size	ES	NES	Normal *P* value	FDR	FWER *P* value
NC-ADPKD/NK, upregulated in NK						
KEGG_drug_metabolism_cytochrome_p450	42	0.8	2.17	0	0	0
KEGG_metabolism_of xenobiotics_by_cytochrome p450	42	0.7	2.06	0	0	0.001
KEGG_histidine_metabolism	22	0.8	1.94	0	0.006	0.019
KEGG_glutathione_metabolism	38	0.7	1.86	0	0.013	0.049
KEGG_fatty_acid_metabolism	32	0.6	1.73	0.004	0.048	0.209
C-ADPKD/NK, upregulated in C-ADPKD						
KEGG_chemokine_signaling_pathway	144	−1	−1.73	0	0.01	0.011
KEGG_axon_guidance	99	−1	−1.68	0	0.03	0.066
KEGG_leukocyte_transendothelial_migration	86	−1	−1.66	0.001	0.028	0.092
GOBP_cell_cell_adhesion	48	0.8	1.8	0	0.001	0.001
NC-ADPKD/C-ADPKD, upregulated in C-ADPKD						
KEGG_leukocyte_transendothelial_migration	86	−1	−1.82	0	0	0
KEGG_chemokine_signaling_pathway	144	−1	−1.74	0	0.024	0.05
KEGG_tight_junction	101	−1	−1.73	0	0.02	0.063
KEGG_axon_guidance	99	−1	−1.73	0	0.016	0.066
GOBP_cell_cell_adhesion	48	0.8	1.8	0	0.007	0.008

Listing of KEGG pathways and GO terms with FDR < 5 %. GSEA expresses all differences as upregulation to minimize class bias due to the intrinsic asymmetry of the method

All GSEA-detected pathway differences between NK and NC-ADPKD cells related to ‘metabolism’ (We will use single ‘’ to identify terms from GO and KEGG). GSEA-detected pathway differences between NK and C-ADPKD cells relate to ‘chemokine signaling,’ ‘cell migration,’ and ‘cell-cell adhesion,’ but not ‘metabolism’. GSEA-detected pathway differences between NC-ADPKD and C-ADPKD cells relate to ‘chemokine signaling,’ ‘cell migration,’ and ‘cell-cell adhesion’ but not ‘metabolism’. However, transcriptogram analyses (see below) show that expression of metabolome genes differs between these pairs as well, but too weakly for GSEA to detect, likely due to the competitive character of GSEA analysis [33].

### Transcriptograms: genome-wide landscapes of gene expression

**Fig. 3:**
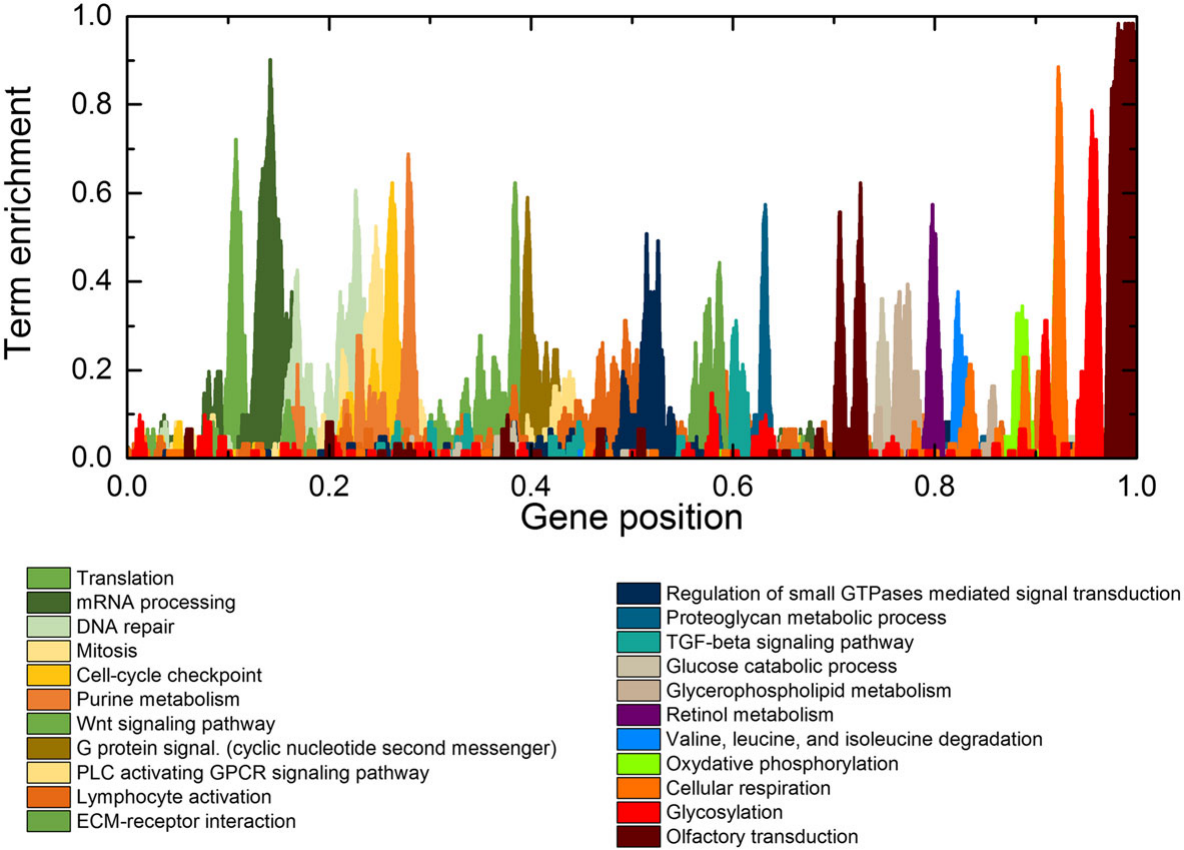
Term enrichment map showing the correspondence between the ordered gene list and major biological terms and pathways. The *x*-axis indicates the relative position in the ordered list of 9684 genes. The colored profiles give the density distributions within the list of specific GO terms or KEGG pathways. A term enrichment value of 1 on the *y*-axis indicates that all genes in an interval of radius *r* = 30 participate in a given GO term or KEGG pathway. *Peaks* mark regions enriched with genes related to the term or pathway indicated in the legend. The legend orders the terms/pathways from left to right

Transcriptograms project expression profiles onto a gene list that is ordered with genes belonging to the same KEGG pathway or GO term clustered in the list. We built an ordered list of 9684 genes (Additional file 2) using a Monte Carlo Simulation that minimizes distances between associated gene products. Specifically, proximity in the list correlates with co-participation in biological processes, based on protein-protein association information available from the STRING database [34, 35]. Transcriptogram values then correspond to the average expression level of the genes located in a window of radius *r* = 30 (window size 2*r* + 1 = 61) along the ordered list. Overlay of selected GO terms and KEGG pathways onto the ordered list then serves as a key to the biological correlates of expression differences and thus aids interpretation of transcriptograms of experimental gene expression data (Fig. 3). The *x*-axis corresponds to the ordered gene list. Peaks represent regions of the list enriched with genes belonging to specific pathways or terms. We normalize the density profiles of term enrichment so that when all genes within a window participate in that pathway or term, the center point of that interval has a value of one. This paper presents transcriptograms as three-part panels, with the transcriptogram itself in the upper third of the panel, the *P* values for the transcriptogram in the middle third of the panel (with horizontal lines indicating *p* = 0.05 and 0.01), and plots of peaks from the term enrichment density profile that correspond to significant peaks in the transcriptogram in the lower third of the panel. We calculated FDR values for all comparisons (Table 3).

**Table 3:** False discovery rates

False discovery rates			
*P* value	NC-ADPKD/NK	C-ADPKD/NK	C-ADPKD/NC-ADPKD
0.1	0.131545	0.140392	0.0970966
0.05	0.137393	0.120033	0.0869387
0.01	0.100738	0.112763	0.0809876
0.005	0.100182	0.1156	0.0804989
0.001	0	0.106	0.0800862
0.0005	0	0	0.0800589
0.0001	0	0	0.08
5.00E−05	0	0	0.08

False discovery rates (FDR) for relative transcriptograms obtained using 500 sample-label permutations

**Fig. 4:**
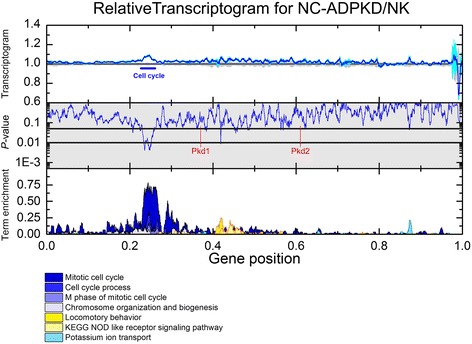
Relative transcriptogram for NC-ADPKD/NK. The *x*-axis indicates the relative positon in the ordered list of 9684 genes. *Upper panel* the relative transcriptograms are presented as means ± s.e.m. with NC-ADPKD (*blue line* ± *cyan region*) and NK (*black line* ± *gray region*) given relative to NK. *Center panel* (*shaded gray*): *P* value from a two-tailed Weyl’s *t* test is plotted for each point of the transcriptograms. *Horizontal lines* mark *P* = 0.05 and *P* = 0.01. Location in the ordered list of Pkd1 and Pkd2, the causative genes for ADPKD, is shown. *Lower panel* Term enrichment for transcriptogram regions with significantly changed expression. A term enrichment value of 1 on the *y*-axis indicates that all genes in an interval of radius *r* = 30 participate in a given GO term or KEGG pathway. As an example of how to read the relative transcriptogram figures, NC-ADPKD cells have higher-than-normal gene expression in the region of the peak labeled ‘cell cycle’ in the *upper panel* with a significance of *P* < 0.01 (*middle panel*). This region is enriched with genes associated with ‘cell cycle’ KEGG terms and GO pathways (marked with a *bar* and identified by referring to the *lower panel*, which shows term enrichment)

Using transcriptogram analysis, we first compared the expression profiles of NK and NC-ADPKD samples (Fig. 4). Across most of the ordered list, the standard errors of the expression levels of genes for the NK and NC-ADPKD cells overlap, showing that gene expression levels of these genes in NC-ADPKD cells do not differ significantly from normal. However, gene positions between 0.22 and 0.26 and the intervals centered at positions 0.46 and 0.88 show significant differences (*P* < 0.01 and FDR < 0.12). The term enrichment profile shows that these significant peaks correspond to ‘cell cycle,’ ‘locomotory behavior,’ ‘NOD (nucleotide-binding oligomerization domain)-like receptor signaling,’ and ‘Potassium ion transport’ (Fig. 4). When we analyzed the gene lists from each interval of differential expression to identify additional pathways and terms, we found additional differentially expressed gene sets related to ‘cell cycle,’ ‘nucleotide metabolism,’ ‘genetic information processing,’ ‘immune response,’ and ‘ion transport’ (Figs. 7, 8, 9, 11, 13). We examined individual gene expression profiles from two of these pathways, ‘NOD-like receptors’ and ‘potassium ion transport’. Genes differentially expressed at significant levels (Figs. 12a, 11b) may be potential targets for drug interventions to prevent or ameliorate very early stages of cyst growth.

**Fig. 5:**
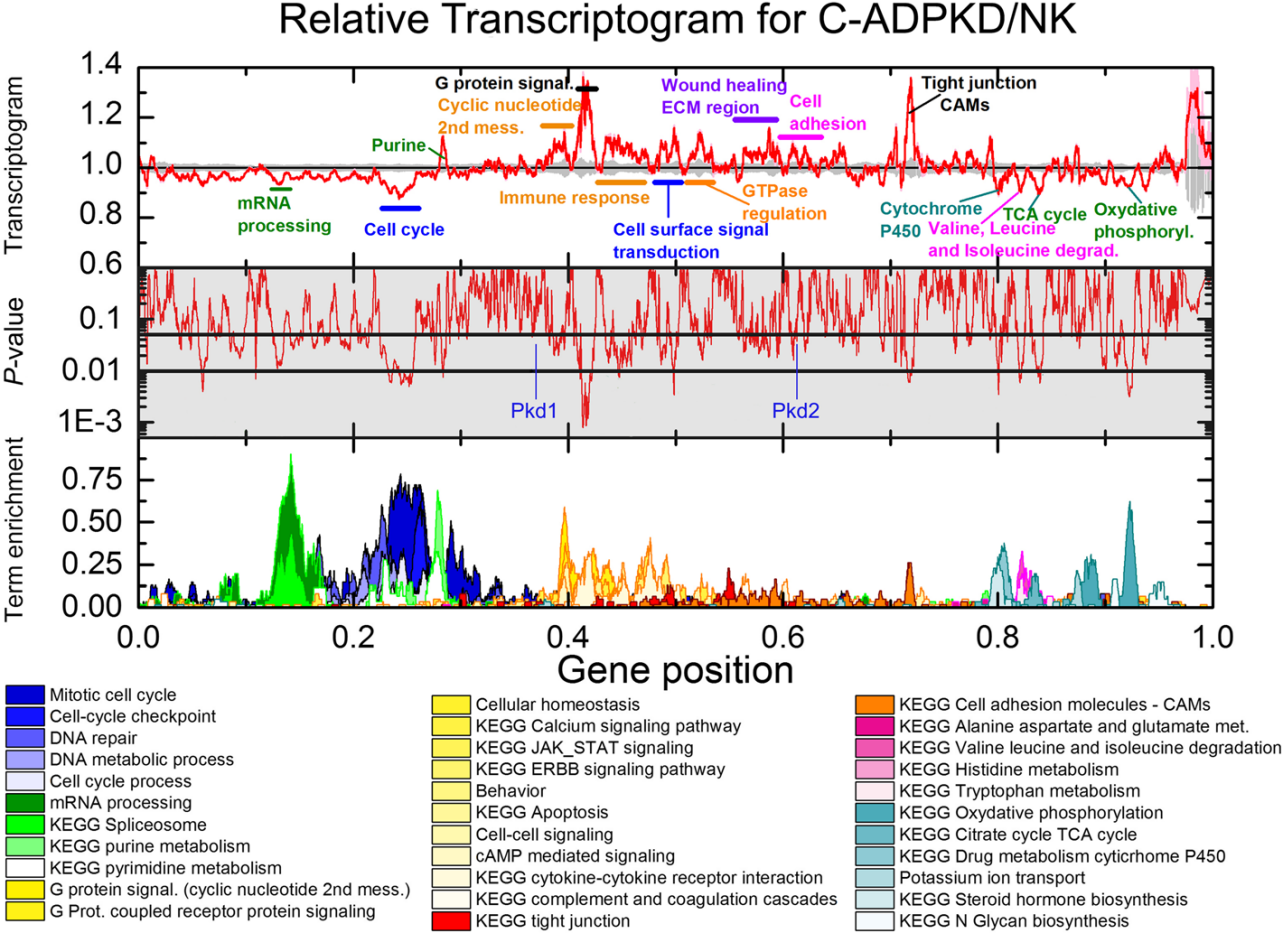
Relative transcriptogram for C-ADPKD/NK. The *x*-axis indicates the relative position in the ordered list of 9684 genes. *Upper panel* We present the relative transcriptograms as means ± s.e.m. with C-ADPKD (*red line* ± *pink region*) and NK (*black line* ± *gray region*) given relative to NK. *Center panel* (*shaded gray*): *P* value from a two-tailed Weyl’s *t* test for each point of the transcriptogram. *Horizontal lines* mark *P* = 0.05 and *P* = 0.01. We label the location in the ordered list of Pkd1 and Pkd2, the causative genes for ADPKD. *Lower panel* Term enrichment for transcriptogram regions with gene expression significantly different from normal. A term enrichment value of 1 on the y-axis indicates that all genes in an interval of radius *r* = 30 participate in a given GO term or KEGG pathway

Transcriptogram analysis comparing the NK and C-ADPKD samples (Fig. 5) detected additional regions of significant difference (where *P* < 0.01 and FDR < 0.12). The term enrichment profile shows that these significant peaks correspond to alterations in ‘RNA processing,’ ‘cell cycle,’ ‘nucleic acid processing and metabolism,’ ‘G-protein signaling,’ ‘immune response,’ and ‘cell fate’ related pathways including ‘Calcium signaling’ and ‘JAK-STAT’ pathways, ‘wound healing,’ ‘extracellular matrix,’ ‘cell adhesion and tight junctions,’ as well as energy-related gene sets for ‘oxidative phosphorylation’ and the ‘TCA (tricarboxylic acid cycle) cycle,’ and the ‘drug-metabolism related cytochrome P450’. Analysis of the gene lists from each interval of differential expression identified additional related pathways and terms (Figs. 7, 8, 9, 10, 11, 12, 13, and 14). Overall, C-ADPKD cells have lower-than-normal metabolic rates and lower-than-normal expression levels of genes associated with ‘cell proliferation,’ ‘RNA (ribonucleic acid) metabolism,’ and ‘sugar, lipid and energy metabolism’. Additionally, their interaction with the extracellular environment is strongly altered as indicated by enhanced expression of ‘cell adhesion,’ ‘tight junction,’ and ‘cell surface signaling’ pathways. Thus, C-ADPKD cells have abnormal interactions with their extracellular environment.

Although cell cycle regulation is abnormal in both NC-ADPKD and C-ADPKD cells, expression of cell-cycle-related genes is higher-than-normal in NC-ADPKD samples and lower-than-normal in C-ADPKD samples. Additional pathways and terms identified from gene lists of the cell cycle or RNA metabolism-associated intervals (Figs. 7, 8, and 9) present a consistent pattern: expression in the NC-ADPKD sample is higher than normal and expression in C-ADPKD sample is lower than normal. This pattern of cell-cycle regulation disruption deserves future investigation since the genes included in this gene set initiate and regulate cell cycle progression making it difficult to predict the net effect on cell proliferation.

**Fig. 6:**
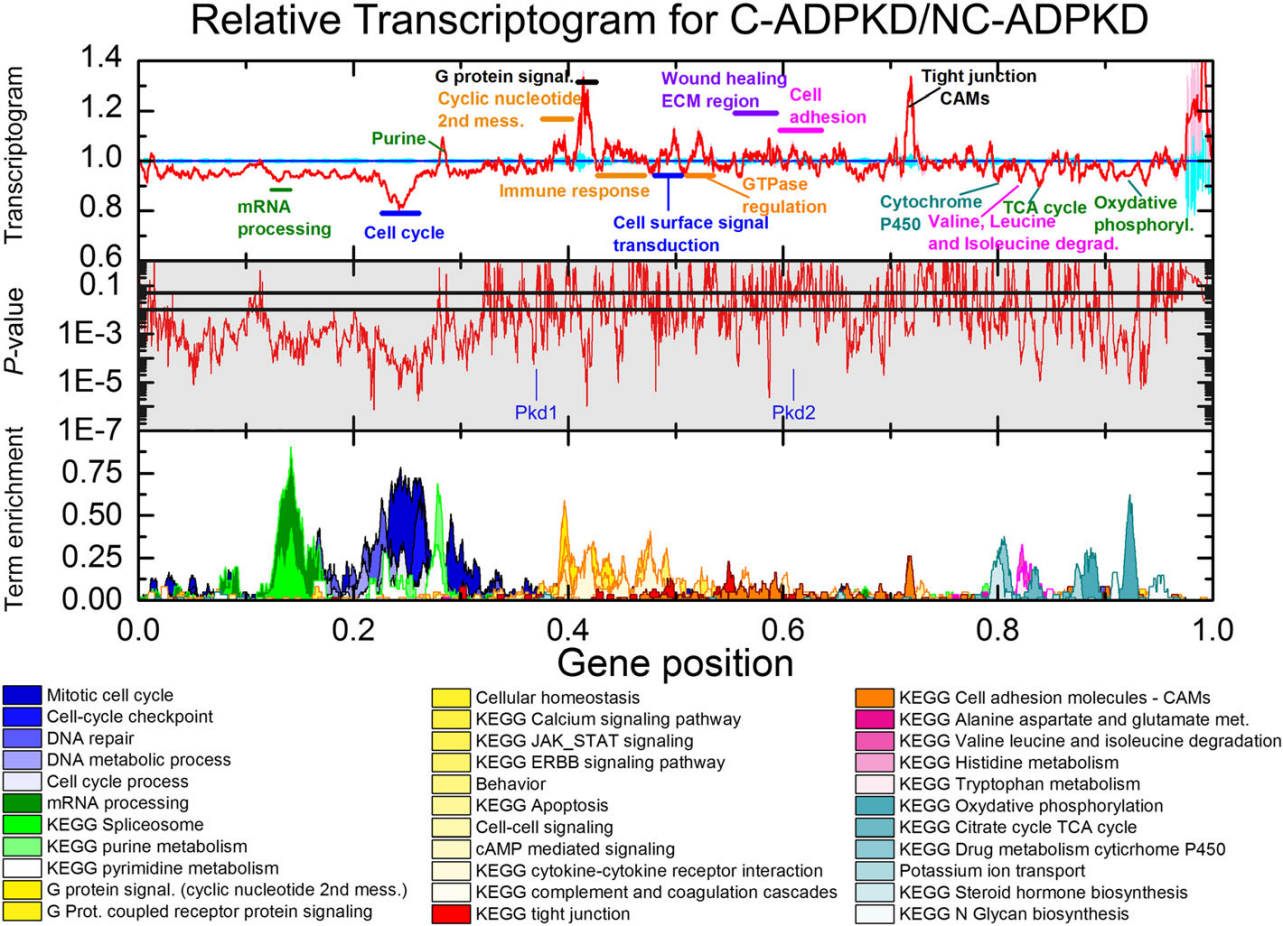
Relative transcriptogram for C-ADPKD/NC-ADPKD. The *x*-axis indicates the relative positon in the ordered list of 9684 genes. *Upper panel* The relative transcriptograms are presented as means ± s.e.m. with C-ADPKD (red line ± pink region) and NC-ADPKD (blue line ± cyan region) relative to NC-ADPKD. Center panel (shaded gray): *P* value from a two tailed Weyl’s *t* test for each point of the transcriptogram. Horizontal lines mark *P* = 0.05 and *P* = 0.01. We label the location in the ordered list of Pkd1 and Pkd2, the causative genes for ADPKD. Lower panel: Term enrichment for transcriptogram regions with gene expression significantly different from normal. A term enrichment value of 1 on the *y*-axis indicates that all genes in an interval of radius *r* = 30 participate in a given GO term or KEGG pathway. *P* values between C-ADPKD and NC-ADPKD cells are smaller than between either C-ADPKD or NC-ADPKD and NK cells (see Figs. 4 and 5) because C-ADPKD and NC-ADPKD cells were derived from the same individual, allowing us to discriminate additional differentially expressed regions

**Fig. 7:**
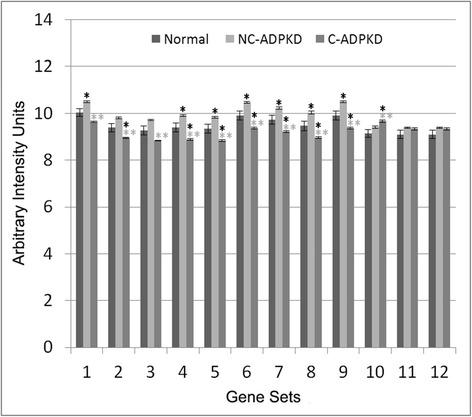
Both cell growth and cell death are altered in ADPKD. NC-ADPKD cells have higher-than-normal expression and C-ADPKD cells have lower-than-normal expression of genes from ‘cell growth’ gene sets. Both NC-ADPKD and C-ADPKD cells have higher-than-normal expression of genes from ‘cell death’ gene sets. We present data as mean ± s.e.m., **P < 0.01, *P < 0.05 (*black asterisk* is in comparison to NK, *gray asterisk* is in comparison to NC-ADPKD). *Cell Growth Gene Sets:* (1) KEGG_CELL_CYCLE, (2) CELL_CYCLE, (3) CELL_CYCLE_CHECKPOINT, (4) CELL_CYCLE_PROCESS, (5) CELL_CYCLE_PHASE, (6) MITOTIC_M_PHASE, (7) MITOTIC_CELL_CYCLE, (8) M_PHASE, (9) MITOSIS*. Cell Death Gene Sets:* (10) KEGG_APOPTOSIS, (11) APOPTOTIC_PROCESS, (12) PROGRAMMED_CELL_DEATH

**Fig. 8:**
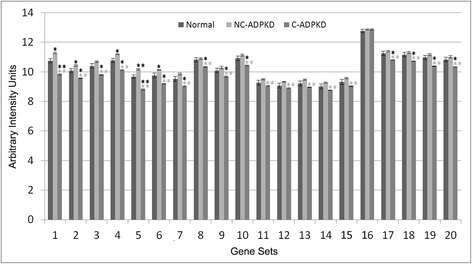
Genetic information processing is altered in ADPKD. NC-ADPKD cells have higher-than-normal expression and C-ADPKD cells have lower-than-normal expression of genes from all ‘Genetic information processing’ gene sets except the KEGG ribosome gene set. We present data as mean ± s.e.m., **P < 0.01, *P < 0.05 (*black asterisk* is in comparison to NK, *gray asterisk* is in comparison to NC-ADPKD). *Genetic Information Processing Gene Sets:* (1) KEGG_DNA_REPLICATION, (2) KEGG_BASE_EXCISION_REPAIR, (3) KEGG_NUCLEOTIDE_EXCISION_REPAIR, (4) KEGG_MISMATCH_REPAIR, (5) KEGG_HOMOLOGOUS_RECOMBINATION, (6) DNA_REPLICATION, (7) DNA_REPAIR, (8) KEGG_RNA_POLYMERASE, (9) KEGG_BASAL_TRANSCRIPTION_FACTORS, (10) KEGG_SPLICEOSOME, (11) TRANSCRIPTION, (12) REGULATION_OF_TRANSCRIPTION, (13) TRANSCRIPTION_DNA_DEPENDENT, (14) REGULATION_OF_TRANSCRIPTION_DNA_DEPENDENT, (15) TRANSCRIPTION_FROM_RNA_POLYMERASE_II_PROMOTER, (16) KEGG_RIBOSOME, (17) RIBONUCLEOPROTEIN_COMPLEX_BIOGENESIS_AND_ASSEMBLY, (18) PROTEIN_RNA_COMPLEX_ASSEMBLY, (19) MRNA_PROCESSING_GO_0006397, (20) RNA_SPLICING_VIA_TRANSESTERIFICATION_REACTIONS

**Fig. 9:**
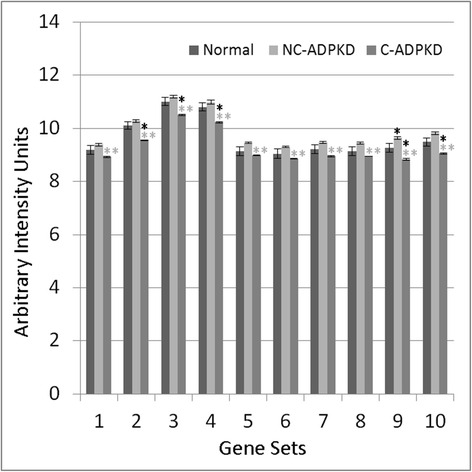
Nucleotide metabolism is abnormal in ADPKD. NC-ADPKD cells have higher-than-normal expression and C-ADPKD cells have lower-than-normal expression of genes from all ‘Nucleotide Metabolism’ gene sets. We present data as mean ± s.e.m., **P < 0.01, *P < 0.05 (*black asterisk* is in comparison to NK, *gray asterisk* is in comparison to NC-ADPKD). *Nucleotide Metabolism Gene Sets:* (1) KEGG_PURINE_METABOLISM, (2) KEGG_PYRIMIDINE_METABOLISM, (3) RNA_SPLICING, (4) MRNA_METABOLIC_PROCESS, (5) NEGATIVE_REGULATION_OF_TRANSCRIPTION, (6) REGULATION OF NUCLEOBASE_NUCLEOSIDE_NUCLEOTIDE_AND_NUCLEIC_ACID_METABOLIC_PROCESS, (7) RNA_BIOSYNTHETIC_PROCESS, (8) NEGATIVE_REGULATION_OF NUCLEOBASE_NUCLEOSIDE_NUCLEOTIDE_AND_NUCLEIC_ACID_METABOLIC_PROCESS, (9) CHROMOSOME_ORGANIZATION_AND_BIOGENESIS, (10) DNA_METABOLIC_PROCESS

**Fig. 10:**
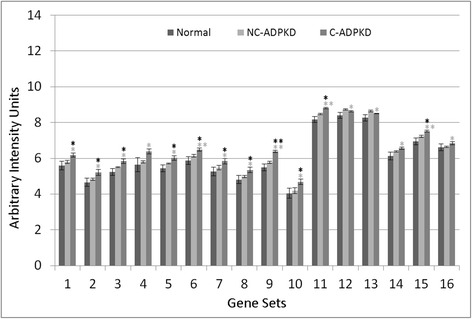
ADPKD upregulates signal transduction. NC-ADPKD and C-ADPKD cells have higher-than-normal expression of genes from all signal transduction gene sets. Expression levels in NC-ADPKD cells are intermediate between those of NK and C-ADPKD cells, exception that expression levels of Wnt and Notch signaling pathways are higher in NC-ADPKD cells. These pathways are normally active primarily during embryonic development. We present data as mean ± s.e.m., **P < 0.01, *P < 0.05 (*black asterisk* is in comparison to NK, *gray asterisk* is in comparison to NC-ADPKD). *Signal Transduction Gene Sets:* (1) G_PROT._COUPLED_RECEPTOR_PROTEIN_SIGNALING, (2) G_PROT._SIGNALING_COUPLED_TO_CYCLIC_NUCLEOTIDE_2ND_MESS, (3) G_PROT._SIGNALING_COUPLED_TO_CAMP_NUCLEOTIDE_2ND_MESS, (4) G_PROT._SIGNALING_ADENYLATE_CYCLASE_ACTIVATING, (5) CAMP_MEDIATED_SIGNALING, (6) CELL_CELL_SIGNALING, (7) SYNAPTIC_TRANSMISSION, (8) CYCLIC_NUCLEOTIDE_MEDIATED_SIGNALING, (9) KEGG_CYTOKINE_CYTOKINE_RECEPTOR_INTERACTION, (10) KEGG_NEUROACTIVE_LIGAND_RECEPTOR_INTERACTION, (11) KEGG_ERBB_SIGNALING_PATHWAY, (12) KEGG_WNT_SIGNALING_PATHWAY, (13) KEGG_NOTCH_SIGNALING_PATHWAY, (14) KEGG_CALCIUM_SIGNALING_PATHWAY, (15) KEGG_JAK_STAT_SIGNALING_PATHWAY, (16) KEGG_ABC_TRANSPORTERS

We then used transcriptogram analysis to compare NC-ADPKD and C-ADPKD samples (Fig. 6). Because NC-ADPKD and NK samples have very similar expression profiles (Fig. 4), the transcriptogram comparison of NC-ADPKD to C-ADPKD samples is similar to the transcriptogram comparison of NK to C-ADPKD samples. However, because NC-ADPKD and C-ADPKD samples derive from cystic and non-cystic tissues from the same kidney, standard errors and consequently, *P* values for this comparison are very small. However, the location of the most significant peaks and valleys are nearly identical in both comparisons. Many of the pathways and terms differentially expressed between NC-ADPKD and C-ADPKD samples (Figs. 6, 7, 8, 9, and 10) are also differentially expressed between NK and C-ADPKD samples.

### Gene sets associated with significantly altered transcriptogram intervals

**Fig. 11:**
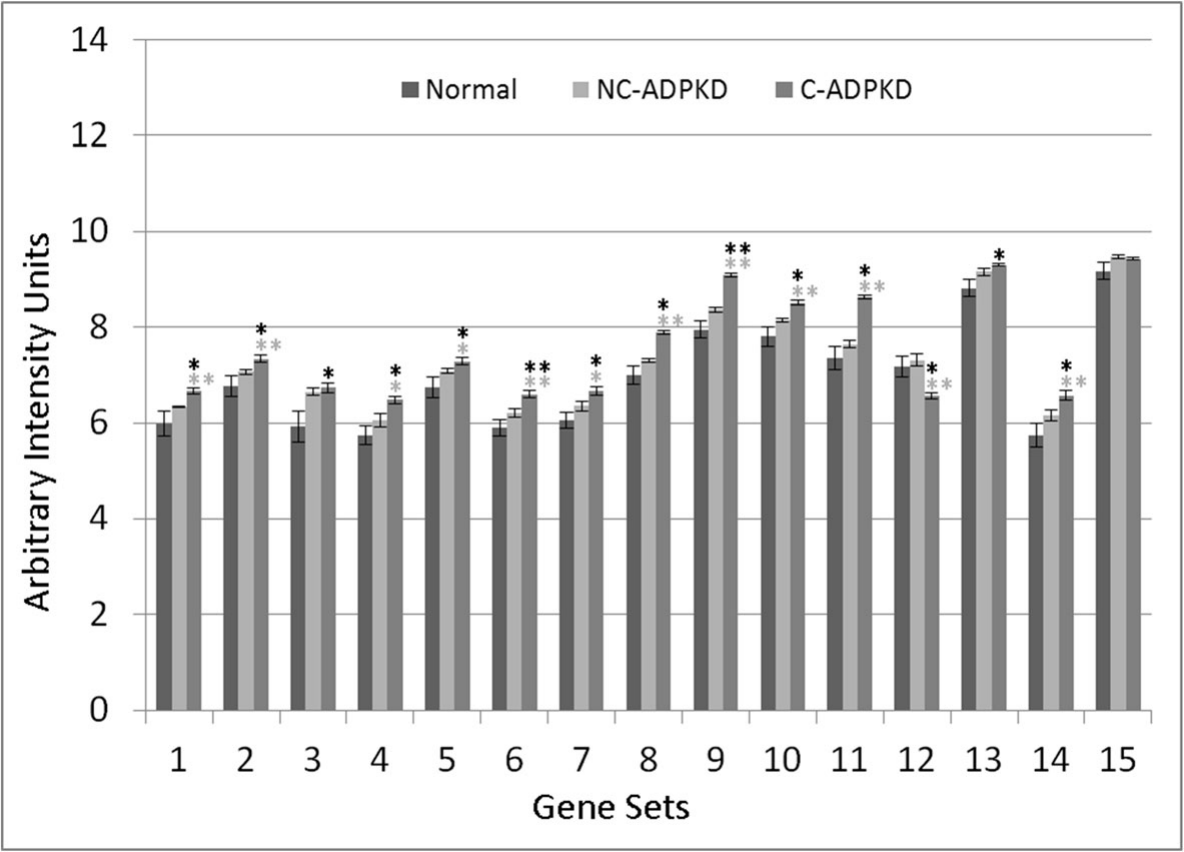
Immune response is abnormal in ADPKD. NC-ADPKD and C-ADPKD cells both express higher-than-normal levels of genes from ‘immune response’ gene sets, exception that C-ADPKD cells have lower-than-normal expression of the KEGG ‘Complement and Coagulation cascade’ gene set (12). We present data as mean ± s.e.m., **P < 0.01, *P < 0.05 (*black asterisk* is in comparison to NK, *gray asterisk* is in comparison to NC-ADPKD). *Immune Response Gene Sets:* (1) INFLAMMATORY_RESPONSE, (2) IMMUNE_RESPONSE, (3) HUMORAL_IMMUNE_RESPONSE, (4) CELL_ACTIVATION, (5) IMMUNE_SYSTEM_PROCESS, (6) LEUKOCYTE_ACTIVATION, (7) LYMPHOCYTE_ACTIVATION, (8) KEGG_CHEMOKINE_SIGNALING_PATHWAY, (9) KEGG_NOD_LIKE_RECEPTOR_SIGNALING_PATHWAY, (10) KEGG_T_CELL_RECEPTOR_SIGNALING_PATHWAY, (11) KEGG_LEUKOCYTE_TRANSENDOTHELIAL_MIGRATION, (12) KEGG_COMPLEMENT_AND_COAGULATION_CASCADES, (13) KEGG_CYTOSOLIC_DNA_SENSING_PATHWAY, (14) KEGG_HEMATOPOIETIC_CELL_LINEAGE, (15) KEGG_FC_GAMMA_R_MEDIATED_PHAGOCYTOSIS

**Fig. 12:**
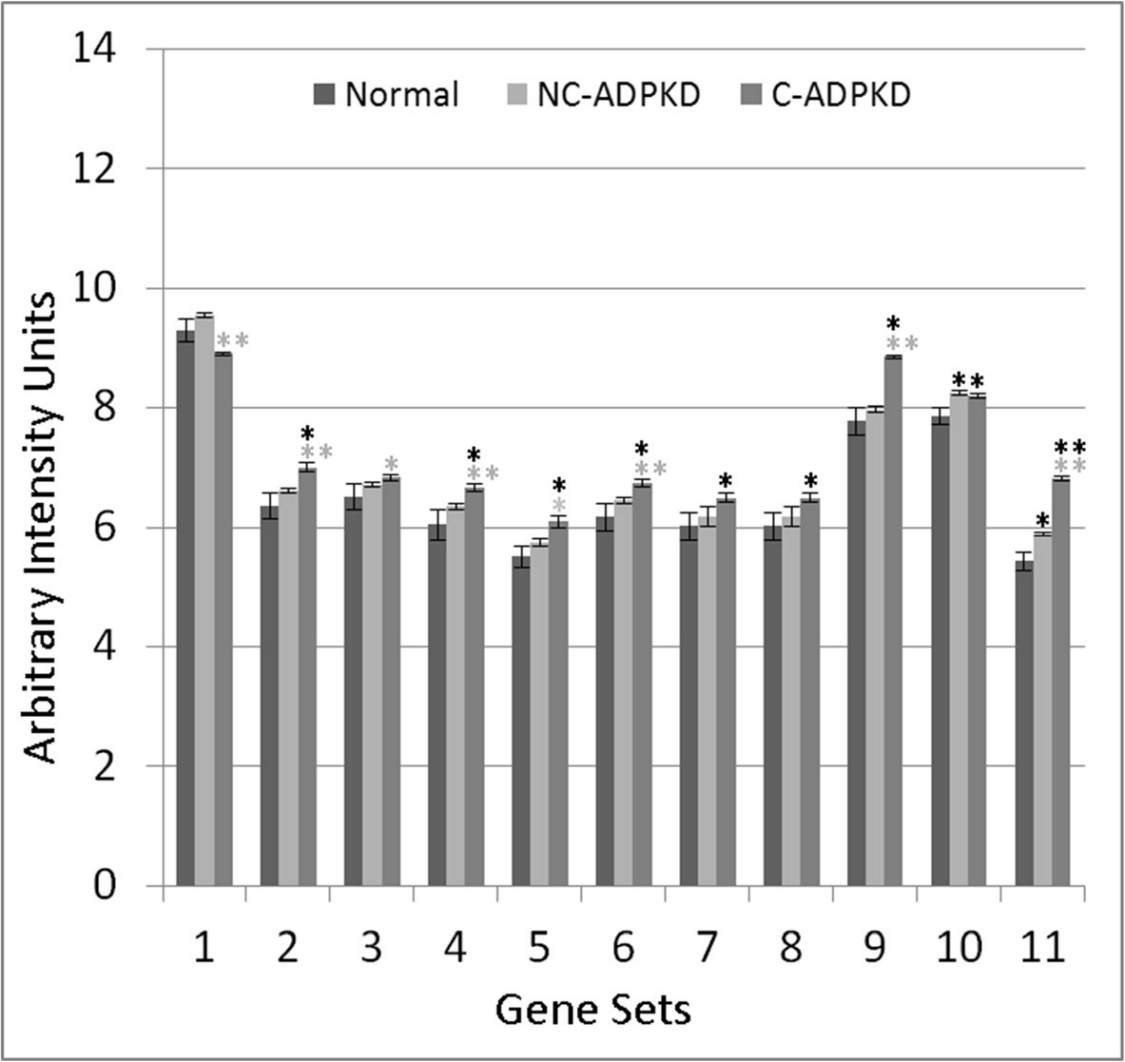
Response to stimulus and cellular processes are abnormal in ADPKD. Both NC-ADPKD and C-ADPKD cells have higher-than-normal expression of genes in the ‘response to stimulus’ gene sets (1–8), except that C-ADPKD cells have lower-than-normal expression of genes in the ‘response to endogenous stimulus’ gene set. C-ADPKD cells have higher-than-normal expression of genes in the ‘cellular processes’ gene sets (9–11). Data are represented as mean ± s.e.m., **P < 0.01, *P < 0.05 (*black asterisk* is in comparison to NK, *gray asterisk* is in comparison to NC-ADPKD). *Response to Stimulus Gene Sets:* (1) RESPONSE_TO_ENDOGENOUS_STIMULUS, (2) RESPONSE_TO_EXTERNAL_STIMULUS, (3) REGULATION_OF_RESPONSE_TO_STIMULUS, (4) DEFENSE_RESPONSE, (5) CELLULAR_DEFENSE_RESPONSE, (6) RESPONSE_TO_WOUNDING, (7) COAGULATION, (8) BLOOD_COAGULATION. *Cellular Processes Gene Sets:* (9) KEGG_TIGHT_JUNCTION, (10) KEGG_GAP_JUNCTION, (11) LOCOMOTORY_BEHAVIOR

**Fig. 13:**
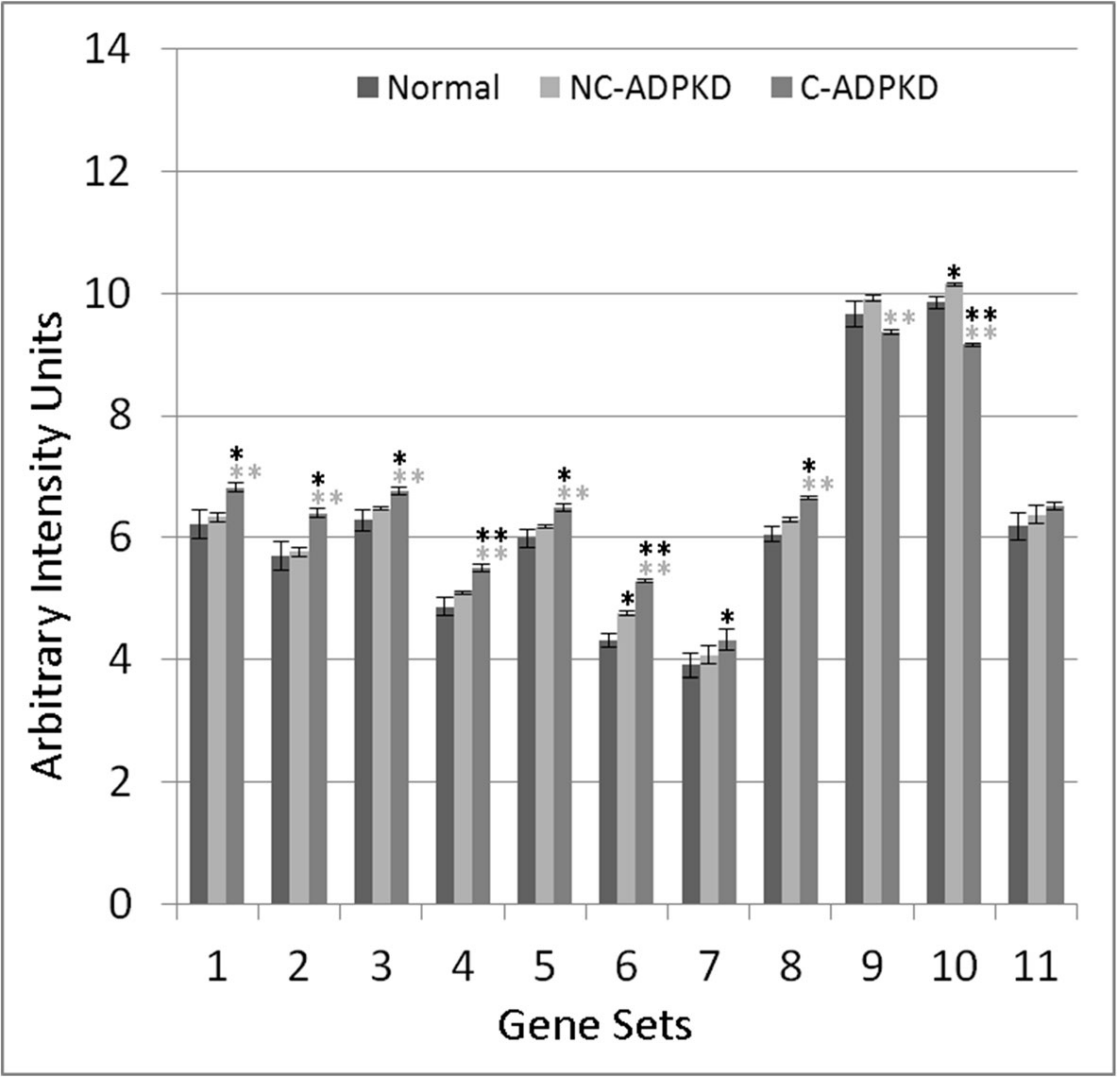
Ion homeostasis and transport are abnormal in ADPKD. NC-ADPKD cells have higher-than-normal and C-ADPKD cells have much higher-than-normal expression of genes in ‘ion homeostasis’ gene sets (1–8). NC-ADPKD cells have higher-than-normal expression of genes in ‘transport’ gene sets (9–11). We represent data as mean ± s.e.m, **P < 0.01, *P < 0.05 (*black asterisk* is in comparison to NK, *gray asterisk* is in comparison to NC-ADPKD). *Ion Homeostasis Gene Sets:* (1) ION_HOMEOSTASIS, (2) CELLULAR_CATION_HOMEOSTASIS, (3) ION_TRANSPORT, (4) METAL_ION_TRANSPORT, (5) CATION_TRANSPORT, (6) POTASSIUM_ION_TRANSPORT, (7) CALCIUM_ION_TRANSPORT, (8) MONOVALENT_INORGANIC_CATION_TRANSPORT. *Transport Gene Sets:* (9) KEGG_VASOPRESSIN_REGULATED_WATER_REABSORPTION, (10) KEGG_PROXIMAL_TUBULE_BICARBONATE_RECLAMATION, (11) REGULATION_OF_BODY_FLUID_LEVELS

**Fig. 14:**
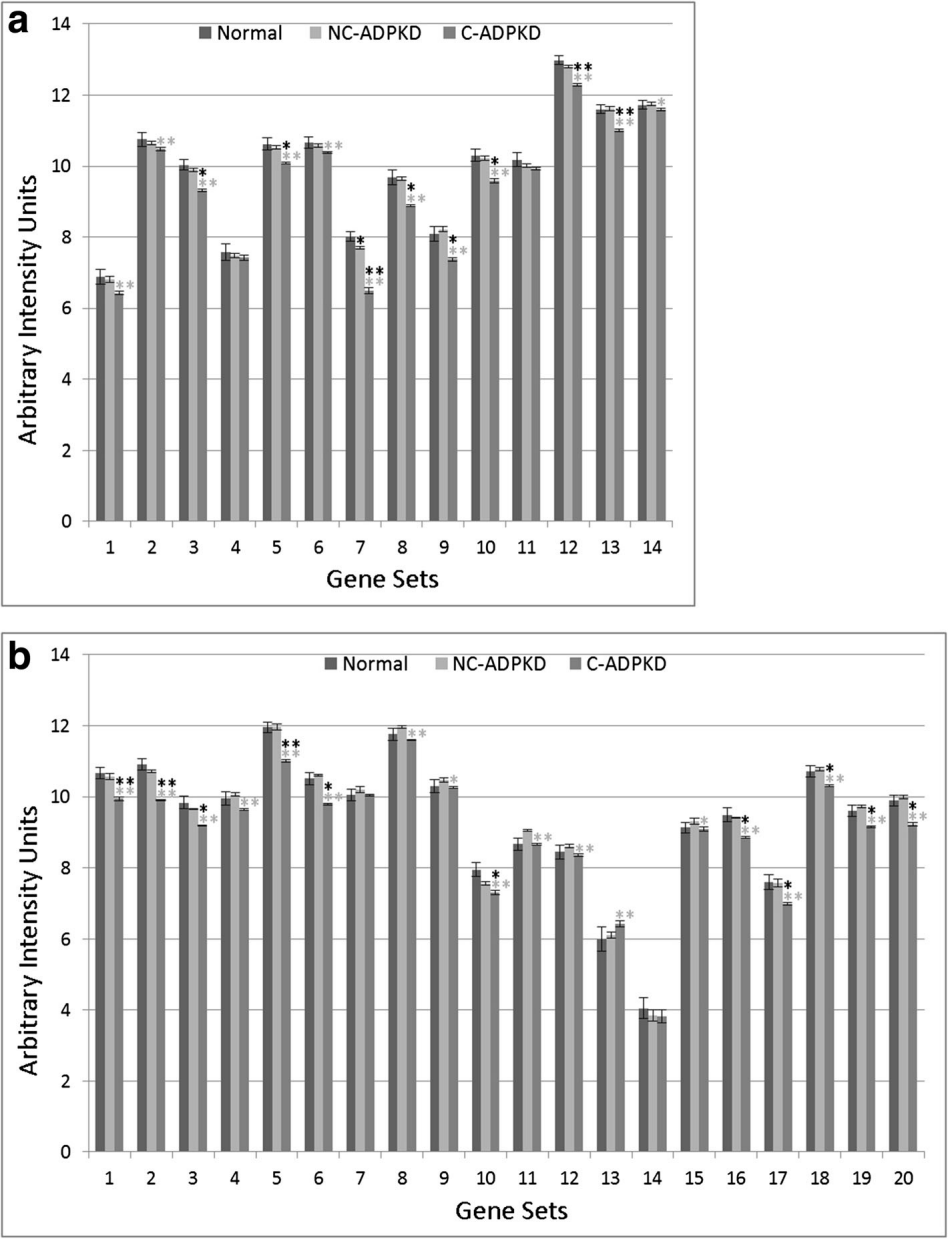
ADPKD broadly perturbs metabolism. **a** Cofactor and vitamin, amino acid, and energy metabolism and **b** carbohydrate, drug, lipid, and glycan metabolism. C-ADPKD cells have lower-than-normal expression of genes in ‘cofactor and vitamin’ gene sets (A1-2) compared to both NK and NC-ADPKD cells. Both NC-ADPKD and C-ADPKD cells have lower-than-normal expression of genes in ‘amino acid’ gene sets (A3-11). C-ADPKD cells have lower-than-normal expression of genes in ‘energy metabolism’ gene sets (A12-14). C-ADPKD cells have lower-than-normal expression of genes in all ‘carbohydrate metabolism’ gene sets (B1-9) except for the KEGG ‘histidine metabolism’ gene set. C-ADPKD cells have lower-than-normal expression of genes in the ‘drug metabolism’ gene set (B10). C-ADPKD cells have lower-than-normal expression of genes in all ‘lipid metabolism’ (B11-18) gene sets except for the KEGG ‘arachidonic acid metabolism’ gene set (B13). C-ADPKD cells have lower-than-normal expression of genes in ‘glycan metabolism’ gene sets (B19-20). We present data as mean ± s.e.m., **P < 0.01, *P < 0.05 (*black asterisk* is in comparison to NK, *gray asterisk* is in comparison to NC-ADPKD). **a** Cofactor and vitamin, amino acid, and energy metabolism gene sets. *Cofactor and Vitamin Gene Sets:* (1) KEGG_RETINOL_METABOLISM, (2) KEGG_PORPHYRIN_AND_CHLOROPHYLL_METABOLISM. *Amino Acid Metabolism Gene Sets:* (3) KEGG_ALANINE_ASPARTATE_AND_GLUTAMATE_METABOLISM, (4) KEGG_GLYCINE_SERINE_AND_THREONINE_METABOLISM, (5) KEGG_VALINE_LEUCINE_AND_ISOLEUCINE_DEGRADATION, (6) KEGG_ARGININE_AND_PROLINE_METABOLISM, (7) KEGG_HISTIDINE_METABOLISM, (8) KEGG_TRYPTOPHAN_METABOLISM, (9) KEGG_BETA_ALANINE_METABOLISM*,* (10) KEGG_SELENOAMINO_ACID_METABOLISM, (11) KEGG_GLUTATHIONE_METABOLISM. *Energy Metabolism Gene Sets:* (12) KEGG_OXIDATIVE_PHOSPHORYLATION, (13) MITOCHONDRIAL_TRANSPORT, (14) MITOCHONDRION_ORGANIZATION_AND_BIOGENESIS. **b** Carbohydrate, drug, lipid and glycan metabolism gene sets. *Carbohydrate Metabolism Gene Sets:* (1) KEGG_GLYOXYLATE_AND_DICARBOXYLATE_METABOLISM, (2) KEGG_PROPANOATE_METABOLISM, (3) KEGG_BUTANOATE_METABOLISM (4) KEGG_GLYCOLYSIS_GLUCONEOGENESIS, (5) KEGG_CITRATE_CYCLE_TCA_CYCLE, (6) KEGG_PYRUVATE_METABOLISM, (7) KEGG_FRUCTOSE_AND_MANNOSE_METABOLISM, (8) KEGG_GALACTOSE_METABOLISM, (9) KEGG_AMINO_SUGAR_AND_NUCLEOTIDE_SUGAR_METABOLISM. *Drug Metabolism Gene Sets;* (10) KEGG_DRUG_METABOLISM_CYTOCHROME_P450. *Lipid Metabolism Gene Sets:* (11) KEGG_GLYCEROLIPID_METABOLISM, (12) KEGG_GLYCEROPHOSPHOLIPID_METABOLISM, (13) KEGG_ARACHIDONIC_ACID_METABOLISM, (14) KEGG_LINOLEIC_ACID_METABOLISM, (15) KEGG_SPHINGOLIPID_METABOLISM, (16) KEGG_FATTY_ACID_METABOLISM, (17) KEGG_STEROID_HORMONE_BIOSYNTHESIS, (18) PHOSPHOLIPID_BIOSYNTHETIC_PROCESS. *Glycan Metabolism Gene Sets:* (19) KEGG_N_GLYCAN_BIOSYNTHESIS, (20) KEGG_GLYCOSYLPHOSPHATIDYLINOSITOL_GPI_ANCHOR_BIOSYNTHESIS

Transcriptogram values indicating significant differential expression do not directly refer to pathways or terms, but rather to intervals of the ordered list enriched with genes for specific pathways and terms. Therefore, subsequent to transcriptogram analysis (Figs. 3, 4, and 5), we fed the gene lists from each identified interval of differential expression into the publicly available David functional tools [36, 37] to identify additional KEGG pathways (pathways) and GO terms (terms) associated with those intervals. We then had to consider the differential expression of particular pathways and terms independently. For each of these pathways and terms, we calculated average expression levels and determined significance using Welch’s two tail *t* test (Figs. 7, 8, 9, 10, 11, 12, 13, and 14). Finally, we categorized these pathways and terms according to the KEGG pathway map and GO term hierarchies, grouping together functionally overlapping pathways and terms from KEGG and GO.

### Differentially regulated KEGG pathways and GO terms identified from gene set analyses

NC-ADPKD samples had higher-than-normal and C-ADPKD samples lower-than-normal expression of pathways and terms associated with ‘cell proliferation’ (Fig. 7). Both NC-ADPKD and C-ADPKD samples had higher-than-normal expression of ‘cell-death-related’ terms. These expanded results agree with the terms identified using the transcriptogram ordered gene list (Figs. 3, 4, and 5). Consistent with the changes seen in ‘cell growth,’ ‘genetic information processing,’ (Fig. 8) and ‘amino acid metabolism’ (Fig. 9), pathways and terms are higher than normal in NC-ADPKD samples and lower than normal in C-ADPKD samples. The one exception to this pattern, the KEGG ‘ribosome pathway,’ is higher than normal in both NC-ADPKD and C-ADPKD samples. These results suggest that treatments that target cell proliferation are likely to be effective in patients in which cysts have not yet begun to form and ineffective in patients with significant cyst formation. Pathways and terms with abnormal expression in NC-ADPKD samples pique our interest because changes that occur before cysts form have the greatest potential as targets for amelioration of cyst formation.

Conventional gene chip analysis has not identified pathways that explained the cell de-differentiation observed in cystic cells [38, 39]. Our analysis has revealed a group of signal transduction pathways and terms that are abnormal in ADPKD (Fig. 10). Both NC-ADPKD and C-ADPKD samples have higher-than-normal expression of most signal-transduction pathways. Elevated ‘cyclic nucleotide related’ pathways are not surprising, given cyclic AMP’s role in promoting cyst expansion [40, 41]. Both NC-ADPKD and C-ADPKD samples have higher-than-normal expression of Wnt and Notch pathways, which enhance proliferation, increase resistance to apoptosis and inhibit differentiation of epithelial cells [42]. The excess expression is greater in NC-ADPKD than in C-ADPKD samples, again suggesting possible targets for prevention of cyst initiation.

Chronic non-resolving inflammation accompanies cyst accumulation in ADPKD [43]. Our analysis identified multiple ‘immune response’ pathways with abnormal expression (Fig. 11). All but one of the identified ‘immune response’ pathways had higher-than-normal expression in NC-ADPKD samples and further increased expression in C-ADPKD samples, consistent with increased inflammatory response as cysts accumulate. Notably, the morphologically normal NC-ADPKD-derived cells already showed higher-than-normal expression of ‘immune response’ pathways, suggesting that components of the immune response could provide targets to ameliorate the progression of ADPKD. An exception to this pattern was the lower-than-normal expression of the KEGG ‘complement and coagulation’ pathway in C-ADPKD samples.

‘Response-to-stimulus’ gene groups are overexpressed in kidney carcinoma [44]. Our analysis identifies multiple ‘response-to-stimulus’ terms with increased expression in ADPKD (Fig. 12), suggesting mechanistic similarities between PKD and kidney carcinoma [45]. All but one of the identified ‘response-to-stimulus’ terms had higher-than-normal expression in NC-ADPKD samples and further increased expression in C-ADPKD samples. In C-ADPKD samples, expression of ‘response-to-endogenous-stimulus’ genes was lower than normal and also lower than in NC-ADPKD samples.

Establishment and maintenance of epithelial cell polarity are prerequisites for normal function of all epithelial organs. Epithelia depend on cell adhesion molecules to adapt and respond to mechanical forces and cell density, while tight junctions control diffusion across the epithelial layer. Transcriptogram analysis shows that both NC-ADPKD and C-ADPKD cells have higher-than-normal expression of genes in the ‘cell-adhesion-molecule’ and ‘tight-junction’ KEGG pathways (Fig. 12). Nephron segments differentially express multiple tight-junction proteins which may determine segment specific paracellular permeability [46]. ADPKD cyst epithelia express claudin-7 in an abnormal distribution on the apical and basolateral membrane [47]. Decreased presence of strong E-cadherin adhesion molecules on the tubule cell surface, accompanied by upregulation of weaker cell-cell adhesion by N-cadherin and cadherin-8 are also known components of ADPKD [48, 49]. This lower-than-normal cell-cell adhesion accompanies higher-than-normal expression of cell motility terms, both hallmarks of epithelial to mesenchymal transition and cellular de-differentiation.

Defects in urine concentration are a well-established component of ADPKD [50]. NC-ADPKD samples had slightly higher-than-normal and C-ADPKD samples had significantly lower-than-normal expression of ‘vasopressin-regulated water reabsorption’ [51] and ‘proximal tubule bicarbonate reclamation’ [52] pathways (Fig. 13).

ADPKD causes widespread disruptions of metabolism (Fig. 14). Of the metabolic pathways and terms identified, ‘cofactor and vitamin metabolism’ and ’amino acid and energy metabolism’ were all decreased in C-ADPKD samples. ‘Carbohydrate, drug, lipid and glycan metabolism’ pathways were lower than normal in C-ADPKD samples with the exception of ‘fructose and mannose,’ ‘galactose,’ and ‘amino sugar and nucleotide sugar metabolism’. ‘Drug metabolism cytochrome P450’ pathway expression levels were lower than normal in both NC-ADPKD and C-ADPKD samples. ‘Lipid metabolism’ pathway expression was significantly abnormal, but with mixed expression effects. ‘Glycan metabolism’ pathway expression was slightly higher than normal in NC-ADPKD samples and lower than normal in C-ADPKD samples.

The differential expression of pathways and terms (Figs. 7, 8, 9, 10, 11, 12, 13, and 14) in the gene sets associated with significantly altered transcriptogram intervals (Figs. 4, 5, and 6) provides strong evidence that:

1. ‘Proliferation,’ ‘genetic information processing,’ and ‘nucleotide metabolism’ pathways and terms are higher-than-normal in NC-ADPKD and lower-than-normal in C-ADPKD, while ‘apoptosis’ is higher-than-normal in both NC-ADPKD and C-ADPKD.
2. ‘Immune response’ pathways and terms overall are upregulated in both NC-ADPKD and C-ADPKD.
3. Changes in ‘signal transduction’ and ‘cellular process’ pathways are consistent with de-differentiation of epithelial cells in ADPKD.
4. ‘Metabolism’ pathways and terms overall are downregulated in ADPKD.

We therefore posit that genes from the pathways and terms that are uniquely altered in NC-ADPKD samples (e.g., before cysts have formed) have the greatest potential as drug targets for ameliorating cyst formation.

### Finding individual candidate genes

**Fig. 15:**
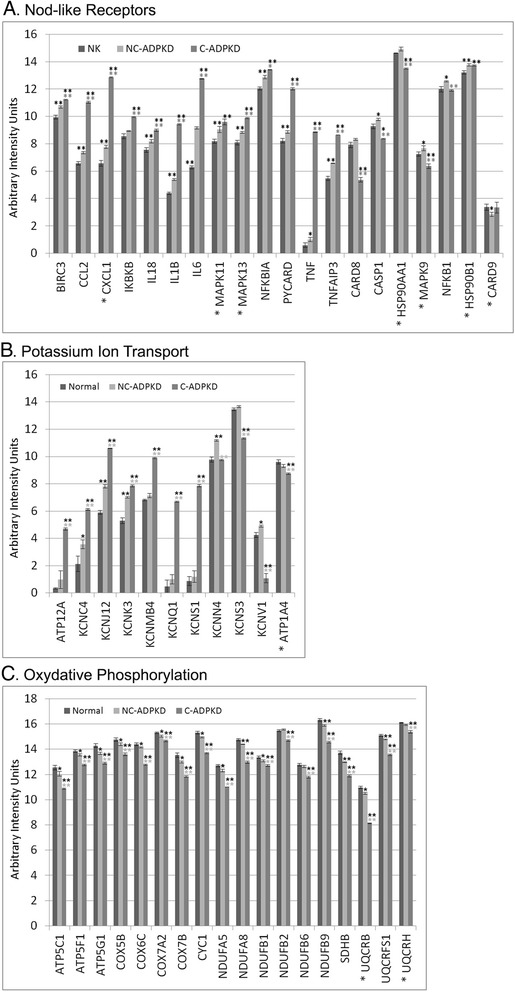
Comparisons of individual gene expression levels for genes selected from differentially expressed gene sets. **a** NOD-like receptor signaling, **b** potassium ion transport, and **c** oxidative phosphorylation. To simplify presentation of these gene sets, where we used two or more probes in the microarray for a single gene, we give mean values and mark the gene names with an “asterisk”. Within gene sets, we can discern changes in individual genes responsible for differential expression of the pathways. We present data as mean ± s.e.m., **P < 0.01, *P < 0.05 (*black asterisk* is in comparison to NK, *gray asterisk* is in comparison to NC-ADPKD). Additional file 4 provides additional information on each gene

We next examine the expression levels of the individual genes (Fig. 15) in selected pathways and terms which transcriptogram analysis identifies as differentially expressed at a significant level (Figs. 7, 8, 9, 10, 11, 12, 13 and 14). Selection of pathways and terms for further examination was based on significance level and relevance to the existing literature. These genes likely play key roles in development of ADPKD and are thus potential targets for drug therapies to prevent or delay ADPKD. Figure 15 shows the levels of expression of individual genes from three pathways and terms, ‘Nod-like receptor signaling’ a term from ‘immune response,’ ‘potassium ion transport’ a term from ‘ion homeostasis,’ and ‘oxidative phosphorylation’ a term from ‘energy metabolism’. We then confirmed expression levels of individual genes in these pathways and terms using qRT-PCR (Additional file 3).

The intracellular NOD-like receptor (NLR) family plays a central role in regulation of the innate immune response. The NLRs NOD1 and NOD2 sense bacterial peptidoglycan fragments and respond by stimulating the activation of NF-κB (nuclear factor kappa beta) and MAPK, cytokine production, and apoptosis. The NLRs NALP1 (NLR-pyrin containing), NALP3 and Ipaf activate caspase-1 through inflammasomes, which generate pro-inflammatory cytokines. NOD-like receptor signaling contributes to multiple renal diseases with inflammatory components, including ureteric obstruction, ischemia reperfusion injury, glomerulonephritis, sepsis, hypoxia, glycerol-induced renal failure, and crystal nephropathy [53–55]. However, neither the Volcano plots nor GSEA analysis found abnormal activity of NOD-like receptor signaling in ADPKD cells. We examined expression of the 20 individual genes involved in the KEGG ‘NOD-like receptor signaling’ pathway (Fig. 15a) that were present in our microarrays. In NC-ADPKD cells, all but one of the 20 NOD-like receptor signaling genes had higher-than-normal expression (Fig. 15a), suggesting that that reducing NOD-like receptor signaling early in ADPKD might ameliorate the progression of the disease. C-ADPKD cells also had higher-than-normal expression levels of several of these genes, including tumor necrosis factor (TNF). TNF blockers are an approved treatment for debilitating chronic inflammatory diseases, in particular rheumatoid arthritis (RA) so tumor necrosis factor (TNF) blockers specifically and NOD-like receptor signaling blockers in general may be worth testing for treatment of ADPKD. Even if they do not slow cyst progression, they may reduce the chronic pain associated with inflammatory diseases like ADPKD.

The kidney is the primary regulator of total body potassium content. Maintenance of proper levels and distribution of potassium across the cell membrane is critical for normal cell function [56]. Further, potassium channels are critical for cAMP-dependent chloride secretion and cyst growth in ADPKD [57]. We examined expression of the 11 individual genes in our microarrays with the GO ‘potassium ion transport’ label (Fig. 15c). Our analysis revealed changes of expression of ‘potassium ion transport’ as a category and of individual genes within that category, which neither Volcano plotting or GSEA analysis was able to identify.

Pkd1^−/−^ cells and kidneys from cystic mice preferentially rely on aerobic glycolysis for their energy demands, a metabolic switch similar to the Warburg effect in cancer cells [58]. Our analysis revealed changes in pathways and terms related to ‘energy metabolism’ that conventional volcano and GSEA analysis missed. We examined expression of the 20 individual genes in the KEGG ‘oxidative phosphorylation’ pathway (Fig. 15b) that were present in our microarrays. Expression of all oxidative phosphorylation related genes was significantly lower than normal in both C-ADPKD and NC-ADPKD samples, with greater reduction in C-ADPKD samples. Volcano plotting also identified phosphoenolpyruvate carboxy-kinase 1 (Pck1) as significantly differentially expressed (Fig. 1); the Pck1 enzyme is a main control point for the regulation of gluconeogenesis. Pck1 expression in NC-ADPKD samples is 5.58 times higher than normal (*p* = 1.54 × 10^− 8^) and is 0.58 times lower than normal (not significant) in C-ADPKD samples.

## Discussion

In this communication, we compare gene expression profiles between a paired age and sex-matched pair of telomerase immortalized human cell lines (NK and PKD). A second grouped pair of immortalized human cell lines (NC-ADPKD and C-ADPKD) was derived from renal cysts and normal appearing nephrons from the same human male polycystic kidney. The choice to use immortalized cell lines as the source material to isolate RNA was based on our prior unsuccessful attempts to obtain good-quality RNA from polycystic kidneys. Due to their large size and the need to cross clamp the kidneys at the renal pedicle, we obtained poor-quality RNA isolated from the nephrectomy specimen likely because of prolonged warm ischemia time. While the use of cell lines allowed us to eliminate the issue of RNA degradation, it also brings forward the question as to how generalizable is the data presented in this communication. In our review, the group that solved the problem of obtaining high-quality RNA from polycystic kidney surgical specimens was Song et al. [17]. Our preliminary analysis of that data set using the transcriptogram approach shows a close concordance between our data and Song’s data. This comparison will be the subject of a future communication. Other evaluations of gene expression data have relied on murine cell lines or RNA isolated from newly formed cysts from conditional knockout models, and results from these studies identify similar themes for aberrant signaling such as immune defense, cell structure and motility, cellular proliferation, apoptosis, and metabolic control [59–62]. These are GSEA findings that are also observed in our study suggesting that a subset of findings observed in our cell lines are generalizable.

Current analyses of genome-wide gene expression measurements (microarrays or RNASeq, for example) such as single gene Volcano plots (Fig. 1) and GSEA gene set analysis (Tables 1 and 2) have failed to identify patterns of gene expression that define specific diseases or reveal the entirety of their molecular mechanisms of pathogenesis. Our ADPKD transcriptogram analysis (Figs. 4, 5, and 6) identified more significantly altered KEGG pathways and GO terms than either Volcano plots (Fig. 2) or GSEA analysis (Tables 1 and 2). Importantly, transcriptogram analysis identifies additional pathways and terms which are consistent with what we know about signaling, functional, and morphological changes that occur in ADPKD, clarifying the mechanisms by which PKD mutations drive cyst formation.

Comparing global gene expression profiles between NK, ADPKD, NC-ADPKD, and C-ADPKD shows that the NC-ADPKD gene profile is most similar to the NK line expression profile. The NK cell line profile is markedly different from both the C-ADPKD and end-stage ADPKD cell line gene expression profiles (Additional file 5: Figure S5). NC-ADPKD, C-ADPKD, and ADPKD cell lines all have truncation mutations in one allele of the PC-1 gene. However, when we sequenced the PC-1 genomic clones, we found no evidence for second somatic mutations in the PC-1 gene [25]. Additionally, there were no differences in total polycystin-1 and 2 protein expression in the ADPKD cell line [25]. The sole distinguishing feature between the NK and ADPKD cell lines was the absence of polycystin-1 in the cilia of the ADPKD cells [63]. Our transcriptogram analysis suggests that some other genetic event, i.e., a second hit, causes the dramatic shift in gene expression which transforms non-cystic ADPDK cells into cystic ADPKD cells [64]. The transition in expression profiles may have been determined during the early steps of cyst emergence that set the subsequent gene expression profile to reflect a fundamentally altered differentiation profile [17] [65]. Alternatively, a second somatic mutation that launched cyst emergence occurred in some other cystogenic gene site where we have incomplete information at present [66, 67]. Notably, in a murine conditional PKD1 knockout, cyst formation is most robust with PKD1 inactivation up to post-natal day 14 [68]. In other knock out models, cyst formation also requires a kidney injury and repair cycle to yield robust cystogenesis suggesting that there is a developmental window, recapitulated during cell repair that leads to a risk of cyst formation when polycystin-1 levels are abnormal [69].

Several of the many signaling pathways altered in cystic epithelia deserve special consideration [70]. Several potassium channel genes are overexpressed in our cystic cell lines. These potassium channel genes are KCNC4 (potassium voltage-gated channel subfamily C member 4), KCNJ12 (potassium voltage-gated channel subfamily J member 12), KCNK3 (potassium two pore domain channel subfamily K member 3), KCNMB4 (potassium calcium-activated channel subfamily M regulatory beta subunit 4), KCNQ1 (potassium voltage-gated family Q member 1), and KCNS1 (potassium voltage-gated channel modifier subfamily S member 1). NC-ADPKD cells have higher-than-normal and C-ADPKD cells have lower-than-normal expression of KCNN4 (potassium calcium-activated channel subfamily N member 4), KCNV1 (potassium voltage gated channel modifier subfamily V member 1), and KCNS3 (potassium voltage-gated channel subfamily A member 3). Notably, KCNN4 is responsible for calcium-dependent cyst fluid secretion and is thus a potential therapeutic target to inhibit cyst growth [57]. Many of these channels act to hyperpolarize the cell membrane, and voltage-sensitive K channel membrane depolarization induces nano-scale assembly of phosphatidyl serine, which in turn results in nano-scale clustering sites for K-ras, thereby promoting cell cycle progression via MAPK downstream signaling [71, 72]. Experimental studies have demonstrated that cyst expansion results in cell stretching [73]. Since some K channels are stretch activated, K channel activation of proliferation could lead to feedback, where cyst expansion driven by fluid secretion directly accelerates cell-cycle progression [73–76].

Our transcriptogram analysis found significantly higher-than-normal expression of downstream mediators of the ‘inflammasome signaling’ pathway in NC-ADPKD and C-ADPKD samples, which may accelerate disease pathogenesis [53, 77–80]. This signaling cascade includes nucleotide-binding oligomerization domain receptors or NOD-like receptors, which have been implicated in renal diseases including ureteric obstruction, ischemia reperfusion injury, glomerulonephritis, sepsis, hypoxia, glycerol-induced renal failure, and crystal nephropathy [53–55, 81]. Our data show that NOD-like receptor signaling may also play an important role in ADPKD. NC-ADPKD and C-ADPKD samples had higher-than-normal (*p* < 0.01) expression of a number of other inflammasome signaling genes, including: interleukin (IL) 6, IL1β, NFκB1A, CCL2 (chemokine (C-C motif) ligand), BIRC3, TNFAIP3, IL18, PYCARD (also called ASC), and MAPK13.

Inflammasome signaling, a part of the innate immune system, includes membrane receptors that recognize conserved pathogen-associated molecular patterns (PAMPs) on invading pathogens or host-derived danger-associated molecular patterns (DAMPs) released in response to stress, reactive oxygen species generation, or cell death [78]. Both Toll-like receptors and C-type lectin pattern recognition receptors (PRRs) can initiate inflammasome signaling at the cell membrane [55]. Additional PRRs include the retinoic acid-inducible gene (RIG-1)-like receptors which recognize double-stranded RNAs (dsRNA) and NOD-like receptors which recognize microbe-associated molecular pattern signatures (MAMPS) and DAMPs. Other intracellular monitors of the system include HIN-200 receptors and RIG-1-like receptors [82–84]. Inflammasome signaling begins with a priming phase, initiated by receptors that signal through myeloid differentiation primary response (MyD88) pathways and other NFκB activating pathways such as IL-1R and the tumor necrosis factor receptor (TNFR) [55, 85]. Priming promotes IL1β, NRLP3, and IL18 transcription and protein synthesis and requires interleukin 1 receptor-associated kinase (IRAK1) activation [86]. K-^+^efflux, adenosine tri-phosphate (ATP), reactive oxygen species (ROS), pore-forming toxins, or crystals [87–91] all lead to inflammasome activation. The purinergic receptor P2X7 [78, 89, 92, 93] is another inflammasome activator, which is more active under conditions of low fluid shear stress. ADPKD cells, lining cyst walls, are sheltered from fluid shear normally found in tubule lumens; hence, it is likely that in vivo P2X7 is more active [29]. Following inflammasome activation, NOD-like receptors such as NLRP3 form macromolecular complexes with ASC (apoptosis-associated speck-like protein, PYCARD), an adaptor with a caspase recruitment domain (CARD) that bridges NLRs and caspase-1 [94]. This macromolecular complex activates caspace-1, which enzymatically cleaves the pro-inflammatory cytokines IL-1β and IL-18 [95]. Inflammasome signaling has transcriptional effects mediated via MAPK to induce NFκB and the pro-inflammatory cytokines IL-1β and IL-18 [86].

The cystic epithelial cells in our study have higher-than-normal expression of the inflammasome adaptor PYCARD/ASC and the signal modulators BIRC3, preparing them to over-respond to inflammasome signals [80, 95]. The cystic epithelial cells also have higher-than-normal levels of the mRNAs encoding downstream effectors of inflammasome signaling, which likely increase expression of IL1β, IL-18, TNFα, monocyte chemotactic protein-1 (MCP-1), and IL-6 [96–98]. While many of these protein biomarkers correlate with disease severity in either animal models of renal cyst disease or urine from patients with ADPKD [99, 100], our analysis indicates that they may also drive cyst genesis and expansion.

Intense fibrosis and loss of functional parenchyma accompany kidney enlargement during ADPKD disease progression [101, 102]. Our transcriptome analysis suggest that cystic epithelial cells can excessively augment innate inflammatory responses that may accelerate fibrosis and stimulate a chronic inflammatory state by driving macrophage polarization and activation [103]. Since intracellular levels of calcium are low in ADPKD cells [70, 104, 105] and levels of P2X7 gene expression are lower than normal [25], reactive oxygen species are the most likely trigger of inflammasome assembly [88]. Even though P2X7 message levels are lower than normal in ADPKD cells, primary cultured cyst epithelial cells secrete high levels of ATP [29, 106], leading to maximal stimulation of any P2X7 receptors that are present. Since P2X7 activation co-stimulates formation of the NALP inflammasome, cystic epithelial cells may be the primary sources of the IL18 and IL1β identified in cyst fluid [100, 107].

Recent evidence suggests that other K channels can activate transport vesicle release in response to inflammasome activation, and the resultant vesicle cargo protein composition depends on the K channel responsible for membrane depolarization [92]. Thus, aberrant endogenous K-channel expression in cystic epithelia may impair auto regulatory responses to interleukin secretion.

We propose a scenario for the persistent inflammation seen in ADPKD kidneys based on our transcriptogram analysis. Our findings show higher-than-normal mRNA expression of MCP-1 in C-ADPKD cells, which would promote mononuclear cell infiltration into cystic kidney parenchyma. Within the parenchyma, IL1β, IL-18, and IL-6 can all activate infiltrated mononuclear cells. High levels of DAMPs released by apoptotic cells, and DAMPs such as hyaluronic acid and protein fragments resulting from proteolytic extracellular matrix fragments in ADPKD cystic kidneys would then further push mononuclear cells towards an M2 phenotype [99, 107–111]. These M2-like monocytes would continuously secrete transforming growth factor (TGF-β) and connective tissue growth factor (CTGF), which would convert cells in the interstitium to myofibroblasts [78, 102]. Other M2-like monocytes would stimulate epithelial cell proliferation thereby driving cyst expansion [103]. The same signals would stimulate Th1 cells to secrete IFN-γ, IL-13, and Th2 cells to secrete IL-4 and IL-5 and Treg cells to secrete IL-10 and TGF-β, exacerbating the dysregulated inflammatory cascade [86]. This pathogenic picture suggests that pharmacologic interventions which attenuate the inflammatory cascade should delay cyst expansion and fibrosis in ADPKD.

## Conclusions

Our analysis of ADPKD provides a workflow which applies to the hierarchical analysis of any large-scale microarray study. We begin with transcriptogram analysis as a method of global triage to identify intervals of altered expression in the ordered gene list, then determine which genes in those intervals of KEGG pathways or GO terms that are differentially expressed (e.g., ‘potassium ion transport’ in Fig. 14), and finally explore the significance of altered expression profiles of single genes within those altered pathways and terms (Fig. 15). The transcriptograms provide crucial mechanistic context for understanding the significance of altered gene expression.

## Methods

### Cell lines and culture conditions

All cell lines derived from human kidneys under a protocol were approved by the Indiana University Institutional Review Board. We immortalized all cell lines with human telomerase as previously described and maintained them in continuous culture [25]. We grew cells with renal epithelial growth media supplemented with 5% fetal bovine serum (Lonza, Bazel, Switzerland and GE Healthcare HyClone, Little Chalfont, Buckinghamshire, UK) and maintained them at 37 °C, 5% CO_2_ atmosphere. We passaged cells twice-weekly using trypsin-EDTA (Sigma-Aldrich, St. Louis, MO). We micro-dissected the source tissue for the NC-ADPKD and C-ADPKD cell lines from an early stage polycystic kidney from a 36-year-old male. We isolated NC-ADPKD cells from intact renal tubules and C-ADPKD cells from surface cysts. All cell lines expressed the proximal tubule markers ANPEP (alanyl aminopeptidase, membrane), FAH (fumarylacetoacetate hydrolase), SLC5A2 (sodium glucose transporter 5A2), LRP2 (LDL receptor related protein), and HNF1A (hepatocyte nuclear factor 1 homeobox A). They did not express proximal marker CUBN (cubulin). All lines expressed the cortical thick ascending limb marker UMOD (uromodulin). No lines expressed SLC12A1 (solute carrier family 12 member 1) or KCNJ1 (potassium voltage-gated channel subfamily J) . All cell lines expressed the distal convoluted tubule markers KLK1 (kallikrein-1) and KL (klotho). Only the NC-ADPKD cells expressed SLC12A3 (solute carrier family 12 member 3). The NK cell line expressed PVALB (parvalbumin). All cell lines expressed the collecting duct markers, AQP2 (aquaporin 2), and SCNN1G (sodium channel epithelial 1 subunit gamma), while no cell lines expressed FXYD2 (sodium/potassium-transporting ATPase gamma). The gene-expression profiles suggest that the cultured cells likely originate from proximal tubule segments. In contrast, the NK kidney cell line is a mixed cell population made from whole kidney collagenase digest. Herbert et al. have described the NK and ADPKD Q4004X cells [25]. We sequenced the HmPKD1 gene using a protocol of Herbert et al., revealing a heterozygous truncation mutation at amino acid 2556 (Q2556X) [25].

### Chemicals and reagents

We purchased all buffers and inorganic chemicals from Sigma-Aldrich (Sigma-Aldrich, St. Louis, MO). We isolated total RNA from cells using TRIzol Reagent (Thermo Fischer Scientific, Waltham, MA) according to manufacturer’s directions. We plated cells onto Costar membrane filter supports at a density of 50,000 cells/cm^2^ and grew them for 5 days to ensure confluence [25].

We labeled 200 ng of purified total RNA using the Agilent QuickAmp labeling kit, 1-color (Agilent Technologies, Palo Alto, CA) and hybridized purified cRNA to a custom Agilent Human Whole Genome 4x180K array, which replicates the content of the v2 4x44k array at least four times (AMIDID 026822). After hybridization at 65 **°**C, 20 rpm for 17 h, we washed arrays in wash buffer 1 (6XSSC (saline sodium citrate), 0.005 % TritonX-102) for 5 min at room temperature and wash buffer 2 (0.06X SSC, 0.005 % Triton X-102) for 5 min at 37 °C. We scanned arrays on the Agilent High-Resolution Microarray B-series Scanner (G2505B) and quantified the images using Agilent Feature Extraction software (version 10.7).

### Microarray data preprocessing and normalization

We processed extracted feature (reporter) intensities from each array separately by subtracting a constant to bring the minimum intensity to 1 and then taking the log, base 2. We mapped the transformed intensities using a non-linear function to ensure that the cumulative distribution profiles of the intensities were comparable between all arrays. We present the resulting comparable feature intensities in arbitrary units. We removed features with low intensity from the analysis if the 90th percentile was below the limit of detection (LoD), which we estimated as the mean of a set of negative controls plus 1.96 times the standard deviation of the negative controls. We also removed features with low range across all samples, which we defined as having a difference between the 95th percentile and 5th percentile of less than log 2 (1.5-fold). We summarized the replicate probes by averaging and constructed a mixed-effects linear model of expression using factors for tissue type (cysts or kidney), status (ADPKD or NK), and cell line (NK, C-ADPKD, NC-ADPKD, ADPKD), treating cell line as a random effect.

### Volcano plotting

We averaged gene expression levels over the replicates for each cell line after normalization and compared the cell lines by calculating the FC. We defined FC as the ratio between the averages and we determined *P* values for each gene expressed. Volcano plots use FC as the *x*-axis and the *P* value as the *y*-axis (Fig. 2). We defined significantly changed genes as those with thresholds of FC > 2 and *P* < 0.01.

### Gene Set Enrichment Analysis (GSEA)

We performed gene set enrichment analysis using the application available at the Broad Institute Gene Set Enrichment Analysis website [26, 33]. We estimated the signal-to-noise ratio and false discovery rates (FDR), from 1000 gene-set permutations, due to the low number of samples in each class (*n* = 3). We tested the Gene Ontology: Biological Process terms and KEGG pathways gene sets. Since we used gene permutations, we considered gene sets to be differentially expressed if FDR < 0.05. We calculated GSEA for NC-ADPKD versus NK, C-ADPKD versus NK, and C-ADPKD versus C-ADPKD.

### Transcriptogram analysis

#### Ordering the gene list

Transcriptograms give an integrated visualization of genome-wide gene expression data by projecting expression levels onto a functionally ordered gene list to produce a genome-wide expression profile [23, 27, 34]. The gene list ordering is based on STRING database protein-protein interactions [34] with confidence scores over 0.800 drawn from all methods to infer associations, with the exception of text mining. The ordering process is performed using a Monte Carlo algorithm that minimizes a cost function:

1 $$ F={\displaystyle \sum_{i=1}^{N-1\ }}\ {\displaystyle \sum_{j=i+1}^N}\ {d}_{i\ j}^{\alpha}\left(\ \left|{A}_{i\ j}-{A}_{i+1\ j}\right|+\left|{A}_{i\ j}-{A}_{i-1\ j}\right|+\left|{A}_{i\ j}-{A}_{i\ j+1}\right|+\left|{A}_{i\ j}-{A}_{i\ j-1}\right|\right), $$

where *N* is the number of genes in the list and *A* is the association matrix. *A*_*i j*_ = 1 when the products of the genes at positions *i* and *j* in the list associate and *A*_*i j*_ = 0 otherwise. *d*_*i j*_ = |*i* − *j*| measures the distance between the locations of genes *i* and *j* in the list. Minimizing *F* causes associated genes to cluster in the list. Minimization starts with a randomly ordered list of all genes whose products have at least one association. Each step chooses a pair of genes randomly, tentatively swaps their positions, and calculates the change in the cost function Δ*F*. The algorithm accepts the position swap whenever Δ*F* ≤ 0. If Δ*F* > 0, the algorithm accepts the swap with probability $\exp -\frac{\Delta F}{T}.$ *T* is a fluctuation amplitude that allows the configuration to escape from local minima of Δ*F*. One Monte-Carlo step (MCS) equals *N* attempted gene-pair exchanges. The simulated annealing algorithm starts with *T* large and decreases *T* by a small amount once every 100 MCS. The ordering algorithm produces a gene list (Additional file 2) where the probability that any two genes associate decays exponentially with their distance in the list.

#### Visualizing KEGG pathway and GO term profiles in the ordered list

Because STRING database interactions use KEGG pathway maps for benchmarking and calibration [112], genes in close proximity in the list are probably in the same KEGG pathway. Consequently, the ordered list groups genes by biological function. We used David’s Annotational Tools [113] to obtain a list of the GO terms and KEGG pathways that associate to intervals of the ordered list. For each of these GO terms and KEGG pathways, we obtain a profile by creating an *N*-element vector, $\overrightarrow{h}$, and setting *h*_*i*_ = 1, if the gene at position *i* in the list participates in the specified GO term or KEGG pathway and *h*_*i*_ = 0, if the gene at position *i* in the list does not participate in the specified GO term or KEGG pathway [113, 114]. For each pathway or term, we obtained a profile for *r* = 30. A value of 1 indicates that all genes in an interval participate in a given GO term or KEGG pathway. We read the resulting profiles along the ordered list as indicative of the level of enrichment with genes of a given GO term or KEGG pathway. Interpretation of transcriptograms is then facilitated by comparing the enrichment profiles from regions with significant difference in their transcriptograms.

#### Transcriptogram calculation

We next map the microarray data onto the ordered list. We then smooth the raw profile to obtain a final *transcriptogram profile*, a new *N* − 2*r* − 1 element vector, $\overrightarrow{k}$ using a running average with a window of size 2*r* + 1 (*r* = 30):

2 $$ {k}_i=\frac{1}{2r+1}{\displaystyle \sum_{j=-r}^r}{h}_{i+j}, $$

where *i* ∈ {*r* + 1, *N* − *r* − 1}*.* The resulting profile is the transcriptogram for the expression data. Profile peaks in $\overrightarrow{k}$ can be interpreted by comparison with the KEGG pathways and GO terms enriched in that region of the ordered list.

#### Class comparison

We next compare transcriptograms between sample classes. First, we produce the transcriptograms. We next average the transcriptograms for all the samples of each class, producing a class-average transcriptogram profile, $\left\langle \overrightarrow{k}\right\rangle$ and corresponding standard error profile ${\overrightarrow{\sigma}}_k$. To compare a reference (*R*) and perturbed (*P*) expression pattern, we divide the perturbed by the normal value for each averaged transcriptogram profile element to obtain the relative transcriptogram ${\overrightarrow{\varDelta}}_{P/R}$:

3 $$ {\varDelta}_i=\frac{k_i^P}{k_i^R}. $$

Regions in ${\overrightarrow{\varDelta}}_{P,R}$ with *Δ*_*i*_ ≫ 1  or ≪ 1 indicate specific perturbed gene expression. The upper panels in Figs. 4, 5, and 6 show the relative transcriptograms*.*

### Significance evaluation

We estimate the significance of differences between two transcriptogram classes using a two-tailed Welch’s *t* test on the class averages to obtain a *P* value for each list position and by creating 500 random permutations of the sample labels (phenotypes) to estimate the FDR. The middle panels in Figs. 4, 5, and 6 show the *P* value for each list position.

## Additional files

Additional file 1: Lists of genes which Volcano plotting identifies as abnormally expressed. (PDF 231 kb)
Additional file 2: Ordered gene list. (PDF 1000 kb)
Additional file 3: Microarray validation by qRT-PCR. (PDF 235 kb)
Additional file 4: Protein expression information on NOD-like receptor signaling, potassium ion transport, and oxidative phosphorylation gene sets from the human protein atlas. (PDF 94.6 kb)
Additional file 5: Relative transcriptogram for ADPKD cells. (PDF 330 kb)

## Abbreviations

ADPKD: Autosomal dominant polycystic kidney disease
ANPEP: Alanyl aminopeptidase, membrane
ASC: Apoptosis-associated speck-like protein
ATP: Adenosine tri-phosphate
C-ADPKD: Cystic autosomal dominant polycystic kidney disease
CARD: Caspase recruitment domain
CCL2: Chemokine (C-C motif) ligand 2
CTGF: Connective tissue growth factor
CUBN: Cubulin
DAMPs: Damage- associated molecular patterns
DNA: Deoxyribonucleic acid
ECM: Extracellular matrix
Erk: Extracellular signal regulated kinase
FAH: Fumarylacetoacetate hydrolase
FC: Fold change
FDA: Federal Drug Administration
FDR: False discovery rate
FXYD: Sodium/potassium-transporting ATPase gamma
G PROT: G protein
GO: Gene ontology
GSEA: Gene Set Enrichment Analysis
HmPKD1: Human polycystic kidney disease gene 1
HmPKD2: Human polycystic kidney disease gene 2
HNF1A: Hepatocyte nuclear factor 1 homeobox A
IL: Interleukin
IRAK1: Interleukin 1 receptor associated kinase 1
Jnk: cJun N- Terminal kinase
KCNC4: Potassium voltage-gated channel subfamily C member 4
KCNJ1: Potassium voltage-gated channel subfamily J
KCNJ12: Potassium voltage-gated channel subfamily J member 12
KCNK3: Potassium two pore domain channel subfamily k member 3
KCNMB4: Potassium calcium-activated channel subfamily M regulatory beta subunit 4
KCNN4: Potassium calcium-activated channel subfamily N member 4
KCNQ1: Potassium voltage gated family Q member 1
KCNS1: Potassium voltage gated channel modifier subfamily S member 1
KCNS3: Potassium voltage gated channel subfamily A member 3
KCNV1: Potassium voltage gated channel modifier subfamily V member 1
KEGG: Kyoto Encyclopedia of Genes and Genomes
KL: Klotho
KLK1: Kallikrein-1
LRP: LDL receptor-related protein
MAPK: Mitogen-activated protein kinase
MCP: Monocyte chemotactic protein
mTOR: Mammalian target of rapamycin
MyD88: Myeloid differentiation primary response 88
NALP1: NLR pyrin containing
NC ADPKD: Non-cystic autosomal dominant polycystic kidney disease
NF-kB: Nuclear factor kappa beta
NK: Normal kidney
NLR: NOD-like receptor
NLRP3: NOD-like receptor protein
NOD: Nucleotide-binding oligomerization domain
P2X: Purinergic receptor
PAMPs: Pathogen associated molecular patterns
PC-1: Polycystin 1
PC-2: Polycystin 2
Pck1: Phosphoenolpyruvate carboxy-kinase 1
PKD: Polycystic kidney disease
PRRs: Pattern recognition receptors
PVALB: Parvalbumin
qRT-PCR: Quantitative reverse transcriptase polymerase chain reaction
RA: Rheumatoid arthritis
RIG-1: Retinoic acid inducible protein
RNA: Ribonucleic acid
ROS: Reactive oxygen species
SCNN1G: Sodium channel epithelial 1 subunit gamma
SLC12A1: Solute carrier family 12 member 1
SLC12A3: Solute carrier family 12 member 3
SLC5A2: Sodium glucose transporter 5A2
Stat: Signal transducer and activator of transcription 1
TCA: Tricarboxylic acid
TGF: Transforming growth factor
TNF: Tumor necrosis factor
Treg: Regulatory T cell
UMOD: Uromodulin
Wgless: Wingless
Wnt: Wingless-related integration site
