# Supplementary material for: Transcriptome analysis reveals manifold mechanisms of cyst development in ADPKD
Source: Hum Genomics. 2016 Nov 21;10:37. doi: 10.1186/s40246-016-0095-x (PMC5117508; doi:10.1186/s40246-016-0095-x)
Supplement: Additional file 1: — Lists of genes which Volcano plotting identifies as abnormally expressed. (PDF 231 kb) [file 40246_2016_95_MOESM1_ESM.pdf]

| Significantly Changed Genes NC-ADPKD vs. NK | | | | | |
|---|---|---|---|---|---|
| Protein | Gene symbol | Position | Relative position | Fold change | P value |
| ensp00000319814 | pck1 | 3713 | 0.383415944 | 5.583712739 | 1.54E-08 |
| ensp00000314151 | klk3 | 5384 | 0.555968608 | -12.17241192 | 3.70E-05 |
| ensp00000231751 | ltf | 5437 | 0.561441553 | 4.50460022 | 8.44E-05 |
| ensp00000358525 | ngf | 4433 | 0.457765386 | 2.005692753 | 9.80E-05 |
| ensp00000296871 | csf2 | 4562 | 0.471086328 | 3.821352941 | 0.000214939 |
| ensp00000317145 | gdnf | 4764 | 0.491945477 | 2.647404444 | 0.000302276 |
| ensp00000352064 | klrc1 | 5982 | 0.61771995 | 5.273866972 | 0.000325934 |
| ensp00000296140 | ccr1 | 4050 | 0.418215613 | -2.889418535 | 0.000710883 |
| ensp00000378359 | adh6 | 7781 | 0.803490293 | -5.081081081 | 0.000790539 |
| ensp00000318867 | cyp8b1 | 7824 | 0.807930607 | -2.797832169 | 0.00127787 |
| ensp00000236147 | sell | 6977 | 0.720466749 | 3.044065439 | 0.00157787 |
| ensp00000268933 | epn3 | 4975 | 0.513733994 | -3.220280846 | 0.00166315 |
| ensp00000355378 | plxnb3 | 6236 | 0.643948781 | 2.084190722 | 0.00173232 |
| ensp00000263413 | c6 | 2525 | 0.260739364 | 7.363227517 | 0.00177939 |
| ensp00000005180 | ccl26 | 4080 | 0.421313507 | 2.413398183 | 0.0018296 |
| ensp00000265440 | tfec | 2702 | 0.279016935 | -2.004648614 | 0.00305922 |
| ensp00000242338 | cntfr | 4628 | 0.477901694 | -3.379596658 | 0.0034224 |
| ensp00000329210 | or2t10 | 9592 | 0.990499793 | -3.053191489 | 0.0038099 |
| ensp00000265643 | gal | 4036 | 0.41676993 | 2.804490385 | 0.00453194 |
| ensp00000305355 | prkcb | 5216 | 0.538620405 | -3.297926156 | 0.00478743 |
| ensp00000269346 | ttyh2 | 390 | 0.040272615 | -3.623704318 | 0.00516668 |
| ensp00000351671 | ccl20 | 4060 | 0.419248245 | 3.235491082 | 0.00536753 |
| ensp00000244709 | trem1 | 8754 | 0.903965304 | 7.405157409 | 0.00566153 |
| ensp00000287641 | sst | 3946 | 0.407476249 | 3.423913043 | 0.00606264 |
| ensp00000288490 | dgki | 7200 | 0.743494424 | 3.658469718 | 0.00684863 |
| ensp00000222482 | cpa4 | 24 | 0.002478315 | 2.332882246 | 0.00742684 |
| ensp00000312326 | aoc3 | 7885 | 0.814229657 | 3.410155335 | 0.00805939 |
| ensp00000339398 | hla-dqa1 | 5722 | 0.590871541 | 2.276194685 | 0.00885913 |
| ensp00000414360 | hla-dqa1 | 6523 | 0.673585295 | 2.276194685 | 0.00885913 |
| ensp00000387892 | hla-dqa1 | 8745 | 0.903035936 | 2.276194685 | 0.00885913 |
| ensp00000409127 | hla-dqa1 | 8746 | 0.903139199 | 2.276194685 | 0.00885913 |
| ensp00000372738 | hla-dqa1 | 8885 | 0.917492772 | 2.276194685 | 0.00885913 |
| ensp00000287713 | nmnat2 | 2775 | 0.286555143 | 3.46 | 0.00905935 |
| ensp00000364430 | sp5 | 3459 | 0.357187113 | 2.751449277 | 0.00995578 |

| Significantly Changed GenesC-ADPKD vs. NK | | | | | |
|---|---|---|---|---|---|
| Protein | Gene symbol | Position | Relative position | Fold change | P value |
| ensp00000303153 | col22a1 | 5701 | 0.588703015 | 14.96810103 | 1.49E-10 |
| ensp00000064724 | cldn11 | 6940 | 0.716646014 | 46.73731658 | 3.97E-10 |
| ensp00000377265 | tfap2b | 334 | 0.03448988 | 22.32258065 | 1.59E-09 |
| ensp00000420295 | pde6b | 2724 | 0.281288724 | 20.58120513 | 2.36E-09 |
| ensp00000270279 | cblc | 4731 | 0.488537794 | 17.0283617 | 8.79E-09 |
| ensp00000297268 | col1a2 | 5661 | 0.584572491 | 8.954545455 | 9.88E-09 |
| ensp00000363298 | bspry | 7345 | 0.758467575 | 29.10415625 | 2.29E-08 |

| | | | | | |
|---|---|---|---|---|---|
| ensp00000332643 | ndn | 3762 | 0.388475836 | 38.32463854 | 2.98E-08 |
| ensp00000284440 | uchl1 | 3180 | 0.328376704 | 5.007540453 | 3.59E-08 |
| ensp00000220772 | sfrp1 | 3693 | 0.381350682 | 28.04589729 | 4.19E-08 |
| ensp00000351206 | txlnb | 7377 | 0.761771995 | 24.56 | 6.23E-08 |
| ensp00000291009 | pip | 9186 | 0.948574969 | 19.4999801 | 1.93E-07 |
| ensp00000354376 | rab25 | 7494 | 0.773853779 | 30.14443333 | 3.60E-07 |
| ensp00000260197 | sorl1 | 8269 | 0.853882693 | 18.9327572 | 5.26E-07 |
| ensp00000306099 | fgb | 5420 | 0.55968608 | -2.5778947 | 6.46E-07 |
| ensp00000368727 | xdh | 7662 | 0.791201983 | 2.361702128 | 7.66E-07 |
| ensp00000296026 | cxcl3 | 3978 | 0.410780669 | 2.197457478 | 7.83E-07 |
| ensp00000309757 | lpl | 5103 | 0.526951673 | 15.17477201 | 8.16E-07 |
| ensp00000255192 | bhmt2 | 7900 | 0.815778604 | 20.72387259 | 8.42E-07 |
| ensp00000156626 | st6galnac1 | 9320 | 0.962412226 | 17.15466717 | 8.63E-07 |
| ensp00000259455 | gabbr2 | 4239 | 0.437732342 | 39.11545984 | 9.32E-07 |
| ensp00000296849 | nkd2 | 3549 | 0.366480793 | -2.766307199 | 9.45E-07 |
| ensp00000262262 | cd33 | 8714 | 0.899834779 | -22.43399286 | 1.01E-06 |
| ensp00000330658 | pappa | 5744 | 0.593143329 | 3.403507519 | 1.04E-06 |
| ensp00000279441 | mmp10 | 5504 | 0.568360182 | 16.341675 | 1.08E-06 |
| ensp00000340200 | glyat | 9084 | 0.938042131 | -7.468387931 | 1.15E-06 |
| ensp00000244458 | pacsin1 | 5553 | 0.573420074 | -2.465950909 | 1.23E-06 |
| ensp00000327107 | pdzd3 | 532 | 0.054935977 | -4.927565661 | 1.29E-06 |
| ensp00000398028 | npr3 | 8551 | 0.883002891 | -2.699724518 | 1.38E-06 |
| ensp00000367462 | olah | 7458 | 0.770136307 | 9.140350877 | 1.39E-06 |
| ensp00000299106 | jam3 | 5214 | 0.538413879 | 23.47543193 | 1.48E-06 |
| ensp00000309270 | chst1 | 9289 | 0.95921107 | -13.54070128 | 1.67E-06 |
| ensp00000258499 | usp44 | 520 | 0.053696819 | 18.84692975 | 1.83E-06 |
| ensp00000296435 | camp | 603 | 0.062267658 | -14.70454545 | 1.91E-06 |
| ensp00000306361 | fga | 5419 | 0.559582817 | -13.74192662 | 1.91E-06 |
| ensp00000355627 | agt | 8848 | 0.913672036 | -2.069642448 | 2.00E-06 |
| ensp00000229030 | fzd10 | 3730 | 0.385171417 | 20.64635445 | 2.14E-06 |
| ensp00000321853 | serpinf2 | 5259 | 0.543060719 | -3.475370806 | 2.33E-06 |
| ensp00000263341 | il1b | 4389 | 0.453221809 | 2.153845275 | 2.68E-06 |
| ensp00000372160 | dok6 | 4726 | 0.488021479 | 11.29884483 | 2.89E-06 |
| ensp00000261254 | ccnd2 | 2763 | 0.285315985 | 2.156579998 | 3.01E-06 |
| ensp00000369071 | postn | 7251 | 0.748760843 | 28.15475 | 3.31E-06 |
| ensp00000355920 | slc22a2 | 6993 | 0.722118959 | 11.50000079 | 3.60E-06 |
| ensp00000306884 | cxcl11 | 4087 | 0.422036349 | 10.19492389 | 3.66E-06 |
| ensp00000260795 | fgfr3 | 4877 | 0.503614209 | -2.836495762 | 3.81E-06 |
| ensp00000410294 | fgfr2 | 4791 | 0.494733581 | -3.046526502 | 4.15E-06 |
| ensp00000417583 | st6galnac5 | 9234 | 0.953531599 | 11.95276532 | 4.74E-06 |
| ensp00000261195 | gys2 | 3635 | 0.375361421 | -2.009956008 | 5.21E-06 |
| ensp00000371372 | atp12a | 8656 | 0.893845518 | 14.38774042 | 5.57E-06 |
| ensp00000294339 | tal1 | 3153 | 0.3255886 | 5.032721806 | 5.85E-06 |
| ensp00000302648 | nrtn | 6002 | 0.619785213 | -4.76539378 | 6.21E-06 |
| ensp00000216274 | ripk3 | 4326 | 0.446716233 | 10.72331959 | 6.23E-06 |
| ensp00000355556 | gng4 | 4194 | 0.433085502 | 2.280995163 | 6.46E-06 |
| ensp00000327453 | acsm2b | 8508 | 0.878562577 | -3.045171811 | 8.63E-06 |

| | | | | | |
|---|---|---|---|---|---|
| ensp00000365301 | fgf14 | 5029 | 0.519310202 | -2.042141398 | 8.79E-06 |
| ensp00000282111 | tcf7l1 | 3411 | 0.352230483 | 11.79311158 | 9.23E-06 |
| ensp00000320935 | slc2a4 | 3712 | 0.383312681 | -9.563322953 | 9.90E-06 |
| ensp00000263182 | bbox1 | 8124 | 0.838909542 | -12.59481673 | 9.91E-06 |
| ensp00000354677 | gpx7 | 6651 | 0.686802974 | 7.051725155 | 1.02E-05 |
| ensp00000226524 | pf4v1 | 3952 | 0.408095828 | 12.05982051 | 1.03E-05 |
| ensp00000414330 | rimkla | 6440 | 0.665014457 | 11.74011864 | 1.07E-05 |
| ensp00000288221 | erc2 | 7029 | 0.725836431 | 2.736919283 | 1.09E-05 |
| ensp00000362171 | nap1l3 | 26 | 0.002684841 | 8.342867624 | 1.13E-05 |
| ensp00000260404 | pak6 | 4735 | 0.488950847 | 8.262298468 | 1.15E-05 |
| ensp00000359074 | l1cam | 5446 | 0.562370921 | 18.02610263 | 1.17E-05 |
| ensp00000316328 | ciita | 5324 | 0.549772821 | -10.93374153 | 1.31E-05 |
| ensp00000379310 | casc1 | 1584 | 0.163568773 | -2.523678254 | 1.33E-05 |
| ensp00000312987 | hnf4a | 3291 | 0.33983891 | -5.543046122 | 1.33E-05 |
| ensp00000368632 | gata3 | 3232 | 0.333746386 | 19.50494108 | 1.43E-05 |
| ensp00000383558 | gcgr | 4125 | 0.425960347 | 17.08108108 | 1.59E-05 |
| ensp00000309572 | tert | 2947 | 0.304316398 | -2.478050696 | 1.93E-05 |
| ensp00000301242 | ppp1r14a | 3676 | 0.379595209 | 2.167777825 | 1.96E-05 |
| ensp00000330959 | il1r2 | 4391 | 0.453428335 | -2.00395962 | 2.10E-05 |
| ensp00000348170 | hp | 8651 | 0.893329203 | -2.368095232 | 2.12E-05 |
| ensp00000282728 | hhex | 9383 | 0.968917803 | 12.70422726 | 2.35E-05 |
| ensp00000304930 | sostdc1 | 5873 | 0.606464271 | -5.391145901 | 2.38E-05 |
| ensp00000371471 | rsad2 | 5347 | 0.552147873 | 24.71424063 | 2.62E-05 |
| ensp00000359478 | abcc2 | 411 | 0.04244114 | -2.303585235 | 2.66E-05 |
| ensp00000295550 | col6a3 | 5688 | 0.587360595 | 16.51404819 | 2.84E-05 |
| ensp00000264708 | pomc | 3865 | 0.399111937 | 11.83694366 | 2.94E-05 |
| ensp00000172229 | ngfr | 4432 | 0.457662123 | -8.698853012 | 3.09E-05 |
| ensp00000381876 | daam2 | 3597 | 0.371437423 | -2.658357341 | 3.10E-05 |
| ensp00000364898 | syk | 4836 | 0.499380421 | 21.21426407 | 3.10E-05 |
| ensp00000350928 | gad1 | 7909 | 0.816707972 | 15.57260127 | 3.71E-05 |
| ensp00000258743 | il6 | 4470 | 0.461586121 | 2.033492823 | 3.86E-05 |
| ensp00000264260 | il18rap | 4285 | 0.442482445 | -15.54783961 | 4.05E-05 |
| ensp00000265944 | myo3a | 6056 | 0.625361421 | 3.104516949 | 4.20E-05 |
| ensp00000259365 | tmod1 | 5808 | 0.599752169 | 7.140842556 | 4.30E-05 |
| ensp00000261233 | irak3 | 4345 | 0.448678232 | 11.61269128 | 4.42E-05 |
| ensp00000220809 | plat | 5318 | 0.549153242 | 2.534677716 | 4.53E-05 |
| ensp00000351190 | itih2 | 6867 | 0.709107807 | -9.540117401 | 4.72E-05 |
| ensp00000226317 | cxcl6 | 4022 | 0.415324246 | 9.928033042 | 4.78E-05 |
| ensp00000310721 | cyp7b1 | 7843 | 0.809892606 | -2.151328221 | 4.87E-05 |
| ensp00000263621 | elane | 5414 | 0.559066501 | -5.95044907 | 5.04E-05 |
| ensp00000284240 | thy1 | 5422 | 0.559892606 | 3.01515322 | 5.18E-05 |
| ensp00000222792 | chn2 | 7872 | 0.812887237 | 4.44528847 | 5.34E-05 |
| ensp00000265709 | ank1 | 6841 | 0.706422966 | 3.365934206 | 5.63E-05 |
| ensp00000263339 | il1a | 4388 | 0.453118546 | 25.83333333 | 5.99E-05 |
| ensp00000394033 | kcnk2 | 9358 | 0.966336225 | -2.94778133 | 6.12E-05 |
| ensp00000335083 | ppp2r2c | 3265 | 0.337154069 | 10.7452234 | 6.27E-05 |
| ensp00000255082 | acy3 | 7956 | 0.821561338 | -6.386378939 | 6.77E-05 |

| | | | | | |
|---|---|---|---|---|---|
| ensp00000342445 | cldn4 | 6958 | 0.71850475 | 2.311060662 | 6.82E-05 |
| ensp00000254262 | c19orf40 | 2132 | 0.22015696 | -2.321802524 | 7.08E-05 |
| ensp00000327145 | flnc | 5683 | 0.586844279 | 2.102519851 | 7.93E-05 |
| ensp00000360918 | ch25h | 7817 | 0.807207765 | 15.7111 | 8.15E-05 |
| ensp00000351682 | cndp1 | 7968 | 0.822800496 | -9.748673662 | 8.22E-05 |
| ensp00000292513 | ptger1 | 4210 | 0.434737712 | 13.25263329 | 8.35E-05 |
| ensp00000275815 | epha1 | 4813 | 0.49700537 | 8.28358209 | 8.42E-05 |
| ensp00000415941 | c4b | 6462 | 0.667286245 | -2.135488051 | 8.57E-05 |
| ensp00000364321 | c4b | 7453 | 0.769619992 | -2.135488051 | 8.57E-05 |
| ensp00000410321 | ly6g5c | 6509 | 0.672139612 | -2.583778941 | 8.69E-05 |
| ensp00000289422 | nrg2 | 4649 | 0.480070219 | 6.826096101 | 8.70E-05 |
| ensp00000344874 | gucy1a2 | 2353 | 0.242978108 | -2.188732882 | 9.71E-05 |
| ensp00000347379 | ocln | 6965 | 0.719227592 | 6.336273128 | 9.76E-05 |
| ensp00000330074 | hist1h1b | 2012 | 0.207765386 | 18.91840596 | 9.78E-05 |
| ensp00000264563 | il11 | 6014 | 0.62102437 | 2.408425824 | 9.86E-05 |
| ensp00000410668 | tnf | 4314 | 0.445477076 | 15 | 0.000100813 |
| ensp00000392858 | tnf | 4353 | 0.449504337 | 15 | 0.000100813 |
| ensp00000398698 | tnf | 4354 | 0.4496076 | 15 | 0.000100813 |
| ensp00000365290 | tnf | 6348 | 0.65551425 | 15 | 0.000100813 |
| ensp00000283921 | hoxa10 | 137 | 0.014147047 | 9.976653706 | 0.000101158 |
| ensp00000264257 | il1rl2 | 8757 | 0.904275093 | 4.180453316 | 0.000102678 |
| ensp00000284818 | ly96 | 4418 | 0.456216439 | 2.16111 | 0.000103689 |
| ensp00000368966 | trpc3 | 5123 | 0.529016935 | -3.765552354 | 0.000105712 |
| ensp00000306157 | il7r | 4596 | 0.474597274 | 7.666674419 | 0.000105783 |
| ensp00000308032 | cyp2s1 | 7800 | 0.805452292 | 6.402355954 | 0.000108339 |
| ensp00000228938 | mgp | 5149 | 0.531701776 | 13.21152576 | 0.000109874 |
| ensp00000296280 | masp1 | 6971 | 0.719847171 | 2.343352668 | 0.000109957 |
| ensp00000343477 | runx3 | 3664 | 0.378356051 | -10.11904762 | 0.000110619 |
| ensp00000367830 | prkcz | 4462 | 0.460760017 | 2.173560877 | 0.000110685 |
| ensp00000290341 | igf2bp1 | 1945 | 0.200846758 | 2.523163083 | 0.000112797 |
| ensp00000261205 | syt1 | 7079 | 0.730999587 | 11.88289189 | 0.000113513 |
| ensp00000367714 | hes5 | 3276 | 0.338289963 | 10.26597901 | 0.000115042 |
| ensp00000217407 | lbp | 4368 | 0.451053284 | -3.325651766 | 0.000115942 |
| ensp00000254508 | nup210 | 1338 | 0.138166047 | 35.36842105 | 0.000116698 |
| ensp00000263080 | aspa | 7957 | 0.821664601 | -10.79278378 | 0.000119527 |
| ensp00000320378 | slc7a8 | 8668 | 0.895084676 | 19.62789578 | 0.000121123 |
| ensp00000282356 | camk4 | 3626 | 0.374432053 | 6.20855947 | 0.000122135 |
| ensp00000306888 | fam151a | 7592 | 0.783973565 | -6.399118421 | 0.000123373 |
| ensp00000357040 | vangl2 | 3540 | 0.365551425 | 25.09087651 | 0.000124046 |
| ensp00000338185 | plcb1 | 4457 | 0.460243701 | 8.916666667 | 0.00012471 |
| ensp00000364475 | fbp1 | 7280 | 0.751755473 | 14.99002499 | 0.000130016 |
| ensp00000355884 | mark1 | 2992 | 0.308963238 | 10.79103672 | 0.000130542 |
| ensp00000339292 | cldn14 | 6963 | 0.719021066 | 14.86486815 | 0.000138106 |
| ensp00000296140 | ccr1 | 4050 | 0.418215613 | -4.435429681 | 0.000140211 |
| ensp00000243213 | il13ra2 | 4592 | 0.474184221 | 29.98513431 | 0.00014043 |
| ensp00000296414 | dapp1 | 4797 | 0.49535316 | 10.9395298 | 0.000142421 |
| ensp00000342114 | icam4 | 5495 | 0.567430814 | 3.498495495 | 0.00014881 |

| | | | | | |
|---|---|---|---|---|---|
| ensp00000337731 | cideb | 4153 | 0.428851714 | -2.568388569 | 0.00015467 |
| ensp00000358301 | adrb1 | 3819 | 0.394361834 | 11.58930499 | 0.000159368 |
| ensp00000266085 | timp3 | 6030 | 0.62267658 | 21.15043261 | 0.00016184 |
| ensp00000167586 | krt14 | 3446 | 0.355844692 | 33.41993316 | 0.000162532 |
| ensp00000298841 | serpina4 | 9340 | 0.964477489 | -3.000003386 | 0.000163405 |
| ensp00000258873 | acsbg1 | 7424 | 0.766625361 | -4.70270588 | 0.000163564 |
| ensp00000342235 | erbb4 | 4694 | 0.484717059 | 14.9926 | 0.000172766 |
| ensp00000353362 | cacna1a | 4242 | 0.438042131 | 19.8111 | 0.000188387 |
| ensp00000340396 | gbp5 | 6568 | 0.678232135 | 7.716490868 | 0.000191289 |
| ensp00000361943 | heyl | 3216 | 0.332094176 | 11.25518018 | 0.000192667 |
| ensp00000272134 | lefty1 | 5813 | 0.600268484 | -5.32751761 | 0.000200717 |
| ensp00000356905 | vnn1 | 8380 | 0.865344899 | 11.61445783 | 0.000203037 |
| ensp00000228850 | akap3 | 8638 | 0.891986782 | 10.21593206 | 0.000206187 |
| ensp00000293272 | ccl5 | 4058 | 0.419041718 | 5.829622222 | 0.000208656 |
| ensp00000234371 | kiss1r | 4274 | 0.441346551 | 4.619836934 | 0.00020948 |
| ensp00000354490 | atp1a2 | 9053 | 0.934840975 | -32.49017647 | 0.000217738 |
| ensp00000332052 | pcsk6 | 7440 | 0.768277571 | -6.497647887 | 0.000225379 |
| ensp00000359410 | ephx4 | 7459 | 0.77023957 | 2.004259799 | 0.000231058 |
| ensp00000173229 | ntn1 | 4856 | 0.501445684 | 21.72220833 | 0.000232817 |
| ensp00000297991 | aqp3 | 5333 | 0.550702189 | -3.356671851 | 0.000240107 |
| ensp00000378359 | adh6 | 7781 | 0.803490293 | -5.936835856 | 0.000245435 |
| ensp00000279804 | ctf1 | 6015 | 0.621127633 | 2.154882227 | 0.000248724 |
| ensp00000285018 | wnt7a | 3757 | 0.387959521 | 18.36170213 | 0.000249232 |
| ensp00000356991 | pvrl4 | 5595 | 0.577757125 | 7.569582524 | 0.000250749 |
| ensp00000296871 | csf2 | 4562 | 0.471086328 | 6.533771242 | 0.000252431 |
| ensp00000296027 | cxcl5 | 4061 | 0.419351508 | 2.500287271 | 0.000260221 |
| ensp00000350616 | ddc | 7945 | 0.820425444 | -12.06565152 | 0.000261183 |
| ensp00000231751 | ltf | 5437 | 0.561441553 | 3.688933896 | 0.000270783 |
| ensp00000225964 | col1a1 | 5662 | 0.584675754 | 15.35682143 | 0.000289198 |
| ensp00000253513 | ido1 | 7944 | 0.820322181 | 7.657572727 | 0.000299825 |
| ensp00000282641 | a1cf | 103 | 0.010636101 | -6.079597015 | 0.000311038 |
| ensp00000307046 | sdc2 | 5650 | 0.583436596 | -2.888027243 | 0.000312413 |
| ensp00000337383 | nlrp3 | 2741 | 0.283044197 | 14.08917617 | 0.000314775 |
| ensp00000290399 | sim2 | 418 | 0.043163982 | 18.41665625 | 0.000322042 |
| ensp00000345512 | sema6a | 6293 | 0.649834779 | 5.151829763 | 0.000329565 |
| ensp00000363157 | tnfsf15 | 6765 | 0.698574969 | 12.05299474 | 0.000337218 |
| ensp00000298472 | slc18a2 | 7030 | 0.725939694 | 15.239103 | 0.000345974 |
| ensp00000379895 | gatm | 8049 | 0.831164808 | 2.57801126 | 0.00035331 |
| ensp00000307694 | kcns1 | 8449 | 0.872470054 | 8.969574536 | 0.000356517 |
| ensp00000308012 | pabpc5 | 258 | 0.026641884 | 23.43753662 | 0.000366896 |
| ensp00000344173 | grm8 | 8340 | 0.861214374 | 6.771611111 | 0.000371604 |
| ensp00000354416 | ccl28 | 3984 | 0.411400248 | 4.05166333 | 0.000383339 |
| ensp00000252729 | cacng6 | 5172 | 0.534076828 | 6.160533057 | 0.000385731 |
| ensp00000296641 | f2rl2 | 4414 | 0.455803387 | 12.03669912 | 0.000389644 |
| ensp00000361508 | pltp | 7867 | 0.812370921 | 2.236915709 | 0.00039091 |
| ensp00000296370 | s100p | 108 | 0.011152416 | 13.04348535 | 0.000391007 |
| ensp00000320886 | mlxipl | 6862 | 0.708591491 | -5.591463415 | 0.000391032 |

| | | | | | |
|---|---|---|---|---|---|
| ensp00000409605 | trim15 | 6426 | 0.663568773 | -9.037300699 | 0.000394494 |
| ensp00000403221 | trim15 | 6770 | 0.699091285 | -9.037300699 | 0.000394494 |
| ensp00000297450 | angpt1 | 4717 | 0.487092111 | -4.061575454 | 0.000406823 |
| ensp00000308576 | rhod | 5007 | 0.517038414 | 7.689050881 | 0.000407363 |
| ensp00000351671 | ccl20 | 4060 | 0.419248245 | 9.987128991 | 0.00040864 |
| ensp00000354207 | ntrk3 | 4738 | 0.489260636 | -3.211367816 | 0.000411597 |
| ensp00000340191 | fpr2 | 4131 | 0.426579926 | -4.893333333 | 0.000411854 |
| ensp00000264399 | prkg2 | 3674 | 0.379388682 | 2.27184466 | 0.000419689 |
| ensp00000263388 | notch3 | 3350 | 0.345931433 | 2.317444816 | 0.000426113 |
| ensp00000309148 | klk6 | 7438 | 0.768071045 | 4.192997118 | 0.00042891 |
| ensp00000346693 | elovl2 | 7559 | 0.780565882 | 3.787798079 | 0.000429405 |
| ensp00000287641 | sst | 3946 | 0.407476249 | 12.5725 | 0.000433269 |
| ensp00000225275 | mpo | 4624 | 0.477488641 | 3.37037037 | 0.000446471 |
| ensp00000253754 | pdlim4 | 6838 | 0.706113176 | 12.92025333 | 0.000446832 |
| ensp00000384264 | cnga1 | 3898 | 0.40251962 | 7.223459652 | 0.000462271 |
| ensp00000312021 | fut1 | 9240 | 0.954151177 | 2.694159794 | 0.000474401 |
| ensp00000338171 | skap1 | 6173 | 0.637443205 | 3.330409357 | 0.000474553 |
| ensp00000363985 | mtmr8 | 9114 | 0.941140025 | 4.137174397 | 0.000478122 |
| ensp00000250448 | foxa1 | 5483 | 0.566191656 | 9.674901235 | 0.000481448 |
| ensp00000201031 | tfap2c | 335 | 0.034593143 | -2.872427984 | 0.000494946 |
| ensp00000270800 | il22ra1 | 4621 | 0.477178852 | -2.812717645 | 0.000498527 |
| ensp00000416387 | fblim1 | 5545 | 0.572593969 | 4.240440681 | 0.000522337 |
| ensp00000318867 | cyp8b1 | 7824 | 0.807930607 | -6.566329881 | 0.000529853 |
| ensp00000358042 | qrsl1 | 68 | 0.007021892 | -2.606190065 | 0.000541189 |
| ensp00000255266 | pde6a | 2725 | 0.281391987 | -9.937875815 | 0.000553976 |
| ensp00000291539 | pde9a | 2744 | 0.283353986 | 11.67157353 | 0.000557508 |
| ensp00000278927 | esam | 6976 | 0.720363486 | 4.07246087 | 0.000564559 |
| ensp00000277480 | lcn2 | 7529 | 0.777467988 | 2.038852459 | 0.000573697 |
| ensp00000367059 | espn | 6547 | 0.67606361 | -2.370971218 | 0.00058212 |
| ensp00000242338 | cntfr | 4628 | 0.477901694 | -4.909469136 | 0.000584223 |
| ensp00000244709 | trem1 | 8754 | 0.903965304 | 11.31032646 | 0.000585961 |
| ensp00000396774 | muc20 | 9268 | 0.957042544 | 4.094853659 | 0.000604168 |
| ensp00000304767 | p2ry1 | 4253 | 0.439178026 | -4.400560965 | 0.00061902 |
| ensp00000331504 | fes | 4659 | 0.48110285 | 10.86573611 | 0.00062077 |
| ensp00000329797 | cadm1 | 9395 | 0.97015696 | 2.262590832 | 0.000628584 |
| ensp00000259988 | fgfbp1 | 7132 | 0.736472532 | 2.086206897 | 0.000628741 |
| ensp00000256935 | dock2 | 4871 | 0.50299463 | 2.075075144 | 0.000640681 |
| ensp00000259206 | il1rn | 8756 | 0.90417183 | 3.430533142 | 0.000646588 |
| ensp00000312397 | klhl3 | 2138 | 0.220776539 | 8.573333333 | 0.000657724 |
| ensp00000314151 | klk3 | 5384 | 0.555968608 | -21.39395455 | 0.000659988 |
| ensp00000297404 | kcnv1 | 8472 | 0.874845105 | -3.929245936 | 0.000663962 |
| ensp00000304004 | foxa3 | 5485 | 0.566398183 | 2.149138382 | 0.000674311 |
| ensp00000254691 | card6 | 7588 | 0.783560512 | 2.090784044 | 0.00069375 |
| ensp00000406367 | gpr124 | 6290 | 0.64952499 | 2.133021824 | 0.000717474 |
| ensp00000249750 | aldh1a2 | 7729 | 0.798120611 | 4.58219335 | 0.000727153 |
| ensp00000361214 | nrg3 | 9183 | 0.94826518 | 15.02439024 | 0.000731798 |
| ensp00000263126 | akr1c4 | 7756 | 0.800908715 | -3.338503876 | 0.000736641 |

| | | | | | |
|---|---|---|---|---|---|
| ensp00000353475 | cldn7 | 6939 | 0.716542751 | 2.236950469 | 0.000737108 |
| ensp00000299333 | scn3b | 286 | 0.029533251 | 10.20224696 | 0.000738427 |
| ensp00000258411 | wnt10a | 3720 | 0.384138786 | 3.658335 | 0.000744936 |
| ensp00000354822 | xaf1 | 5363 | 0.553800083 | 8.348423845 | 0.000769253 |
| ensp00000257818 | lmo2 | 7139 | 0.737195374 | 17.66369233 | 0.000796882 |
| ensp00000278187 | gas2 | 6702 | 0.692069393 | -3.598331796 | 0.000804642 |
| ensp00000265643 | gal | 4036 | 0.41676993 | 9.073721154 | 0.000804845 |
| ensp00000361366 | sftpd | 3294 | 0.340148699 | 7.959649309 | 0.000812159 |
| ensp00000305603 | fut3 | 9242 | 0.954357703 | 16.13636762 | 0.000816259 |
| ensp00000291670 | ftcd | 8260 | 0.852953325 | 4.066668966 | 0.000818603 |
| ensp00000263925 | lnx1 | 6800 | 0.702189178 | 7.854649915 | 0.00084086 |
| ensp00000353198 | pyy | 3951 | 0.407992565 | 5.625857143 | 0.000869072 |
| ensp00000420168 | gsta2 | 7805 | 0.805968608 | -2.053712943 | 0.000871433 |
| ensp00000258443 | edar | 113 | 0.011668732 | 6.442307692 | 0.000882326 |
| ensp00000289429 | cd1a | 7337 | 0.75764147 | 3.995438356 | 0.000897145 |
| ensp00000369003 | trpc4 | 5124 | 0.529120198 | 3.391553037 | 0.000907518 |
| ensp00000362334 | psmb2 | 2515 | 0.259706733 | 2.064373016 | 0.000907935 |
| ensp00000261523 | rora | 3320 | 0.34283354 | 2.164024078 | 0.00091683 |
| ensp00000287713 | nmnat2 | 2775 | 0.286555143 | 9.16 | 0.000925739 |
| ensp00000352064 | klrc1 | 5982 | 0.61771995 | 6.743716957 | 0.000930448 |
| ensp00000302707 | fpr1 | 4013 | 0.414394878 | -4.43949313 | 0.000938893 |
| ensp00000322390 | fgf13 | 5044 | 0.520859149 | 3.629627778 | 0.000944841 |
| ensp00000324527 | myo1d | 7237 | 0.747315159 | 2.064266975 | 0.00095134 |
| ensp00000317145 | gdnf | 4764 | 0.491945477 | 2.506666667 | 0.000953695 |
| ensp00000362784 | or5c1 | 9514 | 0.982445271 | 2.515994704 | 0.000966207 |
| ensp00000277120 | ntrk2 | 4614 | 0.47645601 | 3.437698628 | 0.000967148 |
| ensp00000379204 | bmp7 | 5815 | 0.60047501 | 9.076278878 | 0.000988514 |
| ensp00000410443 | hla-dra | 5721 | 0.590768278 | -3.258780737 | 0.00100605 |
| ensp00000372608 | hla-dra | 5724 | 0.591078067 | -3.258780737 | 0.00100605 |
| ensp00000372746 | hla-dra | 6381 | 0.658921933 | -3.258780737 | 0.00100605 |
| ensp00000402951 | hla-dra | 6501 | 0.671313507 | -3.258780737 | 0.00100605 |
| ensp00000405295 | hla-dra | 9175 | 0.947439075 | -3.258780737 | 0.00100605 |
| ensp00000348888 | pigr | 5184 | 0.535315985 | 8.12586145 | 0.00103682 |
| ensp00000358423 | rragd | 3548 | 0.36637753 | 2.215434084 | 0.00105114 |
| ensp00000282499 | gria4 | 6747 | 0.696716233 | 6.855716418 | 0.00105278 |
| ensp00000307875 | b3gat1 | 6178 | 0.637959521 | 10.12697619 | 0.00105394 |
| ensp00000295156 | vsnl1 | 8115 | 0.837980173 | -2.456845274 | 0.00105602 |
| ensp00000255189 | dmgdh | 8162 | 0.84283354 | 3.358125678 | 0.00107681 |
| ensp00000375748 | fut2 | 9241 | 0.95425444 | 10.67126119 | 0.00108607 |
| ensp00000217086 | sall4 | 7246 | 0.748244527 | 3.858333333 | 0.00108925 |
| ensp00000388548 | cited1 | 3217 | 0.332197439 | -2.125377799 | 0.00109019 |
| ensp00000236147 | sell | 6977 | 0.720466749 | 5.54237476 | 0.00109823 |
| ensp00000357461 | chrnb2 | 8374 | 0.86472532 | 2.688268519 | 0.00110949 |
| ensp00000362616 | nap1l2 | 25 | 0.002581578 | 2.229421053 | 0.00114621 |
| ensp00000291294 | ptgir | 3853 | 0.39787278 | 9.234840909 | 0.00115384 |
| ensp00000341032 | wnt7b | 3699 | 0.38197026 | 2.378927911 | 0.00116582 |
| ensp00000003084 | cftr | 3256 | 0.336224701 | 11.17045455 | 0.00121097 |

| | | | | | |
|---|---|---|---|---|---|
| ensp00000381654 | hmgcll1 | 8205 | 0.847273854 | 3.946425483 | 0.00121305 |
| ensp00000360806 | kcnb1 | 8389 | 0.866274267 | -3.980178378 | 0.00121332 |
| ensp00000420419 | jam2 | 5213 | 0.538310615 | 2.447920967 | 0.00122548 |
| ensp00000260682 | cyp2c9 | 7752 | 0.800495663 | -3.790232759 | 0.00122733 |
| ensp00000287020 | gdf6 | 5925 | 0.611833953 | 2.971303337 | 0.00123088 |
| ensp00000301732 | abca3 | 1120 | 0.115654688 | 2.304288991 | 0.00123502 |
| ensp00000288135 | kit | 4683 | 0.483581165 | 14.87877273 | 0.00124458 |
| ensp00000374309 | lama1 | 5570 | 0.575175547 | 2.254828977 | 0.00125813 |
| ensp00000341682 | slc26a9 | 6924 | 0.714993804 | 5.672131148 | 0.00128743 |
| ensp00000263686 | selp | 6078 | 0.627633209 | 5.862976562 | 0.0013312 |
| ensp00000357013 | cd244 | 5995 | 0.619062371 | 3.562279871 | 0.00135314 |
| ensp00000369323 | npnt | 6080 | 0.627839736 | 7.522699602 | 0.00135654 |
| ensp00000216629 | bdkrb1 | 4133 | 0.426786452 | 2.438705744 | 0.00138145 |
| ensp00000265728 | dbf4 | 2326 | 0.240190004 | -3.208462559 | 0.00139923 |
| ensp00000300406 | gngt2 | 4244 | 0.438248658 | 4.173 | 0.00141045 |
| ensp00000227752 | il10ra | 4641 | 0.479244114 | 9.337016208 | 0.00142385 |
| ensp00000393847 | pla2g10 | 7579 | 0.782631144 | 12.22222222 | 0.00145371 |
| ensp00000361009 | prex1 | 4310 | 0.445064023 | 2.53091308 | 0.00147265 |
| ensp00000285393 | atp6v0d2 | 8558 | 0.883725733 | 4.736609493 | 0.00153481 |
| ensp00000355245 | pax9 | 625 | 0.064539447 | -2.042850455 | 0.00153853 |
| ensp00000329668 | shc4 | 4848 | 0.500619579 | 2.510723217 | 0.00156615 |
| ensp00000305355 | prkcb | 5216 | 0.538620405 | -11.06915007 | 0.00157441 |
| ensp00000373024 | c6orf15 | 6334 | 0.654068567 | 8.120682655 | 0.00157648 |
| ensp00000261374 | hs3st2 | 6068 | 0.626600578 | 4.147854417 | 0.00158546 |
| ensp00000354478 | dlx1 | 1325 | 0.136823627 | 2.72444 | 0.00158805 |
| ensp00000311997 | nefh | 5803 | 0.599235853 | 4.125568493 | 0.00159632 |
| ensp00000358309 | epha7 | 4809 | 0.496592317 | 13.66240298 | 0.00160497 |
| ensp00000356505 | ncf2 | 5279 | 0.545125981 | 4.906145631 | 0.00162509 |
| ensp00000245479 | sox9 | 1758 | 0.181536555 | 5.547356454 | 0.00163661 |
| ensp00000359603 | col24a1 | 5698 | 0.588393226 | 3.747743243 | 0.00165979 |
| ensp00000355378 | plxnb3 | 6236 | 0.643948781 | 3.530927835 | 0.00166183 |
| ensp00000360561 | entpd8 | 2616 | 0.270136307 | -5.09375 | 0.00174171 |
| ensp00000155840 | kcnq1 | 8273 | 0.854295746 | 13.92360417 | 0.00176815 |
| ensp00000365943 | pcsk5 | 6258 | 0.64622057 | 2.404695593 | 0.0017839 |
| ensp00000377303 | renbp | 8856 | 0.914498141 | 2.379312909 | 0.00178769 |
| ensp00000362299 | eng | 3465 | 0.357806691 | 2.375110758 | 0.00183576 |
| ensp00000262752 | rps6ka6 | 4454 | 0.459933912 | 6.074764521 | 0.00184097 |
| ensp00000285949 | cyp26c1 | 7664 | 0.791408509 | 2.30658642 | 0.00185084 |
| ensp00000295731 | ihh | 5853 | 0.604399009 | -4.138646018 | 0.00185817 |
| ensp00000340937 | col17a1 | 5697 | 0.588289963 | 3.474293284 | 0.00187266 |
| ensp00000260187 | usp2 | 2982 | 0.307930607 | -2.591016178 | 0.0018845 |
| ensp00000310244 | rasgrp1 | 5067 | 0.523234201 | 10.97143484 | 0.00189432 |
| ensp00000363680 | eda | 114 | 0.011771995 | 6.245617225 | 0.00190006 |
| ensp00000304408 | col3a1 | 5660 | 0.584469228 | 24.16664286 | 0.00190219 |
| ensp00000220507 | rhov | 5073 | 0.523853779 | 2.264705882 | 0.00192221 |
| ensp00000301908 | pnoc | 4009 | 0.413981826 | 11.00002658 | 0.0019231 |
| ensp00000352561 | calcr | 3854 | 0.397976043 | -2.050762099 | 0.00193555 |

| | | | | | |
|---|---|---|---|---|---|
| ensp00000257549 | sds | 8150 | 0.841594382 | -2.041204946 | 0.00193727 |
| ensp00000330442 | plb1 | 7575 | 0.782218092 | 2.057804905 | 0.00195238 |
| ensp00000294117 | gng3 | 4193 | 0.432982239 | -3.991602517 | 0.00196417 |
| ensp00000385149 | neu4 | 8139 | 0.840458488 | -4.69307163 | 0.0019793 |
| ensp00000252971 | mnx1 | 9381 | 0.968711276 | 9.34321638 | 0.0019813 |
| ensp00000271636 | cgn | 5144 | 0.531185461 | 2.025493608 | 0.00201688 |
| ensp00000205948 | apoh | 5787 | 0.597583643 | -4.41176656 | 0.00212133 |
| ensp00000200307 | ccl7 | 4077 | 0.421003717 | 6.596389516 | 0.00213953 |
| ensp00000288490 | dgki | 7200 | 0.743494424 | 5.563097117 | 0.00214467 |
| ensp00000302269 | vav1 | 4831 | 0.498864106 | 8.061946045 | 0.00216638 |
| ensp00000289746 | cdh15 | 4579 | 0.472841801 | -3.013908521 | 0.00218102 |
| ensp00000299339 | cldn10 | 6957 | 0.718401487 | 11.38725 | 0.00219681 |
| ensp00000288139 | cacna1d | 5174 | 0.534283354 | -3.67293478 | 0.00226372 |
| ensp00000369962 | igsf5 | 5212 | 0.538207352 | -5.372006149 | 0.00226662 |
| ensp00000354280 | prss3 | 5619 | 0.58023544 | 3.344739927 | 0.00230447 |
| ensp00000417229 | eif2a | 1283 | 0.132486576 | -2.374434138 | 0.00241877 |
| ensp00000296029 | pf4 | 4336 | 0.447748864 | 5.332610865 | 0.00244271 |
| ensp00000249016 | mchr1 | 4104 | 0.423791822 | 9.590485714 | 0.00244593 |
| ensp00000332116 | pde4b | 2714 | 0.280256093 | 10.5 | 0.00245842 |
| ensp00000386896 | itga6 | 5563 | 0.574452705 | 3.319878658 | 0.00248379 |
| ensp00000336829 | fgg | 5421 | 0.559789343 | -5.572908774 | 0.00249683 |
| ensp00000312506 | cspg4 | 6090 | 0.628872367 | 5.084418886 | 0.00261399 |
| ensp00000375069 | col23a1 | 5712 | 0.58983891 | -2.40740625 | 0.00274797 |
| ensp00000408617 | hdac9 | 2967 | 0.30638166 | 9.840931637 | 0.00275596 |
| ensp00000318212 | kcnh6 | 8456 | 0.873192895 | -4.558305498 | 0.00276444 |
| ensp00000222482 | cpa4 | 24 | 0.002478315 | 3.25407915 | 0.00285372 |
| ensp00000243997 | atp5e | 8622 | 0.890334572 | -3.247026786 | 0.00296649 |
| ensp00000362566 | hpca | 6752 | 0.697232549 | 6.510376145 | 0.00298115 |
| ensp00000284562 | gsta5 | 7807 | 0.806175134 | -4.267326733 | 0.00300445 |
| ensp00000363081 | dkk1 | 3735 | 0.385687732 | 2.177283439 | 0.00303483 |
| ensp00000319343 | cpt1c | 7610 | 0.785832301 | 7.304276923 | 0.00306099 |
| ensp00000233954 | il1rl1 | 7414 | 0.76559273 | 12.99999651 | 0.00306689 |
| ensp00000341138 | epb41l3 | 3614 | 0.373192895 | 6.621118012 | 0.00309331 |
| ensp00000363512 | alox5 | 7677 | 0.792750929 | 4.410008516 | 0.00318019 |
| ensp00000360762 | ankrd1 | 3587 | 0.370404791 | 5.122907753 | 0.00328646 |
| ensp00000350332 | mybpc2 | 5914 | 0.610698059 | 8.791497358 | 0.00332724 |
| ensp00000256722 | cmpk2 | 2634 | 0.271995043 | 6.383092912 | 0.00340272 |
| ensp00000338548 | fgf1 | 5084 | 0.524989674 | 3.284856784 | 0.00340361 |
| ensp00000262345 | il12rb2 | 4529 | 0.467678645 | 4.808360694 | 0.00340984 |
| ensp00000328674 | cldn6 | 6942 | 0.71685254 | 7.301037128 | 0.00347216 |
| ensp00000255465 | ccna1 | 2422 | 0.250103263 | 2.707922906 | 0.00352968 |
| ensp00000317310 | cabp1 | 5185 | 0.535419248 | -6.916673077 | 0.0035445 |
| ensp00000239243 | msx2 | 1241 | 0.128149525 | -2.869558651 | 0.00367765 |
| ensp00000354609 | cnksr1 | 5122 | 0.528913672 | 3.305861305 | 0.00376426 |
| ensp00000264012 | cdh3 | 3799 | 0.392296572 | 4.947531915 | 0.00376706 |
| ensp00000261826 | p2rx7 | 1192 | 0.123089632 | 6.387604651 | 0.00387559 |
| ensp00000303208 | pcsk9 | 6295 | 0.650041305 | 3.779419939 | 0.00388316 |

| | | | | | |
|---|---|---|---|---|---|
| ensp00000264917 | pde8b | 3848 | 0.397356464 | -2.138188095 | 0.00399098 |
| ensp00000256759 | fst | 5875 | 0.606670797 | 4.086333062 | 0.0041566 |
| ensp00000384169 | fbln2 | 5947 | 0.614105741 | 4.18673822 | 0.00422588 |
| ensp00000350256 | ccr9 | 4030 | 0.416150351 | 8.755317825 | 0.0042585 |
| ensp00000262441 | glp2r | 3845 | 0.397046675 | -2.034159885 | 0.00427936 |
| ensp00000372170 | msx1 | 1690 | 0.174514663 | -2.549055221 | 0.00432845 |
| ensp00000225245 | ccl3 | 4075 | 0.420797191 | 5.081635246 | 0.0044796 |
| ensp00000364336 | tbxa2r | 4411 | 0.455493598 | 5.515969457 | 0.00448403 |
| ensp00000378577 | cldn3 | 6953 | 0.717988435 | 4.915029933 | 0.00449438 |
| ensp00000386069 | adra2c | 3994 | 0.412432879 | 2.357584383 | 0.00453235 |
| ensp00000267502 | rdh12 | 7723 | 0.797501033 | -5.388354746 | 0.00458433 |
| ensp00000240617 | plbd1 | 7480 | 0.772408096 | 5.221183822 | 0.00459683 |
| ensp00000252945 | cyp2e1 | 7749 | 0.800185874 | 4 | 0.00462298 |
| ensp00000219150 | coro1a | 5921 | 0.6114209 | 2.460607273 | 0.00464528 |
| ensp00000245919 | fosb | 4487 | 0.463341594 | 2.95041844 | 0.00466014 |
| ensp00000254661 | ramp1 | 3779 | 0.390231309 | 6.517368732 | 0.00466874 |
| ensp00000346901 | fmo1 | 7759 | 0.801218505 | -3.24648659 | 0.00471725 |
| ensp00000336591 | ppp2r2b | 3264 | 0.337050805 | 4.920607743 | 0.00482198 |
| ensp00000233809 | igfbp2 | 5745 | 0.593246592 | 4.080906149 | 0.00493268 |
| ensp00000379154 | rasgef1a | 6382 | 0.659025196 | 3.298980152 | 0.00493576 |
| ensp00000388373 | ppfia2 | 2914 | 0.300908715 | -2.266749347 | 0.00507743 |
| ensp00000264568 | bmpr1b | 5818 | 0.6007848 | -2.400946537 | 0.00511849 |
| ensp00000359892 | slc44a5 | 4804 | 0.496076002 | 14.08958819 | 0.0052223 |
| ensp00000371432 | prlr | 4758 | 0.491325898 | -4.990023262 | 0.00537009 |
| ensp00000330330 | tmprss2 | 1514 | 0.156340355 | 3.961846101 | 0.00544492 |
| ensp00000360626 | kcng1 | 8471 | 0.874741842 | 2.192008772 | 0.00548366 |
| ensp00000312185 | elmo1 | 4872 | 0.503097893 | 7.217391304 | 0.00552998 |
| ensp00000343318 | b3galt5 | 9225 | 0.95260223 | -9.166666667 | 0.0056483 |
| ensp00000357789 | flg | 7174 | 0.740809583 | 3.172686575 | 0.00566665 |
| ensp00000369858 | alox5ap | 7647 | 0.789653036 | 3.171370722 | 0.0057822 |
| ensp00000264839 | rims1 | 6991 | 0.721912433 | 3.015742907 | 0.00581889 |
| ensp00000358802 | kcnc4 | 8444 | 0.871953738 | 2.87344199 | 0.00590851 |
| ensp00000230361 | guca1b | 1939 | 0.200227179 | -4.090204077 | 0.00592181 |
| ensp00000295408 | mertk | 5599 | 0.578170178 | 3.137204687 | 0.0059453 |
| ensp00000369050 | cyp1a1 | 7719 | 0.79708798 | 3.924212607 | 0.00595916 |
| ensp00000321106 | tac1 | 4169 | 0.430503924 | 3.768290385 | 0.00597637 |
| ensp00000263816 | lrp2 | 5830 | 0.602023957 | -4.765757295 | 0.00609611 |
| ensp00000363079 | mbl2 | 7449 | 0.769206939 | -10.17392189 | 0.00612646 |
| ensp00000361914 | oxct2 | 8025 | 0.828686493 | 3.261347418 | 0.00618922 |
| ensp00000374372 | sptb | 4741 | 0.489570425 | 3.666669725 | 0.00621648 |
| ensp00000353154 | nfasc | 6839 | 0.706216439 | 2.323279443 | 0.00684147 |
| ensp00000360020 | diras3 | 1487 | 0.153552251 | 9.315779668 | 0.00685814 |
| ensp00000296474 | mst1r | 3615 | 0.373296159 | 3.75684117 | 0.00708146 |
| ensp00000414598 | mrvi1 | 3621 | 0.373915737 | -3.022807892 | 0.00718912 |
| ensp00000354900 | gjb1 | 9103 | 0.940004131 | -5.028484382 | 0.00719417 |
| ensp00000260598 | khk | 7322 | 0.756092524 | -2.540983607 | 0.00722526 |
| ensp00000410715 | sfrp4 | 3695 | 0.381557208 | -6.084287356 | 0.00725236 |

| | | | | | |
|---|---|---|---|---|---|
| ensp00000386209 | or2a2 | 9556 | 0.986782321 | 2 | 0.00735018 |
| ensp00000301466 | soat2 | 7821 | 0.807620818 | 2.837209302 | 0.0073978 |
| ensp00000328181 | nog | 5890 | 0.608219744 | 4.13475 | 0.00751963 |
| ensp00000393379 | kif5c | 6160 | 0.636100785 | 6.515693505 | 0.00762179 |
| ensp00000257572 | hrk | 3443 | 0.355534903 | 4.382121951 | 0.00774568 |
| ensp00000366249 | ubd | 2106 | 0.217472119 | 3.714301906 | 0.00793468 |
| ensp00000199764 | ceacam6 | 9012 | 0.930607187 | 7.245759642 | 0.00805557 |
| ensp00000324494 | grhl1 | 3431 | 0.354295746 | 4.037837838 | 0.00818461 |
| ensp00000085219 | cd22 | 4952 | 0.511358943 | -7.858325 | 0.00826404 |
| ensp00000282096 | pde3b | 2717 | 0.280565882 | 14.28 | 0.00828169 |
| ensp00000338072 | avpr2 | 3821 | 0.39456836 | 3.011224688 | 0.00832125 |
| ensp00000369531 | ubqlnl | 1520 | 0.156959934 | -2.617716237 | 0.00833804 |
| ensp00000320130 | dync1i1 | 3136 | 0.323833127 | 3.203238345 | 0.00838799 |
| ensp00000369530 | alox15b | 7676 | 0.792647666 | 2.395152474 | 0.00843067 |
| ensp00000306459 | b4galt6 | 8588 | 0.886823627 | 3.208 | 0.00845798 |
| ensp00000329454 | rcan2 | 531 | 0.054832714 | 2.145310944 | 0.00852552 |
| ensp00000337255 | kcne1 | 8484 | 0.876084263 | 4.472982752 | 0.0088586 |
| ensp00000254695 | rap1gap2 | 3795 | 0.391883519 | 3.166352353 | 0.00886671 |
| ensp00000414187 | cpeb1 | 2529 | 0.261152416 | 2.51465711 | 0.00890127 |
| ensp00000221700 | cyp4f2 | 7618 | 0.786658406 | -4.257257878 | 0.00891864 |
| ensp00000368872 | insc | 315 | 0.032527881 | 3.889868757 | 0.0089638 |
| ensp00000244333 | lypd3 | 84 | 0.008674102 | 2.05186353 | 0.00908366 |
| ensp00000387230 | upp2 | 2684 | 0.277158199 | -7.304689606 | 0.00939085 |
| ensp00000263373 | sptbn4 | 4740 | 0.489467162 | 3.515093224 | 0.0094471 |
| ensp00000234347 | prtn3 | 3451 | 0.356361008 | -2.292222128 | 0.00945511 |
| ensp00000333122 | nr4a3 | 8987 | 0.928025609 | 2.415669323 | 0.00948869 |
| ensp00000304782 | ces3 | 2738 | 0.282734407 | 2.754067073 | 0.00962081 |
| ensp00000353864 | pak3 | 4736 | 0.48905411 | 2.27728698 | 0.00970403 |
| ensp00000239849 | tnfsf11 | 6437 | 0.664704667 | 4.760421875 | 0.00984965 |
| ensp00000329210 | or2t10 | 9592 | 0.990499793 | -2.777428314 | 0.00990853 |
| ensp00000078445 | creb3l3 | 3889 | 0.401590252 | -5.285117651 | 0.0099227 |

**Significantly Changed Genes C-ADPKD vs. NC-ADPKD**

| Protein | Gene Symbol | Position | Relative Position | Fold Change | P value |
|---|---|---|---|---|---|
| ensp00000064724 | cldn11 | 6940 | 0.716646014 | 44.51178571 | 2.56E-10 |
| ensp00000342445 | cldn4 | 6958 | 0.71850475 | 2.383543642 | 3.59E-10 |
| ensp00000220772 | sfrp1 | 3693 | 0.381350682 | 29.67964047 | 6.60E-09 |
| ensp00000291009 | pip | 9186 | 0.948574969 | 17.53212618 | 7.01E-09 |
| ensp00000296370 | s100p | 108 | 0.011152416 | 13.33333333 | 7.43E-09 |
| ensp00000270279 | cblc | 4731 | 0.488537794 | 17.91788961 | 8.59E-09 |
| ensp00000260404 | pak6 | 4735 | 0.488950847 | 11.07691699 | 1.31E-08 |
| ensp00000364898 | syk | 4836 | 0.499380421 | 24.75 | 2.05E-08 |
| ensp00000296027 | cxcl5 | 4061 | 0.419351508 | 2.185981453 | 2.18E-08 |
| ensp00000229030 | fzd10 | 3730 | 0.385171417 | 19.23864686 | 2.27E-08 |
| ensp00000332643 | ndn | 3762 | 0.388475836 | 33.91955172 | 4.18E-08 |
| ensp00000364336 | tbxa2r | 4411 | 0.455493598 | 2.289182119 | 9.45E-08 |

| | | | | | |
|---|---|---|---|---|---|
| ensp00000309270 | chst1 | 9289 | 0.95921107 | -15.91204827 | 1.01E-07 |
| ensp00000338185 | plcb1 | 4457 | 0.460243701 | 8.126586393 | 1.09E-07 |
| ensp00000371471 | rsad2 | 5347 | 0.552147873 | 22.38824987 | 1.25E-07 |
| ensp00000354376 | rab25 | 7494 | 0.773853779 | 22.79829177 | 2.30E-07 |
| ensp00000263339 | il1a | 4388 | 0.453118546 | 21.37931034 | 2.95E-07 |
| ensp00000255192 | bhmt2 | 7900 | 0.815778604 | 21.19846473 | 5.11E-07 |
| ensp00000312021 | fut1 | 9240 | 0.954151177 | 3.408705236 | 5.46E-07 |
| ensp00000258499 | usp44 | 520 | 0.053696819 | 16.49109508 | 5.65E-07 |
| ensp00000369962 | igsf5 | 5212 | 0.538207352 | -5.439998176 | 7.30E-07 |
| ensp00000356505 | ncf2 | 5279 | 0.545125981 | 5.573523686 | 7.36E-07 |
| ensp00000350928 | gad1 | 7909 | 0.816707972 | 13.09152333 | 7.61E-07 |
| ensp00000167586 | krt14 | 3446 | 0.355844692 | 18.98865794 | 9.42E-07 |
| ensp00000329468 | adap2 | 6190 | 0.639198678 | 2.008806911 | 9.81E-07 |
| ensp00000358042 | qrsl1 | 68 | 0.007021892 | -2.536358531 | 9.84E-07 |
| ensp00000362171 | nap1l3 | 26 | 0.002684841 | 12.58620467 | 1.55E-06 |
| ensp00000330658 | pappa | 5744 | 0.593143329 | 2.035979733 | 1.59E-06 |
| ensp00000357040 | vangl2 | 3540 | 0.365551425 | 22.72943851 | 1.69E-06 |
| ensp00000279804 | ctf1 | 6015 | 0.621127633 | 2.069220061 | 1.69E-06 |
| ensp00000386896 | itga6 | 5563 | 0.574452705 | 2.835539697 | 1.82E-06 |
| ensp00000296849 | nkd2 | 3549 | 0.366480793 | -2.658345964 | 1.85E-06 |
| ensp00000330074 | hist1h1b | 2012 | 0.207765386 | 15.98278618 | 2.05E-06 |
| ensp00000368632 | gata3 | 3232 | 0.333746386 | 17.58931035 | 2.81E-06 |
| ensp00000369071 | postn | 7251 | 0.748760843 | 18.47654025 | 2.91E-06 |
| ensp00000259396 | orm1 | 8431 | 0.870611318 | -2.138476574 | 3.12E-06 |
| ensp00000398028 | npr3 | 8551 | 0.883002891 | -2.405876033 | 3.14E-06 |
| ensp00000348170 | hp | 8651 | 0.893329203 | -2.445684988 | 3.21E-06 |
| ensp00000321853 | serpinf2 | 5259 | 0.543060719 | -3.221041573 | 3.55E-06 |
| ensp00000377265 | tfap2b | 334 | 0.03448988 | 21.40208392 | 3.72E-06 |
| ensp00000270800 | il22ra1 | 4621 | 0.477178852 | -2.674908702 | 3.85E-06 |
| ensp00000243213 | il13ra2 | 4592 | 0.474184221 | 17.93752494 | 3.90E-06 |
| ensp00000379310 | casc1 | 1584 | 0.163568773 | -2.344579919 | 4.36E-06 |
| ensp00000358423 | rragd | 3548 | 0.36637753 | 2.234205521 | 4.49E-06 |
| ensp00000355920 | slc22a2 | 6993 | 0.722118959 | 15.60712457 | 4.70E-06 |
| ensp00000312987 | hnf4a | 3291 | 0.33983891 | -4.495562301 | 4.82E-06 |
| ensp00000296435 | camp | 603 | 0.062267658 | -12.98484091 | 4.83E-06 |
| ensp00000359410 | ephx4 | 7459 | 0.77023957 | 2.543785453 | 4.84E-06 |
| ensp00000306099 | fgb | 5420 | 0.55968608 | -2.406823649 | 5.40E-06 |
| ensp00000339292 | cldn14 | 6963 | 0.719021066 | 16.92308225 | 6.12E-06 |
| ensp00000417583 | st6galnac5 | 9234 | 0.953531599 | 11.24444444 | 6.15E-06 |
| ensp00000228938 | mgp | 5149 | 0.531701776 | 13.27536232 | 7.02E-06 |
| ensp00000295550 | col6a3 | 5688 | 0.587360595 | 5.25159387 | 7.51E-06 |
| ensp00000250448 | foxa1 | 5483 | 0.566191656 | 4.321700959 | 8.74E-06 |
| ensp00000343318 | b3galt5 | 9225 | 0.95260223 | -9.211116667 | 8.76E-06 |
| ensp00000347379 | ocln | 6965 | 0.719227592 | 3.548515545 | 9.36E-06 |
| ensp00000306361 | fga | 5419 | 0.559582817 | -13.13548185 | 9.49E-06 |
| ensp00000291294 | ptgir | 3853 | 0.39787278 | 5.46636568 | 9.78E-06 |
| ensp00000255189 | dmgdh | 8162 | 0.84283354 | 10.00514948 | 9.82E-06 |

| | | | | | |
|---|---|---|---|---|---|
| ensp00000245312 | slc10a2 | 8413 | 0.868752582 | -3.27800551 | 1.00E-05 |
| ensp00000264260 | il18rap | 4285 | 0.442482445 | -14.10436879 | 1.00E-05 |
| ensp00000301732 | abca3 | 1120 | 0.115654688 | 2.602772021 | 1.08E-05 |
| ensp00000282111 | tcf7l1 | 3411 | 0.352230483 | 5.294101257 | 1.26E-05 |
| ensp00000303153 | col22a1 | 5701 | 0.588703015 | 14.21212121 | 1.38E-05 |
| ensp00000335083 | ppp2r2c | 3265 | 0.337154069 | 13.4959812 | 1.40E-05 |
| ensp00000293272 | ccl5 | 4058 | 0.419041718 | 5.427583054 | 1.46E-05 |
| ensp00000357789 | flg | 7174 | 0.740809583 | 3.458573177 | 1.49E-05 |
| ensp00000298472 | slc18a2 | 7030 | 0.725939694 | 12.08618786 | 1.54E-05 |
| ensp00000383558 | gcgr | 4125 | 0.425960347 | 15.66943444 | 1.87E-05 |
| ensp00000363079 | mbl2 | 7449 | 0.769206939 | -11.27826198 | 1.92E-05 |
| ensp00000344874 | gucy1a2 | 2353 | 0.242978108 | -2.812584289 | 2.02E-05 |
| ensp00000406367 | gpr124 | 6290 | 0.64952499 | 2.048008152 | 2.05E-05 |
| ensp00000260197 | sorl1 | 8269 | 0.853882693 | 18.16936949 | 2.12E-05 |
| ensp00000309148 | klk6 | 7438 | 0.768071045 | 5.281660553 | 2.21E-05 |
| ensp00000226524 | pf4v1 | 3952 | 0.408095828 | 10.77097651 | 2.28E-05 |
| ensp00000321735 | slc16a8 | 7312 | 0.755059893 | 10.12357289 | 2.32E-05 |
| ensp00000284440 | uchl1 | 3180 | 0.328376704 | 3.498111152 | 2.45E-05 |
| ensp00000312397 | klhl3 | 2138 | 0.220776539 | 14.39551165 | 2.67E-05 |
| ensp00000352272 | myoz1 | 6213 | 0.64157373 | 12.20213 | 2.71E-05 |
| ensp00000252971 | mnx1 | 9381 | 0.968711276 | 20.04543632 | 2.93E-05 |
| ensp00000306157 | il7r | 4596 | 0.474597274 | 7.607705852 | 2.93E-05 |
| ensp00000228850 | akap3 | 8638 | 0.891986782 | 8.813735294 | 3.09E-05 |
| ensp00000302648 | nrtn | 6002 | 0.619785213 | -4.096161723 | 3.23E-05 |
| ensp00000253513 | ido1 | 7944 | 0.820322181 | 11.53880822 | 3.27E-05 |
| ensp00000261769 | cdh1 | 3594 | 0.371127633 | 2.39973029 | 3.31E-05 |
| ensp00000266085 | timp3 | 6030 | 0.62267658 | 27.15913314 | 3.40E-05 |
| ensp00000354207 | ntrk3 | 4738 | 0.489260636 | -3.690931034 | 3.41E-05 |
| ensp00000263182 | bbox1 | 8124 | 0.838909542 | -16.54309781 | 3.53E-05 |
| ensp00000363157 | tnfsf15 | 6765 | 0.698574969 | 16.5454486 | 3.70E-05 |
| ensp00000288221 | erc2 | 7029 | 0.725836431 | 3.912391026 | 3.74E-05 |
| ensp00000245912 | tnfsf14 | 6700 | 0.691862867 | -3.779492308 | 4.00E-05 |
| ensp00000262262 | cd33 | 8714 | 0.899834779 | -23.50946557 | 4.35E-05 |
| ensp00000283921 | hoxa10 | 137 | 0.014147047 | 9.357671298 | 4.64E-05 |
| ensp00000420419 | jam2 | 5213 | 0.538310615 | 3.130434783 | 4.65E-05 |
| ensp00000289422 | nrg2 | 4649 | 0.480070219 | 8.783217634 | 4.66E-05 |
| ensp00000394033 | kcnk2 | 9358 | 0.966336225 | -3.576407304 | 4.74E-05 |
| ensp00000316328 | ciita | 5324 | 0.549772821 | -10.40362675 | 4.94E-05 |
| ensp00000172229 | ngfr | 4432 | 0.457662123 | -10.84090293 | 5.03E-05 |
| ensp00000310721 | cyp7b1 | 7843 | 0.809892606 | -2.123720859 | 5.17E-05 |
| ensp00000282728 | hhex | 9383 | 0.968917803 | 12.79431915 | 5.25E-05 |
| ensp00000260795 | fgfr3 | 4877 | 0.503614209 | -2.477534897 | 5.33E-05 |
| ensp00000254695 | rap1gap2 | 3795 | 0.391883519 | 2.168210373 | 5.56E-05 |
| ensp00000303208 | pcsk9 | 6295 | 0.650041305 | 6.908596774 | 5.64E-05 |
| ensp00000353362 | cacna1a | 4242 | 0.438042131 | 10.87193849 | 5.65E-05 |
| ensp00000287020 | gdf6 | 5925 | 0.611833953 | 3.737120456 | 5.69E-05 |
| ensp00000308576 | rhod | 5007 | 0.517038414 | 13.2041868 | 5.87E-05 |

| | | | | | |
|---|---|---|---|---|---|
| ensp00000296474 | mst1r | 3615 | 0.373296159 | 3.279520179 | 5.92E-05 |
| ensp00000272134 | lefty1 | 5813 | 0.600268484 | -6.222710141 | 6.77E-05 |
| ensp00000254508 | nup210 | 1338 | 0.138166047 | 32 | 6.99E-05 |
| ensp00000257818 | lmo2 | 7139 | 0.737195374 | 21.93407905 | 7.07E-05 |
| ensp00000173229 | ntn1 | 4856 | 0.501445684 | 19.30862963 | 7.07E-05 |
| ensp00000396774 | muc20 | 9268 | 0.957042544 | 7.027908359 | 7.16E-05 |
| ensp00000299106 | jam3 | 5214 | 0.538413879 | 15.5651896 | 7.21E-05 |
| ensp00000003084 | cftr | 3256 | 0.336224701 | 12.03672978 | 7.33E-05 |
| ensp00000346693 | elovl2 | 7559 | 0.780565882 | 2.627751269 | 7.53E-05 |
| ensp00000351682 | cndp1 | 7968 | 0.822800496 | -9.05347543 | 7.93E-05 |
| ensp00000292513 | ptger1 | 4210 | 0.434737712 | 15.8364717 | 8.22E-05 |
| ensp00000284240 | thy1 | 5422 | 0.559892606 | 2.811004464 | 8.67E-05 |
| ensp00000419361 | adcy5 | 3931 | 0.405927303 | 4.247963415 | 8.77E-05 |
| ensp00000308032 | cyp2s1 | 7800 | 0.805452292 | 5.656962305 | 9.28E-05 |
| ensp00000410668 | tnf | 4314 | 0.445477076 | 8.85 | 9.34E-05 |
| ensp00000392858 | tnf | 4353 | 0.449504337 | 8.85 | 9.34E-05 |
| ensp00000398698 | tnf | 4354 | 0.4496076 | 8.85 | 9.34E-05 |
| ensp00000365290 | tnf | 6348 | 0.65551425 | 8.85 | 9.34E-05 |
| ensp00000338171 | skap1 | 6173 | 0.637443205 | 2.576923077 | 9.34E-05 |
| ensp00000367830 | prkcz | 4462 | 0.460760017 | 2.335155393 | 9.91E-05 |
| ensp00000277480 | lcn2 | 7529 | 0.777467988 | 3.161949153 | 0.000107292 |
| ensp00000291539 | pde9a | 2744 | 0.283353986 | 7.755728846 | 0.000114027 |
| ensp00000386069 | adra2c | 3994 | 0.412432879 | 2.460959935 | 0.000114964 |
| ensp00000330959 | il1r2 | 4391 | 0.453428335 | -2.007916865 | 0.000117318 |
| ensp00000254262 | c19orf40 | 2132 | 0.22015696 | -2.521800719 | 0.000118643 |
| ensp00000308461 | rnd1 | 5027 | 0.519103676 | 3.513906282 | 0.000118796 |
| ensp00000320378 | slc7a8 | 8668 | 0.895084676 | 12.88549398 | 0.000122585 |
| ensp00000342114 | icam4 | 5495 | 0.567430814 | 4.697574719 | 0.000122653 |
| ensp00000290399 | sim2 | 418 | 0.043163982 | 14.98305507 | 0.000127176 |
| ensp00000342235 | erbb4 | 4694 | 0.484717059 | 14.45713967 | 0.000138045 |
| ensp00000264257 | il1rl2 | 8757 | 0.904275093 | 4.672275072 | 0.000139219 |
| ensp00000296414 | dapp1 | 4797 | 0.49535316 | 15.68 | 0.000140646 |
| ensp00000350332 | mybpc2 | 5914 | 0.610698059 | 9.26458263 | 0.000141893 |
| ensp00000351190 | itih2 | 6867 | 0.709107807 | -9.481288493 | 0.000145183 |
| ensp00000264708 | pomc | 3865 | 0.399111937 | 11.11223356 | 0.000145725 |
| ensp00000327453 | acsm2b | 8508 | 0.878562577 | -2.966131424 | 0.000149728 |
| ensp00000287641 | sst | 3946 | 0.407476249 | 3.671968254 | 0.00015939 |
| ensp00000279441 | mmp10 | 5504 | 0.568360182 | 13.07334 | 0.00016102 |
| ensp00000368966 | trpc3 | 5123 | 0.529016935 | -3.109954129 | 0.000168462 |
| ensp00000296641 | f2rl2 | 4414 | 0.455803387 | 10.0152519 | 0.000170865 |
| ensp00000354490 | atp1a2 | 9053 | 0.934840975 | -36.35294118 | 0.000171798 |
| ensp00000263126 | akr1c4 | 7756 | 0.800908715 | -3.033589147 | 0.000181995 |
| ensp00000319343 | cpt1c | 7610 | 0.785832301 | 8.461386748 | 0.000186937 |
| ensp00000358309 | epha7 | 4809 | 0.496592317 | 8.511904762 | 0.000187425 |
| ensp00000340200 | glyat | 9084 | 0.938042131 | -6.418103448 | 0.000191124 |
| ensp00000297450 | angpt1 | 4717 | 0.487092111 | -3.552769539 | 0.000195147 |
| ensp00000332116 | pde4b | 2714 | 0.280256093 | 6.485298886 | 0.000199562 |

| | | | | | |
|---|---|---|---|---|---|
| ensp00000357461 | chrnb2 | 8374 | 0.86472532 | 3.584358025 | 0.000211202 |
| ensp00000199764 | ceacam6 | 9012 | 0.930607187 | 9.000004737 | 0.000212397 |
| ensp00000359074 | l1cam | 5446 | 0.562370921 | 11.67886666 | 0.000216961 |
| ensp00000287713 | nmnat2 | 2775 | 0.286555143 | 2.647398844 | 0.000221315 |
| ensp00000348888 | pigr | 5184 | 0.535315985 | 13.99997108 | 0.000224224 |
| ensp00000373024 | c6orf15 | 6334 | 0.654068567 | 9.145639947 | 0.000235536 |
| ensp00000234371 | kiss1r | 4274 | 0.441346551 | 6.694612758 | 0.000244109 |
| ensp00000288135 | kit | 4683 | 0.483581165 | 8.115700922 | 0.000246724 |
| ensp00000253754 | pdlim4 | 6838 | 0.706113176 | 7.686134192 | 0.000247255 |
| ensp00000410294 | fgfr2 | 4791 | 0.494733581 | -2.570081272 | 0.000251745 |
| ensp00000360918 | ch25h | 7817 | 0.807207765 | 15.88761136 | 0.000256918 |
| ensp00000258411 | wnt10a | 3720 | 0.384138786 | 3.310710407 | 0.000261545 |
| ensp00000355884 | mark1 | 2992 | 0.308963238 | 9.770276872 | 0.000264497 |
| ensp00000381654 | hmgcll1 | 8205 | 0.847273854 | 5.122513326 | 0.000264558 |
| ensp00000341138 | epb41l3 | 3614 | 0.373192895 | 5.835755774 | 0.000266616 |
| ensp00000294339 | tal1 | 3153 | 0.3255886 | 7.06122299 | 0.000275796 |
| ensp00000363512 | alox5 | 7677 | 0.792750929 | 2.007598811 | 0.000282394 |
| ensp00000271636 | cgn | 5144 | 0.531185461 | 3.475094137 | 0.000294001 |
| ensp00000258443 | edar | 113 | 0.011668732 | 4.466666667 | 0.000294604 |
| ensp00000249750 | aldh1a2 | 7729 | 0.798120611 | 3.15566782 | 0.000303209 |
| ensp00000354822 | xaf1 | 5363 | 0.553800083 | 6.23311232 | 0.000305488 |
| ensp00000329797 | cadm1 | 9395 | 0.97015696 | 2.126596943 | 0.000307571 |
| ensp00000306888 | fam151a | 7592 | 0.783973565 | -6.929828947 | 0.000307596 |
| ensp00000351206 | txlnb | 7377 | 0.761771995 | 15.22315308 | 0.00032163 |
| ensp00000156626 | st6galnac1 | 9320 | 0.962412226 | 10.27161111 | 0.000332787 |
| ensp00000420295 | pde6b | 2724 | 0.281288724 | 16.49314624 | 0.000334515 |
| ensp00000367462 | olah | 7458 | 0.770136307 | 5.663043478 | 0.000343653 |
| ensp00000365943 | pcsk5 | 6258 | 0.64622057 | 2.600439038 | 0.000345547 |
| ensp00000308012 | pabpc5 | 258 | 0.026641884 | 22.72727273 | 0.000345593 |
| ensp00000274353 | bhmt | 7901 | 0.815881867 | 3.387465458 | 0.000356248 |
| ensp00000362566 | hpca | 6752 | 0.697232549 | 10.1883183 | 0.000370248 |
| ensp00000254661 | ramp1 | 3779 | 0.390231309 | 3.833335484 | 0.000380896 |
| ensp00000340191 | fpr2 | 4131 | 0.426579926 | -5.257773333 | 0.000393429 |
| ensp00000364140 | col15a1 | 5711 | 0.589735646 | -3.522905925 | 0.000399961 |
| ensp00000225964 | col1a1 | 5662 | 0.584675754 | 5.696078431 | 0.000410597 |
| ensp00000261523 | rora | 3320 | 0.34283354 | 2.038628588 | 0.00042725 |
| ensp00000263621 | elane | 5414 | 0.559066501 | -5.04023517 | 0.00043389 |
| ensp00000226317 | cxcl6 | 4022 | 0.415324246 | 8.296319444 | 0.000444926 |
| ensp00000261007 | chrna1 | 7584 | 0.78314746 | -6.845712483 | 0.000456533 |
| ensp00000085219 | cd22 | 4952 | 0.511358943 | -9.583325 | 0.000458843 |
| ensp00000234071 | proc | 5498 | 0.567740603 | 2.342576916 | 0.000465124 |
| ensp00000416387 | fblim1 | 5545 | 0.572593969 | 5.8275 | 0.000470826 |
| ensp00000350616 | ddc | 7945 | 0.820425444 | -10.25757576 | 0.000484651 |
| ensp00000338072 | avpr2 | 3821 | 0.39456836 | 4.91742435 | 0.00048667 |
| ensp00000282499 | gria4 | 6747 | 0.696716233 | 4.125757862 | 0.000491604 |
| ensp00000364475 | fbp1 | 7280 | 0.751755473 | 14.2762 | 0.000508197 |
| ensp00000307046 | sdc2 | 5650 | 0.583436596 | -2.918913284 | 0.000514775 |

| | | | | | |
|---|---|---|---|---|---|
| ensp00000297268 | col1a2 | 5661 | 0.584572491 | 4.697925758 | 0.00052293 |
| ensp00000332052 | pcsk6 | 7440 | 0.768277571 | -4.892014085 | 0.000528441 |
| ensp00000352547 | gpat2 | 7406 | 0.764766625 | 2.261837253 | 0.000529003 |
| ensp00000409605 | trim15 | 6426 | 0.663568773 | -6.174825175 | 0.000545512 |
| ensp00000403221 | trim15 | 6770 | 0.699091285 | -6.174825175 | 0.000545512 |
| ensp00000297991 | aqp3 | 5333 | 0.550702189 | -3.222395023 | 0.000548472 |
| ensp00000306884 | cxcl11 | 4087 | 0.422036349 | 8.531914894 | 0.000554234 |
| ensp00000285018 | wnt7a | 3757 | 0.387959521 | 11.55803055 | 0.00056281 |
| ensp00000363965 | alpl | 6312 | 0.651796778 | -2.19113986 | 0.000568676 |
| ensp00000320886 | mlxipl | 6862 | 0.708591491 | -5.20325 | 0.000575481 |
| ensp00000227752 | il10ra | 4641 | 0.479244114 | 7.012446893 | 0.000581354 |
| ensp00000264917 | pde8b | 3848 | 0.397356464 | -2.947228776 | 0.000584285 |
| ensp00000320935 | slc2a4 | 3712 | 0.383312681 | -8.401746027 | 0.000588367 |
| ensp00000322390 | fgf13 | 5044 | 0.520859149 | 5.444441667 | 0.000610457 |
| ensp00000363298 | bspry | 7345 | 0.758467575 | 13.62927006 | 0.000611927 |
| ensp00000309572 | tert | 2947 | 0.304316398 | -2.583397779 | 0.000615036 |
| ensp00000361943 | heyl | 3216 | 0.332094176 | 5.164573306 | 0.000631158 |
| ensp00000362570 | fndc5 | 6540 | 0.675340768 | -4.185714286 | 0.000645738 |
| ensp00000361214 | nrg3 | 9183 | 0.94826518 | 15.4 | 0.000646211 |
| ensp00000414330 | rimkla | 6440 | 0.665014457 | 14.04055678 | 0.000648366 |
| ensp00000330330 | tmprss2 | 1514 | 0.156340355 | 2.717753535 | 0.000654348 |
| ensp00000351671 | ccl20 | 4060 | 0.419248245 | 3.086742858 | 0.000674984 |
| ensp00000343477 | runx3 | 3664 | 0.378356051 | -10.34920635 | 0.000681859 |
| ensp00000289746 | cdh15 | 4579 | 0.472841801 | -2.94631621 | 0.000711769 |
| ensp00000393847 | pla2g10 | 7579 | 0.782631144 | 4.474577788 | 0.000741751 |
| ensp00000209728 | cdc6 | 2374 | 0.245146634 | -2.776470588 | 0.000742506 |
| ensp00000340937 | col17a1 | 5697 | 0.588289963 | 2.020384539 | 0.000754216 |
| ensp00000309757 | lpl | 5103 | 0.526951673 | 8.448644082 | 0.000758161 |
| ensp00000255082 | acy3 | 7956 | 0.821561338 | -4.985809619 | 0.000781381 |
| ensp00000259206 | il1rn | 8756 | 0.90417183 | 3.684653968 | 0.000783651 |
| ensp00000304767 | p2ry1 | 4253 | 0.439178026 | -4.042606776 | 0.000787137 |
| ensp00000335500 | c7orf57 | 6285 | 0.649008674 | -14.03368086 | 0.000813442 |
| ensp00000302269 | vav1 | 4831 | 0.498864106 | 8.801927536 | 0.000815719 |
| ensp00000263925 | lnx1 | 6800 | 0.702189178 | 3.3358 | 0.000822816 |
| ensp00000339398 | hla-dqa1 | 5722 | 0.590871541 | -3.501084967 | 0.000827537 |
| ensp00000414360 | hla-dqa1 | 6523 | 0.673585295 | -3.501084967 | 0.000827537 |
| ensp00000387892 | hla-dqa1 | 8745 | 0.903035936 | -3.501084967 | 0.000827537 |
| ensp00000409127 | hla-dqa1 | 8746 | 0.903139199 | -3.501084967 | 0.000827537 |
| ensp00000372738 | hla-dqa1 | 8885 | 0.917492772 | -3.501084967 | 0.000827537 |
| ensp00000312326 | aoc3 | 7885 | 0.814229657 | -2.213190813 | 0.000828752 |
| ensp00000363081 | dkk1 | 3735 | 0.385687732 | 3.318771845 | 0.000834814 |
| ensp00000307694 | kcns1 | 8449 | 0.872470054 | 6.552775 | 0.000842488 |
| ensp00000005180 | ccl26 | 4080 | 0.421313507 | -2.561272059 | 0.000860035 |
| ensp00000357013 | cd244 | 5995 | 0.619062371 | 6.458066801 | 0.000863736 |
| ensp00000417229 | eif2a | 1283 | 0.132486576 | -2.491023563 | 0.000868779 |
| ensp00000359478 | abcc2 | 411 | 0.04244114 | -2.10425179 | 0.00088582 |
| ensp00000295156 | vsnl1 | 8115 | 0.837980173 | -2.48561279 | 0.000892839 |

| | | | | | |
|---|---|---|---|---|---|
| ensp00000368401 | pax6 | 6292 | 0.649731516 | 4.61732852 | 0.000900372 |
| ensp00000161559 | ceacam1 | 8612 | 0.889301941 | 2.807851737 | 0.00093237 |
| ensp00000301908 | pnoc | 4009 | 0.413981826 | 9.244701324 | 0.000949483 |
| ensp00000233809 | igfbp2 | 5745 | 0.593246592 | 3.665697674 | 0.000958719 |
| ensp00000155840 | kcnq1 | 8273 | 0.854295746 | 6.639047553 | 0.000960882 |
| ensp00000311997 | nefh | 5803 | 0.599235853 | 3.680234867 | 0.000985835 |
| ensp00000357753 | ivl | 3908 | 0.403552251 | 4.828663551 | 0.000988758 |
| ensp00000355370 | cntf | 4610 | 0.476042957 | -2.140802817 | 0.000990514 |
| ensp00000302707 | fpr1 | 4013 | 0.414394878 | -4.429771308 | 0.000999422 |
| ensp00000200307 | ccl7 | 4077 | 0.421003717 | 4.380001752 | 0.00100403 |
| ensp00000337383 | nlrp3 | 2741 | 0.283044197 | 13.19746997 | 0.00102865 |
| ensp00000275815 | epha1 | 4813 | 0.49700537 | 6.686746988 | 0.00103728 |
| ensp00000381876 | daam2 | 3597 | 0.371437423 | -2.38965928 | 0.00106433 |
| ensp00000264012 | cdh3 | 3799 | 0.392296572 | 9.965714384 | 0.00107132 |
| ensp00000360181 | sh2d1a | 6174 | 0.637546468 | 4.188782749 | 0.00108245 |
| ensp00000292596 | ltc4s | 7648 | 0.789756299 | 3.639490643 | 0.00108253 |
| ensp00000265643 | gal | 4036 | 0.41676993 | 3.235426017 | 0.00108592 |
| ensp00000357153 | cd1d | 6277 | 0.648182569 | 3.054218707 | 0.00110678 |
| ensp00000385149 | neu4 | 8139 | 0.840458488 | -4.995047027 | 0.00112194 |
| ensp00000217407 | lbp | 4368 | 0.451053284 | -3.031703216 | 0.00116372 |
| ensp00000276925 | cdkn2b | 2759 | 0.284902933 | 4.549023529 | 0.00116468 |
| ensp00000262752 | rps6ka6 | 4454 | 0.459933912 | 6.018513889 | 0.00118815 |
| ensp00000298841 | serpina4 | 9340 | 0.964477489 | -3.019191895 | 0.00119611 |
| ensp00000367714 | hes5 | 3276 | 0.338289963 | 13.04053643 | 0.00122215 |
| ensp00000363985 | mtmr8 | 9114 | 0.941140025 | 3.568707469 | 0.00122751 |
| ensp00000384264 | cnga1 | 3898 | 0.40251962 | 5.824324324 | 0.00123806 |
| ensp00000268933 | epn3 | 4975 | 0.513733994 | 4.101400035 | 0.00123978 |
| ensp00000259455 | gabbr2 | 4239 | 0.437732342 | 12.87340957 | 0.00124122 |
| ensp00000299339 | cldn10 | 6957 | 0.718401487 | 13.19885046 | 0.00124638 |
| ensp00000304408 | col3a1 | 5660 | 0.584469228 | 20.2999394 | 0.00125116 |
| ensp00000275016 | cyp39a1 | 7815 | 0.807001239 | 2.864525369 | 0.00126559 |
| ensp00000354478 | dlx1 | 1325 | 0.136823627 | 2.394528279 | 0.00134141 |
| ensp00000312837 | rab11fip4 | 7504 | 0.774886411 | 3.116567663 | 0.00137797 |
| ensp00000374372 | sptb | 4741 | 0.489570425 | 2.572969041 | 0.00141025 |
| ensp00000310244 | rasgrp1 | 5067 | 0.523234201 | 7.773282499 | 0.00141894 |
| ensp00000263413 | c6 | 2525 | 0.260739364 | -9.658811965 | 0.00142211 |
| ensp00000356905 | vnn1 | 8380 | 0.865344899 | 4.504672897 | 0.0014259 |
| ensp00000356991 | pvrl4 | 5595 | 0.577757125 | 5.414354167 | 0.00145337 |
| ensp00000221700 | cyp4f2 | 7618 | 0.786658406 | -6.302907632 | 0.00147472 |
| ensp00000359892 | slc44a5 | 4804 | 0.496076002 | 9.165067151 | 0.00150483 |
| ensp00000289429 | cd1a | 7337 | 0.75764147 | 3.252790612 | 0.00162062 |
| ensp00000384169 | fbln2 | 5947 | 0.614105741 | 2.176953881 | 0.00162401 |
| ensp00000367059 | espn | 6547 | 0.67606361 | -2.567453911 | 0.00162452 |
| ensp00000360806 | kcnb1 | 8389 | 0.866274267 | -4.605405405 | 0.00164498 |
| ensp00000260187 | usp2 | 2982 | 0.307930607 | -2.331424765 | 0.00166925 |
| ensp00000337731 | cideb | 4153 | 0.428851714 | -2.44824173 | 0.00167489 |
| ensp00000216115 | bik | 3463 | 0.357600165 | 2.402587875 | 0.00169197 |

| | | | | | |
|---|---|---|---|---|---|
| ensp00000255266 | pde6a | 2725 | 0.281391987 | -9.552795309 | 0.00169751 |
| ensp00000201031 | tfap2c | 335 | 0.034593143 | -3.179699588 | 0.00178998 |
| ensp00000355245 | pax9 | 625 | 0.064539447 | -2.438453981 | 0.00182432 |
| ensp00000353198 | pyy | 3951 | 0.407992565 | 7.587172098 | 0.00182649 |
| ensp00000297404 | kcnv1 | 8472 | 0.874845105 | -4.516933898 | 0.0018502 |
| ensp00000319814 | pck1 | 3713 | 0.383415944 | -9.602272727 | 0.00187759 |
| ensp00000336591 | ppp2r2b | 3264 | 0.337050805 | 3.079187765 | 0.0018851 |
| ensp00000291670 | ftcd | 8260 | 0.852953325 | 5.157452354 | 0.00188577 |
| ensp00000410321 | ly6g5c | 6509 | 0.672139612 | -2.493239874 | 0.00188894 |
| ensp00000386209 | or2a2 | 9556 | 0.986782321 | 3.924814981 | 0.00195691 |
| ensp00000304004 | foxa3 | 5485 | 0.566398183 | 2.061748587 | 0.00207326 |
| ensp00000369858 | alox5ap | 7647 | 0.789653036 | 2.104338927 | 0.0021103 |
| ensp00000265728 | dbf4 | 2326 | 0.240190004 | -3.600010141 | 0.00220018 |
| ensp00000305424 | or1f1 | 9666 | 0.998141264 | -4.536315901 | 0.00223536 |
| ensp00000408617 | hdac9 | 2967 | 0.30638166 | 8.927854565 | 0.00225298 |
| ensp00000305603 | fut3 | 9242 | 0.954357703 | 8.874999598 | 0.00226943 |
| ensp00000261233 | irak3 | 4345 | 0.448678232 | 9.993945455 | 0.00236812 |
| ensp00000358092 | prdm1 | 1754 | 0.181123503 | 2.363633481 | 0.00237627 |
| ensp00000333122 | nr4a3 | 8987 | 0.928025609 | 2.727139021 | 0.00238737 |
| ensp00000249016 | mchr1 | 4104 | 0.423791822 | 9.072081081 | 0.00242392 |
| ensp00000299333 | scn3b | 286 | 0.029533251 | 10.4367931 | 0.00245294 |
| ensp00000222792 | chn2 | 7872 | 0.812887237 | 5.144111417 | 0.00245543 |
| ensp00000305355 | prkcb | 5216 | 0.538620405 | -3.356397188 | 0.00245884 |
| ensp00000278187 | gas2 | 6702 | 0.692069393 | -2.803355237 | 0.00256702 |
| ensp00000345512 | sema6a | 6293 | 0.649834779 | 3.874014223 | 0.00260485 |
| ensp00000372160 | dok6 | 4726 | 0.488021479 | 9.144177142 | 0.00268846 |
| ensp00000346160 | dusp19 | 7537 | 0.778294093 | -3.375857457 | 0.00269066 |
| ensp00000277120 | ntrk2 | 4614 | 0.47645601 | 2.418577414 | 0.0027134 |
| ensp00000290341 | igf2bp1 | 1945 | 0.200846758 | 2.035827103 | 0.00274454 |
| ensp00000356898 | ddr2 | 7680 | 0.793060719 | 2.821721945 | 0.00277948 |
| ensp00000360762 | ankrd1 | 3587 | 0.370404791 | 8.733342857 | 0.00279899 |
| ensp00000336829 | fgg | 5421 | 0.559789343 | -5.823418529 | 0.00306269 |
| ensp00000358301 | adrb1 | 3819 | 0.394361834 | 5.547012821 | 0.00311097 |
| ensp00000328181 | nog | 5890 | 0.608219744 | 2.929650665 | 0.00321233 |
| ensp00000245479 | sox9 | 1758 | 0.181536555 | 4.328553036 | 0.00326749 |
| ensp00000349595 | atp2a1 | 6095 | 0.629388682 | -3.268860162 | 0.00326965 |
| ensp00000377303 | renbp | 8856 | 0.914498141 | 4.088888889 | 0.00330581 |
| ensp00000307875 | b3gat1 | 6178 | 0.637959521 | 12.15237143 | 0.00332778 |
| ensp00000233954 | il1rl1 | 7414 | 0.76559273 | 12.02151613 | 0.0033761 |
| ensp00000410443 | hla-dra | 5721 | 0.590768278 | -3.314692788 | 0.003499 |
| ensp00000372608 | hla-dra | 5724 | 0.591078067 | -3.314692788 | 0.003499 |
| ensp00000372746 | hla-dra | 6381 | 0.658921933 | -3.314692788 | 0.003499 |
| ensp00000402951 | hla-dra | 6501 | 0.671313507 | -3.314692788 | 0.003499 |
| ensp00000405295 | hla-dra | 9175 | 0.947439075 | -3.314692788 | 0.003499 |
| ensp00000257549 | sds | 8150 | 0.841594382 | -2.125836443 | 0.00350846 |
| ensp00000420716 | c1orf228 | 6898 | 0.712308963 | 2.22689618 | 0.00354317 |
| ensp00000327251 | nos2 | 5053 | 0.521788517 | 2.065224055 | 0.00357044 |

| | | | | | |
|---|---|---|---|---|---|
| ensp00000225245 | ccl3 | 4075 | 0.420797191 | 5.107692308 | 0.00369454 |
| ensp00000261374 | hs3st2 | 6068 | 0.626600578 | 2.904637342 | 0.00370175 |
| ensp00000285393 | atp6v0d2 | 8558 | 0.883725733 | 3.093306394 | 0.00373616 |
| ensp00000354677 | gpx7 | 6651 | 0.686802974 | 7.303577608 | 0.00374474 |
| ensp00000263923 | kdr | 4692 | 0.484510533 | -2.500995051 | 0.00378614 |
| ensp00000225474 | csf3 | 7114 | 0.734613796 | 8.292403509 | 0.0038637 |
| ensp00000305464 | apln | 4035 | 0.416666667 | 2.484323143 | 0.00386891 |
| ensp00000216274 | ripk3 | 4326 | 0.446716233 | 6.324013986 | 0.00393956 |
| ensp00000328968 | scn5a | 3606 | 0.372366791 | 3.240819085 | 0.0039674 |
| ensp00000252945 | cyp2e1 | 7749 | 0.800185874 | 6.293701893 | 0.00402717 |
| ensp00000263686 | selp | 6078 | 0.627633209 | 5.984783891 | 0.00432483 |
| ensp00000357905 | dmbt1 | 264 | 0.027261462 | -2.631569391 | 0.00435826 |
| ensp00000331504 | fes | 4659 | 0.48110285 | 8.823301194 | 0.0043813 |
| ensp00000387230 | upp2 | 2684 | 0.277158199 | -4.242184186 | 0.00440393 |
| ensp00000363680 | eda | 114 | 0.011771995 | 4.956952008 | 0.00448789 |
| ensp00000339992 | myb | 3487 | 0.36007848 | -2.274347367 | 0.0045276 |
| ensp00000304930 | sostdc1 | 5873 | 0.606464271 | -3.527680269 | 0.00461872 |
| ensp00000255380 | chrm3 | 4187 | 0.43236266 | 2.564606309 | 0.00464241 |
| ensp00000375069 | col23a1 | 5712 | 0.58983891 | -2.449072917 | 0.00464399 |
| ensp00000378577 | cldn3 | 6953 | 0.717988435 | 8.244995612 | 0.00469634 |
| ensp00000263816 | lrp2 | 5830 | 0.602023957 | -4.817455279 | 0.00485124 |
| ensp00000256722 | cmpk2 | 2634 | 0.271995043 | 4.182967696 | 0.00497197 |
| ensp00000328674 | cldn6 | 6942 | 0.71685254 | 6.59372627 | 0.00497697 |
| ensp00000312506 | cspg4 | 6090 | 0.628872367 | 3.764424887 | 0.00498414 |
| ensp00000295408 | mertk | 5599 | 0.578170178 | 2.432624113 | 0.00503116 |
| ensp00000219150 | coro1a | 5921 | 0.6114209 | 2.073547573 | 0.00505603 |
| ensp00000327107 | pdzd3 | 532 | 0.054935977 | -2.835643912 | 0.00506406 |
| ensp00000372170 | msx1 | 1690 | 0.174514663 | -2.068887121 | 0.00525792 |
| ensp00000354416 | ccl28 | 3984 | 0.411400248 | 3.699994906 | 0.0053093 |
| ensp00000360561 | entpd8 | 2616 | 0.270136307 | -5.036453125 | 0.00537546 |
| ensp00000379204 | bmp7 | 5815 | 0.60047501 | 12.17046838 | 0.00538245 |
| ensp00000259365 | tmod1 | 5808 | 0.599752169 | 3.627913467 | 0.00552092 |
| ensp00000262441 | glp2r | 3845 | 0.397046675 | -2.45807227 | 0.00552915 |
| ensp00000350256 | ccr9 | 4030 | 0.416150351 | 10.68828482 | 0.00553522 |
| ensp00000284562 | gsta5 | 7807 | 0.806175134 | -3.655113861 | 0.00556951 |
| ensp00000393379 | kif5c | 6160 | 0.636100785 | 3.418813132 | 0.00557111 |
| ensp00000349490 | mfng | 3315 | 0.342317224 | -2.21000321 | 0.00565854 |
| ensp00000240617 | plbd1 | 7480 | 0.772408096 | 5.751281095 | 0.00597988 |
| ensp00000288139 | cacna1d | 5174 | 0.534283354 | -2.900814747 | 0.00603223 |
| ensp00000369003 | trpc4 | 5124 | 0.529120198 | 2.297961833 | 0.00609968 |
| ensp00000379895 | gatm | 8049 | 0.831164808 | 3.683649682 | 0.00618313 |
| ensp00000340396 | gbp5 | 6568 | 0.678232135 | 3.555827924 | 0.00633805 |
| ensp00000296029 | pf4 | 4336 | 0.447748864 | 3.115830116 | 0.00639547 |
| ensp00000355718 | dll1 | 3480 | 0.359355638 | 4.486208798 | 0.0065011 |
| ensp00000369323 | npnt | 6080 | 0.627839736 | 5.242091045 | 0.00652353 |
| ensp00000296028 | ppbp | 4200 | 0.433705081 | 5.014992493 | 0.00658458 |
| ensp00000301061 | wnt10b | 3572 | 0.368855845 | -3.650203704 | 0.0068929 |

| | | | | | |
|---|---|---|---|---|---|
| ensp00000360020 | diras3 | 1487 | 0.153552251 | 3.898676697 | 0.00737116 |
| ensp00000368253 | nr0b1 | 3311 | 0.341904172 | -2.192654692 | 0.00753756 |
| ensp00000369531 | ubqlnl | 1520 | 0.156959934 | -2.663072706 | 0.00764914 |
| ensp00000356671 | serpinc1 | 5519 | 0.569909128 | -2.263686567 | 0.00765692 |
| ensp00000078445 | creb3l3 | 3889 | 0.401590252 | -7.92148433 | 0.0076583 |
| ensp00000239243 | msx2 | 1241 | 0.128149525 | -2.961089334 | 0.00776777 |
| ensp00000257572 | hrk | 3443 | 0.355534903 | 2.695003652 | 0.00785935 |
| ensp00000335657 | cck | 4164 | 0.429987608 | 4.532377361 | 0.0078705 |
| ensp00000225275 | mpo | 4624 | 0.477488641 | 2.951349756 | 0.00787797 |
| ensp00000265459 | nrxn2 | 8395 | 0.866893846 | -3.358123967 | 0.00813586 |
| ensp00000358896 | as3mt | 8218 | 0.848616274 | 7.953492996 | 0.00814644 |
| ensp00000282096 | pde3b | 2717 | 0.280565882 | 13.55697919 | 0.00819945 |
| ensp00000369497 | brca2 | 2217 | 0.228934325 | -3.768656716 | 0.00823625 |
| ensp00000357927 | bnipl | 6357 | 0.656443618 | -2.560306397 | 0.00836968 |
| ensp00000264839 | rims1 | 6991 | 0.721912433 | 2.487001666 | 0.0085401 |
| ensp00000361813 | bex1 | 4417 | 0.456113176 | 2.205605867 | 0.00856805 |
| ensp00000312185 | elmo1 | 4872 | 0.503097893 | 6.303793479 | 0.00862253 |
| ensp00000414598 | mrvi1 | 3621 | 0.373915737 | -3.42020658 | 0.0087332 |
| ensp00000345229 | dner | 3444 | 0.355638166 | -3.341556939 | 0.00884802 |
| ensp00000262345 | il12rb2 | 4529 | 0.467678645 | 4.023335345 | 0.00892369 |
| ensp00000295731 | ihh | 5853 | 0.604399009 | -3.90560177 | 0.00903458 |
| ensp00000371372 | atp12a | 8656 | 0.893845518 | 4.779662637 | 0.00927954 |
| ensp00000379154 | rasgef1a | 6382 | 0.659025196 | 4.102567308 | 0.00929213 |
| ensp00000317214 | capn6 | 2581 | 0.266522098 | 2.039216912 | 0.00935106 |
| ensp00000260598 | khk | 7322 | 0.756092524 | -4.606557377 | 0.00940445 |
| ensp00000354609 | cnksr1 | 5122 | 0.528913672 | 2.561688312 | 0.00973041 |
