# Supplementary material for: Transcriptome analysis reveals manifold mechanisms of cyst development in ADPKD
Source: Hum Genomics. 2016 Nov 21;10:37. doi: 10.1186/s40246-016-0095-x (PMC5117508; doi:10.1186/s40246-016-0095-x)
Supplement: Additional file 2: — Ordered gene list. (PDF 1000 kb) [file 40246_2016_95_MOESM2_ESM.pdf]

Protein	Gene Name	Position #	Ordered List Gene Position
ensp00000354003	gypa	1	0.000103263
ensp00000318409	tmem176b	3	0.000309789
ensp00000004103	tmem176a	4	0.000413052
ensp00000244426	fbxo9	5	0.000516316
ensp00000417601	fbxl2	6	0.000619579
ensp00000420357	rwdd1	9	0.000929368
ensp00000225729	drg2	10	0.001032631
ensp00000368799	kbtbd6	11	0.001135894
ensp00000368797	kbtbd7	12	0.001239157
ensp00000331152	mxi1	13	0.00134242
ensp00000306887	tmem126a	15	0.001548947
ensp00000338235	mtdh	16	0.00165221
ensp00000296597	ndufaf2	17	0.001755473
ensp00000332706	pura	19	0.001961999
ensp00000379051	purb	20	0.002065262
ensp00000388320	ctu2	22	0.002271789
ensp00000264265	lxn	23	0.002375052
ensp00000222482	cpa4	24	0.002478315
ensp00000362616	nap1l2	25	0.002581578
ensp00000362171	nap1l3	26	0.002684841
ensp00000297258	fabp5	27	0.002788104
ensp00000357711	s100a7	28	0.002891367
ensp00000365693	msrb2	30	0.003097893
ensp00000313921	msra	32	0.00330442
ensp00000012134	hivep2	33	0.003407683
ensp00000368698	hivep1	34	0.003510946
ensp00000300107	clpx	35	0.003614209
ensp00000347324	msrb3	37	0.003820735
ensp00000275300	slc22a3	38	0.003923998
ensp00000355930	slc22a1	39	0.004027261
ensp00000282391	pan3	40	0.004130525
ensp00000333920	ttf1	41	0.004233788
ensp00000368754	baz2a	42	0.004337051
ensp00000254325	rfx1	43	0.004440314
ensp00000347514	mier1	44	0.004543577
ensp00000368391	tpd52	45	0.00464684
ensp00000265322	pecr	49	0.005059893
ensp00000215567	tecr	50	0.005163156
ensp00000295491	mrps18c	51	0.005266419
ensp00000265304	ssbp1	52	0.005369682
ensp00000389998	tmem67	55	0.005679471
ensp00000376827	mks1	56	0.005782734
ensp00000335094	tmem17	57	0.005885998
ensp00000261499	b9d1	59	0.006092524
ensp00000398391	cc2d2a	60	0.006195787
ensp00000304941	tctn2	61	0.00629905

ensp00000356774	ahi1	62	0.006402313
ensp00000075120	slc2a3	63	0.006505576
ensp00000358042	qrsl1	68	0.007021892
ensp00000229384	gatc	69	0.007125155
ensp00000356134	tfb1m	70	0.007228418
ensp00000420588	tfam	71	0.007331681
ensp00000355471	tfb2m	73	0.007538207
ensp00000304440	has3	74	0.00764147
ensp00000333926	abcc5	75	0.007744734
ensp00000222115	has1	76	0.007847997
ensp00000338573	gnl3l	77	0.00795126
ensp00000362026	trmt2b	78	0.008054523
ensp00000366271	kdm2b	79	0.008157786
ensp00000265651	fbxo3	80	0.008261049
ensp00000329632	fbxl7	81	0.008364312
ensp00000393154	fbxo15	82	0.008467575
ensp00000244333	lypd3	84	0.008674102
ensp00000289361	btn3a1	85	0.008777365
ensp00000315775	cog4	86	0.008880628
ensp00000204726	golga3	87	0.008983891
ensp00000299886	cog1	88	0.009087154
ensp00000397441	cog6	89	0.009190417
ensp00000348751	btn3a2	94	0.009706733
ensp00000244061	rnf114	95	0.009809996
ensp00000410071	tubb	96	0.009913259
ensp00000401321	notch4	101	0.010429575
ensp00000282641	a1cf	103	0.010636101
ensp00000296370	s100p	108	0.011152416
ensp00000282493	pdzd2	109	0.011255679
ensp00000374409	pkp4	112	0.011565469
ensp00000258443	edar	113	0.011668732
ensp00000363680	eda	114	0.011771995
ensp00000355629	cog2	115	0.011875258
ensp00000305442	cog7	116	0.011978521
ensp00000297135	cog5	117	0.012081784
ensp00000258654	cog3	118	0.012185048
ensp00000305459	cog8	119	0.012288311
ensp00000369129	dsp	120	0.012391574
ensp00000363851	eda2r	121	0.012494837
ensp00000333329	usp17l2	123	0.012701363
ensp00000240304	luc7l3	130	0.013424205
ensp00000232978	nktr	131	0.013527468
ensp00000362702	nek6	134	0.013837257
ensp00000356355	nek7	135	0.01394052
ensp00000238616	nek9	136	0.014043784
ensp00000283921	hoxa10	137	0.014147047
ensp00000326296	meis2	138	0.01425031

ensp00000405890	pbx1	139	0.014353573
ensp00000272369	meis1	140	0.014456836
ensp00000282516	nipbl	142	0.014663362
ensp00000312029	ucp2	144	0.014869888
ensp00000320821	kif1c	145	0.014973152
ensp00000417677	zbtb8os	146	0.015076415
ensp00000351132	rhot1	147	0.015179678
ensp00000328998	trak1	148	0.015282941
ensp00000354850	mgea5	149	0.015386204
ensp00000258457	c2orf49	150	0.015489467
ensp00000380734	fam98b	151	0.01559273
ensp00000233084	ddx1	152	0.015695993
ensp00000160382	actl6b	153	0.015799257
ensp00000269138	ss18	154	0.01590252
ensp00000261700	c14orf166	155	0.016005783
ensp00000361584	znf691	157	0.016212309
ensp00000359740	znf451	159	0.016418835
ensp00000359991	pgam1	160	0.016522098
ensp00000385057	apobec3g	161	0.016625361
ensp00000354775	pja2	162	0.016728625
ensp00000309749	apobec3f	163	0.016831888
ensp00000355001	pou3f3	164	0.016935151
ensp00000240619	tas2r10	166	0.017141677
ensp00000247883	tas2r5	171	0.017657993
ensp00000341625	cyb5a	174	0.017967782
ensp00000365243	cox4i2	175	0.018071045
ensp00000226798	frg1	176	0.018174308
ensp00000263384	fam32a	177	0.018277571
ensp00000355217	snx6	178	0.018380834
ensp00000362608	pim1	179	0.018484097
ensp00000389182	rpp30	181	0.018690624
ensp00000382218	zswim7	182	0.018793887
ensp00000295959	rpp14	183	0.01889715
ensp00000278070	pprc1	184	0.019000413
ensp00000223190	nrf1	185	0.019103676
ensp00000387006	cwc22	186	0.019206939
ensp00000355596	disc1	187	0.019310202
ensp00000315112	rnf31	189	0.019516729
ensp00000381698	sharpin	190	0.019619992
ensp00000293441	shank1	191	0.019723255
ensp00000260058	crebzf	192	0.019826518
ensp00000304689	thap11	193	0.019929781
ensp00000278903	ei24	197	0.020342834
ensp00000245441	nin	199	0.02054936
ensp00000389381	yy2	200	0.020652623
ensp00000338814	bag5	201	0.020755886
ensp00000328671	wdr4	202	0.020859149

ensp00000364801	hspa1b	203	0.020962412
ensp00000285737	lonp2	204	0.021065675
ensp00000327724	tas2r60	207	0.021375465
ensp00000240691	tas2r9	213	0.021995043
ensp00000335677	foxk2	215	0.02220157
ensp00000297792	kdm1b	216	0.022304833
ensp00000351416	ankrd17	217	0.022408096
ensp00000362314	eif4ebp2	218	0.022511359
ensp00000328103	eif4enif1	219	0.022614622
ensp00000196169	tdrd3	220	0.022717885
ensp00000350170	fxr1	221	0.022821148
ensp00000225388	nufip2	223	0.023027675
ensp00000357442	pygo2	224	0.023130938
ensp00000302327	pygo1	225	0.023234201
ensp00000353863	znf148	226	0.023337464
ensp00000355228	hmgn2	227	0.023440727
ensp00000350580	hist1h2bb	228	0.02354399
ensp00000259791	hist1h2ab	229	0.023647253
ensp00000340210	cd59	230	0.023750516
ensp00000354458	c8a	232	0.023957043
ensp00000224181	c8g	233	0.024060306
ensp00000360281	c8b	234	0.024163569
ensp00000322061	c7	235	0.024266832
ensp00000324956	rnf14	237	0.024473358
ensp00000381567	tmf1	238	0.024576621
ensp00000314499	gak	239	0.024679884
ensp00000386520	rad54l2	240	0.024783147
ensp00000339906	pax4	241	0.024886411
ensp00000359506	fmr1	243	0.025092937
ensp00000340505	senp8	244	0.0251962
ensp00000379183	panx2	245	0.025299463
ensp00000262646	rab2a	246	0.025402726
ensp00000327541	blzf1	247	0.025505989
ensp00000309031	vcpip1	248	0.025609252
ensp00000272638	ubxn4	249	0.025712515
ensp00000321617	ankzf1	250	0.025815779
ensp00000370293	hus1b	251	0.025919042
ensp00000234160	gorasp2	252	0.026022305
ensp00000257700	rint1	254	0.026228831
ensp00000234739	bcl9	256	0.026435357
ensp00000217073	pabpc1l	257	0.02653862
ensp00000308012	pabpc5	258	0.026641884
ensp00000281589	pabpc3	259	0.026745147
ensp00000362621	pabpc1l2b	261	0.026951673
ensp00000337623	rnaseh2b	262	0.027054936
ensp00000308193	rnaseh2c	263	0.027158199
ensp00000357905	dmbt1	264	0.027261462

ensp00000272424	tprkb	265	0.027364725
ensp00000314311	larp7	266	0.027467988
ensp00000368459	nufip1	267	0.027571252
ensp00000237853	ell2	268	0.027674515
ensp00000232424	hes1	270	0.027881041
ensp00000272937	hes6	271	0.027984304
ensp00000242462	neurog3	272	0.028087567
ensp00000371786	onecut3	273	0.02819083
ensp00000252809	gdf15	274	0.028294093
ensp00000308926	ppm1j	277	0.028603883
ensp00000306734	sesn1	278	0.028707146
ensp00000344215	tp53inp1	281	0.029016935
ensp00000216639	vrk1	282	0.029120198
ensp00000259939	rnf144b	283	0.029223461
ensp00000397157	perp	284	0.029326724
ensp00000299333	scn3b	286	0.029533251
ensp00000219551	rhbdl1	287	0.029636514
ensp00000331462	znf704	288	0.029739777
ensp00000354045	rrbp1	289	0.02984304
ensp00000323663	dbf4b	290	0.029946303
ensp00000314441	mettl1	291	0.030049566
ensp00000250937	dohh	292	0.030152829
ensp00000210060	dhps	293	0.030256093
ensp00000392147	ppp6c	294	0.030359356
ensp00000262843	mid2	295	0.030462619
ensp00000312678	mid1	296	0.030565882
ensp00000216218	st13	297	0.030669145
ensp00000361900	tomm34	298	0.030772408
ensp00000349525	ahsa2	299	0.030875671
ensp00000299440	rag1	300	0.030978934
ensp00000228289	znf268	302	0.031185461
ensp00000269973	znf45	304	0.031391987
ensp00000326933	sertad2	305	0.03149525
ensp00000348722	pus7	306	0.031598513
ensp00000315182	shq1	307	0.031701776
ensp00000308332	utp23	308	0.031805039
ensp00000370842	il33	309	0.031908302
ensp00000402406	psmb8	311	0.032114829
ensp00000252329	psmg3	312	0.032218092
ensp00000417748	ddi2	313	0.032321355
ensp00000329915	psmg1	314	0.032424618
ensp00000368872	insc	315	0.032527881
ensp00000295822	eif5a2	316	0.032631144
ensp00000336702	eif5a	317	0.032734407
ensp00000353375	map4	319	0.032940933
ensp00000355899	rrp15	320	0.033044197
ensp00000253247	nol11	321	0.03314746

ensp00000380982	kiaa0020	322	0.033250723
ensp00000274289	plk2	323	0.033353986
ensp00000266269	patz1	326	0.033663775
ensp00000346120	ddx21	328	0.033870302
ensp00000258772	ddx56	329	0.033973565
ensp00000261173	atp2b1	331	0.034180091
ensp00000308620	rag2	332	0.034283354
ensp00000377265	tfap2b	334	0.03448988
ensp00000201031	tfap2c	335	0.034593143
ensp00000351593	smtn	336	0.034696406
ensp00000054950	rcn1	337	0.03479967
ensp00000369373	trim5	338	0.034902933
ensp00000319170	dhfrl1	340	0.035109459
ensp00000258484	epc2	341	0.035212722
ensp00000320893	asb8	342	0.035315985
ensp00000343298	lrrc41	343	0.035419248
ensp00000362728	kpna6	344	0.035522511
ensp00000354778	cntnap2	345	0.035625774
ensp00000407310	ppp1r10	346	0.035729038
ensp00000330349	ddx41	347	0.035832301
ensp00000216277	papola	348	0.035935564
ensp00000250024	e2f8	349	0.036038827
ensp00000365505	plp2	350	0.03614209
ensp00000371388	srxn1	351	0.036245353
ensp00000338371	tnrc6b	352	0.036348616
ensp00000379144	tnrc6a	353	0.036451879
ensp00000336783	tnrc6c	354	0.036555143
ensp00000352271	xrcc2	356	0.036761669
ensp00000381237	hm13	358	0.036968195
ensp00000344504	h1f0	359	0.037071458
ensp00000016946	rgpd5	360	0.037174721
ensp00000369162	riok1	361	0.037277984
ensp00000378461	pcbp4	363	0.037484511
ensp00000338770	znf420	365	0.037691037
ensp00000361186	tp53rk	367	0.037897563
ensp00000206542	osgep	368	0.038000826
ensp00000265260	pcnp	369	0.038104089
ensp00000362107	fhl3	370	0.038207352
ensp00000420267	nr6a1	371	0.038310615
ensp00000295934	hesx1	372	0.038413879
ensp00000343746	coro2a	373	0.038517142
ensp00000360660	npdc1	375	0.038723668
ensp00000262888	kcnn4	377	0.038930194
ensp00000311505	iqcb1	378	0.039033457
ensp00000306138	grm5	379	0.03913672
ensp00000377372	gap43	380	0.039239983
ensp00000319104	supt6h	382	0.03944651

ensp00000383301	znf74	383	0.039549773
ensp00000235329	mfn2	384	0.039653036
ensp00000313454	uba6	385	0.039756299
ensp00000359380	scd	386	0.039859562
ensp00000358297	rnf115	387	0.039962825
ensp00000339566	hist1h1c	388	0.040066088
ensp00000284503	neil2	389	0.040169352
ensp00000269346	ttyh2	390	0.040272615
ensp00000333537	sall2	392	0.040479141
ensp00000292782	dcun1d1	394	0.040685667
ensp00000276326	fbxo25	396	0.040892193
ensp00000410007	fbxo46	397	0.040995456
ensp00000258200	fbxl8	399	0.041201983
ensp00000264126	gpsm2	400	0.041305246
ensp00000401867	mxd3	401	0.041408509
ensp00000350331	asb13	403	0.041615035
ensp00000417587	peg10	405	0.041821561
ensp00000298510	prdx3	406	0.041924824
ensp00000328251	mina	407	0.042028088
ensp00000224807	sfxn3	409	0.042234614
ensp00000263239	ddx18	410	0.042337877
ensp00000359478	abcc2	411	0.04244114
ensp00000200652	slc22a4	412	0.042544403
ensp00000294338	pdzk1ip1	413	0.042647666
ensp00000233893	hspe1	416	0.042957456
ensp00000290399	sim2	418	0.043163982
ensp00000262901	sim1	419	0.043267245
ensp00000366482	fxn	420	0.043370508
ensp00000310966	cd3eap	421	0.043473771
ensp00000347178	hspb9	422	0.043577034
ensp00000387278	mpp4	423	0.043680297
ensp00000301011	zc3h18	425	0.043886824
ensp00000262498	c16orf80	426	0.043990087
ensp00000318415	rbmxl1	427	0.04409335
ensp00000350090	hnrnpa1l2	428	0.044196613
ensp00000323687	ube2o	429	0.044299876
ensp00000310590	usp36	430	0.044403139
ensp00000320508	rnf185	431	0.044506402
ensp00000389277	tmbim6	432	0.044609665
ensp00000383911	ubac2	433	0.044712929
ensp00000335106	katna1	434	0.044816192
ensp00000297012	hist1h2aa	435	0.044919455
ensp00000255612	pram1	436	0.045022718
ensp00000265755	gtf2ird1	437	0.045125981
ensp00000358372	nbpf8	438	0.045229244
ensp00000219204	arl2bp	440	0.04543577
ensp00000301068	rhebl1	441	0.045539033

ensp00000216024	dmc1	442	0.045642297
ensp00000334130	c11orf30	443	0.04574556
ensp00000346080	rpl22l1	446	0.046055349
ensp00000298910	lrrk2	447	0.046158612
ensp00000332359	tceal2	448	0.046261875
ensp00000361707	tceal1	450	0.046468401
ensp00000283943	trip12	451	0.046571665
ensp00000216237	l3mbtl2	452	0.046674928
ensp00000307705	hist1h1e	453	0.046778191
ensp00000344393	fcf1	455	0.046984717
ensp00000304133	scg2	456	0.04708798
ensp00000242159	hoxa7	457	0.047191243
ensp00000417303	klf8	458	0.047294506
ensp00000348849	rps26	459	0.04739777
ensp00000253329	ppil4	460	0.047501033
ensp00000306999	esco2	462	0.047707559
ensp00000269142	taf4b	463	0.047810822
ensp00000393159	rfxank	465	0.048017348
ensp00000262376	ubn1	466	0.048120611
ensp00000263208	hira	467	0.048223874
ensp00000411099	tlk1	468	0.048327138
ensp00000307870	ccs	469	0.048430401
ensp00000354346	matr3	471	0.048636927
ensp00000335614	ppfia3	474	0.048946716
ensp00000348784	igbp1	475	0.049049979
ensp00000383402	ppp4r1	476	0.049153242
ensp00000385722	ints1	478	0.049359769
ensp00000318641	ints3	479	0.049463032
ensp00000360821	ubac1	483	0.049876084
ensp00000259335	kiaa0368	484	0.049979347
ensp00000396813	psmb9	485	0.05008261
ensp00000410818	mad2l1bp	488	0.0503924
ensp00000288071	ddx19b	489	0.050495663
ensp00000274680	fars2	490	0.050598926
ensp00000265529	kif9	492	0.050805452
ensp00000160827	kif22	493	0.050908715
ensp00000381824	sac3d1	494	0.051011979
ensp00000318374	leng8	495	0.051115242
ensp00000337405	pcid2	497	0.051321768
ensp00000358571	hipk1	498	0.051425031
ensp00000367991	8-Sep	499	0.051528294
ensp00000330221	spsb1	501	0.05173482
ensp00000311609	spsb4	502	0.051838083
ensp00000345917	lyar	503	0.051941347
ensp00000406888	tcof1	504	0.05204461
ensp00000370968	mybbp1a	505	0.052147873
ensp00000343619	hoxa9	507	0.052354399

ensp00000239165	hoxb7	508	0.052457662
ensp00000291582	aire	509	0.052560925
ensp00000225648	hoxb6	510	0.052664188
ensp00000226105	rangrf	511	0.052767451
ensp00000381290	chtf8	513	0.052973978
ensp00000306123	rpap1	514	0.053077241
ensp00000258455	mrps9	515	0.053180504
ensp00000276585	mrps28	516	0.053283767
ensp00000350815	nr3c2	517	0.05338703
ensp00000346483	ddrgk1	518	0.053490293
ensp00000297229	usp49	519	0.053593556
ensp00000258499	usp44	520	0.053696819
ensp00000349932	ptprs	521	0.053800083
ensp00000316240	dnajc14	523	0.054006609
ensp00000354580	mrpl21	524	0.054109872
ensp00000332162	c1orf64	527	0.054419661
ensp00000310088	ptms	529	0.054626188
ensp00000329454	rcan2	531	0.054832714
ensp00000327107	pdzd3	532	0.054935977
ensp00000342143	pdzk1	533	0.05503924
ensp00000357484	gopc	534	0.055142503
ensp00000261514	clcn3	535	0.055245766
ensp00000331305	tob2	536	0.055349029
ensp00000348593	zfyve27	537	0.055452292
ensp00000311280	elf4	538	0.055555556
ensp00000261574	ipo5	539	0.055658819
ensp00000327179	cirh1a	540	0.055762082
ensp00000268802	nob1	541	0.055865345
ensp00000371169	rcl1	542	0.055968608
ensp00000373810	nop16	543	0.056071871
ensp00000005558	ifrd1	544	0.056175134
ensp00000261741	rbm19	545	0.056278397
ensp00000314193	wdr75	546	0.05638166
ensp00000308179	wdr3	548	0.056588187
ensp00000370589	nop56	549	0.05669145
ensp00000264279	nop58	550	0.056794713
ensp00000254940	nip7	551	0.056897976
ensp00000263657	pno1	552	0.057001239
ensp00000326981	imp3	554	0.057207765
ensp00000371101	nol10	555	0.057311029
ensp00000328854	noc4l	557	0.057517555
ensp00000232888	rrp9	558	0.057620818
ensp00000244230	mphosph10	559	0.057724081
ensp00000202816	esf1	560	0.057827344
ensp00000253237	grwd1	561	0.057930607
ensp00000413572	nle1	562	0.05803387
ensp00000229214	krr1	564	0.058240397

ensp00000291576	pwp2	565	0.05834366
ensp00000368887	pak1ip1	567	0.058550186
ensp00000250237	qtrt1	568	0.058653449
ensp00000366672	dtd1	569	0.058756712
ensp00000360412	noc3l	570	0.058859975
ensp00000362105	utp11l	571	0.058963238
ensp00000283109	riok2	572	0.059066501
ensp00000363642	bms1	573	0.059169765
ensp00000259239	imp4	574	0.059273028
ensp00000236051	ebna1bp2	575	0.059376291
ensp00000253107	ppan	577	0.059582817
ensp00000382392	nop2	578	0.05968608
ensp00000230340	bysl	579	0.059789343
ensp00000257829	nat10	580	0.059892606
ensp00000285814	mki67ip	581	0.059995869
ensp00000362153	gnl2	582	0.060099133
ensp00000353246	mak16	583	0.060202396
ensp00000402338	rpf2	584	0.060305659
ensp00000354040	gtpbp4	585	0.060408922
ensp00000348596	sdad1	587	0.060615448
ensp00000363384	znf593	588	0.060718711
ensp00000304151	bop1	592	0.061131764
ensp00000301364	tsr1	593	0.061235027
ensp00000261015	wdr12	594	0.06133829
ensp00000338862	brix1	595	0.061441553
ensp00000364320	mrto4	596	0.061544816
ensp00000346725	pes1	597	0.061648079
ensp00000359688	rpf1	598	0.061751342
ensp00000322396	rrs1	599	0.061854606
ensp00000226796	gar1	600	0.061957869
ensp00000296435	camp	603	0.062267658
ensp00000338673	cdk10	605	0.062474184
ensp00000234170	cebpz	607	0.06268071
ensp00000395772	gnl3	608	0.062783974
ensp00000303398	mtbp	609	0.062887237
ensp00000340578	rassf6	610	0.0629905
ensp00000417970	fam120b	611	0.063093763
ensp00000368332	arx	612	0.063197026
ensp00000317992	noc2l	613	0.063300289
ensp00000290551	btg2	614	0.063403552
ensp00000256015	btg1	615	0.063506815
ensp00000367817	podxl	618	0.063816605
ensp00000415822	atxn1l	620	0.064023131
ensp00000323913	trim55	621	0.064126394
ensp00000355580	kcnk1	622	0.064229657
ensp00000247153	cfp	623	0.06433292
ensp00000316950	myef2	624	0.064436183

ensp00000355245	pax9	625	0.064539447
ensp00000299001	piwil4	626	0.06464271
ensp00000343745	dicer1	629	0.064952499
ensp00000266987	tarbp2	630	0.065055762
ensp00000318176	prkra	631	0.065159025
ensp00000265351	xpo5	632	0.065262288
ensp00000307525	nmd3	633	0.065365551
ensp00000414237	ints2	634	0.065468815
ensp00000398208	ints9	635	0.065572078
ensp00000327889	ints5	637	0.065778604
ensp00000310260	ints6	638	0.065881867
ensp00000343274	ints8	639	0.06598513
ensp00000261693	scarb1	641	0.066191656
ensp00000221801	fbl	642	0.066294919
ensp00000320557	chfr	643	0.066398183
ensp00000249364	calu	644	0.066501446
ensp00000340176	rbpms	645	0.066604709
ensp00000272223	osr1	646	0.066707972
ensp00000358062	aim1	647	0.066811235
ensp00000300249	mapre2	649	0.067017761
ensp00000359024	nolc1	651	0.067224287
ensp00000367808	c1orf86	652	0.067327551
ensp00000304336	pydc1	653	0.067430814
ensp00000376849	casp5	655	0.06763734
ensp00000218340	rp2	656	0.067740603
ensp00000337040	unc119	657	0.067843866
ensp00000366997	dis3	658	0.067947129
ensp00000361433	exosc2	659	0.068050392
ensp00000265564	exosc7	660	0.068153656
ensp00000263579	dcps	661	0.068256919
ensp00000324203	wrap53	662	0.068360182
ensp00000274606	nhp2	663	0.068463445
ensp00000358563	dkc1	664	0.068566708
ensp00000268043	pif1	665	0.068669971
ensp00000221855	tbcb	666	0.068773234
ensp00000355560	tbce	668	0.06897976
ensp00000347719	tbcd	669	0.069083024
ensp00000244625	tbcc	670	0.069186287
ensp00000397453	ube2w	671	0.06928955
ensp00000330813	brap	672	0.069392813
ensp00000302051	ecel1	673	0.069496076
ensp00000346027	rpl21	676	0.069805865
ensp00000355317	wdtc1	677	0.069909128
ensp00000297579	dcaf13	678	0.070012392
ensp00000352928	dda1	679	0.070115655
ensp00000356737	gorab	680	0.070218918
ensp00000261326	mocos	682	0.070425444

ensp00000303019	gphn		684	0.07063197
ensp00000321711	dis3l		685	0.070735233
ensp00000352678	supv3l1		686	0.070838496
ensp00000348107	c1d		687	0.07094176
ensp00000258169	mphosph6		688	0.071045023
ensp00000322408	kdm5d		689	0.071148286
ensp00000363064	znf76		691	0.071354812
ensp00000265990	btaf1		692	0.071458075
ensp00000260447	gchfr		693	0.071561338
ensp00000295453	alppl2		694	0.071664601
ensp00000295463	alpi		695	0.071767865
ensp00000339692	rassf4		696	0.071871128
ensp00000336616	rassf3		697	0.071974391
ensp00000328939	uty		698	0.072077654
ensp00000342812	usp9y		699	0.072180917
ensp00000155093	zfy		701	0.072387443
ensp00000336725	ddx3y		702	0.072490706
ensp00000382840	ddx3x		703	0.072593969
ensp00000366843	atxn2		704	0.072697233
ensp00000299626	alg8		705	0.072800496
ensp00000392330	bcap31		707	0.073007022
ensp00000328287	rnf123		708	0.073110285
ensp00000348704	kpna5		709	0.073213548
ensp00000307181	fam103a1		712	0.073523337
ensp00000266483	alg10		713	0.073626601
ensp00000310120	alg10b		714	0.073729864
ensp00000306275	kcnk3		717	0.074039653
ensp00000265727	adam22		718	0.074142916
ensp00000375857	lrrfip1		719	0.074246179
ensp00000338727	lrrfip2		720	0.074349442
ensp00000246747	arl2		721	0.074452705
ensp00000377769	arl13b		722	0.074555969
ensp00000391311		5-Sep	723	0.074659232
ensp00000364204	pink1		725	0.074865758
ensp00000366396	xrn2		727	0.075072284
ensp00000298746	trub1		728	0.075175547
ensp00000263629	mtif2		729	0.07527881
ensp00000365624	gripap1		731	0.075485337
ensp00000312222	zhx3		733	0.075691863
ensp00000285407	klf10		734	0.075795126
ensp00000279227	fermt3		735	0.075898389
ensp00000342858	fermt2		736	0.076001652
ensp00000273062	ctdsp1		737	0.076104915
ensp00000273179	ctdspl		738	0.076208178
ensp00000381148	ctdsp2		739	0.076311442
ensp00000264951	xrn1		741	0.076517968
ensp00000398597	exosc6		742	0.076621231

ensp00000366135	exosc10	743	0.076724494
ensp00000315476	exosc4	744	0.076827757
ensp00000374354	exosc8	745	0.07693102
ensp00000323046	exosc3	746	0.077034283
ensp00000221233	exosc5	747	0.077137546
ensp00000368984	exosc9	748	0.07724081
ensp00000314608	klhl11	749	0.077344073
ensp00000298992	abtb2	750	0.077447336
ensp00000328352	kctd11	751	0.077550599
ensp00000306561	ogg1	752	0.077653862
ensp00000217372	cdk5rap1	753	0.077757125
ensp00000246868	sbds	754	0.077860388
ensp00000387911	parn	755	0.077963651
ensp00000359939	exosc1	756	0.078066914
ensp00000373715	dcp2	757	0.078170178
ensp00000381216	khsrp	758	0.078273441
ensp00000282486	mbnl1	759	0.078376704
ensp00000371155	nupl1	760	0.078479967
ensp00000268957	tob1	762	0.078686493
ensp00000265192	paip2	763	0.078789756
ensp00000302768	paip1	764	0.078893019
ensp00000355279	cnot7	765	0.078996283
ensp00000354676	ostc	770	0.079512598
ensp00000376472	stt3a	772	0.079719124
ensp00000295770	stt3b	773	0.079822387
ensp00000222329	erf	775	0.080028914
ensp00000300181	tsc22d4	776	0.080132177
ensp00000313050	gbas	777	0.08023544
ensp00000004982	hspb6	779	0.080441966
ensp00000318085	hinfp	782	0.080751755
ensp00000339086	mkl2	783	0.080855019
ensp00000314132	bok	785	0.081061545
ensp00000326884	arfrp1	786	0.081164808
ensp00000401514	dnah1	787	0.081268071
ensp00000251775	snx4	788	0.081371334
ensp00000373825	dnah2	789	0.081474597
ensp00000301921	sox7	793	0.08188765
ensp00000262715	tep1	794	0.081990913
ensp00000342535	nlgn4y	796	0.082197439
ensp00000309606	znf408	797	0.082300702
ensp00000320503	edc3	798	0.082403965
ensp00000310596	lsm1	800	0.082610492
ensp00000252622	lsm7	801	0.082713755
ensp00000296581	lsm6	802	0.082817018
ensp00000410758	lsm5	803	0.082920281
ensp00000206474	haus4	804	0.083023544
ensp00000369871	haus6	807	0.083333333

ensp00000243706	haus3	808	0.083436596
ensp00000253669	haus8	809	0.08353986
ensp00000264018	ddx6	811	0.083746386
ensp00000300146	patl1	812	0.083849649
ensp00000364813	lsm2	813	0.083952912
ensp00000297109	sap30l	814	0.084056175
ensp00000250916	klf16	815	0.084159438
ensp00000307023	klf11	816	0.084262701
ensp00000311273	dnah7	819	0.084572491
ensp00000216068	dnal4	821	0.084779017
ensp00000382379	ankrd28	822	0.08488228
ensp00000297908	mrrf	823	0.084985543
ensp00000241600	mrps2	824	0.085088806
ensp00000380702	mycbp	825	0.085192069
ensp00000337386	zfp36l1	826	0.085295333
ensp00000336712	tnpo1	827	0.085398596
ensp00000248673	zfp36	828	0.085501859
ensp00000280362	pts	829	0.085605122
ensp00000375881	alpp	830	0.085708385
ensp00000240185	tardbp	831	0.085811648
ensp00000361949	pabpc4	832	0.085914911
ensp00000220959	ubr5	833	0.086018174
ensp00000270632	spib	835	0.086224701
ensp00000337439	dact1	837	0.086431227
ensp00000367841	dynlt3	838	0.08653449
ensp00000327191	cct6b	840	0.086741016
ensp00000358529	tspan2	844	0.087154069
ensp00000349052	oscp1	845	0.087257332
ensp00000355145	prmt6	846	0.087360595
ensp00000273783	eif2b5	847	0.087463858
ensp00000266126	eif2b2	848	0.087567121
ensp00000353575	eif2b3	849	0.087670384
ensp00000252602	mrpl34	851	0.08787691
ensp00000371734	kank1	852	0.087980173
ensp00000316948	clk4	854	0.0881867
ensp00000388330	tsfm	856	0.088393226
ensp00000315017	mrpl1	857	0.088496489
ensp00000274311	pelo	858	0.088599752
ensp00000297857	zhx1	860	0.088806278
ensp00000299427	tpp1	861	0.088909542
ensp00000363559	eif6	862	0.089012805
ensp00000296802	nsa2	863	0.089116068
ensp00000296577	abce1	864	0.089219331
ensp00000320252	ascc3	865	0.089322594
ensp00000292314	ccdc12	866	0.089425857
ensp00000261951	cnot6	867	0.08952912
ensp00000229195	cnot2	868	0.089632383

ensp00000330060	cnot10	869	0.089735646
ensp00000320949	cnot1	872	0.090045436
ensp00000273064	rqcd1	873	0.090148699
ensp00000264903	cnot6l	875	0.090355225
ensp00000350990	tnks1bp1	876	0.090458488
ensp00000354673	cnot4	877	0.090561751
ensp00000053468	mrps10	878	0.090665014
ensp00000296102	mrpl33	879	0.090768278
ensp00000341082	mrpl20	880	0.090871541
ensp00000225969	mrpl27	881	0.090974804
ensp00000333837	mrpl12	882	0.091078067
ensp00000354525	mrpl24	884	0.091284593
ensp00000317376	mrps11	885	0.091387856
ensp00000288937	mrpl17	886	0.091491119
ensp00000373404	mrpl2	887	0.091594382
ensp00000253099	mrpl4	888	0.091697646
ensp00000264995	mrpl3	889	0.091800909
ensp00000420714	mrps14	890	0.091904172
ensp00000362036	mrps16	891	0.092007435
ensp00000350686	ankle2	892	0.092110698
ensp00000230640	skiv2l2	894	0.092317224
ensp00000337313	zcchc8	895	0.092420487
ensp00000222644	mpp6	896	0.092523751
ensp00000306697	mrap	897	0.092627014
ensp00000369895	gemin8	898	0.092730277
ensp00000367265	ckap4	899	0.09283354
ensp00000233616	mogs	900	0.092936803
ensp00000305230	srp9	901	0.093040066
ensp00000411177	mrpl22	904	0.093349855
ensp00000285298	mrps17	905	0.093453119
ensp00000354722	eif1ay	906	0.093556382
ensp00000237530	rpn2	912	0.09417596
ensp00000282999	srp19	914	0.094382487
ensp00000308897	mrpl11	916	0.094589013
ensp00000306548	mrpl13	917	0.094692276
ensp00000245539	mrps7	918	0.094795539
ensp00000308845	mrps12	919	0.094898802
ensp00000322439	tufm	920	0.095002065
ensp00000419038	gfm1	921	0.095105328
ensp00000296805	gfm2	922	0.095208591
ensp00000260102	mrpl15	923	0.095311855
ensp00000296411	metap1	924	0.095415118
ensp00000298428	sec61a2	929	0.095931433
ensp00000354812	c9orf114	933	0.096344486
ensp00000299714	sec11c	936	0.096654275
ensp00000263672	spcs2	937	0.096757538
ensp00000233025	spcs1	938	0.096860801

ensp00000312066	srp68	942	0.097273854
ensp00000362688	eif3i	945	0.097583643
ensp00000261868	eif3j	946	0.097686906
ensp00000348708	upf2	947	0.097790169
ensp00000262803	upf1	948	0.097893432
ensp00000260443	rsl24d1	949	0.097996696
ensp00000298283	rpl10l	952	0.098306485
ensp00000250784	rps4y1	955	0.098616274
ensp00000288666	rps4y2	956	0.098719537
ensp00000232905	eif1b	959	0.099029327
ensp00000368927	eif1ax	960	0.09913259
ensp00000416892	eif3l	961	0.099235853
ensp00000319910	eif3m	962	0.099339116
ensp00000370258	eif3cl	963	0.099442379
ensp00000332604	eif3c	965	0.099648905
ensp00000369681	usp3	966	0.099752169
ensp00000296255	rpn1	967	0.099855432
ensp00000369757	rps6	972	0.100371747
ensp00000262056	eif4b	973	0.10047501
ensp00000252115	poldip3	975	0.100681537
ensp00000398131	gspt1	976	0.1007848
ensp00000389103	rpl23a	980	0.101197852
ensp00000346067	rpsa	990	0.102230483
ensp00000346012	rpl36al	994	0.102643536
ensp00000364543	skiv2l	997	0.102953325
ensp00000412310	skiv2l	998	0.103056588
ensp00000318646	rps15a	1005	0.10377943
ensp00000268661	rpl3l	1006	0.103882693
ensp00000265044	ssr3	1007	0.103985956
ensp00000244763	ssr1	1009	0.104192482
ensp00000221975	rps19	1012	0.104502272
ensp00000216554	eif5	1018	0.10512185
ensp00000289371	eif5b	1019	0.105225114
ensp00000353741	etf1	1020	0.105328377
ensp00000263073	smg6	1021	0.10543164
ensp00000379339	rps29	1022	0.105534903
ensp00000346015	rpl27a	1023	0.105638166
ensp00000377865	rpl23	1024	0.105741429
ensp00000262584	rpl8	1026	0.105947955
ensp00000287038	rpl30	1036	0.106980587
ensp00000339795	rpl7	1045	0.107909955
ensp00000362744	rps4x	1047	0.108116481
ensp00000307889	rpl13	1049	0.108323007
ensp00000310040	eif3f	1053	0.108736059
ensp00000276682	eif3h	1054	0.108839323
ensp00000220849	eif3e	1055	0.108942586
ensp00000248342	eif3k	1056	0.109045849

ensp00000354739	rpl12	1066	0.11007848
ensp00000293842	rpl26	1069	0.110388269
ensp00000346050	rps3a	1072	0.110698059
ensp00000368350	tpt1	1074	0.110904585
ensp00000302886	pa2g4	1080	0.111524164
ensp00000221784	pdcd5	1083	0.111833953
ensp00000323580	ift88	1084	0.111937216
ensp00000260570	ift172	1085	0.112040479
ensp00000240327	spop	1086	0.112143742
ensp00000391944	eef1d	1088	0.112350268
ensp00000217182	eef1a2	1089	0.112453532
ensp00000369042	ipo7	1090	0.112556795
ensp00000326381	eif4a2	1094	0.112969847
ensp00000313007	pabpc1	1095	0.11307311
ensp00000341247	gspt2	1096	0.113176373
ensp00000401450	rpl28	1097	0.113279637
ensp00000274242	rpl37	1100	0.113589426
ensp00000309334	rpl15	1102	0.113795952
ensp00000294189	rpl29	1103	0.113899215
ensp00000307940	eef2	1105	0.114105741
ensp00000256383	eif2s1	1106	0.114209005
ensp00000364119	eif2s2	1107	0.114312268
ensp00000378160	rpl34	1108	0.114415531
ensp00000296674	rps23	1109	0.114518794
ensp00000311028	rps14	1111	0.11472532
ensp00000346088	rpl22	1113	0.114931846
ensp00000328023	srpr	1114	0.115035109
ensp00000341730	rpl10	1115	0.115138373
ensp00000234677	sars	1117	0.115344899
ensp00000263867	capg	1118	0.115448162
ensp00000301732	abca3	1120	0.115654688
ensp00000347906	prmt2	1121	0.115757951
ensp00000262213	tram1	1122	0.115861214
ensp00000342181	srp72	1123	0.115964477
ensp00000350278	slc30a7	1125	0.116171004
ensp00000319240	magohb	1127	0.11637753
ensp00000386156	wibg	1128	0.116480793
ensp00000402515	smg1	1129	0.116584056
ensp00000269349	eif4a3	1130	0.116687319
ensp00000364448	upf3a	1131	0.116790582
ensp00000264645	casc3	1132	0.116893846
ensp00000369996	atxn3l	1135	0.117203635
ensp00000357998	sec63	1136	0.117306898
ensp00000337688	sec62	1137	0.117410161
ensp00000370373	fkbp1b	1138	0.117513424
ensp00000262850	rragb	1139	0.117616687
ensp00000369899	rraga	1140	0.11771995

ensp00000351596	ttc37	1141	0.117823214
ensp00000400626	skiv2l	1142	0.117926477
ensp00000233615	wbp1	1143	0.11802974
ensp00000294753	znf496	1144	0.118133003
ensp00000261884	trip4	1145	0.118236266
ensp00000040584	hoxc8	1147	0.118442792
ensp00000336606	rad54b	1148	0.118546055
ensp00000339627	fli1	1149	0.118649318
ensp00000262426	foxf1	1150	0.118752582
ensp00000261438	klf3	1151	0.118855845
ensp00000383719	ube2j2	1152	0.118959108
ensp00000366748	aup1	1156	0.11937216
ensp00000330054	eef1a1	1157	0.119475423
ensp00000309198	ube3c	1158	0.119578686
ensp00000348632	rbck1	1159	0.11968195
ensp00000280557	denr	1161	0.119888476
ensp00000340329	caprin1	1162	0.119991739
ensp00000359837	zzz3	1163	0.120095002
ensp00000404623	daxx	1165	0.120301528
ensp00000326813	yod1	1166	0.120404791
ensp00000369075	cenpb	1167	0.120508055
ensp00000291281	prkd2	1168	0.120611318
ensp00000360922	stau1	1169	0.120714581
ensp00000344524	ppp1r9a	1170	0.120817844
ensp00000339145	rrp1b	1171	0.120921107
ensp00000261167	wbp11	1172	0.12102437
ensp00000337632	sarnp	1173	0.121127633
ensp00000346236	ddx46	1174	0.121230896
ensp00000246190	necab3	1175	0.121334159
ensp00000321724	insl3	1176	0.121437423
ensp00000262177	dnajb6	1178	0.121643949
ensp00000243253	sec61a1	1179	0.121747212
ensp00000341538	sec61g	1180	0.121850475
ensp00000223641	sec61b	1181	0.121953738
ensp00000380460	plaa	1182	0.122057001
ensp00000229268	usp5	1183	0.122160264
ensp00000366999	hist1h3d	1184	0.122263527
ensp00000254810	h3f3b	1185	0.122366791
ensp00000292524	lrrc14	1187	0.122573317
ensp00000307833	fbxo22	1188	0.12267658
ensp00000255608	btbd2	1189	0.122779843
ensp00000295974	apbb2	1190	0.122883106
ensp00000227638	panx1	1191	0.122986369
ensp00000261826	p2rx7	1192	0.123089632
ensp00000376973	isy1	1194	0.123296159
ensp00000300417	lrsam1	1195	0.123399422
ensp00000218004	nxt2	1197	0.123605948

ensp00000254998	nxt1	1198	0.123709211
ensp00000357882	pstk	1199	0.123812474
ensp00000318154	mrfap1l1	1200	0.123915737
ensp00000258416	eif4e2	1202	0.124122264
ensp00000340281	eif4g2	1203	0.124225527
ensp00000393324	eif4e3	1204	0.12432879
ensp00000378504	nxf3	1206	0.124535316
ensp00000263032	nxf5	1207	0.124638579
ensp00000242810	klhl24	1209	0.124845105
ensp00000232766	klhl18	1210	0.124948368
ensp00000297071	tra2a	1211	0.125051632
ensp00000260210	bud13	1212	0.125154895
ensp00000380414	dek	1213	0.125258158
ensp00000334280	tacc2	1214	0.125361421
ensp00000347444	tdrd7	1215	0.125464684
ensp00000321703	tacc1	1216	0.125567947
ensp00000264313	slain2	1217	0.12567121
ensp00000352442	hist1h2bm	1221	0.126084263
ensp00000373730	h2afj	1222	0.126187526
ensp00000366581	hist1h4b	1223	0.126290789
ensp00000216832	pnn	1224	0.126394052
ensp00000253363	rbm39	1225	0.126497315
ensp00000158771	derl2	1229	0.126910368
ensp00000280700	ngly1	1230	0.127013631
ensp00000265097	thoc3	1231	0.127116894
ensp00000326531	thoc6	1232	0.127220157
ensp00000245838	thoc2	1233	0.12732342
ensp00000380969	thoc5	1234	0.127426683
ensp00000278279	tut1	1235	0.127529946
ensp00000265560	usp4	1236	0.127633209
ensp00000228284	sart3	1237	0.127736473
ensp00000292535	cux1	1238	0.127839736
ensp00000305973	hoxc4	1239	0.127942999
ensp00000239243	msx2	1241	0.128149525
ensp00000295899	thoc7	1242	0.128252788
ensp00000261600	thoc1	1244	0.128459314
ensp00000260970	ppig	1246	0.128665841
ensp00000286175	ppil3	1247	0.128769104
ensp00000373657	c17orf85	1250	0.129078893
ensp00000358605	smndc1	1251	0.129182156
ensp00000242784	c19orf43	1252	0.129285419
ensp00000390427	ppil2	1254	0.129491945
ensp00000337194	prpf4b	1255	0.129595209
ensp00000308546	mepce	1256	0.129698472
ensp00000414006	lsm2	1257	0.129801735
ensp00000414634	lsm2	1259	0.130008261
ensp00000302160	lsm3	1261	0.130214787

ensp00000380336	smu1	1262	0.13031805
ensp00000354518	znf830	1263	0.130421314
ensp00000296015	ttc14	1264	0.130524577
ensp00000267812	mfap1	1265	0.13062784
ensp00000360652	cxorf56	1266	0.130731103
ensp00000310448	sart1	1267	0.130834366
ensp00000360365	mcts1	1268	0.130937629
ensp00000252137	dgcr14	1270	0.131144155
ensp00000262415	dhx8	1271	0.131247418
ensp00000257181	prpf38a	1274	0.131557208
ensp00000336741	dhx15	1277	0.131866997
ensp00000246957	trap1	1278	0.13197026
ensp00000308944	hltf	1279	0.132073523
ensp00000356473	shprh	1280	0.132176786
ensp00000261973	rbm25	1281	0.13228005
ensp00000312981	usp39	1282	0.132383313
ensp00000417229	eif2a	1283	0.132486576
ensp00000340766	smg7	1285	0.132693102
ensp00000364289	ncbp1	1287	0.132899628
ensp00000326806	ncbp2	1288	0.133002891
ensp00000276201	upf3b	1289	0.133106154
ensp00000333001	rbm8a	1290	0.133209418
ensp00000293831	eif4a1	1291	0.133312681
ensp00000376309	hnrnpa3	1292	0.133415944
ensp00000341826	hnrnpa1	1293	0.133519207
ensp00000370083	smn1	1294	0.13362247
ensp00000370119	smn2	1295	0.133725733
ensp00000261667	kpna3	1296	0.133828996
ensp00000309831	snupn	1297	0.133932259
ensp00000321706	gemin4	1298	0.134035523
ensp00000358716	ddx20	1299	0.134138786
ensp00000270257	gemin7	1300	0.134242049
ensp00000285873	gemin5	1301	0.134345312
ensp00000281950	gemin6	1302	0.134448575
ensp00000263309	clns1a	1303	0.134551838
ensp00000315379	prpf3	1306	0.134861627
ensp00000324274	zcchc10	1308	0.135068154
ensp00000360497	rnf113a	1309	0.135171417
ensp00000297151	slu7	1310	0.13527468
ensp00000369218	rbm17	1311	0.135377943
ensp00000355050	ctnnbl1	1312	0.135481206
ensp00000347592	sap30bp	1314	0.135687732
ensp00000400591	snrpe	1315	0.135790995
ensp00000300413	snrpd1	1316	0.135894259
ensp00000412566	snrpb	1317	0.135997522
ensp00000383059	arglu1	1318	0.136100785
ensp00000352956	rbm23	1319	0.136204048

ensp00000377262	srpk2	1320	0.136307311
ensp00000391069	srpk1	1321	0.136410574
ensp00000332790	hist2h2ab	1323	0.1366171
ensp00000296215	snip1	1324	0.136720363
ensp00000354478	dlx1	1325	0.136823627
ensp00000319341	lsm10	1326	0.13692689
ensp00000286307	lsm11	1327	0.137030153
ensp00000270617	znf473	1328	0.137133416
ensp00000396301	ik	1330	0.137339942
ensp00000316109	aggf1	1332	0.137546468
ensp00000416959	tra2b	1333	0.137649732
ensp00000301740	srrm2	1334	0.137752995
ensp00000219169	nutf2	1336	0.137959521
ensp00000305503	nup62	1337	0.138062784
ensp00000254508	nup210	1338	0.138166047
ensp00000231498	nup155	1339	0.13826931
ensp00000294172	nxf1	1340	0.138372573
ensp00000326261	srrm1	1342	0.1385791
ensp00000268482	dhx38	1348	0.139198678
ensp00000291552	u2af1	1349	0.139301941
ensp00000305556	pcbp1	1351	0.139508468
ensp00000313199	hnrnpd	1353	0.139714994
ensp00000305790	sf3b3	1354	0.139818257
ensp00000335321	sf3b1	1355	0.13992152
ensp00000317123	snrnp200	1356	0.140024783
ensp00000304350	prpf8	1357	0.140128046
ensp00000215956	nhp2l1	1358	0.140231309
ensp00000319690	hnrnpc	1359	0.140334572
ensp00000221448	snrnp70	1360	0.140437836
ensp00000352308	ddx42	1361	0.140541099
ensp00000227524	prpf19	1363	0.140747625
ensp00000368801	wbp4	1364	0.140850888
ensp00000246194	raly	1365	0.140954151
ensp00000265866	hnrnph3	1366	0.141057414
ensp00000362803	ppil1	1367	0.141160677
ensp00000365747	pqbp1	1368	0.141263941
ensp00000156471	aqr	1369	0.141367204
ensp00000354951	qki	1370	0.141470467
ensp00000262633	rbm42	1371	0.14157373
ensp00000199814	rbm22	1372	0.141676993
ensp00000358554	bcas2	1373	0.141780256
ensp00000351108	hnrnpab	1374	0.141883519
ensp00000417686	slbp	1375	0.141986782
ensp00000272348	snrpg	1376	0.142090045
ensp00000266735	snrpf	1377	0.142193309
ensp00000215829	snrpd3	1379	0.142399835
ensp00000363313	prpf4	1380	0.142503098

ensp00000216252	phf5a	1382	0.142709624
ensp00000309166	rbm4	1385	0.143019413
ensp00000361927	hnrnph2	1386	0.143122677
ensp00000361162	toe1	1387	0.14322594
ensp00000156109	gpkow	1388	0.143329203
ensp00000311677	ppp1r8	1389	0.143432466
ensp00000271628	sf3b4	1390	0.143535729
ensp00000265872	ccar1	1391	0.143638992
ensp00000375863	hnrnpul1	1392	0.143742255
ensp00000316042	hnrnpa0	1393	0.143845518
ensp00000269601	txnl4a	1394	0.143948781
ensp00000310723	ddx23	1395	0.144052045
ensp00000349428	ptbp1	1396	0.144155308
ensp00000243563	snrpa	1397	0.144258571
ensp00000244520	snrpc	1398	0.144361834
ensp00000366607	sf1	1399	0.144465097
ensp00000215793	sf3a1	1400	0.14456836
ensp00000365439	hnrnpk	1401	0.144671623
ensp00000412788	ptbp2	1403	0.14487815
ensp00000348108	khdrbs3	1404	0.144981413
ensp00000322887	cirbp	1405	0.145084676
ensp00000296702	tcerg1	1407	0.145291202
ensp00000346694	hnrnpa2b1	1408	0.145394465
ensp00000254108	fus	1409	0.145497728
ensp00000216727	pabpn1	1410	0.145600991
ensp00000304903	cd2bp2	1411	0.145704254
ensp00000221494	sf3a2	1412	0.145807518
ensp00000343054	rbm5	1413	0.145910781
ensp00000352438	pcbp2	1414	0.146014044
ensp00000314491	srrt	1415	0.146117307
ensp00000359727	bag2	1417	0.146323833
ensp00000337226	cdca4	1418	0.146427096
ensp00000364639	rbm7	1419	0.146530359
ensp00000356541	sf3b5	1421	0.146736886
ensp00000298281	pcf11	1422	0.146840149
ensp00000345412	cpsf7	1423	0.146943412
ensp00000300291	nudt21	1424	0.147046675
ensp00000298875	cpsf2	1425	0.147149938
ensp00000339353	cpsf1	1426	0.147253201
ensp00000307863	u2af2	1427	0.147356464
ensp00000361658	nup188	1428	0.147459727
ensp00000345895	nup50	1429	0.14756299
ensp00000295119	nup35	1430	0.147666254
ensp00000209873	aaas	1431	0.147769517
ensp00000310668	nup93	1432	0.14787278
ensp00000262077	nup153	1433	0.147976043
ensp00000352400	nup214	1434	0.148079306

ensp00000367454	dffb	1435	0.148182569
ensp00000262710	acin1	1436	0.148285832
ensp00000297540	phax	1437	0.148389095
ensp00000258742	nupl2	1438	0.148492359
ensp00000360286	rae1	1439	0.148595622
ensp00000316032	nup98	1440	0.148698885
ensp00000382779	seh1l	1441	0.148802148
ensp00000351506	mrpl42	1442	0.148905411
ensp00000391774	cpsf6	1443	0.149008674
ensp00000292476	cpsf4	1444	0.149111937
ensp00000359645	rbmx	1446	0.149318463
ensp00000322016	puf60	1447	0.149421727
ensp00000349892	mycbp2	1449	0.149628253
ensp00000346886	gabpa	1451	0.149834779
ensp00000404121	ilf3	1452	0.149938042
ensp00000360532	cdc5l	1453	0.150041305
ensp00000349168	hnrnph1	1454	0.150144568
ensp00000338477	hnrnpf	1455	0.150247831
ensp00000254193	snrpa1	1456	0.150351095
ensp00000315791	cstf3	1457	0.150454358
ensp00000325376	hnrnpm	1458	0.150557621
ensp00000356520	dhx9	1459	0.150660884
ensp00000283179	hnrnpu	1460	0.150764147
ensp00000221419	hnrnpl	1461	0.15086741
ensp00000385269	elavl1	1462	0.150970673
ensp00000303191	plrg1	1463	0.151073936
ensp00000366557	crnkl1	1464	0.1511772
ensp00000378165	znf207	1465	0.151280463
ensp00000218364	htatsf1	1466	0.151383726
ensp00000298717	mettl3	1467	0.151486989
ensp00000304704	clp1	1468	0.151590252
ensp00000363745	hnrnpr	1469	0.151693515
ensp00000263697	dnajc8	1470	0.151796778
ensp00000362110	sf3a3	1471	0.151900041
ensp00000246071	snrpb2	1472	0.152003304
ensp00000263694	snrnp40	1473	0.152106568
ensp00000306614	ppih	1475	0.152313094
ensp00000264233	polq	1476	0.152416357
ensp00000370555	gyg2	1477	0.15251962
ensp00000313983	whsc1l1	1478	0.152622883
ensp00000305899	suv420h1	1479	0.152726146
ensp00000380033	ddx17	1480	0.152829409
ensp00000357341	ncoa7	1482	0.153035936
ensp00000234142	greb1	1483	0.153139199
ensp00000292123	safb	1484	0.153242462
ensp00000361473	kdm4a	1485	0.153345725
ensp00000398812	fkbpl	1486	0.153448988

ensp00000360020	diras3	1487	0.153552251
ensp00000337265	osbpl9	1488	0.153655514
ensp00000171111	keap1	1490	0.15386204
ensp00000370150	wrnip1	1491	0.153965304
ensp00000310275	banf1	1492	0.154068567
ensp00000285968	nup205	1493	0.15417183
ensp00000264883	nup54	1494	0.154275093
ensp00000225696	nup88	1495	0.154378356
ensp00000356448	tpr	1496	0.154481619
ensp00000368856	nfx1	1497	0.154584882
ensp00000304370	cdc40	1498	0.154688145
ensp00000238112	cpsf3	1502	0.155101198
ensp00000362063	cstf2	1503	0.155204461
ensp00000217109	cstf1	1504	0.155307724
ensp00000381031	ewsr1	1505	0.155410987
ensp00000295148	c2orf44	1506	0.15551425
ensp00000262265	pih1d1	1508	0.155720777
ensp00000262032	ikzf4	1510	0.155927303
ensp00000311579	tnks	1511	0.156030566
ensp00000360689	tnks2	1512	0.156133829
ensp00000323659	kdm3a	1513	0.156237092
ensp00000330330	tmprss2	1514	0.156340355
ensp00000414377	ehmt2	1516	0.156546881
ensp00000326477	setdb2	1517	0.156650145
ensp00000386759	setd2	1518	0.156753408
ensp00000319208	suv39h2	1519	0.156856671
ensp00000369531	ubqlnl	1520	0.156959934
ensp00000347997	ubqln3	1521	0.157063197
ensp00000350967	elp2	1522	0.15716646
ensp00000267973	wdr61	1523	0.157269723
ensp00000343515	tcea2	1526	0.157579513
ensp00000320346	ell3	1528	0.157786039
ensp00000328773	hexim1	1529	0.157889302
ensp00000395497	gtf2h4	1533	0.158302354
ensp00000396251	gtf2h4	1534	0.158405618
ensp00000259895	gtf2h4	1535	0.158508881
ensp00000332791	klhl15	1536	0.158612144
ensp00000356584	tor1aip2	1537	0.158715407
ensp00000348578	g3bp1	1538	0.15881867
ensp00000219473	usp10	1539	0.158921933
ensp00000360992	stambpl1	1540	0.159025196
ensp00000363095	trim32	1541	0.159128459
ensp00000352676	zranb1	1542	0.159231722
ensp00000321503	pgam5	1543	0.159334986
ensp00000218348	usp11	1544	0.159438249
ensp00000225792	ddx5	1545	0.159541512
ensp00000319169	prmt5	1546	0.159644775

ensp00000235090	wdr77	1547	0.159748038
ensp00000359818	mms19	1548	0.159851301
ensp00000262173	rnmt	1549	0.159954564
ensp00000264157	ccnt2	1550	0.160057827
ensp00000256897	ccnh	1552	0.160264354
ensp00000261245	mnat1	1553	0.160367617
ensp00000256443	cdk7	1554	0.16047088
ensp00000348089	ercc6	1555	0.160574143
ensp00000356405	cdc73	1557	0.160780669
ensp00000264211	eif4g3	1558	0.160883932
ensp00000280892	eif4e	1559	0.160987195
ensp00000316879	eif4g1	1560	0.161090458
ensp00000355261	smg5	1561	0.161193722
ensp00000196061	plod1	1562	0.161296985
ensp00000361626	ybx1	1563	0.161400248
ensp00000358635	syncrip	1564	0.161503511
ensp00000299601	leo1	1565	0.161606774
ensp00000225504	supt4h1	1566	0.161710037
ensp00000395574	tceb3	1567	0.1618133
ensp00000261900	ccnt1	1568	0.161916563
ensp00000221265	paf1	1572	0.162329616
ensp00000256398	elp3	1573	0.162432879
ensp00000298937	elp4	1574	0.162536142
ensp00000363779	ikbkap	1575	0.162639405
ensp00000374280	rtf1	1576	0.162742668
ensp00000381740	parp3	1577	0.162845931
ensp00000264010	ctcf	1578	0.162949195
ensp00000278612	npat	1579	0.163052458
ensp00000237264	tbpl1	1580	0.163155721
ensp00000283875	gtf2e1	1581	0.163258984
ensp00000298173	gtf2a1	1582	0.163362247
ensp00000379372	gtf2a2	1583	0.16346551
ensp00000379310	casc1	1584	0.163568773
ensp00000337353	tdp1	1586	0.163775299
ensp00000258821	ttc5	1587	0.163878563
ensp00000342105	dyrk2	1588	0.163981826
ensp00000374529	ccnk	1589	0.164085089
ensp00000418287	ciao1	1590	0.164188352
ensp00000387471	fam96b	1591	0.164291615
ensp00000281456	slc25a4	1594	0.164601404
ensp00000361686	vdac2	1597	0.164911194
ensp00000225174	ppif	1598	0.165014457
ensp00000358497	rngtt	1599	0.16511772
ensp00000323421	smc1a	1600	0.165220983
ensp00000340823	gtf2f2	1601	0.165324246
ensp00000314949	polr2a	1603	0.165530772
ensp00000312735	polr2b	1604	0.165634036

ensp00000272645	polr2d	1605	0.165737299
ensp00000219252	polr2c	1606	0.165840562
ensp00000301788	polr2g	1607	0.165943825
ensp00000221859	polr2i	1608	0.166047088
ensp00000292614	polr2j	1609	0.166150351
ensp00000367705	bcor	1612	0.16646014
ensp00000265343	aff4	1613	0.166563404
ensp00000305689	aff1	1614	0.166666667
ensp00000369695	mllt3	1615	0.16676993
ensp00000252674	mllt1	1616	0.166873193
ensp00000381657	dot1l	1617	0.166976456
ensp00000373354	setmar	1618	0.167079719
ensp00000357836	snx27	1619	0.167182982
ensp00000360671	slc25a5	1620	0.167286245
ensp00000370808	slc25a6	1621	0.167389508
ensp00000303754	ppid	1622	0.167492772
ensp00000376328	tesc	1623	0.167596035
ensp00000338348	ube2g2	1624	0.167699298
ensp00000216297	supt16h	1626	0.167905824
ensp00000278412	ssrp1	1627	0.168009087
ensp00000250896	mknk2	1629	0.168215613
ensp00000228741	elk3	1630	0.168318876
ensp00000351137	xab2	1631	0.16842214
ensp00000375809	ercc2	1632	0.168525403
ensp00000228955	gtf2h3	1633	0.168628666
ensp00000285398	ercc3	1634	0.168731929
ensp00000265963	gtf2h1	1635	0.168835192
ensp00000262809	ell	1636	0.168938455
ensp00000299543	ctdp1	1637	0.169041718
ensp00000263377	brd4	1638	0.169144981
ensp00000380695	suds3	1639	0.169248245
ensp00000349748	sfpq	1640	0.169351508
ensp00000276079	nono	1641	0.169454771
ensp00000343966	pspc1	1642	0.169558034
ensp00000310520	ercc4	1645	0.169867823
ensp00000343505	tnfsf13	1646	0.169971086
ensp00000361777	set	1647	0.170074349
ensp00000417864	anp32a	1648	0.170177613
ensp00000215742	thap7	1649	0.170280876
ensp00000349541	ptrf	1650	0.170384139
ensp00000240316	coil	1651	0.170487402
ensp00000340271	taf3	1652	0.170590665
ensp00000362068	taf8	1653	0.170693928
ensp00000358854	taf5	1654	0.170797191
ensp00000355051	taf13	1655	0.170900454
ensp00000354633	taf11	1656	0.171003717
ensp00000348168	gtf2e2	1657	0.171106981

ensp00000296223	polr2h	1658	0.171210244
ensp00000403852	polr2f	1659	0.171313507
ensp00000215587	polr2e	1661	0.171520033
ensp00000361465	polr1c	1662	0.171623296
ensp00000263331	polr1b	1663	0.171726559
ensp00000263857	polr1a	1664	0.171829822
ensp00000302640	ubtf	1665	0.171933086
ensp00000198767	rrn3	1666	0.172036349
ensp00000291688	mcm3ap	1667	0.172139612
ensp00000366390	spdyc	1668	0.172242875
ensp00000335628	spdya	1669	0.172346138
ensp00000302456	klf13	1670	0.172449401
ensp00000327072	taf1a	1671	0.172552664
ensp00000367802	taf1c	1672	0.172655927
ensp00000263663	taf1b	1673	0.17275919
ensp00000314971	taf1d	1674	0.172862454
ensp00000263209	dgcr8	1676	0.17306898
ensp00000357863	gtf3c6	1677	0.173172243
ensp00000400312	ulk3	1679	0.173378769
ensp00000262319	telo2	1680	0.173482032
ensp00000380718	cdyl	1682	0.173688558
ensp00000329167	whsc1	1683	0.173791822
ensp00000257915	tfcp2	1684	0.173895085
ensp00000216106	hmgxb4	1685	0.173998348
ensp00000360163	smarca1	1686	0.174101611
ensp00000307208	bptf	1687	0.174204874
ensp00000398930	sgce	1688	0.174308137
ensp00000266744	ascl1	1689	0.1744114
ensp00000372170	msx1	1690	0.174514663
ensp00000309558	taf15	1691	0.174617926
ensp00000339861	eny2	1692	0.17472119
ensp00000312709	taf7	1693	0.174824453
ensp00000252996	taf4	1694	0.174927716
ensp00000263974	taf12	1695	0.175030979
ensp00000367406	taf2	1697	0.175237505
ensp00000339917	taf9b	1698	0.175340768
ensp00000370201	taf9	1699	0.175444031
ensp00000256996	ddb2	1700	0.175547295
ensp00000364270	xpa	1701	0.175650558
ensp00000318313	ambra1	1702	0.175753821
ensp00000241436	polk	1703	0.175857084
ensp00000242576	ung	1704	0.175960347
ensp00000285021	xpc	1705	0.17606361
ensp00000350708	rad23b	1706	0.176166873
ensp00000321365	rad23a	1707	0.176270136
ensp00000262746	prdx1	1708	0.176373399
ensp00000296417	h2afz	1709	0.176476663

ensp00000324573	flii	1710	0.176579926
ensp00000235372	prdm2	1711	0.176683189
ensp00000356234	kdm5b	1712	0.176786452
ensp00000329029	brf1	1713	0.176889715
ensp00000346697	bmf	1714	0.176992978
ensp00000408994	det1	1715	0.177096241
ensp00000381590	atxn7	1716	0.177199504
ensp00000225396	tada2a	1717	0.177302767
ensp00000258281	taf5l	1719	0.177509294
ensp00000294168	taf6l	1720	0.177612557
ensp00000356848	tada1	1721	0.17771582
ensp00000324122	prpf31	1722	0.177819083
ensp00000411851	c17orf49	1723	0.177922346
ensp00000276072	taf1	1724	0.178025609
ensp00000344537	taf6	1725	0.178128872
ensp00000217893	taf9	1726	0.178232135
ensp00000360515	supt3h	1727	0.178335399
ensp00000336750	supt7l	1728	0.178438662
ensp00000259235	sap130	1729	0.178541925
ensp00000261497	usp22	1730	0.178645188
ensp00000308022	tada2b	1731	0.178748451
ensp00000265038	ercc8	1733	0.178954977
ensp00000384109	cul4b	1734	0.17905824
ensp00000364589	cul4a	1735	0.179161504
ensp00000364037	tex10	1740	0.179677819
ensp00000363124	phf20	1741	0.179781082
ensp00000363944	las1l	1743	0.179987608
ensp00000219905	mga	1744	0.180090872
ensp00000402527	senp6	1746	0.180297398
ensp00000361990	ubr2	1747	0.180400661
ensp00000357799	s100a10	1748	0.180503924
ensp00000314029	senp3	1749	0.180607187
ensp00000221413	ruvbl2	1750	0.18071045
ensp00000318297	ruvbl1	1751	0.180813713
ensp00000347733	trrap	1752	0.180916976
ensp00000307684	tada3	1753	0.18102024
ensp00000358092	prdm1	1754	0.181123503
ensp00000337513	sra1	1755	0.181226766
ensp00000338868	phf8	1756	0.181330029
ensp00000332194	hist2h2ac	1757	0.181433292
ensp00000245479	sox9	1758	0.181536555
ensp00000262518	srcap	1759	0.181639818
ensp00000359531	gtf2b	1760	0.181743081
ensp00000230354	tbp	1761	0.181846344
ensp00000302478	polr1d	1762	0.181949608
ensp00000224073	edf1	1764	0.182156134
ensp00000324444	mbip	1765	0.182259397

ensp00000367203	kdm6a	1767	0.182465923
ensp00000384048	paxip1	1768	0.182569186
ensp00000376204	ash1l	1771	0.182878976
ensp00000346453	ipo4	1772	0.182982239
ensp00000229595	asf1a	1773	0.183085502
ensp00000275780	tlk2	1774	0.183188765
ensp00000370109	psip1	1775	0.183292028
ensp00000316114	ccdc101	1777	0.183498554
ensp00000359290	dr1	1778	0.183601817
ensp00000307850	drap1	1779	0.183705081
ensp00000340896	ash2l	1782	0.18401487
ensp00000351446	wdr5	1783	0.184118133
ensp00000264515	rbbp5	1784	0.184221396
ensp00000406288	chd8	1785	0.184324659
ensp00000356480	rnf2	1787	0.184531185
ensp00000258428	rev1	1788	0.184634449
ensp00000264926	rad18	1789	0.184737712
ensp00000372316	poln	1790	0.184840975
ensp00000217800	poli	1791	0.184944238
ensp00000216133	cbx7	1792	0.185047501
ensp00000251900	scml2	1793	0.185150764
ensp00000384490	cbx6	1794	0.185254027
ensp00000337500	pcgf5	1795	0.18535729
ensp00000269385	cbx8	1796	0.185460553
ensp00000354033	pcgf2	1797	0.185563817
ensp00000354724	pcgf3	1798	0.18566708
ensp00000381840	aebp2	1799	0.185770343
ensp00000404658	ezh1	1800	0.185873606
ensp00000263360	eed	1801	0.185976869
ensp00000320147	ezh2	1802	0.186080132
ensp00000316578	suz12	1803	0.186183395
ensp00000341280	jarid2	1804	0.186286658
ensp00000295066	dpy30	1805	0.186389922
ensp00000390475	cxxc1	1806	0.186493185
ensp00000267197	setd1b	1807	0.186596448
ensp00000262519	setd1a	1808	0.186699711
ensp00000417132	bap1	1809	0.186802974
ensp00000363787	ring1	1810	0.186906237
ensp00000233630	pcgf1	1811	0.1870095
ensp00000358862	pcgf6	1812	0.187112763
ensp00000364839	asxl1	1814	0.18731929
ensp00000361180	gtf3c5	1815	0.187422553
ensp00000361219	gtf3c4	1816	0.187525816
ensp00000263956	gtf3c3	1817	0.187629079
ensp00000264720	gtf3c2	1818	0.187732342
ensp00000348510	gtf3c1	1819	0.187835605
ensp00000308750	cbx2	1821	0.188042131

ensp00000238721	tp53i3	1822	0.188145394
ensp00000202556	ppp1r13b	1824	0.188351921
ensp00000341351	dcaf5	1825	0.188455184
ensp00000231948	crbn	1826	0.188558447
ensp00000355114	dcaf12	1827	0.18866171
ensp00000364404	dcaf17	1828	0.188764973
ensp00000318227	dcaf8	1829	0.188868236
ensp00000298532	snapc4	1831	0.189074762
ensp00000319597	snapc5	1832	0.189178026
ensp00000221573	snapc2	1833	0.189281289
ensp00000265801	lzts1	1835	0.189487815
ensp00000356999	usf1	1836	0.189591078
ensp00000356817	dcaf6	1838	0.189797604
ensp00000371682	dcaf16	1839	0.189900867
ensp00000351147	dcaf4	1840	0.190004131
ensp00000323680	dcaf11	1841	0.190107394
ensp00000282344	usp12	1842	0.190210657
ensp00000348611	phlpp2	1844	0.190417183
ensp00000313171	atad5	1845	0.190520446
ensp00000294053	clpb	1846	0.190623709
ensp00000272995	cops7b	1848	0.190830235
ensp00000350386	chm	1849	0.190933499
ensp00000313869	gorasp1	1850	0.191036762
ensp00000416097	golga2	1851	0.191140025
ensp00000370532	gtf3a	1852	0.191243288
ensp00000353219	nfix	1854	0.191449814
ensp00000384198	ino80d	1855	0.191553077
ensp00000262982	cse1l	1856	0.19165634
ensp00000273480	rnf7	1857	0.191759603
ensp00000346340	cops8	1858	0.191862867
ensp00000347251	gps1	1859	0.19196613
ensp00000264389	cops4	1860	0.192069393
ensp00000229251	cops7a	1861	0.192172656
ensp00000366237	dffa	1862	0.192275919
ensp00000343701	kpna1	1863	0.192379182
ensp00000396127	ran	1864	0.192482445
ensp00000290158	kpnb1	1865	0.192585708
ensp00000261396	nup133	1866	0.192688971
ensp00000342262	nup43	1867	0.192792235
ensp00000243578	b9d2	1868	0.192895498
ensp00000302898	aurkc	1869	0.192998761
ensp00000349486	scrib	1870	0.193102024
ensp00000362937	rcc1	1871	0.193205287
ensp00000365851	bmi1	1873	0.193411813
ensp00000396439	ring1	1874	0.193515076
ensp00000257118	phc2	1875	0.19361834
ensp00000269397	cbx4	1876	0.193721603

ensp00000417980	ehmt1	1877	0.193824866
ensp00000268717	cops3	1878	0.193928129
ensp00000381863	usp19	1879	0.194031392
ensp00000335434	wdr20	1881	0.194237918
ensp00000407818	usp46	1882	0.194341181
ensp00000350512	cops5	1884	0.194547708
ensp00000366953	dcaf10	1886	0.194754234
ensp00000308344	dcaf7	1887	0.194857497
ensp00000252015	trpc4ap	1888	0.19496076
ensp00000312789	dyrk1b	1889	0.195064023
ensp00000341483	ranbp3	1890	0.195167286
ensp00000386992	ipo11	1891	0.195270549
ensp00000266732	tmpo	1892	0.195373812
ensp00000353458	baz1a	1893	0.195477076
ensp00000220913	chrac1	1894	0.195580339
ensp00000351697	rev3l	1895	0.195683602
ensp00000378736	crcp	1896	0.195786865
ensp00000344547	ptma	1897	0.195890128
ensp00000332455	kpna2	1899	0.196096654
ensp00000367721	nup160	1900	0.196199917
ensp00000245544	nup85	1901	0.196303181
ensp00000229179	nup107	1902	0.196406444
ensp00000251074	nup37	1903	0.196509707
ensp00000264670	nsun2	1904	0.19661297
ensp00000216267	brd1	1906	0.196819496
ensp00000373340	brpf1	1907	0.196922759
ensp00000322142	ing5	1908	0.197026022
ensp00000391457	ino80c	1911	0.197335812
ensp00000349640	mcrs1	1912	0.197439075
ensp00000274031	setd7	1914	0.197645601
ensp00000244769	atxn1	1915	0.197748864
ensp00000419494	rybp	1918	0.198058653
ensp00000410994	brd2	1919	0.198161917
ensp00000282903	plod2	1920	0.19826518
ensp00000223127	plod3	1921	0.198368443
ensp00000255613	suv420h2	1923	0.198574969
ensp00000361310	polh	1924	0.198678232
ensp00000265339	ube2b	1925	0.198781495
ensp00000340305	ube2v1	1926	0.198884758
ensp00000258123	usp15	1927	0.198988021
ensp00000392270	strap	1928	0.199091285
ensp00000216330	fkbp3	1929	0.199194548
ensp00000325677	rnf40	1930	0.199297811
ensp00000355205	ino80	1931	0.199401074
ensp00000303977	ino80e	1932	0.199504337
ensp00000336842	actr8	1933	0.1996076
ensp00000415678	pfdn6	1934	0.199710863

ensp00000412319	pfdn6	1935	0.199814126
ensp00000363734	pfdn6	1936	0.19991739
ensp00000332995	setd8	1937	0.200020653
ensp00000346032	anxa2	1938	0.200123916
ensp00000230361	guca1b	1939	0.200227179
ensp00000261047	guca1c	1940	0.200330442
ensp00000272602	cnga3	1943	0.200640231
ensp00000358857	emd	1944	0.200743494
ensp00000290341	igf2bp1	1945	0.200846758
ensp00000374213	ep400	1946	0.200950021
ensp00000272771	tmeff2	1947	0.201053284
ensp00000310094	taok2	1948	0.201156547
ensp00000376317	taok3	1949	0.20125981
ensp00000233331	ino80b	1950	0.201363073
ensp00000243903	actr5	1952	0.201569599
ensp00000400476	nfrkb	1953	0.201672862
ensp00000264108	hat1	1955	0.201879389
ensp00000320566	ing3	1957	0.202085915
ensp00000263062	epc1	1958	0.202189178
ensp00000254900	brd8	1959	0.202292441
ensp00000362166	meaf6	1960	0.202395704
ensp00000226319	phf17	1961	0.202498967
ensp00000350249	pot1	1964	0.202808757
ensp00000267415	tinf2	1965	0.20291202
ensp00000221431	sars2	1968	0.203221809
ensp00000344866	fbxl5	1969	0.203325072
ensp00000346240	fbxo2	1970	0.203428335
ensp00000253023	ube2m	1971	0.203531599
ensp00000354340	uba3	1972	0.203634862
ensp00000290810	nae1	1973	0.203738125
ensp00000370648	rdh14	1974	0.203841388
ensp00000392028	chd7	1975	0.203944651
ensp00000357965	setdb1	1976	0.204047914
ensp00000364858	card16	1977	0.204151177
ensp00000348258	hist1h4l	1978	0.20425444
ensp00000352980	hist1h4a	1979	0.204357703
ensp00000350275	hist1h3a	1980	0.204460967
ensp00000355657	hist3h3	1981	0.20456423
ensp00000350767	hist4h4	1982	0.204667493
ensp00000364894	msh5	1983	0.204770756
ensp00000393963	kifc1	1984	0.204874019
ensp00000306682	ppm1d	1985	0.204977282
ensp00000360613	ube2a	1987	0.205183808
ensp00000373772	rnf20	1988	0.205287071
ensp00000323822	fbxo11	1990	0.205493598
ensp00000307143	donson	1992	0.205700124
ensp00000319248	zeb1	1993	0.205803387

ensp00000397323	ehmt2	1994	0.20590665
ensp00000225603	cbx1	1995	0.206009913
ensp00000269468	mbd1	1996	0.206113176
ensp00000358160	hist1h3h	1997	0.206216439
ensp00000261168	atf7ip	1998	0.206319703
ensp00000281043	mycn	1999	0.206422966
ensp00000309555	hcfc1	2000	0.206526229
ensp00000362824	ogt	2001	0.206629492
ensp00000229812	stk38	2003	0.206836018
ensp00000312697	dmap1	2004	0.206939281
ensp00000247843	yeats4	2005	0.207042544
ensp00000318352	mrfap1	2007	0.207249071
ensp00000353581	hist1h3e	2009	0.207455597
ensp00000276893	uhrf2	2011	0.207662123
ensp00000330074	hist1h1b	2012	0.207765386
ensp00000408176	mutyh	2013	0.207868649
ensp00000282572	ccno	2014	0.207971912
ensp00000300145	xrcc6bp1	2015	0.208075176
ensp00000358531	sycp1	2017	0.208281702
ensp00000293695	syce2	2018	0.208384965
ensp00000250416	parp2	2019	0.208488228
ensp00000355778	h3f3a	2020	0.208591491
ensp00000362441	atrx	2021	0.208694754
ensp00000370936	e2f6	2022	0.208798017
ensp00000301396	pelp1	2023	0.20890128
ensp00000315212	rnf4	2024	0.209004544
ensp00000341282	syce1	2025	0.209107807
ensp00000350162	sycp2	2026	0.20921107
ensp00000265368	syne1	2028	0.209417596
ensp00000350719	syne2	2029	0.209520859
ensp00000266743	sycp3	2030	0.209624122
ensp00000377496	acd	2031	0.209727385
ensp00000367787	lig3	2032	0.209830648
ensp00000398644	nub1	2033	0.209933912
ensp00000358576	dclre1b	2034	0.210037175
ensp00000307298	fem1b	2035	0.210140438
ensp00000357292	ubqln4	2038	0.210450227
ensp00000276603	terf1	2039	0.21055349
ensp00000349313	nhej1	2040	0.210656753
ensp00000299206	poll	2041	0.210760017
ensp00000242248	polm	2042	0.21086328
ensp00000323511	pnkp	2044	0.211069806
ensp00000400806	aptx	2046	0.211276332
ensp00000331327	wt1	2049	0.211586121
ensp00000333873	cib1	2050	0.211689385
ensp00000327054	lmnb2	2051	0.211792648
ensp00000357283	lmna	2052	0.211895911

ensp00000261366	lmnb1	2053	0.211999174
ensp00000300086	terf2ip	2054	0.212102437
ensp00000297338	rad21	2055	0.2122057
ensp00000224862	fbxl15	2057	0.212412226
ensp00000310841	fbxo31	2058	0.212515489
ensp00000339957	usp47	2059	0.212618753
ensp00000380256	ccnf	2060	0.212722016
ensp00000351052	zc3hc1	2061	0.212825279
ensp00000266087	fbxo7	2062	0.212928542
ensp00000384523	nfia	2063	0.213031805
ensp00000396843	nfic	2064	0.213135068
ensp00000243346	nmi	2065	0.213238331
ensp00000003302	usp28	2066	0.213341594
ensp00000338413	uba1	2068	0.213548121
ensp00000366819	uchl3	2069	0.213651384
ensp00000320898	rnf168	2070	0.213754647
ensp00000254942	terf2	2071	0.21385791
ensp00000354720	smc3	2073	0.214064436
ensp00000218089	stag2	2074	0.214167699
ensp00000372689	stag1	2075	0.214270962
ensp00000313851	pds5b	2076	0.214374226
ensp00000303427	pds5a	2077	0.214477489
ensp00000298767	wapal	2078	0.214580752
ensp00000319318	stag3	2079	0.214684015
ensp00000357669	npr1	2081	0.214890541
ensp00000342011	xrcc4	2083	0.215097067
ensp00000367527	dclre1c	2084	0.21520033
ensp00000251547	fbxo44	2085	0.215303594
ensp00000292853	fbxo27	2086	0.215406857
ensp00000365944	fbxo6	2087	0.21551012
ensp00000281623	fbxo4	2088	0.215613383
ensp00000310686	fbxw8	2089	0.215716646
ensp00000357260	pmf1	2090	0.215819909
ensp00000370557	mis12	2091	0.215923172
ensp00000363055	zwint	2092	0.216026435
ensp00000257934	espl1	2093	0.216129698
ensp00000346130	svip	2095	0.216336225
ensp00000216879	nsfl1c	2097	0.216542751
ensp00000375921	pax3	2099	0.216749277
ensp00000267890	ttbk2	2100	0.21685254
ensp00000321507	azin1	2101	0.216955803
ensp00000350633	sertad1	2102	0.217059067
ensp00000366249	ubd	2106	0.217472119
ensp00000361446	polr3a	2107	0.217575382
ensp00000228347	polr3b	2108	0.217678645
ensp00000347345	polr3h	2109	0.217781908
ensp00000293860	polr3k	2110	0.217885171

ensp00000299853	polr3e	2111	0.217988435
ensp00000366828	polr3f	2112	0.218091698
ensp00000358262	chd1l	2113	0.218194961
ensp00000298139	wrn	2114	0.218298224
ensp00000343741	atr	2116	0.21850475
ensp00000369855	asb9	2117	0.218608013
ensp00000355011	ilf2	2119	0.218814539
ensp00000313420	prkdc	2120	0.218917803
ensp00000364310	h2afx	2121	0.219021066
ensp00000265433	nbn	2122	0.219124329
ensp00000362578	rnf8	2123	0.219227592
ensp00000373060	mdc1	2124	0.219330855
ensp00000259008	brip1	2125	0.219434118
ensp00000376903	banp	2126	0.219537381
ensp00000209884	klhl20	2127	0.219640644
ensp00000343273	klhl7	2128	0.219743907
ensp00000343526	usp1	2129	0.219847171
ensp00000262630	zbtb32	2130	0.219950434
ensp00000267430	fancm	2131	0.220053697
ensp00000254262	c19orf40	2132	0.22015696
ensp00000285679	usp25	2134	0.220363486
ensp00000351933	klhl9	2135	0.220466749
ensp00000262820	klhl13	2136	0.220570012
ensp00000331682	klhl22	2137	0.220673276
ensp00000312397	klhl3	2138	0.220776539
ensp00000342924	mcph1	2139	0.220879802
ensp00000261584	palb2	2140	0.220983065
ensp00000358151	hist2h2be	2141	0.221086328
ensp00000316176	ube2n	2143	0.221292854
ensp00000369857	fam175a	2144	0.221396117
ensp00000298492	fam175b	2145	0.22149938
ensp00000365938	uggt2	2146	0.221602644
ensp00000226225	tnfaip1	2147	0.221705907
ensp00000311202	kctd13	2148	0.22180917
ensp00000291900	zer1	2149	0.221912433
ensp00000320675	asb2	2151	0.222118959
ensp00000410088	ncaph2	2152	0.222222222
ensp00000261609	herc2	2154	0.222428748
ensp00000368174	mcm8	2155	0.222532012
ensp00000401018	gins3	2156	0.222635275
ensp00000352222	ctps2	2157	0.222738538
ensp00000216294	snapc1	2159	0.222945064
ensp00000271411	pou2f1	2160	0.223048327
ensp00000311513	rsf1	2161	0.22315159
ensp00000283131	smarca5	2163	0.223358116
ensp00000362592	rbbp4	2164	0.22346138
ensp00000369427	rbbp7	2165	0.223564643

ensp00000271002	itgb3bp	2167	0.223771169
ensp00000351284	rad52	2168	0.223874432
ensp00000340879	rad1	2169	0.223977695
ensp00000366876	phf13	2174	0.224494011
ensp00000356230	klhl12	2175	0.224597274
ensp00000248566	shfm1	2176	0.224700537
ensp00000355961	ints7	2177	0.2248038
ensp00000317110	cort	2178	0.224907063
ensp00000302951	stra13	2179	0.225010326
ensp00000317039	rmi1	2180	0.225113589
ensp00000354856	clk2	2181	0.225216853
ensp00000326830	clk1	2182	0.225320116
ensp00000265081	msh3	2183	0.225423379
ensp00000406490	pms1	2184	0.225526642
ensp00000265849	pms2	2185	0.225629905
ensp00000326819	fancb	2187	0.225836431
ensp00000333283	c17orf70	2188	0.225939694
ensp00000385021	fancl	2189	0.226042957
ensp00000330875	fancf	2190	0.226146221
ensp00000229769	fance	2191	0.226249484
ensp00000287647	fancd2	2192	0.226352747
ensp00000349823	smarcal1	2193	0.22645601
ensp00000343392	xrcc3	2194	0.226559273
ensp00000348020	mlh3	2195	0.226662536
ensp00000321636	top3a	2196	0.226765799
ensp00000377384	psmc3ip	2198	0.226972325
ensp00000336701	rad51c	2199	0.227075589
ensp00000256246	tex15	2200	0.227178852
ensp00000325863	mre11a	2203	0.227488641
ensp00000260810	topbp1	2204	0.227591904
ensp00000370151	rad17	2205	0.227695167
ensp00000261881	tipin	2206	0.22779843
ensp00000244661	hist1h3b	2207	0.227901694
ensp00000363021	rpa2	2208	0.228004957
ensp00000223129	rpa3	2209	0.22810822
ensp00000347232	blm	2210	0.228211483
ensp00000231790	mlh1	2211	0.228314746
ensp00000254719	rpa1	2212	0.228418009
ensp00000315700	chaf1b	2213	0.228521272
ensp00000301280	chaf1a	2214	0.228624535
ensp00000253024	trim28	2215	0.228727798
ensp00000321239	rchy1	2216	0.228831062
ensp00000369497	brca2	2217	0.228934325
ensp00000367910	fancg	2218	0.229037588
ensp00000373952	fanca	2219	0.229140851
ensp00000289081	fancc	2220	0.229244114
ensp00000296930	npm1	2221	0.229347377

ensp00000298687	ndrg2	2222	0.22945064
ensp00000303088	polr3d	2223	0.229553903
ensp00000382058	polr3g	2224	0.229657166
ensp00000358320	polr3gl	2225	0.22976043
ensp00000416599	znrd1	2226	0.229863693
ensp00000367029	polr1e	2229	0.230173482
ensp00000378890	gch1	2231	0.230380008
ensp00000013034	nme1	2232	0.230483271
ensp00000416658	nme6	2233	0.230586534
ensp00000265191	nme5	2234	0.230689798
ensp00000356785	nme7	2235	0.230793061
ensp00000393953	pnpt1	2237	0.230999587
ensp00000371230	ak3	2238	0.23110285
ensp00000284811	tceb1	2239	0.231206113
ensp00000262306	tceb2	2240	0.231309376
ensp00000325919	psmg2	2242	0.231515903
ensp00000292616	lrwd1	2243	0.231619166
ensp00000362131	rpa4	2244	0.231722429
ensp00000323099	atrip	2245	0.231825692
ensp00000258774	hus1	2246	0.231928955
ensp00000311360	rad9a	2247	0.232032218
ensp00000055077	rfc2	2248	0.232135481
ensp00000369411	rfc3	2249	0.232238744
ensp00000382133	dna2	2250	0.232342007
ensp00000263382	asf1b	2251	0.232445271
ensp00000356243	ube2t	2252	0.232548534
ensp00000310842	fanci	2253	0.232651797
ensp00000281453	mlf1ip	2254	0.23275506
ensp00000335463	casc5	2255	0.232858323
ensp00000283006	cenph	2256	0.232961586
ensp00000364737	cenpp	2257	0.233064849
ensp00000337289	cenpq	2258	0.233168112
ensp00000260662	cenpo	2259	0.233271375
ensp00000218507	cenpi	2260	0.233374639
ensp00000242872	cenpk	2261	0.233477902
ensp00000336868	cenpa	2262	0.233581165
ensp00000377007	cenpn	2263	0.233684428
ensp00000220514	oip5	2264	0.233787691
ensp00000414109	hjurp	2265	0.233890954
ensp00000378356	kif20a	2266	0.233994217
ensp00000300403	tpx2	2267	0.23409748
ensp00000260359	nusap1	2268	0.234200743
ensp00000228843	rad51ap1	2269	0.234304007
ensp00000265748	anln	2270	0.23440727
ensp00000356319	kif14	2271	0.234510533
ensp00000240488	mnd1	2272	0.234613796
ensp00000311873	exo1	2273	0.234717059

ensp00000355958	dtl	2274	0.234820322
ensp00000275517	cdca5	2275	0.234923585
ensp00000300035	kiaa0101	2276	0.235026848
ensp00000348657	ncapg2	2277	0.235130112
ensp00000287394	atad2	2278	0.235233375
ensp00000260753	kif20b	2279	0.235336638
ensp00000262315	chtf18	2280	0.235439901
ensp00000263274	lig1	2281	0.235543164
ensp00000307288	mcm7	2282	0.235646427
ensp00000264156	mcm6	2283	0.23574969
ensp00000262105	mcm4	2284	0.235852953
ensp00000229854	mcm3	2285	0.235956216
ensp00000216122	mcm5	2286	0.23605948
ensp00000265056	mcm2	2287	0.236162743
ensp00000305480	fen1	2288	0.236266006
ensp00000286398	smc2	2289	0.236369269
ensp00000341382	smc4	2290	0.236472532
ensp00000353793	wdhd1	2291	0.236575795
ensp00000216367	pole2	2292	0.236679058
ensp00000263681	pold3	2294	0.236885584
ensp00000363284	pole3	2295	0.236988848
ensp00000420176	pole4	2296	0.237092111
ensp00000311368	pold4	2298	0.237298637
ensp00000261424	rfc1	2299	0.2374019
ensp00000262460	gins1	2300	0.237505163
ensp00000276533	gins4	2301	0.237608426
ensp00000221486	rnaseh2a	2302	0.237711689
ensp00000325017	ncapd2	2303	0.237814952
ensp00000265148	cenpe	2305	0.238021479
ensp00000282074	spc25	2306	0.238124742
ensp00000355922	cenpf	2307	0.238228005
ensp00000261597	ndc80	2308	0.238331268
ensp00000316121	cdca8	2309	0.238434531
ensp00000311429	zwilch	2310	0.238537794
ensp00000334675	ercc6l	2311	0.238641057
ensp00000234420	msh6	2312	0.238744321
ensp00000233146	msh2	2313	0.238847584
ensp00000355153	cdkn2a	2314	0.238950847
ensp00000263433	ppp1r12c	2315	0.23905411
ensp00000216714	apex1	2316	0.239157373
ensp00000262887	xrcc1	2317	0.239260636
ensp00000359356	evi5	2318	0.239363899
ensp00000359790	dst	2319	0.239467162
ensp00000322180	dscc1	2320	0.239570425
ensp00000217429	fam83d	2323	0.239880215
ensp00000257909	troap	2324	0.239983478
ensp00000265728	dbf4	2326	0.240190004

ensp00000246032	stk35	2330	0.240603057
ensp00000363524	kif4a	2331	0.24070632
ensp00000375705	ahctf1	2332	0.240809583
ensp00000348527	cenpl	2333	0.240912846
ensp00000397131	spc24	2334	0.241016109
ensp00000261716	taok1	2335	0.241119372
ensp00000263753	sgol1	2336	0.241222635
ensp00000350447	sgol2	2337	0.241325898
ensp00000296509	mad2l1	2338	0.241429162
ensp00000301905	pbk	2339	0.241532425
ensp00000335357	cdkn3	2340	0.241635688
ensp00000298048	melk	2341	0.241738951
ensp00000323300	spag5	2342	0.241842214
ensp00000253462	gins2	2343	0.241945477
ensp00000229201	timeless	2344	0.24204874
ensp00000312995	clspn	2345	0.242152003
ensp00000355759	parp1	2346	0.242255266
ensp00000368438	pcna	2347	0.24235853
ensp00000350491	prim1	2348	0.242461793
ensp00000368349	pola1	2349	0.242565056
ensp00000265465	pola2	2351	0.242771582
ensp00000322570	pole	2352	0.242874845
ensp00000344874	gucy1a2	2353	0.242978108
ensp00000264424	gucy1b3	2354	0.243081371
ensp00000296518	gucy1a3	2355	0.243184634
ensp00000385000	kif2a	2357	0.243391161
ensp00000215980	cenpm	2358	0.243494424
ensp00000361298	kif2c	2359	0.243597687
ensp00000301633	birc5	2360	0.24370095
ensp00000328236	kntc1	2361	0.243804213
ensp00000263181	kif18a	2362	0.243907476
ensp00000200135	zw10	2363	0.244010739
ensp00000319417	ska3	2364	0.244114002
ensp00000328228	cdca2	2365	0.244217266
ensp00000239027	hells	2366	0.244320529
ensp00000361043	rad54l	2368	0.244527055
ensp00000229265	cdca3	2370	0.244733581
ensp00000166345	trip13	2371	0.244836844
ensp00000234626	cdc7	2373	0.245043371
ensp00000209728	cdc6	2374	0.245146634
ensp00000266970	cdk2	2375	0.245249897
ensp00000350283	brca1	2376	0.24535316
ensp00000278616	atm	2377	0.245456423
ensp00000267868	rad51	2378	0.245559686
ensp00000323050	rbbp8	2379	0.245662949
ensp00000306968	cdca7	2380	0.245766212
ensp00000301634	tk1	2381	0.245869475

ensp00000315644	tyms	2382	0.245972739
ensp00000278916	chek1	2383	0.246076002
ensp00000408295	rfc5	2384	0.246179265
ensp00000296273	rfc4	2385	0.246282528
ensp00000271452	nuf2	2386	0.246385791
ensp00000302530	bub1	2387	0.246489054
ensp00000247191	dlgap5	2388	0.246592317
ensp00000274026	ccna2	2389	0.24669558
ensp00000411532	top2a	2390	0.246798843
ensp00000302955	rrm2	2391	0.246902107
ensp00000300738	rrm1	2392	0.24700537
ensp00000357643	mki67	2393	0.247108633
ensp00000240423	ncaph	2394	0.247211896
ensp00000377793	prc1	2395	0.247315159
ensp00000306473	shcbp1	2396	0.247418422
ensp00000367276	ckap2	2397	0.247521685
ensp00000264596	neil3	2399	0.247728211
ensp00000355090	arhgap11a	2400	0.247831475
ensp00000364976	cks2	2402	0.248038001
ensp00000286800	bach1	2403	0.248141264
ensp00000360944	stil	2405	0.24834779
ensp00000305204	ckap2l	2406	0.248451053
ensp00000324020	kif15	2407	0.248554316
ensp00000230510	ttk	2408	0.24865758
ensp00000356379	aspm	2409	0.248760843
ensp00000251496	ncapg	2410	0.248864106
ensp00000360540	cep55	2411	0.248967369
ensp00000377492	hmmr	2412	0.249070632
ensp00000260731	kif11	2413	0.249173895
ensp00000260363	kif23	2414	0.249277158
ensp00000309871	racgap1	2415	0.249380421
ensp00000229758	fbxo5	2416	0.249483684
ensp00000230056	gmnn	2420	0.249896737
ensp00000301019	cdt1	2421	0.25
ensp00000255465	ccna1	2422	0.250103263
ensp00000382562	mad1l1	2423	0.250206526
ensp00000219172	cenpt	2425	0.250413052
ensp00000362850	dsn1	2426	0.250516316
ensp00000355944	nsl1	2427	0.250619579
ensp00000319664	nudc	2428	0.250722842
ensp00000364582	rcc2	2429	0.250826105
ensp00000217026	mybl2	2431	0.251032631
ensp00000398124	e2f5	2432	0.251135894
ensp00000309622	tfdp2	2433	0.251239157
ensp00000364519	tfdp1	2434	0.25134242
ensp00000345571	e2f1	2435	0.251445684
ensp00000209875	cbx5	2436	0.251548947

ensp00000336687	cbx3	2437	0.25165221
ensp00000347213	entpd2	2439	0.251858736
ensp00000362282	mocs1	2440	0.251961999
ensp00000341083	npr2	2444	0.252375052
ensp00000370376	dut	2445	0.252478315
ensp00000265421	polb	2446	0.252581578
ensp00000333433	ska2	2447	0.252684841
ensp00000285116	ska1	2448	0.252788104
ensp00000378295	incenp	2449	0.252891367
ensp00000333982	ndel1	2450	0.25299463
ensp00000381932	dyrk1a	2451	0.253097893
ensp00000341947	lin54	2454	0.253407683
ensp00000329102	lin9	2455	0.253510946
ensp00000270861	plk4	2456	0.253614209
ensp00000355966	nek2	2457	0.253717472
ensp00000256442	ccnb1	2459	0.253923998
ensp00000288207	ccnb2	2460	0.254027261
ensp00000276014	ccnb3	2461	0.254130525
ensp00000235310	mad2l2	2462	0.254233788
ensp00000260947	bard1	2464	0.254440314
ensp00000372023	chek2	2465	0.254543577
ensp00000311083	cks1b	2466	0.25464684
ensp00000244573	hist1h1a	2468	0.254853366
ensp00000368730	pde7a	2469	0.254956629
ensp00000311453	pde8a	2470	0.255059893
ensp00000286063	pde11a	2471	0.255163156
ensp00000326550	tacc3	2472	0.255266419
ensp00000342307	foxm1	2473	0.255369682
ensp00000261313	pebp1	2474	0.255472945
ensp00000303706	cdc25a	2475	0.255576208
ensp00000402084	wee1	2476	0.255679471
ensp00000323246	e2f7	2477	0.255782734
ensp00000319778	aurkaip1	2478	0.255885998
ensp00000372721	psmb9	2479	0.255989261
ensp00000406797	psmb8	2480	0.256092524
ensp00000313922	oaz3	2482	0.25629905
ensp00000314813	oaz1	2483	0.256402313
ensp00000261817	psmd9	2484	0.256505576
ensp00000351314	psmb10	2488	0.256918629
ensp00000357858	bub3	2489	0.257021892
ensp00000300093	plk1	2490	0.257125155
ensp00000380378	pafah1b1	2491	0.257228418
ensp00000263710	clasp1	2492	0.257331681
ensp00000352581	clasp2	2493	0.257434944
ensp00000260746	arl3	2494	0.257538207
ensp00000295901	psmd6	2496	0.257744734
ensp00000348442	psmd12	2497	0.257847997

ensp00000292644	psmc2	2498	0.25795126
ensp00000044462	psma4	2501	0.258261049
ensp00000365233	tpp2	2504	0.258570838
ensp00000370222	pomp	2505	0.258674102
ensp00000278935	cep164	2506	0.258777365
ensp00000359300	cetn2	2507	0.258880628
ensp00000345892	nde1	2508	0.258983891
ensp00000364721	mapre1	2509	0.259087154
ensp00000346566	ckap5	2510	0.259190417
ensp00000313950	aurkb	2511	0.25929368
ensp00000216911	aurka	2512	0.259396943
ensp00000386212	psmb11	2513	0.259500207
ensp00000384211	psme4	2514	0.25960347
ensp00000362334	psmb2	2515	0.259706733
ensp00000225426	psmb3	2516	0.259809996
ensp00000259457	psmb7	2517	0.259913259
ensp00000261479	psma6	2519	0.260119785
ensp00000264639	psmd3	2520	0.260223048
ensp00000309474	psmd1	2521	0.260326311
ensp00000396937	psmd13	2522	0.260429575
ensp00000210313	psmd5	2523	0.260532838
ensp00000263413	c6	2525	0.260739364
ensp00000306340	cdkl2	2526	0.260842627
ensp00000250092	cd68	2527	0.26094589
ensp00000216802	psme2	2528	0.261049153
ensp00000414187	cpeb1	2529	0.261152416
ensp00000280154	pdcd4	2530	0.261255679
ensp00000261714	blmh	2531	0.261358943
ensp00000253003	adrm1	2533	0.261565469
ensp00000304697	ubb	2536	0.261875258
ensp00000356425	uchl5	2537	0.261978521
ensp00000313811	usp20	2538	0.262081784
ensp00000393087	hspa1b	2539	0.262185048
ensp00000344259	ube2l3	2540	0.262288311
ensp00000303709	ube2e1	2541	0.262391574
ensp00000327704	psmf1	2542	0.262494837
ensp00000310129	psmd2	2543	0.2625981
ensp00000261303	psmc1	2544	0.262701363
ensp00000157812	psmc4	2545	0.262804626
ensp00000344936	pttg1	2546	0.262907889
ensp00000287598	bub1b	2547	0.263011152
ensp00000308450	cdc20	2548	0.263114416
ensp00000348838	ube2c	2549	0.263217679
ensp00000216455	psma3	2550	0.263320942
ensp00000293362	psme3	2551	0.263424205
ensp00000372155	psme1	2552	0.263527468
ensp00000261712	psmd11	2553	0.263630731

ensp00000386541	psmd14	2555	0.263837257
ensp00000261601	usp14	2556	0.26394052
ensp00000378529	fzr1	2557	0.264043784
ensp00000228872	cdkn1b	2558	0.264147047
ensp00000244741	cdkn1a	2559	0.26425031
ensp00000274255	skp2	2560	0.264353573
ensp00000356641	rfwd2	2561	0.264456836
ensp00000363641	txn	2563	0.264663362
ensp00000343535	usp7	2564	0.264766625
ensp00000339109	anapc1	2565	0.264869888
ensp00000363322	cdc26	2566	0.264973152
ensp00000261819	anapc5	2567	0.265076415
ensp00000346987	anapc13	2569	0.265282941
ensp00000251413	tubg1	2571	0.265489467
ensp00000348554	cdc16	2572	0.26559273
ensp00000394394	anapc7	2574	0.265799257
ensp00000231487	skp1	2575	0.26590252
ensp00000326804	cul1	2576	0.266005783
ensp00000216225	rbx1	2577	0.266109046
ensp00000264414	cul3	2578	0.266212309
ensp00000371419	parp4	2580	0.266418835
ensp00000317214	capn6	2581	0.266522098
ensp00000311665	paaf1	2583	0.266728625
ensp00000378350	cdc23	2584	0.266831888
ensp00000318775	anapc4	2585	0.266935151
ensp00000314004	anapc2	2586	0.267038414
ensp00000310071	anapc10	2587	0.267141677
ensp00000343412	bre	2590	0.267451466
ensp00000381998	brcc3	2591	0.267554729
ensp00000264552	ube2s	2592	0.267657993
ensp00000265348	cul7	2593	0.267761256
ensp00000364389	cdc14b	2595	0.267967782
ensp00000291295	calm3	2596	0.268071045
ensp00000217964	tbl1x	2597	0.268174308
ensp00000272298	calm2	2598	0.268277571
ensp00000250457	egln3	2599	0.268380834
ensp00000355601	egln1	2600	0.268484097
ensp00000307080	egln2	2601	0.268587361
ensp00000281708	fbxw7	2602	0.268690624
ensp00000347834	fbxl3	2603	0.268793887
ensp00000359206	btrc	2604	0.26889715
ensp00000265094	fbxw11	2605	0.269000413
ensp00000215574	cdc34	2606	0.269103676
ensp00000262643	ccne1	2607	0.269206939
ensp00000309181	ccne2	2608	0.269310202
ensp00000365877	suv39h1	2609	0.269413466
ensp00000265734	cdk6	2610	0.269516729

ensp00000362768	rbl1	2611	0.269619992
ensp00000368686	e2f4	2612	0.269723255
ensp00000262133	rbl2	2613	0.269826518
ensp00000413720	cdkn1c	2614	0.269929781
ensp00000342889	polr2k	2615	0.270033044
ensp00000360561	entpd8	2616	0.270136307
ensp00000301825	entpd3	2617	0.27023957
ensp00000369456	itpa	2619	0.270446097
ensp00000317659	guk1	2620	0.27054936
ensp00000366898	hif3a	2621	0.270652623
ensp00000256474	vhl	2622	0.270755886
ensp00000363880	cul2	2623	0.270859149
ensp00000348577	rangap1	2625	0.271065675
ensp00000327583	ranbp1	2626	0.271168938
ensp00000299759	rrad	2627	0.271272202
ensp00000403293	fbxo43	2628	0.271375465
ensp00000300835	prr14	2629	0.271478728
ensp00000377280	fbxo8	2630	0.271581991
ensp00000251810	rrm2b	2631	0.271685254
ensp00000346577	ak5	2632	0.271788517
ensp00000360939	cmpk1	2633	0.27189178
ensp00000256722	cmpk2	2634	0.271995043
ensp00000304802	dtymk	2635	0.272098306
ensp00000248553	hspb1	2636	0.27220157
ensp00000359910	psma7	2638	0.272408096
ensp00000298852	psmc3	2639	0.272511359
ensp00000401802	psmc6	2640	0.272614622
ensp00000310572	psmc5	2641	0.272717885
ensp00000217958	psmd10	2642	0.272821148
ensp00000295926	ccnl1	2643	0.272924411
ensp00000410076	casp1	2644	0.273027675
ensp00000388566	casp4	2645	0.273130938
ensp00000299697	tk2	2646	0.273234201
ensp00000349576	dctd	2647	0.273337464
ensp00000229335	aicda	2648	0.273440727
ensp00000270225	sae1	2649	0.27354399
ensp00000246548	uba2	2650	0.273647253
ensp00000304226	hipk3	2651	0.273750516
ensp00000351901	card8	2652	0.273853779
ensp00000264093	dguok	2654	0.274060306
ensp00000357255	bglap	2655	0.274163569
ensp00000300302	herpud1	2656	0.274266832
ensp00000259512	derl1	2657	0.274370095
ensp00000337053	sel1l	2658	0.274473358
ensp00000318165	os9	2659	0.274576621
ensp00000361289	uck1	2660	0.274679884
ensp00000394791	senp1	2661	0.274783147

ensp00000272430	rtkn	2662	0.274886411
ensp00000363836	ccny	2663	0.274989674
ensp00000357748	bccip	2664	0.275092937
ensp00000356853	uck2	2666	0.275299463
ensp00000286648	dck	2667	0.275402726
ensp00000256578	ampd2	2671	0.275815779
ensp00000259727	gmpr	2672	0.275919042
ensp00000345096	impdh1	2673	0.276022305
ensp00000362481	uprt	2675	0.276228831
ensp00000351170	gda	2677	0.276435357
ensp00000312304	tpmt	2678	0.27653862
ensp00000352516	dnmt1	2679	0.276641884
ensp00000328547	dnmt3b	2680	0.276745147
ensp00000264709	dnmt3a	2681	0.27684841
ensp00000299964	nnmt	2682	0.276951673
ensp00000232607	umps	2683	0.277054936
ensp00000387230	upp2	2684	0.277158199
ensp00000252029	tymp	2685	0.277261462
ensp00000310661	pde7b	2686	0.277364725
ensp00000334910	pde2a	2687	0.277467988
ensp00000243052	pde1b	2688	0.277571252
ensp00000331574	pde1a	2689	0.277674515
ensp00000379485	pde1c	2690	0.277777778
ensp00000373674	nt5m	2691	0.277881041
ensp00000245552	nt5c	2692	0.277984304
ensp00000339479	nt5c2	2695	0.278294093
ensp00000392859	gmpr2	2696	0.278397356
ensp00000380024	ing4	2697	0.27850062
ensp00000358323	txnip	2699	0.278707146
ensp00000332448	adat3	2700	0.278810409
ensp00000361965	ada	2701	0.278913672
ensp00000265440	tfec	2702	0.279016935
ensp00000382595	paics	2703	0.279120198
ensp00000419851	gmps	2704	0.279223461
ensp00000257770	nt5e	2706	0.279429988
ensp00000335246	entpd5	2707	0.279533251
ensp00000307674	cant1	2708	0.279636514
ensp00000365840	entpd6	2709	0.279739777
ensp00000351520	entpd4	2710	0.27984304
ensp00000298556	hprt1	2711	0.279946303
ensp00000347689	pde4c	2713	0.280152829
ensp00000332116	pde4b	2714	0.280256093
ensp00000384806	pde4d	2715	0.280359356
ensp00000270474	pde4a	2716	0.280462619
ensp00000282096	pde3b	2717	0.280565882
ensp00000362249	ak1	2719	0.280772408
ensp00000346921	ak2	2720	0.280875671

ensp00000267584	ak7	2722	0.281082197
ensp00000420295	pde6b	2724	0.281288724
ensp00000255266	pde6a	2725	0.281391987
ensp00000251102	cngb1	2727	0.281598513
ensp00000330032	upp1	2728	0.281701776
ensp00000359211	dpyd	2729	0.281805039
ensp00000306894	etv5	2730	0.281908302
ensp00000378782	qprt	2733	0.282218092
ensp00000401508	naprt1	2735	0.282424618
ensp00000364212	cda	2737	0.282631144
ensp00000304782	ces3	2738	0.282734407
ensp00000353720	ces1	2740	0.282940933
ensp00000337383	nlrp3	2741	0.283044197
ensp00000222308	fkbp8	2743	0.283250723
ensp00000291539	pde9a	2744	0.283353986
ensp00000347046	pde5a	2746	0.283560512
ensp00000287600	pde6d	2747	0.283663775
ensp00000219479	nme4	2750	0.283973565
ensp00000339933	pklr	2751	0.284076828
ensp00000262238	yy1	2754	0.284386617
ensp00000397552	actl6a	2755	0.28448988
ensp00000340330	kat5	2756	0.284593143
ensp00000294702	gfi1	2758	0.28479967
ensp00000276925	cdkn2b	2759	0.284902933
ensp00000267163	rb1	2760	0.285006196
ensp00000227507	ccnd1	2761	0.285109459
ensp00000257904	cdk4	2762	0.285212722
ensp00000261254	ccnd2	2763	0.285315985
ensp00000337056	cdkn2d	2764	0.285419248
ensp00000359385	glmn	2767	0.285729038
ensp00000373884	ryr3	2768	0.285832301
ensp00000342667	rnf19a	2769	0.285935564
ensp00000283195	ranbp2	2771	0.28614209
ensp00000375080	ppp2r3b	2772	0.286245353
ensp00000286353	acpl2	2774	0.286451879
ensp00000287713	nmnat2	2775	0.286555143
ensp00000340523	nmnat3	2776	0.286658406
ensp00000230792	nudt12	2777	0.286761669
ensp00000341679	nadk	2778	0.286864932
ensp00000264663	nnt	2779	0.286968195
ensp00000216194	adsl	2780	0.287071458
ensp00000236959	atic	2781	0.287174721
ensp00000321584	impdh2	2782	0.287277984
ensp00000228495	kctd10	2783	0.287381247
ensp00000352324	atxn3	2784	0.287484511
ensp00000365576	ubqln1	2785	0.287587774
ensp00000345195	ubqln2	2786	0.287691037

ensp00000343001	ube4b	2787	0.2877943
ensp00000282058	haus1	2788	0.287897563
ensp00000260372	haus2	2789	0.288000826
ensp00000371308	cenpj	2790	0.288104089
ensp00000260383	tubgcp4	2791	0.288207352
ensp00000379931	ube2e2	2794	0.288517142
ensp00000381717	ube2d2	2796	0.288723668
ensp00000349722	ube2d3	2797	0.288826931
ensp00000364235	rnf5	2798	0.288930194
ensp00000290649	amfr	2799	0.289033457
ensp00000351777	vcp	2800	0.28913672
ensp00000342510	cep97	2802	0.289343247
ensp00000308021	cep290	2803	0.28944651
ensp00000317156	cep192	2805	0.289653036
ensp00000344314	ofd1	2806	0.289756299
ensp00000313752	ssna1	2807	0.289859562
ensp00000278886	ninl	2808	0.289962825
ensp00000355499	sdccag8	2809	0.290066088
ensp00000257287	cep135	2812	0.290375878
ensp00000369127	dnaja1	2813	0.290479141
ensp00000276570	dnajc5b	2815	0.290685667
ensp00000406751	cry2	2816	0.29078893
ensp00000008527	cry1	2817	0.290892193
ensp00000254657	per2	2818	0.290995456
ensp00000222402	ube2d4	2819	0.29109872
ensp00000409151	hspa1l	2820	0.291201983
ensp00000310219	hspa6	2821	0.291305246
ensp00000247207	hspa2	2822	0.291408509
ensp00000324897	ube2i	2823	0.291511772
ensp00000405965	sumo2	2824	0.291615035
ensp00000330343	sumo3	2825	0.291718298
ensp00000262127	cep76	2826	0.291821561
ensp00000336524	cep63	2827	0.291924824
ensp00000264935	cep72	2828	0.292028088
ensp00000407964	nedd1	2829	0.292131351
ensp00000252936	tubgcp2	2830	0.292234614
ensp00000283645	tubgcp5	2832	0.29244114
ensp00000264448	alms1	2833	0.292544403
ensp00000365782	cep78	2835	0.292750929
ensp00000218758	acp5	2837	0.292957456
ensp00000273130	dync1li1	2838	0.293060719
ensp00000348965	dync1h1	2839	0.293163982
ensp00000380308	dync1i2	2840	0.293267245
ensp00000259632	dctn3	2841	0.293370508
ensp00000317902	cep57	2843	0.293577034
ensp00000382271	cep152	2844	0.293680297
ensp00000254605	rrp8	2845	0.293783561

ensp00000338019	dnajb2	2847	0.293990087
ensp00000345575	dnajb12	2848	0.29409335
ensp00000314030	dnaja2	2849	0.294196613
ensp00000255631	hspbp1	2850	0.294299876
ensp00000270593	acpt	2851	0.294403139
ensp00000292180	flad1	2852	0.294506402
ensp00000354238	enpp1	2853	0.294609665
ensp00000350265	enpp3	2854	0.294712929
ensp00000311766	atad3b	2855	0.294816192
ensp00000381461	ppme1	2856	0.294919455
ensp00000355979	sertad4	2857	0.295022718
ensp00000263620	arid3a	2859	0.295229244
ensp00000322542	gtf2i	2860	0.295332507
ensp00000358770	slk	2861	0.29543577
ensp00000354522	top1	2863	0.295642297
ensp00000341024	satb1	2864	0.29574556
ensp00000347602	arid4a	2865	0.295848823
ensp00000276461	erlin2	2866	0.295952086
ensp00000358081	bag3	2867	0.296055349
ensp00000237937	zfand5	2868	0.296158612
ensp00000358241	acp6	2870	0.296365138
ensp00000326424	nadsyn1	2871	0.296468401
ensp00000265016	bst1	2872	0.296571665
ensp00000328269	hmg20b	2875	0.296881454
ensp00000340019	hspd1	2876	0.296984717
ensp00000225916	kat2a	2877	0.29708798
ensp00000263754	kat2b	2878	0.297191243
ensp00000261531	snw1	2879	0.297294506
ensp00000339723	cir1	2880	0.29739777
ensp00000366410	nmnat1	2881	0.297501033
ensp00000408907	hspa1a	2882	0.297604296
ensp00000355031	per3	2883	0.297707559
ensp00000314420	per1	2884	0.297810822
ensp00000280614	ccrn4l	2885	0.297914085
ensp00000372322	zmym2	2886	0.298017348
ensp00000396052	brms1	2887	0.298120611
ensp00000304169	pitx2	2890	0.298430401
ensp00000233121	mapre3	2891	0.298533664
ensp00000383145	sfi1	2892	0.298636927
ensp00000393583	azi1	2893	0.29874019
ensp00000264982	cep70	2894	0.298843453
ensp00000343818	cdk5rap2	2895	0.298946716
ensp00000355812	fgfr1op	2896	0.299049979
ensp00000352929	csnk1e	2897	0.299153242
ensp00000324464	csnk1d	2898	0.299256506
ensp00000261965	tubgcp3	2899	0.299359769
ensp00000352572	pcnt	2900	0.299463032

ensp00000266503	arntl2	2901	0.299566295
ensp00000308741	clock	2902	0.299669558
ensp00000338283	npas2	2903	0.299772821
ensp00000374357	arntl	2904	0.299876084
ensp00000249396	sirt2	2905	0.299979347
ensp00000365991	dnajc3	2907	0.300185874
ensp00000254322	dnajb1	2908	0.300289137
ensp00000354111	dnajc5	2909	0.3003924
ensp00000227378	hspa8	2910	0.300495663
ensp00000322845	zmym3	2912	0.300702189
ensp00000253925	ppfia1	2913	0.300805452
ensp00000388373	ppfia2	2914	0.300908715
ensp00000355692	mrpl55	2915	0.301011979
ensp00000353735	topors	2916	0.301115242
ensp00000340989	sfn	2918	0.301321768
ensp00000360025	gadd45a	2919	0.301425031
ensp00000245960	cdc25b	2920	0.301528294
ensp00000370113	ppp2r2a	2921	0.301631557
ensp00000324804	ppp2r1a	2922	0.30173482
ensp00000418447	ppp2ca	2923	0.301838083
ensp00000221138	ppp2cb	2924	0.301941347
ensp00000311344	ppp2r1b	2925	0.30204461
ensp00000308699	rec8	2926	0.302147873
ensp00000306330	ywhag	2928	0.302354399
ensp00000264335	ywhae	2929	0.302457662
ensp00000363377	foxo4	2930	0.302560925
ensp00000356150	mdm4	2931	0.302664188
ensp00000261182	nap1l1	2932	0.302767451
ensp00000216479	ahsa1	2933	0.302870715
ensp00000305958	stip1	2934	0.302973978
ensp00000001008	fkbp4	2935	0.303077241
ensp00000266427	etv6	2937	0.303283767
ensp00000256429	mbd2	2938	0.30338703
ensp00000369716	chd3	2939	0.303490293
ensp00000357644	gatad2b	2940	0.303593556
ensp00000351552	gatad2a	2941	0.303696819
ensp00000349508	chd4	2942	0.303800083
ensp00000278823	mta2	2943	0.303903346
ensp00000355249	e2f2	2944	0.304006609
ensp00000262904	e2f3	2945	0.304109872
ensp00000318195	ncl	2946	0.304213135
ensp00000309572	tert	2947	0.304316398
ensp00000262033	ptges3	2948	0.304419661
ensp00000286428	vbp1	2949	0.304522924
ensp00000381045	ube3a	2950	0.304626188
ensp00000364929	ing1	2951	0.304729451
ensp00000349723	bcl11b	2954	0.30503924

ensp00000349049	kdm1a	2955	0.305142503
ensp00000262241	rcor1	2956	0.305245766
ensp00000369131	sin3b	2957	0.305349029
ensp00000371973	sap18	2958	0.305452292
ensp00000256495	bhlhe40	2960	0.305658819
ensp00000242728	bhlhe41	2961	0.305762082
ensp00000156825	mbd3	2962	0.305865345
ensp00000398824	phf21a	2963	0.305968608
ensp00000304308	znf217	2964	0.306071871
ensp00000295757	hdac11	2965	0.306175134
ensp00000329933	phf12	2966	0.306278397
ensp00000408617	hdac9	2967	0.30638166
ensp00000408910	dctn2	2969	0.306588187
ensp00000354791	dctn1	2970	0.30669145
ensp00000414906	dctn4	2971	0.306794713
ensp00000225402	aatf	2973	0.307001239
ensp00000300408	phb	2974	0.307104502
ensp00000311816	rest	2975	0.307207765
ensp00000376076	sumo1	2977	0.307414292
ensp00000266000	daxx	2978	0.307517555
ensp00000376611	tdg	2980	0.307724081
ensp00000259803	gcm1	2981	0.307827344
ensp00000260187	usp2	2982	0.307930607
ensp00000288266	appl1	2984	0.308137133
ensp00000264444	mxd1	2985	0.308240397
ensp00000290921	ctbp1	2986	0.30834366
ensp00000311825	ctbp2	2987	0.308446923
ensp00000396704	top2b	2988	0.308550186
ensp00000358921	actr1a	2989	0.308653449
ensp00000417899	mecom	2990	0.308756712
ensp00000302501	zeb2	2991	0.308859975
ensp00000355884	mark1	2992	0.308963238
ensp00000411397	mark3	2993	0.309066501
ensp00000370826	hr	2995	0.309273028
ensp00000219596	mefv	2997	0.309479554
ensp00000247470	pycard	2998	0.309582817
ensp00000269280	nlrp1	2999	0.30968608
ensp00000296504	sap30	3000	0.309789343
ensp00000353622	sin3a	3001	0.309892606
ensp00000362649	hdac1	3002	0.309995869
ensp00000381331	hdac2	3003	0.310099133
ensp00000362674	hdac8	3004	0.310202396
ensp00000321835	etv4	3006	0.310408922
ensp00000325875	hsp90ab1	3008	0.310615448
ensp00000262300	pkmyt1	3010	0.310821974
ensp00000382362	phb2	3011	0.310925238
ensp00000311219	trim59	3012	0.311028501

ensp00000348298	lcor	3013	0.311131764
ensp00000339769	smek2	3014	0.311235027
ensp00000337125	smek1	3015	0.31133829
ensp00000293774	krt4	3016	0.311441553
ensp00000344683	cdk5rap3	3017	0.311544816
ensp00000289865	usp21	3018	0.311648079
ensp00000334808	usp16	3019	0.311751342
ensp00000300843	mark4	3021	0.311957869
ensp00000356989	pfdn2	3023	0.312164395
ensp00000164133	ppp2r5b	3025	0.312370921
ensp00000295951	slmap	3026	0.312474184
ensp00000229395	fgfr1op2	3027	0.312577447
ensp00000263918	strn	3031	0.3129905
ensp00000350071	strn3	3032	0.313093763
ensp00000365730	stk24	3033	0.313197026
ensp00000325748	stk25	3034	0.313300289
ensp00000318697	tubb6	3038	0.313713342
ensp00000360473	pfdn4	3040	0.313919868
ensp00000261813	pfdn1	3041	0.314023131
ensp00000279387	ppp4c	3042	0.314126394
ensp00000351885	ppp2r4	3043	0.314229657
ensp00000344635	ccng1	3044	0.31433292
ensp00000265717	prkar2b	3046	0.314539447
ensp00000348573	akap9	3047	0.31464271
ensp00000321656	cdc25c	3048	0.314745973
ensp00000335084	ppp1cc	3049	0.314849236
ensp00000312122	sec13	3050	0.314952499
ensp00000395535	mecp2	3051	0.315055762
ensp00000368924	tfap2a	3052	0.315159025
ensp00000361275	plk3	3053	0.315262288
ensp00000012443	ppp5c	3054	0.315365551
ensp00000276440	dock5	3055	0.315468815
ensp00000308413	rps6kb2	3056	0.315572078
ensp00000366863	tbc1d4	3057	0.315675341
ensp00000231368	lnpep	3058	0.315778604
ensp00000262320	axin1	3059	0.315881867
ensp00000302625	axin2	3060	0.31598513
ensp00000417963	ppp2r5d	3061	0.316088393
ensp00000364802	hspa1a	3062	0.316191656
ensp00000302961	hspa4	3063	0.316294919
ensp00000237612	abcg2	3064	0.316398183
ensp00000265724	abcb1	3065	0.316501446
ensp00000287295	aifm1	3066	0.316604709
ensp00000363019	ube2d1	3067	0.316707972
ensp00000368684	rassf2	3069	0.316914498
ensp00000349547	rassf1	3070	0.317017761
ensp00000215909	lgals1	3071	0.317121024

ensp00000323670	zbtb7a	3072	0.317224287
ensp00000338157	zbtb16	3073	0.317327551
ensp00000227163	spi1	3074	0.317430814
ensp00000232014	bcl6	3075	0.317534077
ensp00000308227	hmga1	3077	0.317740603
ensp00000264834	klf1	3078	0.317843866
ensp00000258091	cct7	3080	0.318050392
ensp00000275603	cct6a	3081	0.318153656
ensp00000317334	tcp1	3083	0.318360182
ensp00000280326	cct5	3086	0.318669971
ensp00000390500	stk3	3088	0.318876497
ensp00000262653	cbfa2t2	3090	0.319083024
ensp00000233202	slc11a1	3092	0.31928955
ensp00000345464	nhlrc1	3093	0.319392813
ensp00000356489	epm2a	3094	0.319496076
ensp00000362979	tgif2	3095	0.319599339
ensp00000327959	tgif1	3096	0.319702602
ensp00000334061	hdac6	3097	0.319805865
ensp00000307183	ing2	3098	0.319909128
ensp00000367545	tp73	3099	0.320012392
ensp00000314080	hic1	3100	0.320115655
ensp00000300305	runx1	3101	0.320218918
ensp00000345681	gata2	3103	0.320425444
ensp00000338207	lmo1	3104	0.320528707
ensp00000160373	cttnbp2	3105	0.32063197
ensp00000271277	cttnbp2nl	3106	0.320735233
ensp00000314129	tfe3	3107	0.320838496
ensp00000295834	fabp1	3109	0.321045023
ensp00000263780	chmp2b	3110	0.321148286
ensp00000290330	snf8	3111	0.321251549
ensp00000367299	vps36	3112	0.321354812
ensp00000366565	vps28	3114	0.321561338
ensp00000318629	vps37a	3116	0.321767865
ensp00000320416	vps37d	3117	0.321871128
ensp00000223500	chmp5	3119	0.322077654
ensp00000324491	chmp7	3120	0.322180917
ensp00000317468	chmp6	3121	0.32228418
ensp00000297265	chmp4c	3122	0.322387443
ensp00000324205	chmp4a	3123	0.322490706
ensp00000217402	chmp4b	3124	0.322593969
ensp00000156084	otud5	3125	0.322697233
ensp00000255688	rarres3	3126	0.322800496
ensp00000265562	ptpn23	3131	0.323316811
ensp00000295777	serpini1	3133	0.323523337
ensp00000301336	rilp	3134	0.323626601
ensp00000221114	dctn6	3135	0.323729864
ensp00000320130	dync1i1	3136	0.323833127

ensp00000258198	dync1li2	3137	0.32393639
ensp00000381167	dync2h1	3138	0.324039653
ensp00000300087	dctn5	3139	0.324142916
ensp00000359685	ptp4a1	3140	0.324246179
ensp00000244050	snai1	3141	0.324349442
ensp00000365280	id1	3142	0.324452705
ensp00000367797	ski	3143	0.324555969
ensp00000351250	trim33	3144	0.324659232
ensp00000369703	tubb2a	3145	0.324762495
ensp00000336799	tuba1b	3146	0.324865758
ensp00000369784	tubal3	3148	0.325072284
ensp00000248437	tuba4a	3149	0.325175547
ensp00000382982	tuba3c	3150	0.32527881
ensp00000301072	tuba1c	3151	0.325382074
ensp00000301071	tuba1a	3152	0.325485337
ensp00000294339	tal1	3153	0.3255886
ensp00000326630	zfpm1	3155	0.325795126
ensp00000256119	ca1	3156	0.325898389
ensp00000384179	zfpm2	3157	0.326001652
ensp00000269216	gata6	3159	0.326208178
ensp00000349124	ppp4r2	3160	0.326311442
ensp00000296490	wdr82	3161	0.326414705
ensp00000351894	ncoa6	3162	0.326517968
ensp00000260129	tgs1	3163	0.326621231
ensp00000405574	tbl1xr1	3164	0.326724494
ensp00000302967	hdac3	3165	0.326827757
ensp00000268712	ncor1	3166	0.32693102
ensp00000259119	skil	3167	0.327034283
ensp00000358918	sufu	3168	0.327137546
ensp00000295709	stk36	3169	0.32724081
ensp00000262370	mgrn1	3171	0.327447336
ensp00000355924	smyd2	3172	0.327550599
ensp00000356602	vta1	3173	0.327653862
ensp00000238497	vps4b	3174	0.327757125
ensp00000370330	erbb2ip	3176	0.327963651
ensp00000335153	hsp90aa1	3177	0.328066914
ensp00000219548	stub1	3178	0.328170178
ensp00000340278	park7	3179	0.328273441
ensp00000284440	uchl1	3180	0.328376704
ensp00000310301	sp3	3181	0.328479967
ensp00000329357	sp1	3182	0.32858323
ensp00000367207	myc	3183	0.328686493
ensp00000351490	max	3184	0.328789756
ensp00000364895	zbtb17	3185	0.328893019
ensp00000026218	pigq	3187	0.329099546
ensp00000310440	chmp2a	3190	0.329409335
ensp00000288199	rnf111	3191	0.329512598

ensp00000225983	hdac5	3192	0.329615861
ensp00000264606	hdac4	3193	0.329719124
ensp00000216029	cby1	3194	0.329822387
ensp00000364912	spen	3196	0.330028914
ensp00000338272	hey1	3197	0.330132177
ensp00000262965	tcf3	3198	0.33023544
ensp00000331057	tcf12	3199	0.330338703
ensp00000392617	smarcd2	3200	0.330441966
ensp00000275248	arid1b	3201	0.330545229
ensp00000381791	dpf3	3202	0.330648492
ensp00000259818	tubb2b	3203	0.330751755
ensp00000366534	foxh1	3204	0.330855019
ensp00000341805	phf10	3205	0.330958282
ensp00000320485	arid1a	3206	0.331061545
ensp00000342434	baz1b	3209	0.331371334
ensp00000349213	pbrm1	3210	0.331474597
ensp00000335044	arid2	3211	0.33157786
ensp00000256460	camk1	3212	0.331681124
ensp00000279146	aip	3213	0.331784387
ensp00000319977	ndrg1	3214	0.33188765
ensp00000361943	heyl	3216	0.332094176
ensp00000388548	cited1	3217	0.332197439
ensp00000263121	smarcb1	3222	0.332713755
ensp00000265773	smarca2	3224	0.332920281
ensp00000254480	smarcc1	3225	0.333023544
ensp00000323967	smarce1	3226	0.333126807
ensp00000340514	znf14	3227	0.33323007
ensp00000262854	huwe1	3228	0.333333333
ensp00000262971	pias4	3229	0.333436596
ensp00000249636	pias1	3230	0.33353986
ensp00000381648	pias2	3231	0.333643123
ensp00000368632	gata3	3232	0.333746386
ensp00000345206	rbpj	3233	0.333849649
ensp00000365682	tle1	3235	0.334056175
ensp00000257555	hnf1a	3236	0.334159438
ensp00000221561	aes	3237	0.334262701
ensp00000373992	pou2f2	3239	0.334469228
ensp00000164227	bcl3	3240	0.334572491
ensp00000242057	ahr	3241	0.334675754
ensp00000234091	id2	3242	0.334779017
ensp00000242261	twist1	3243	0.33488228
ensp00000212015	sirt1	3244	0.334985543
ensp00000417281	mdm2	3245	0.335088806
ensp00000269305	tp53	3246	0.335192069
ensp00000355865	park2	3248	0.335398596
ensp00000261368	sncaip	3249	0.335501859
ensp00000249373	smo	3250	0.335605122

ensp00000363689	id3	3251	0.335708385
ensp00000327758	nkx2-5	3252	0.335811648
ensp00000320413	rab23	3254	0.336018174
ensp00000003084	cftr	3256	0.336224701
ensp00000228682	gli1	3257	0.336327964
ensp00000354586	gli2	3259	0.33653449
ensp00000282561	gja1	3260	0.336637753
ensp00000363822	ar	3261	0.336741016
ensp00000231509	nr3c1	3262	0.336844279
ensp00000336591	ppp2r2b	3264	0.337050805
ensp00000335083	ppp2r2c	3265	0.337154069
ensp00000320768	rcan1	3267	0.337360595
ensp00000384026	hmga2	3268	0.337463858
ensp00000248071	klf2	3269	0.337567121
ensp00000305769	smad1	3270	0.337670384
ensp00000341551	smad4	3271	0.337773647
ensp00000262160	smad2	3272	0.33787691
ensp00000332973	smad3	3273	0.337980173
ensp00000352514	runx2	3274	0.338083437
ensp00000367714	hes5	3276	0.338289963
ensp00000292599	maml1	3278	0.338496489
ensp00000365735	tle4	3280	0.338703015
ensp00000262953	tle2	3281	0.338806278
ensp00000333275	nr2c1	3283	0.339012805
ensp00000264637	thra	3284	0.339116068
ensp00000348827	thrb	3285	0.339219331
ensp00000310928	ppard	3286	0.339322594
ensp00000320447	nr2c2	3288	0.33952912
ensp00000379701	hnf4g	3289	0.339632383
ensp00000325819	nr2f1	3290	0.339735646
ensp00000312987	hnf4a	3291	0.33983891
ensp00000291527	tff1	3293	0.340045436
ensp00000361366	sftpd	3294	0.340148699
ensp00000395498	pax8	3295	0.340251962
ensp00000261740	trpv4	3297	0.340458488
ensp00000316357	usp9x	3298	0.340561751
ensp00000346440	tcf4	3299	0.340665014
ensp00000359022	elovl3	3300	0.340768278
ensp00000295641	stk11ip	3301	0.340871541
ensp00000296861	tnfrsf21	3302	0.340974804
ensp00000303192	rgl1	3303	0.341078067
ensp00000276914	plin2	3304	0.34118133
ensp00000332695	rarg	3305	0.341284593
ensp00000261532	esrrb	3306	0.341387856
ensp00000366093	rorb	3307	0.341491119
ensp00000233557	nrbp1	3308	0.341594382
ensp00000402590	rxrb	3309	0.341697646

ensp00000368253	nr0b1	3311	0.341904172
ensp00000355014	pja1	3312	0.342007435
ensp00000368086	rab3ip	3313	0.342110698
ensp00000307971	rfng	3314	0.342213961
ensp00000349490	mfng	3315	0.342317224
ensp00000322885	dtx2	3316	0.342420487
ensp00000252891	numbl	3317	0.342523751
ensp00000307479	arnt2	3318	0.342627014
ensp00000246672	nr1d1	3319	0.342730277
ensp00000261523	rora	3320	0.34283354
ensp00000291442	nr2f6	3321	0.342936803
ensp00000332823	nr2c2ap	3323	0.343143329
ensp00000386171	esrrg	3324	0.343246592
ensp00000000442	esrra	3325	0.343349855
ensp00000310006	nr1d2	3326	0.343453119
ensp00000287820	pparg	3327	0.343556382
ensp00000229022	vdr	3329	0.343762908
ensp00000254066	rara	3330	0.343866171
ensp00000206249	esr1	3331	0.343969434
ensp00000323858	ddx54	3333	0.34417596
ensp00000264977	ppp2r3a	3334	0.344279223
ensp00000305924	ppp4r4	3335	0.344382487
ensp00000307786	cycs	3336	0.34448575
ensp00000407181	ppp1r10	3337	0.344589013
ensp00000337675	ebag9	3338	0.344692276
ensp00000332296	rarb	3339	0.344795539
ensp00000344479	nr4a2	3340	0.344898802
ensp00000327025	rorc	3342	0.345105328
ensp00000376250	ccdc50	3344	0.345311855
ensp00000364902	pofut1	3345	0.345415118
ensp00000227451	dtx4	3347	0.345621644
ensp00000257600	dtx1	3348	0.345724907
ensp00000277541	notch1	3349	0.34582817
ensp00000263388	notch3	3350	0.345931433
ensp00000410674	notch4	3351	0.346034696
ensp00000205143	dll3	3352	0.34613796
ensp00000338050	dtx3	3353	0.346241223
ensp00000252595	slc27a1	3354	0.346344486
ensp00000338018	hif1a	3355	0.346447749
ensp00000351407	arnt	3356	0.346551012
ensp00000263734	epas1	3357	0.346654275
ensp00000080059	hdac7	3358	0.346757538
ensp00000356623	cited2	3359	0.346860801
ensp00000367608	ca9	3360	0.346964064
ensp00000299163	hif1an	3361	0.347067328
ensp00000369126	csnk1a1l	3362	0.347170591
ensp00000233607	apc2	3364	0.347377117

ensp00000257430	apc	3365	0.34748038
ensp00000222330	gsk3a	3366	0.347583643
ensp00000291525	tff3	3367	0.347686906
ensp00000346762	snd1	3368	0.347790169
ensp00000219431	mpg	3370	0.347996696
ensp00000262059	calcoco1	3371	0.348099959
ensp00000320940	ncoa1	3372	0.348203222
ensp00000348551	ncor2	3373	0.348306485
ensp00000361066	ncoa3	3374	0.348409748
ensp00000264867	ppargc1a	3375	0.348513011
ensp00000282892	med21	3376	0.348616274
ensp00000265350	med20	3377	0.348719537
ensp00000293777	med11	3378	0.3488228
ensp00000362082	ccnd3	3379	0.348926064
ensp00000262662	cdkn2c	3380	0.349029327
ensp00000353059	apaf1	3381	0.34913259
ensp00000358022	mcl1	3382	0.349235853
ensp00000222574	hbp1	3384	0.349442379
ensp00000241651	myog	3386	0.349648905
ensp00000355124	krt19	3387	0.349752169
ensp00000255641	csnk1g2	3388	0.349855432
ensp00000353904	csnk1g3	3389	0.349958695
ensp00000305777	csnk1g1	3390	0.350061958
ensp00000360060	frat1	3391	0.350165221
ensp00000360058	frat2	3392	0.350268484
ensp00000326119	pmaip1	3393	0.350371747
ensp00000404503	bbc3	3394	0.35047501
ensp00000363591	bak1	3395	0.350578273
ensp00000296161	dtx3l	3396	0.350681537
ensp00000261537	mib1	3397	0.3507848
ensp00000328169	jag2	3398	0.350888063
ensp00000257724	mdfic	3399	0.350991326
ensp00000328879	tbl1y	3400	0.351094589
ensp00000261461	ppp2r5a	3402	0.351301115
ensp00000333905	ppp2r5c	3403	0.351404378
ensp00000262709	tox4	3405	0.351610905
ensp00000358464	shoc2	3407	0.351817431
ensp00000349156	siah1	3408	0.351920694
ensp00000322457	siah2	3409	0.352023957
ensp00000340347	tcf7	3410	0.35212722
ensp00000282111	tcf7l1	3411	0.352230483
ensp00000358404	tcf7l2	3412	0.352333746
ensp00000363804	klf4	3413	0.35243701
ensp00000323720	med14	3414	0.352540273
ensp00000348610	med24	3415	0.352643536
ensp00000300651	med1	3416	0.352746799
ensp00000327213	nrip1	3417	0.352850062

ensp00000352900	rxrg	3418	0.352953325
ensp00000263119	cabin1	3419	0.353056588
ensp00000381098	grip1	3420	0.353159851
ensp00000355231	becn1	3422	0.353366378
ensp00000340820	mapt	3423	0.353469641
ensp00000363812	rxrb	3425	0.353676167
ensp00000357625	bnip3	3426	0.35377943
ensp00000315442	nr1h4	3428	0.353985956
ensp00000412045	txnrd1	3430	0.354192482
ensp00000324494	grhl1	3431	0.354295746
ensp00000234310	ppp3r1	3433	0.354502272
ensp00000378323	ppp3ca	3434	0.354605535
ensp00000265333	vdac1	3436	0.354812061
ensp00000357440	hsf2	3440	0.355225114
ensp00000352712	dach1	3441	0.355328377
ensp00000344352	atf3	3442	0.35543164
ensp00000257572	hrk	3443	0.355534903
ensp00000345229	dner	3444	0.355638166
ensp00000341882	cntn6	3445	0.355741429
ensp00000167586	krt14	3446	0.355844692
ensp00000370003	bnip3l	3447	0.355947955
ensp00000384625	nlk	3448	0.356051219
ensp00000297316	sox17	3449	0.356154482
ensp00000234347	prtn3	3451	0.356361008
ensp00000361892	stk4	3453	0.356567534
ensp00000324729	sav1	3454	0.356670797
ensp00000350267	brpf3	3455	0.35677406
ensp00000337127	sod2	3457	0.356980587
ensp00000364430	sp5	3459	0.357187113
ensp00000216115	bik	3463	0.357600165
ensp00000362299	eng	3465	0.357806691
ensp00000260605	dync2li1	3467	0.358013218
ensp00000320291	osbpl1a	3468	0.358116481
ensp00000356056	dynlt1	3469	0.358219744
ensp00000296755	map1b	3470	0.358323007
ensp00000326767	med25	3471	0.35842627
ensp00000263205	med15	3472	0.358529533
ensp00000251871	med17	3474	0.358736059
ensp00000363193	med12	3475	0.358839323
ensp00000380888	med13	3476	0.358942586
ensp00000370938	cdk8	3477	0.359045849
ensp00000358222	ccnc	3478	0.359149112
ensp00000256646	notch2	3479	0.359252375
ensp00000355718	dll1	3480	0.359355638
ensp00000254958	jag1	3481	0.359458901
ensp00000339527	foxo3	3482	0.359562164
ensp00000368880	foxo1	3483	0.359665428

ensp00000302564	bcl2l1	3484	0.359768691
ensp00000329623	bcl2	3485	0.359871954
ensp00000020945	snai2	3486	0.359975217
ensp00000339992	myb	3487	0.36007848
ensp00000265165	lef1	3488	0.360181743
ensp00000304277	tm4sf1	3489	0.360285006
ensp00000357047	med23	3490	0.360388269
ensp00000256379	med6	3491	0.360491532
ensp00000258648	med4	3493	0.360698059
ensp00000290663	med8	3494	0.360801322
ensp00000286317	med7	3495	0.360904585
ensp00000396219	mef2c	3500	0.3614209
ensp00000271555	mef2d	3501	0.361524164
ensp00000357804	them4	3502	0.361627427
ensp00000217233	trib3	3503	0.36173069
ensp00000300056	pex11a	3504	0.361833953
ensp00000301455	angptl4	3505	0.361937216
ensp00000337340	med19	3506	0.362040479
ensp00000255764	med10	3507	0.362143742
ensp00000268711	med9	3508	0.362247005
ensp00000346634	thrap3	3509	0.362350268
ensp00000311747	rbm14	3510	0.362453532
ensp00000262506	csnk2a2	3512	0.362660058
ensp00000217244	csnk2a1	3513	0.362763321
ensp00000250405	bcl2l2	3515	0.362969847
ensp00000293288	bax	3516	0.36307311
ensp00000322909	fhl2	3517	0.363176373
ensp00000265723	abcb4	3518	0.363279637
ensp00000355996	g0s2	3519	0.3633829
ensp00000297347	med30	3520	0.363486163
ensp00000314343	med29	3521	0.363589426
ensp00000362948	med18	3522	0.363692689
ensp00000342343	med22	3523	0.363795952
ensp00000281928	med13l	3524	0.363899215
ensp00000292035	med27	3526	0.364105741
ensp00000261879	aph1b	3528	0.364312268
ensp00000413697	tom1	3531	0.364622057
ensp00000310800	vangl1	3532	0.36472532
ensp00000345064	prickle1	3533	0.364828583
ensp00000295902	prickle2	3534	0.364931846
ensp00000399511	tnik	3535	0.365035109
ensp00000316054	dvl3	3536	0.365138373
ensp00000005340	dvl2	3537	0.365241636
ensp00000368169	dvl1	3538	0.365344899
ensp00000330523	cthrc1	3539	0.365448162
ensp00000357040	vangl2	3540	0.365551425
ensp00000386165	cebpd	3543	0.365861214

ensp00000305422	cebpb	3544	0.365964477
ensp00000366915	klf5	3545	0.366067741
ensp00000348069	srebf1	3546	0.366171004
ensp00000362092	rragc	3547	0.366274267
ensp00000358423	rragd	3548	0.36637753
ensp00000296849	nkd2	3549	0.366480793
ensp00000379441	jmy	3550	0.366584056
ensp00000262304	pkd1	3551	0.366687319
ensp00000227155	cd82	3552	0.366790582
ensp00000386161	wwox	3553	0.366893846
ensp00000354218	ppp1r13l	3554	0.366997109
ensp00000242480	egr2	3555	0.367100372
ensp00000263253	ep300	3556	0.367203635
ensp00000262367	crebbp	3557	0.367306898
ensp00000325690	carm1	3558	0.367410161
ensp00000399968	ncoa2	3559	0.367513424
ensp00000419692	rxra	3560	0.367616687
ensp00000262735	ppara	3561	0.36771995
ensp00000312649	ppargc1b	3562	0.367823214
ensp00000416293	slc2a1	3563	0.367926477
ensp00000395337	ldha	3564	0.36802974
ensp00000307305	ddit4	3565	0.368133003
ensp00000270162	sik1	3566	0.368236266
ensp00000292055	sik3	3567	0.368339529
ensp00000361718	ngfrap1	3569	0.368546055
ensp00000341698	akt1s1	3570	0.368649318
ensp00000301061	wnt10b	3572	0.368855845
ensp00000225728	med31	3574	0.369062371
ensp00000265293	wwc1	3577	0.36937216
ensp00000367398	nphp4	3578	0.369475423
ensp00000341785	adipor1	3580	0.36968195
ensp00000349616	adipor2	3581	0.369785213
ensp00000266659	glipr1	3582	0.369888476
ensp00000345772	tead3	3583	0.369991739
ensp00000262188	smarcd3	3584	0.370095002
ensp00000381522	chd9	3585	0.370198265
ensp00000308165	cd36	3586	0.370301528
ensp00000360762	ankrd1	3587	0.370404791
ensp00000353847	wwtr1	3588	0.370508055
ensp00000282441	yap1	3589	0.370611318
ensp00000352926	tead4	3590	0.370714581
ensp00000310701	tead2	3591	0.370817844
ensp00000333950	fmn1	3593	0.37102437
ensp00000261769	cdh1	3594	0.371127633
ensp00000379156	mprip	3595	0.371230896
ensp00000247170	daam1	3596	0.371334159
ensp00000381876	daam2	3597	0.371437423

ensp00000372035	lats2	3598	0.371540686
ensp00000387739	amotl1	3599	0.371643949
ensp00000249883	amotl2	3600	0.371747212
ensp00000267953	bcl2a1	3601	0.371850475
ensp00000278968	tagln	3603	0.372057001
ensp00000354588	tead1	3604	0.372160264
ensp00000363868	abca1	3605	0.372263527
ensp00000328968	scn5a	3606	0.372366791
ensp00000350785	dapk1	3607	0.372470054
ensp00000410452	stmn1	3608	0.372573317
ensp00000356832	sgk1	3610	0.372779843
ensp00000275525	igfbp1	3611	0.372883106
ensp00000269593	igfbp4	3612	0.372986369
ensp00000341138	epb41l3	3614	0.373192895
ensp00000296474	mst1r	3615	0.373296159
ensp00000219476	tsc2	3616	0.373399422
ensp00000298552	tsc1	3617	0.373502685
ensp00000253339	lats1	3618	0.373605948
ensp00000356954	ctgf	3619	0.373709211
ensp00000258449	tgfbrap1	3620	0.373812474
ensp00000414598	mrvi1	3621	0.373915737
ensp00000369689	calml5	3622	0.374019
ensp00000349467	calm1	3624	0.374225527
ensp00000315299	calml3	3625	0.37432879
ensp00000282356	camk4	3626	0.374432053
ensp00000353344	ets2	3627	0.374535316
ensp00000337464	prr5	3628	0.374638579
ensp00000265960	mapkap1	3629	0.374741842
ensp00000025008	rb1cc1	3630	0.374845105
ensp00000305976	sik2	3631	0.374948368
ensp00000310649	brsk1	3632	0.375051632
ensp00000272065	acp1	3633	0.375154895
ensp00000317904	gys1	3634	0.375258158
ensp00000261195	gys2	3635	0.375361421
ensp00000257905	ppp1r1a	3636	0.375464684
ensp00000319370	kcnmb3	3638	0.37567121
ensp00000354877	ulk2	3639	0.375774473
ensp00000324560	ulk1	3640	0.375877736
ensp00000366506	hrh2	3642	0.376084263
ensp00000304501	adora2b	3643	0.376187526
ensp00000342136	creb3	3645	0.376394052
ensp00000329140	creb3l2	3646	0.376497315
ensp00000288400	creb3l1	3647	0.376600578
ensp00000350359	creb5	3648	0.376703841
ensp00000309103	bad	3649	0.376807105
ensp00000324806	gsk3b	3650	0.376910368
ensp00000344456	ctnnb1	3651	0.377013631

ensp00000309503	ywhaz	3652	0.377116894
ensp00000300161	ywhab	3653	0.377220157
ensp00000238081	ywhaq	3654	0.37732342
ensp00000248975	ywhah	3655	0.377426683
ensp00000268763	ksr1	3656	0.377529946
ensp00000308383	lsp1	3657	0.377633209
ensp00000258418	cab39	3658	0.377736473
ensp00000194530	stradb	3659	0.377839736
ensp00000336655	strada	3660	0.377942999
ensp00000324856	stk11	3661	0.378046262
ensp00000327255	ppm1a	3662	0.378149525
ensp00000415151	cbfb	3663	0.378252788
ensp00000343477	runx3	3664	0.378356051
ensp00000358105	aph1a	3665	0.378459314
ensp00000294785	ncstn	3666	0.378562577
ensp00000310697	brsk2	3669	0.378872367
ensp00000334928	ptprb	3670	0.37897563
ensp00000312741	camkk2	3671	0.379078893
ensp00000158166	camkk1	3672	0.379182156
ensp00000352608	ryr1	3673	0.379285419
ensp00000264399	prkg2	3674	0.379388682
ensp00000301242	ppp1r14a	3676	0.379595209
ensp00000238994	ppp1r3c	3678	0.379801735
ensp00000308318	ppp1r3b	3679	0.379904998
ensp00000346148	prkaa1	3681	0.380111524
ensp00000229328	prkab1	3682	0.380214787
ensp00000233944	prkag3	3683	0.38031805
ensp00000323867	prkag1	3684	0.380421314
ensp00000286627	kcnma1	3685	0.380524577
ensp00000258111	kcnmb4	3686	0.38062784
ensp00000274629	kcnmb1	3687	0.380731103
ensp00000240139	ppp3cc	3688	0.380834366
ensp00000220772	sfrp1	3693	0.381350682
ensp00000410715	sfrp4	3695	0.381557208
ensp00000294304	lrp5	3696	0.381660471
ensp00000331418	ptprm	3697	0.381763734
ensp00000341032	wnt7b	3699	0.38197026
ensp00000358698	wnt2b	3700	0.382073523
ensp00000308887	wnt5b	3702	0.38228005
ensp00000262187	rheb	3703	0.382383313
ensp00000307272	rptor	3704	0.382486576
ensp00000354558	mtor	3705	0.382589839
ensp00000371888	mlst8	3706	0.382693102
ensp00000349959	rictor	3707	0.382796365
ensp00000361458	tsc22d3	3708	0.382899628
ensp00000296084	ryk	3709	0.383002891
ensp00000322304	porcn	3710	0.383106154

ensp00000261439	tbc1d1	3711	0.383209418
ensp00000320935	slc2a4	3712	0.383312681
ensp00000319814	pck1	3713	0.383415944
ensp00000293549	wnt1	3714	0.383519207
ensp00000225512	wnt3	3715	0.38362247
ensp00000258411	wnt10a	3720	0.384138786
ensp00000233948	wnt6	3722	0.384345312
ensp00000354607	fzd5	3724	0.384551838
ensp00000351605	fzd6	3726	0.384758364
ensp00000286201	fzd7	3727	0.384861627
ensp00000363826	fzd8	3728	0.384964891
ensp00000287934	fzd1	3729	0.385068154
ensp00000229030	fzd10	3730	0.385171417
ensp00000345785	fzd9	3731	0.38527468
ensp00000240093	fzd3	3732	0.385377943
ensp00000323901	fzd2	3733	0.385481206
ensp00000264634	wnt5a	3734	0.385584469
ensp00000363081	dkk1	3735	0.385687732
ensp00000360290	prkaa2	3736	0.385790995
ensp00000254101	prkab2	3737	0.385894259
ensp00000287878	prkag2	3738	0.385997522
ensp00000261669	cab39l	3739	0.386100785
ensp00000290953	agrp	3740	0.386204048
ensp00000226359	afp	3741	0.386307311
ensp00000216037	xbp1	3742	0.386410574
ensp00000370253	nkx3-1	3743	0.386513837
ensp00000285311	dkk2	3744	0.3866171
ensp00000331242	kremen1	3745	0.386720363
ensp00000304422	kremen2	3746	0.386823627
ensp00000253332	akap12	3749	0.387133416
ensp00000025301	akap11	3750	0.387236679
ensp00000297373	phkg1	3751	0.387339942
ensp00000362643	phka1	3752	0.387443205
ensp00000329968	phkg2	3753	0.387546468
ensp00000328178	ppp1r2	3754	0.387649732
ensp00000261349	lrp6	3755	0.387752995
ensp00000284523	wnt3a	3756	0.387856258
ensp00000285018	wnt7a	3757	0.387959521
ensp00000253475	chmp1a	3759	0.388166047
ensp00000228437	prdm4	3760	0.38826931
ensp00000343706	mageh1	3761	0.388372573
ensp00000332643	ndn	3762	0.388475836
ensp00000256637	sort1	3763	0.3885791
ensp00000409872	znf274	3764	0.388682363
ensp00000369274	phka2	3765	0.388785626
ensp00000313504	phkb	3766	0.388888889
ensp00000364847	maged1	3768	0.389095415

ensp00000265164	casp6	3769	0.389198678
ensp00000312664	casp2	3770	0.389301941
ensp00000318822	bid	3771	0.389405204
ensp00000326170	rffl	3773	0.389611731
ensp00000276218	gpr119	3775	0.389818257
ensp00000263281	gipr	3777	0.390024783
ensp00000254661	ramp1	3779	0.390231309
ensp00000340736	gyg1	3780	0.390334572
ensp00000216962	pygb	3781	0.390437836
ensp00000216392	pygl	3782	0.390541099
ensp00000410833	gbe1	3783	0.390644362
ensp00000401445	ern1	3784	0.390747625
ensp00000328203	traip	3785	0.390850888
ensp00000326737	tnfrsf12a	3786	0.390954151
ensp00000265354	srf	3790	0.391367204
ensp00000384085	etv1	3791	0.391470467
ensp00000295600	mitf	3792	0.39157373
ensp00000376765	pias3	3793	0.391676993
ensp00000373487	krt18	3794	0.391780256
ensp00000254695	rap1gap2	3795	0.391883519
ensp00000363897	rap1gap	3796	0.391986782
ensp00000264012	cdh3	3799	0.392296572
ensp00000378400	lima1	3800	0.392399835
ensp00000260408	adam10	3802	0.392606361
ensp00000326366	psen1	3803	0.392709624
ensp00000355747	psen2	3804	0.392812887
ensp00000311032	casp3	3806	0.393019413
ensp00000267169	diablo	3807	0.393122677
ensp00000258080	htra2	3808	0.39322594
ensp00000321999	pth1r	3811	0.393535729
ensp00000262178	vipr2	3812	0.393638992
ensp00000299766	mc4r	3813	0.393742255
ensp00000278175	adm	3815	0.393948781
ensp00000336630	adora2a	3816	0.394052045
ensp00000343782	adrb3	3818	0.394258571
ensp00000358301	adrb1	3819	0.394361834
ensp00000305372	adrb2	3820	0.394465097
ensp00000338072	avpr2	3821	0.39456836
ensp00000245457	ptger2	3826	0.395084676
ensp00000303424	ptgdr	3829	0.395394465
ensp00000379086	adm2	3830	0.395497728
ensp00000302846	ptger4	3831	0.395600991
ensp00000254235	adcy7	3832	0.395704254
ensp00000297323	adcy1	3833	0.395807518
ensp00000286355	adcy8	3834	0.395910781
ensp00000312126	adcy4	3835	0.396014044
ensp00000318077	mc5r	3838	0.396323833

ensp00000337949	htr7	3839	0.396427096
ensp00000289753	htr6	3841	0.396633622
ensp00000340691	eif4ebp1	3842	0.396736886
ensp00000262441	glp2r	3845	0.397046675
ensp00000176195	sct	3847	0.397253201
ensp00000264917	pde8b	3848	0.397356464
ensp00000240652	iapp	3850	0.39756299
ensp00000331746	calca	3851	0.397666254
ensp00000327246	vipr1	3852	0.397769517
ensp00000291294	ptgir	3853	0.39787278
ensp00000352561	calcr	3854	0.397976043
ensp00000308024	pcsk1	3855	0.398079306
ensp00000264381	bche	3856	0.398182569
ensp00000371527	spata13	3857	0.398285832
ensp00000340742	dock7	3858	0.398389095
ensp00000358795	neurl	3859	0.398492359
ensp00000276571	crh	3860	0.398595622
ensp00000379213	pthlh	3861	0.398698885
ensp00000264708	pomc	3865	0.399111937
ensp00000328708	rxfp3	3867	0.399318463
ensp00000242152	npy	3868	0.399421727
ensp00000176183	drd4	3869	0.39952499
ensp00000223642	c5	3870	0.399628253
ensp00000245907	c3	3871	0.399731516
ensp00000267377	sstr1	3875	0.400144568
ensp00000330138	sstr3	3877	0.400351095
ensp00000408005	slc9a3r2	3879	0.400557621
ensp00000366248	cort	3880	0.400660884
ensp00000369647	avp	3882	0.40086741
ensp00000404814	atf6b	3883	0.400970673
ensp00000265495	elf2	3884	0.401073936
ensp00000342381	vrk2	3885	0.4011772
ensp00000378332	tgfb1i1	3886	0.401280463
ensp00000078445	creb3l3	3889	0.401590252
ensp00000336790	atf4	3890	0.401693515
ensp00000376177	calcrl	3891	0.401796778
ensp00000344411	tas1r3	3893	0.402003304
ensp00000264938	slc9a3	3894	0.402106568
ensp00000261207	ppp1r12a	3895	0.402209831
ensp00000296122	ppp1cb	3896	0.402313094
ensp00000384264	cnga1	3898	0.40251962
ensp00000251337	gnat2	3901	0.402829409
ensp00000334134	rgs9bp	3902	0.402932672
ensp00000369638	snx1	3904	0.403139199
ensp00000413625	fnbp1	3905	0.403242462
ensp00000251973	card10	3906	0.403345725
ensp00000349076	atn1	3907	0.403448988

ensp00000357753	ivl	3908	0.403552251
ensp00000252818	jund	3909	0.403655514
ensp00000264110	atf2	3910	0.403758777
ensp00000340671	ddit3	3911	0.40386204
ensp00000324173	hspa5	3912	0.403965304
ensp00000393596	birc6	3913	0.404068567
ensp00000348283	wwp2	3914	0.40417183
ensp00000218652	ndfip2	3917	0.404481619
ensp00000242719	rnf11	3918	0.404584882
ensp00000347314	spg20	3919	0.404688145
ensp00000350195	map3k6	3923	0.405101198
ensp00000355146	gprasp1	3924	0.405204461
ensp00000206262	rgs17	3926	0.405410987
ensp00000234961	oprd1	3928	0.405617513
ensp00000419361	adcy5	3931	0.405927303
ensp00000294016	adcy9	3932	0.406030566
ensp00000260600	adcy3	3933	0.406133829
ensp00000334564	polr3c	3934	0.406237092
ensp00000405041	pou5f1	3935	0.406340355
ensp00000379457	faf1	3936	0.406443618
ensp00000353731	dpp4	3938	0.406650145
ensp00000308549	adora1	3939	0.406753408
ensp00000358963	htr1b	3941	0.406959934
ensp00000302079	c3ar1	3943	0.40716646
ensp00000014930	hebp1	3944	0.407269723
ensp00000287641	sst	3946	0.407476249
ensp00000339377	npy5r	3948	0.407682776
ensp00000330070	npw	3949	0.407786039
ensp00000353198	pyy	3951	0.407992565
ensp00000226524	pf4v1	3952	0.408095828
ensp00000222902	ccl24	3953	0.408199091
ensp00000381129	grk4	3957	0.408612144
ensp00000377204	grk6	3958	0.408715407
ensp00000337065	cxcl14	3960	0.408921933
ensp00000293276	ccl15	3963	0.409231722
ensp00000230568	ly86	3965	0.409438249
ensp00000262406	rgs9	3971	0.410057827
ensp00000336914	fgd3	3972	0.41016109
ensp00000296026	cxcl3	3978	0.410780669
ensp00000259631	ccl27	3979	0.410883932
ensp00000308815	ccl19	3980	0.410987195
ensp00000293275	ccl16	3982	0.411193722
ensp00000354416	ccl28	3984	0.411400248
ensp00000293778	cxcl16	3985	0.411503511
ensp00000342560	hrh3	3991	0.41212309
ensp00000367930	oxer1	3992	0.412226353
ensp00000280155	adra2a	3993	0.412329616

ensp00000386069	adra2c	3994	0.412432879
ensp00000225992	ppy	3998	0.412845931
ensp00000249041	galr3	3999	0.412949195
ensp00000363643	p2ry4	4003	0.413362247
ensp00000336764	oprl1	4008	0.413878563
ensp00000301908	pnoc	4009	0.413981826
ensp00000357301	rxfp4	4010	0.414085089
ensp00000332766	npb	4011	0.414188352
ensp00000302707	fpr1	4013	0.414394878
ensp00000310263	tnfrsf10d	4015	0.414601404
ensp00000221132	tnfrsf10a	4016	0.414704667
ensp00000006053	cx3cl1	4017	0.414807931
ensp00000367605	ccl3l3	4020	0.41511772
ensp00000226317	cxcl6	4022	0.415324246
ensp00000354652	npy1r	4023	0.415427509
ensp00000354859	drd2	4025	0.415634036
ensp00000350198	sstr2	4026	0.415737299
ensp00000332504	ccr10	4027	0.415840562
ensp00000350256	ccr9	4030	0.416150351
ensp00000382166	cx3cr1	4031	0.416253614
ensp00000305464	apln	4035	0.416666667
ensp00000265643	gal	4036	0.41676993
ensp00000355520	rgs7	4039	0.417079719
ensp00000358099	rgs10	4041	0.417286245
ensp00000259406	rgs3	4042	0.417389508
ensp00000395708	rapgef3	4043	0.417492772
ensp00000386229	rgs14	4045	0.417699298
ensp00000397181	rgs4	4046	0.417802561
ensp00000297313	rgs20	4048	0.418009087
ensp00000296140	ccr1	4050	0.418215613
ensp00000246657	ccr7	4051	0.418318876
ensp00000339393	ccr6	4054	0.418628666
ensp00000362795	cxcr3	4055	0.418731929
ensp00000306512	il8	4056	0.418835192
ensp00000250151	ccl4	4057	0.418938455
ensp00000293272	ccl5	4058	0.419041718
ensp00000351671	ccl20	4060	0.419248245
ensp00000296027	cxcl5	4061	0.419351508
ensp00000349003	ptger3	4064	0.419661297
ensp00000333194	rgs19	4067	0.419971086
ensp00000307235	eif2ak3	4069	0.420177613
ensp00000263791	eif2ak4	4070	0.420280876
ensp00000199389	eif2ak1	4071	0.420384139
ensp00000233057	eif2ak2	4072	0.420487402
ensp00000358596	dusp5	4073	0.420590665
ensp00000225245	ccl3	4075	0.420797191
ensp00000225831	ccl2	4076	0.420900454

ensp00000200307	ccl7	4077	0.421003717
ensp00000219244	ccl17	4078	0.421106981
ensp00000005180	ccl26	4080	0.421313507
ensp00000383199	nedd4l	4081	0.42141677
ensp00000262435	smurf2	4082	0.421520033
ensp00000364133	tgfbr1	4083	0.421623296
ensp00000351905	tgfbr2	4084	0.421726559
ensp00000358730	adora3	4086	0.421933086
ensp00000306884	cxcl11	4087	0.422036349
ensp00000292303	ccr5	4091	0.422449401
ensp00000379110	cxcl1	4093	0.422655927
ensp00000373169	drd3	4094	0.42275919
ensp00000409378	chrm4	4095	0.422862454
ensp00000319984	chrm2	4096	0.422965717
ensp00000229264	gnb3	4097	0.42306898
ensp00000248150	gng13	4098	0.423172243
ensp00000367872	gnb1	4099	0.423275506
ensp00000261837	gnb5	4100	0.423378769
ensp00000363411	gng10	4102	0.423585295
ensp00000249016	mchr1	4104	0.423791822
ensp00000257497	anxa1	4106	0.423998348
ensp00000307713	bdkrb2	4107	0.424101611
ensp00000361668	edn2	4108	0.424204874
ensp00000312455	cflar	4109	0.424308137
ensp00000286186	casp10	4110	0.4244114
ensp00000351273	casp8	4111	0.424514663
ensp00000301838	fadd	4112	0.424617926
ensp00000344666	nf2	4113	0.42472119
ensp00000360141	gnas	4114	0.424824453
ensp00000248996	gnaz	4115	0.424927716
ensp00000312999	gnai2	4116	0.425030979
ensp00000262493	gnao1	4117	0.425134242
ensp00000286548	gnaq	4118	0.425237505
ensp00000384665	lpar2	4121	0.425547295
ensp00000351755	lpar1	4122	0.425650558
ensp00000334448	gng2	4123	0.425753821
ensp00000387662	gcg	4124	0.425857084
ensp00000383558	gcgr	4125	0.425960347
ensp00000352839	npsr1	4127	0.426166873
ensp00000264218	nmu	4128	0.426270136
ensp00000255262	nmur2	4130	0.426476663
ensp00000340191	fpr2	4131	0.426579926
ensp00000216629	bdkrb1	4133	0.426786452
ensp00000295619	prok2	4138	0.427302767
ensp00000331358	gast	4139	0.427406031
ensp00000362183	grik3	4140	0.427509294
ensp00000263026	eef2k	4141	0.427612557

ensp00000227758	birc2	4142	0.42771582
ensp00000263464	birc3	4143	0.427819083
ensp00000327647	cradd	4144	0.427922346
ensp00000276431	tnfrsf10b	4145	0.428025609
ensp00000241261	tnfsf10	4146	0.428128872
ensp00000287322	bag4	4147	0.428232135
ensp00000410772	daxx	4148	0.428335399
ensp00000376609	grk5	4149	0.428438662
ensp00000312262	adrbk1	4150	0.428541925
ensp00000235382	rgs2	4151	0.428645188
ensp00000337731	cideb	4153	0.428851714
ensp00000327315	tiam2	4154	0.428954977
ensp00000282077	pdk1	4155	0.42905824
ensp00000344220	pdpk1	4156	0.429161504
ensp00000263826	akt3	4157	0.429264767
ensp00000375892	akt2	4158	0.42936803
ensp00000270202	akt1	4159	0.429471293
ensp00000225577	rps6kb1	4160	0.429574556
ensp00000263915	grb14	4161	0.429677819
ensp00000267017	npff	4162	0.429781082
ensp00000335657	cck	4164	0.429987608
ensp00000309771	p2ry6	4166	0.430194135
ensp00000337128	edn3	4167	0.430297398
ensp00000321106	tac1	4169	0.430503924
ensp00000344353	lpar6	4170	0.430607187
ensp00000263967	pik3ca	4171	0.43071045
ensp00000274335	pik3r1	4172	0.430813713
ensp00000368683	edn1	4173	0.430916976
ensp00000315011	ednra	4174	0.43102024
ensp00000335610	qrfpr	4176	0.431226766
ensp00000323516	uts2r	4177	0.431330029
ensp00000217386	oxt	4178	0.431433292
ensp00000276414	gnrh1	4179	0.431536555
ensp00000359793	ptgfr	4182	0.431846344
ensp00000255380	chrm3	4187	0.43236266
ensp00000252506	gadd45g	4188	0.432465923
ensp00000215631	gadd45b	4189	0.432569186
ensp00000300873	gng8	4191	0.432775713
ensp00000371594	gng7	4192	0.432878976
ensp00000294117	gng3	4193	0.432982239
ensp00000355556	gng4	4194	0.433085502
ensp00000248564	gng11	4195	0.433188765
ensp00000232564	gnb4	4196	0.433292028
ensp00000246115	s1pr4	4197	0.433395291
ensp00000305416	s1pr1	4198	0.433498554
ensp00000350878	s1pr3	4199	0.433601817
ensp00000296028	ppbp	4200	0.433705081

ensp00000284981	app	4202	0.433911607
ensp00000307822	npffr2	4203	0.43401487
ensp00000282018	cysltr2	4207	0.434427922
ensp00000362401	cysltr1	4208	0.434531185
ensp00000324270	oxtr	4209	0.434634449
ensp00000292513	ptger1	4210	0.434737712
ensp00000310405	xcr1	4211	0.434840975
ensp00000386884	cxcr4	4212	0.434944238
ensp00000363548	cxcl12	4213	0.435047501
ensp00000335311	ednrb	4216	0.43535729
ensp00000362810	hcrtr1	4217	0.435460553
ensp00000262613	slc9a3r1	4219	0.43566708
ensp00000351908	map3k5	4220	0.435770343
ensp00000319279	malt1	4222	0.435976869
ensp00000380150	card11	4224	0.436183395
ensp00000275015	nfkbie	4225	0.436286658
ensp00000361141	opn4	4229	0.436699711
ensp00000245983	gnrh2	4230	0.436802974
ensp00000369643	grpr	4231	0.436906237
ensp00000362403	tacr2	4232	0.4370095
ensp00000246553	ffar1	4233	0.437112763
ensp00000262375	dnaja3	4235	0.43731929
ensp00000301264	dapk3	4236	0.437422553
ensp00000328088	pawr	4237	0.437525816
ensp00000295025	rel	4238	0.437629079
ensp00000259455	gabbr2	4239	0.437732342
ensp00000353362	cacna1a	4242	0.438042131
ensp00000300406	gngt2	4244	0.438248658
ensp00000305260	gnb2	4245	0.438351921
ensp00000312988	nfkbib	4246	0.438455184
ensp00000216797	nfkbia	4247	0.438558447
ensp00000226574	nfkb1	4248	0.43866171
ensp00000384273	rela	4249	0.438764973
ensp00000377141	arrb1	4250	0.438868236
ensp00000304767	p2ry1	4253	0.439178026
ensp00000358867	gnai3	4254	0.439281289
ensp00000343027	gnai1	4255	0.439384552
ensp00000263025	mapk3	4256	0.439487815
ensp00000215832	mapk1	4257	0.439591078
ensp00000078429	gna11	4258	0.439694341
ensp00000345487	qrfp	4260	0.439900867
ensp00000310305	p2ry2	4262	0.440107394
ensp00000238699	gpr68	4263	0.440210657
ensp00000246549	ffar2	4264	0.44031392
ensp00000356162	kiss1	4266	0.440520446
ensp00000328818	gpr132	4267	0.440623709
ensp00000307445	ltb4r	4270	0.440933499

ensp00000277942	npffr1	4272	0.441140025
ensp00000234371	kiss1r	4274	0.441346551
ensp00000241256	ghsr	4275	0.441449814
ensp00000363089	tlr4	4276	0.441553077
ensp00000260010	tlr2	4277	0.44165634
ensp00000354932	tlr1	4278	0.441759603
ensp00000371376	tlr6	4279	0.441862867
ensp00000369960	adra1a	4283	0.442275919
ensp00000233957	il18r1	4284	0.442379182
ensp00000264260	il18rap	4285	0.442482445
ensp00000280357	il18	4286	0.442585708
ensp00000222823	nod1	4287	0.442688971
ensp00000317891	tnip1	4288	0.442792235
ensp00000360797	card9	4289	0.442895498
ensp00000279488	dusp6	4290	0.442998761
ensp00000355866	dusp10	4291	0.443102024
ensp00000288943	dusp2	4293	0.44330855
ensp00000228862	dusp16	4294	0.443411813
ensp00000283228	ptprr	4295	0.443515076
ensp00000365435	tnfrsf1b	4296	0.44361834
ensp00000237289	tnfaip3	4297	0.443721603
ensp00000374455	sqstm1	4298	0.443824866
ensp00000339151	ikbkb	4299	0.443928129
ensp00000359424	chuk	4300	0.444031392
ensp00000358622	ikbkg	4301	0.444134655
ensp00000220751	ripk2	4302	0.444237918
ensp00000306662	adra1b	4305	0.444547708
ensp00000368766	adra1d	4306	0.444650971
ensp00000301974	ptafr	4307	0.444754234
ensp00000268058	pml	4308	0.444857497
ensp00000263056	map3k8	4309	0.44496076
ensp00000361009	prex1	4310	0.445064023
ensp00000334105	plcb4	4311	0.445167286
ensp00000279230	plcb3	4312	0.445270549
ensp00000345281	dusp22	4313	0.445373812
ensp00000410668	tnf	4314	0.445477076
ensp00000371138	fkbp1a	4315	0.445580339
ensp00000370297	cer1	4316	0.445683602
ensp00000200453	ppp1r15a	4317	0.445786865
ensp00000347858	xiap	4318	0.445890128
ensp00000293308	krt8	4319	0.445993391
ensp00000239223	dusp1	4320	0.446096654
ensp00000356070	mapkapk2	4321	0.446199917
ensp00000350639	mapkapk3	4322	0.446303181
ensp00000345984	traf3ip2	4323	0.446406444
ensp00000354158	erc1	4325	0.44661297
ensp00000216274	ripk3	4326	0.446716233

ensp00000248244	ticam1	4328	0.446922759
ensp00000386341	ticam2	4329	0.447026022
ensp00000358335	map3k7	4332	0.447335812
ensp00000262158	smad7	4333	0.447439075
ensp00000221930	tgfb1	4334	0.447542338
ensp00000223095	serpine1	4335	0.447645601
ensp00000296029	pf4	4336	0.447748864
ensp00000316228	clec4m	4337	0.447852127
ensp00000359552	pkn2	4338	0.44795539
ensp00000354718	akap13	4339	0.448058653
ensp00000267460	peli2	4340	0.448161917
ensp00000351789	peli1	4341	0.44826518
ensp00000376445	tirap	4342	0.448368443
ensp00000379625	myd88	4343	0.448471706
ensp00000270517	ecsit	4344	0.448574969
ensp00000261233	irak3	4345	0.448678232
ensp00000358997	irak1	4347	0.448884758
ensp00000295797	prkci	4348	0.448988021
ensp00000357727	s100a9	4350	0.449194548
ensp00000357721	s100a8	4351	0.449297811
ensp00000333656	sigirr	4352	0.449401074
ensp00000392858	tnf	4353	0.449504337
ensp00000398698	tnf	4354	0.4496076
ensp00000225737	akap10	4355	0.449710863
ensp00000337736	akap1	4356	0.449814126
ensp00000353415	prkar1b	4358	0.450020653
ensp00000351410	prkar1a	4359	0.450123916
ensp00000322532	peli3	4360	0.450227179
ensp00000314733	tollip	4361	0.450330442
ensp00000072516	il1rap	4362	0.450433705
ensp00000294066	map4k2	4363	0.450536968
ensp00000332171	dmtf1	4365	0.450743494
ensp00000244289	lipe	4366	0.450846758
ensp00000217407	lbp	4368	0.451053284
ensp00000398736	cyr61	4369	0.451156547
ensp00000382423	map3k1	4370	0.45125981
ensp00000247668	traf2	4372	0.451466336
ensp00000361359	cd40	4373	0.451569599
ensp00000303315	junb	4374	0.451672862
ensp00000306245	fos	4375	0.451776126
ensp00000360266	jun	4376	0.451879389
ensp00000310170	fosl1	4377	0.451982652
ensp00000346294	s100a4	4378	0.452085915
ensp00000330237	casp9	4379	0.452189178
ensp00000162749	tnfrsf1a	4380	0.452292441
ensp00000341268	tradd	4381	0.452395704
ensp00000362994	traf1	4383	0.45260223

ensp00000302239	usp8	4385	0.452808757
ensp00000256458	irak2	4387	0.453015283
ensp00000263339	il1a	4388	0.453118546
ensp00000263341	il1b	4389	0.453221809
ensp00000233946	il1r1	4390	0.453325072
ensp00000330959	il1r2	4391	0.453428335
ensp00000263881	map4k3	4392	0.453531599
ensp00000245817	tnfsf9	4393	0.453634862
ensp00000243440	batf3	4395	0.453841388
ensp00000318057	egr3	4396	0.453944651
ensp00000247161	elk1	4397	0.454047914
ensp00000350681	elk4	4398	0.454151177
ensp00000345853	dusp9	4400	0.454357703
ensp00000240100	dusp4	4401	0.454460967
ensp00000296483	dusp7	4402	0.45456423
ensp00000329539	dusp8	4403	0.454667493
ensp00000379612	tax1bp1	4404	0.454770756
ensp00000189444	nfkb2	4405	0.454874019
ensp00000221452	relb	4406	0.454977282
ensp00000419371	azi2	4407	0.455080545
ensp00000354777	tbkbp1	4408	0.455183808
ensp00000258947	calcoco2	4409	0.455287071
ensp00000348273	mbp	4410	0.455390335
ensp00000364336	tbxa2r	4411	0.455493598
ensp00000321326	f2r	4412	0.455596861
ensp00000248076	f2rl3	4413	0.455700124
ensp00000296641	f2rl2	4414	0.455803387
ensp00000296677	f2rl1	4415	0.45590665
ensp00000361813	bex1	4417	0.456113176
ensp00000284818	ly96	4418	0.456216439
ensp00000304236	cd14	4419	0.456319703
ensp00000264716	fosl2	4421	0.456526229
ensp00000267569	jdp2	4422	0.456629492
ensp00000211287	mapk13	4423	0.456732755
ensp00000215659	mapk12	4424	0.456836018
ensp00000333685	mapk11	4425	0.456939281
ensp00000229794	mapk14	4426	0.457042544
ensp00000354394	stat1	4427	0.457145808
ensp00000264657	stat3	4428	0.457249071
ensp00000376436	ets1	4429	0.457352334
ensp00000327048	maf	4430	0.457455597
ensp00000321410	mapk9	4431	0.45755886
ensp00000172229	ngfr	4432	0.457662123
ensp00000358525	ngf	4433	0.457765386
ensp00000368884	rps6ka3	4435	0.457971912
ensp00000386050	rps6ka2	4436	0.458075176
ensp00000327850	nfatc1	4437	0.458178439

ensp00000379330	nfatc2	4438	0.458281702
ensp00000300659	nfatc3	4439	0.458384965
ensp00000396538	nfat5	4440	0.458488228
ensp00000388910	nfatc4	4441	0.458591491
ensp00000419425	ppia	4442	0.458694754
ensp00000259075	tank	4443	0.458798017
ensp00000401980	mavs	4444	0.45890128
ensp00000363998	itch	4445	0.459004544
ensp00000345530	nedd4	4446	0.459107807
ensp00000265428	wwp1	4447	0.45921107
ensp00000332468	traf3	4449	0.459417596
ensp00000329967	tbk1	4450	0.459520859
ensp00000356087	ikbke	4451	0.459624122
ensp00000261991	rps6ka5	4452	0.459727385
ensp00000333896	rps6ka4	4453	0.459830648
ensp00000262752	rps6ka6	4454	0.459933912
ensp00000311005	mapk7	4455	0.460037175
ensp00000338185	plcb1	4457	0.460243701
ensp00000260402	plcb2	4458	0.460346964
ensp00000354927	map3k3	4459	0.460450227
ensp00000331288	tmem173	4460	0.46055349
ensp00000308928	cyld	4461	0.460656753
ensp00000367830	prkcz	4462	0.460760017
ensp00000219255	pard6a	4463	0.46086328
ensp00000347169	numb	4464	0.460966543
ensp00000309968	adam17	4465	0.461069806
ensp00000271889	creb3l4	4467	0.461276332
ensp00000387699	creb1	4468	0.461379595
ensp00000359573	lmo4	4469	0.461482858
ensp00000258743	il6	4470	0.461586121
ensp00000357470	il6r	4471	0.461689385
ensp00000178640	map2k5	4472	0.461792648
ensp00000357306	rit1	4473	0.461895911
ensp00000321805	rit2	4474	0.461999174
ensp00000334329	unc5b	4476	0.4622057
ensp00000332737	unc5a	4478	0.462412226
ensp00000383303	dscam	4479	0.462515489
ensp00000370503	ccm2	4480	0.462618753
ensp00000226004	dusp3	4482	0.462825279
ensp00000282753	grm1	4484	0.463031805
ensp00000356529	rgs16	4485	0.463135068
ensp00000200457	trip6	4486	0.463238331
ensp00000245919	fosb	4487	0.463341594
ensp00000352157	mapk10	4488	0.463444857
ensp00000353483	mapk8	4489	0.463548121
ensp00000400717	gna13	4491	0.463754647
ensp00000275364	gna12	4492	0.46385791

ensp00000267079	map3k12	4493	0.463961173
ensp00000250894	mapk8ip3	4494	0.464064436
ensp00000347979	fas	4495	0.464167699
ensp00000228534	il23a	4497	0.464374226
ensp00000384053	csf2rb	4499	0.464580752
ensp00000309597	map3k11	4501	0.464787278
ensp00000241014	mapk8ip1	4502	0.464890541
ensp00000381066	map2k7	4503	0.464993804
ensp00000345083	map2k3	4504	0.465097067
ensp00000351997	map2k6	4505	0.46520033
ensp00000375986	map3k4	4506	0.465303594
ensp00000339428	socs2	4507	0.465406857
ensp00000341327	socs4	4508	0.46551012
ensp00000300134	stat6	4509	0.465613383
ensp00000351255	stat4	4510	0.465716646
ensp00000293328	stat5b	4511	0.465819909
ensp00000263102	ccdc6	4512	0.465923172
ensp00000395465	ncoa4	4513	0.466026435
ensp00000299767	hsp90b1	4514	0.466129698
ensp00000265986	ide	4517	0.466439488
ensp00000263904	stam2	4518	0.466542751
ensp00000366746	stam	4519	0.466646014
ensp00000331201	hgs	4520	0.466749277
ensp00000362014	dnm1	4521	0.46685254
ensp00000366244	araf	4522	0.466955803
ensp00000288602	braf	4523	0.467059067
ensp00000251849	raf1	4524	0.46716233
ensp00000302486	map2k1	4525	0.467265593
ensp00000262445	map2k4	4526	0.467368856
ensp00000256707	kidins220	4528	0.467575382
ensp00000262345	il12rb2	4529	0.467678645
ensp00000303231	il12a	4530	0.467781908
ensp00000314425	il12rb1	4532	0.467988435
ensp00000264818	tyk2	4533	0.468091698
ensp00000296545	il15	4535	0.468298224
ensp00000264497	il21	4536	0.468401487
ensp00000319060	camk2g	4538	0.468608013
ensp00000339740	camk2d	4539	0.468711276
ensp00000258682	camk2b	4540	0.468814539
ensp00000315615	akap5	4547	0.469537381
ensp00000268182	iqgap1	4548	0.469640644
ensp00000400010	ptprj	4549	0.469743907
ensp00000344115	cdh5	4550	0.469847171
ensp00000353656	cdh4	4551	0.469950434
ensp00000264463	cdh10	4552	0.470053697
ensp00000268603	cdh11	4556	0.470466749
ensp00000268613	cdh13	4557	0.470570012

ensp00000286364	rasa2	4559	0.470776539
ensp00000310722	mos	4560	0.470879802
ensp00000174621	trpv1	4561	0.470983065
ensp00000296871	csf2	4562	0.471086328
ensp00000370935	csf2ra	4563	0.471189591
ensp00000289153	pik3cb	4564	0.471292854
ensp00000222254	pik3r2	4565	0.471396117
ensp00000262741	pik3r3	4566	0.47149938
ensp00000366563	pik3cd	4569	0.47180917
ensp00000313829	khdrbs1	4570	0.471912433
ensp00000288840	smad6	4572	0.472118959
ensp00000354621	smurf1	4573	0.472222222
ensp00000339191	cav1	4574	0.472325485
ensp00000360683	ptpn1	4575	0.472428748
ensp00000265071	cdh6	4576	0.472532012
ensp00000267383	cdh24	4578	0.472738538
ensp00000289746	cdh15	4579	0.472841801
ensp00000267859	bnip2	4580	0.472945064
ensp00000262013	spag9	4581	0.473048327
ensp00000263577	cdon	4582	0.47315159
ensp00000330341	socs3	4583	0.473254853
ensp00000329418	socs1	4584	0.473358116
ensp00000339804	tslp	4588	0.473771169
ensp00000263851	il7	4589	0.473874432
ensp00000338010	il21r	4590	0.473977695
ensp00000330393	lepr	4591	0.474080958
ensp00000243213	il13ra2	4592	0.474184221
ensp00000360730	il13ra1	4593	0.474287485
ensp00000244174	il9r	4594	0.474390748
ensp00000369312	il15ra	4595	0.474494011
ensp00000306157	il7r	4596	0.474597274
ensp00000370979	crlf2	4597	0.474700537
ensp00000263087	itgae	4598	0.4748038
ensp00000311113	jup	4599	0.474907063
ensp00000382004	ctnnd1	4600	0.475010326
ensp00000304669	ctnna1	4601	0.475113589
ensp00000269141	cdh2	4602	0.475216853
ensp00000355398	ctnna2	4603	0.475320116
ensp00000397297	ntf3	4604	0.475423379
ensp00000301411	ntf4	4605	0.475526642
ensp00000366729	tnfrsf9	4606	0.475629905
ensp00000328207	tnfrsf18	4607	0.475733168
ensp00000368124	camk1d	4609	0.475939694
ensp00000355370	cntf	4610	0.476042957
ensp00000215781	osm	4611	0.476146221
ensp00000330572	mapk8ip2	4612	0.476249484
ensp00000340698	gipc1	4613	0.476352747

ensp00000277120	ntrk2	4614	0.47645601
ensp00000414303	bdnf	4615	0.476559273
ensp00000313644	map4k4	4616	0.476662536
ensp00000295400	tgfa	4617	0.476765799
ensp00000249075	lif	4618	0.476869062
ensp00000365380	foxp3	4619	0.476972325
ensp00000270800	il22ra1	4621	0.477178852
ensp00000225275	mpo	4624	0.477488641
ensp00000216341	gzmb	4626	0.477695167
ensp00000274276	osmr	4627	0.47779843
ensp00000242338	cntfr	4628	0.477901694
ensp00000263409	lifr	4629	0.478004957
ensp00000338799	il6st	4630	0.47810822
ensp00000326500	il11ra	4631	0.478211483
ensp00000310406	rin1	4632	0.478314746
ensp00000343463	map3k2	4634	0.478521272
ensp00000320176	hcls1	4635	0.478624535
ensp00000263379	il27ra	4637	0.478831062
ensp00000221847	ebi3	4638	0.478934325
ensp00000409346	cish	4639	0.479037588
ensp00000328133	il20rb	4640	0.479140851
ensp00000227752	il10ra	4641	0.479244114
ensp00000361548	mpl	4642	0.479347377
ensp00000352358	trpv6	4644	0.479553903
ensp00000394794	ptpn13	4645	0.479657166
ensp00000237380	med28	4646	0.47976043
ensp00000357182	sh2d2a	4647	0.479863693
ensp00000353508	map2	4648	0.479966956
ensp00000289422	nrg2	4649	0.480070219
ensp00000349275	nrg1	4650	0.480173482
ensp00000244869	ereg	4651	0.480276745
ensp00000307387	pdcd6ip	4652	0.480380008
ensp00000342755	rnf41	4653	0.480483271
ensp00000233668	dok1	4655	0.480689798
ensp00000349477	klrg1	4656	0.480793061
ensp00000281092	fer	4657	0.480896324
ensp00000370912	tec	4658	0.480999587
ensp00000331504	fes	4659	0.48110285
ensp00000305133	socs5	4660	0.481206113
ensp00000330659	socs7	4661	0.481309376
ensp00000329127	prkch	4662	0.481412639
ensp00000306124	prkce	4663	0.481515903
ensp00000263125	prkcq	4664	0.481619166
ensp00000348278	stk39	4666	0.481825692
ensp00000273261	lrig1	4667	0.481928955
ensp00000230990	hbegf	4668	0.482032218
ensp00000332049	cd86	4673	0.482548534

ensp00000216223	il2rb	4674	0.482651797
ensp00000365016	irs2	4678	0.483064849
ensp00000222139	epor	4682	0.483477902
ensp00000288135	kit	4683	0.483581165
ensp00000228280	kitlg	4684	0.483684428
ensp00000356705	dnm3	4687	0.483994217
ensp00000266481	dnm1l	4688	0.48409748
ensp00000286301	csf1r	4690	0.484304007
ensp00000263923	kdr	4692	0.484510533
ensp00000342235	erbb4	4694	0.484717059
ensp00000269571	erbb2	4695	0.484820322
ensp00000222005	cdc37	4696	0.484923585
ensp00000299293	frs2	4697	0.485026848
ensp00000348099	pdlim7	4706	0.485956216
ensp00000337163	sh2b1	4707	0.48605948
ensp00000345492	sh2b3	4708	0.486162743
ensp00000290200	il10rb	4709	0.486266006
ensp00000356713	ifngr1	4711	0.486472532
ensp00000290219	ifngr2	4712	0.486575795
ensp00000270139	ifnar1	4713	0.486679058
ensp00000343957	ifnar2	4714	0.486782321
ensp00000226091	efnb3	4715	0.486885584
ensp00000245323	efnb2	4716	0.486988848
ensp00000297450	angpt1	4717	0.487092111
ensp00000381097	ephb1	4719	0.487298637
ensp00000204961	efnb1	4720	0.4874019
ensp00000363763	ephb2	4721	0.487505163
ensp00000350896	ephb4	4722	0.487608426
ensp00000376684	ephb6	4723	0.487711689
ensp00000284154	grap	4724	0.487814952
ensp00000310771	grb7	4725	0.487918216
ensp00000372160	dok6	4726	0.488021479
ensp00000366306	spry2	4727	0.488124742
ensp00000380921	sh3kbp1	4728	0.488228005
ensp00000264033	cbl	4729	0.488331268
ensp00000264122	cblb	4730	0.488434531
ensp00000270279	cblc	4731	0.488537794
ensp00000324890	cd28	4732	0.488641057
ensp00000352208	myof	4733	0.488744321
ensp00000260404	pak6	4735	0.488950847
ensp00000353864	pak3	4736	0.48905411
ensp00000278568	pak1	4737	0.489157373
ensp00000354207	ntrk3	4738	0.489260636
ensp00000368314	nrcam	4739	0.489363899
ensp00000263373	sptbn4	4740	0.489467162
ensp00000374372	sptb	4741	0.489570425
ensp00000349259	sptbn1	4742	0.489673689

ensp00000256078	kras	4743	0.489776952
ensp00000358548	nras	4744	0.489880215
ensp00000309845	hras	4745	0.489983478
ensp00000274376	rasa1	4746	0.490086741
ensp00000333568	prkd1	4747	0.490190004
ensp00000368759	nedd9	4748	0.490293267
ensp00000371341	tnk2	4750	0.490499793
ensp00000267101	erbb3	4751	0.490603057
ensp00000384675	sos1	4752	0.49070632
ensp00000401303	shc1	4754	0.490912846
ensp00000295006	capn2	4756	0.491119372
ensp00000230882	ghr	4757	0.491222635
ensp00000371432	prlr	4758	0.491325898
ensp00000371067	jak2	4759	0.491429162
ensp00000391676	jak3	4760	0.491532425
ensp00000343204	jak1	4761	0.491635688
ensp00000170630	il4r	4762	0.491738951
ensp00000393725	gfra1	4763	0.491842214
ensp00000317145	gdnf	4764	0.491945477
ensp00000288986	nck1	4765	0.49204874
ensp00000348461	rac1	4766	0.492152003
ensp00000314458	cdc42	4767	0.492255266
ensp00000361423	abl1	4768	0.49235853
ensp00000256925	cables1	4769	0.492461793
ensp00000364893	arhgef7	4770	0.492565056
ensp00000378338	git1	4771	0.492668319
ensp00000300574	crk	4772	0.492771582
ensp00000346300	crkl	4773	0.492874845
ensp00000341189	ptk2	4774	0.492978108
ensp00000220003	csk	4775	0.493081371
ensp00000162330	bcar1	4776	0.493184634
ensp00000304895	irs1	4778	0.493391161
ensp00000304701	sh2b2	4779	0.493494424
ensp00000381793	grb10	4780	0.493597687
ensp00000347464	git2	4781	0.49370095
ensp00000332816	ptk2b	4782	0.493804213
ensp00000299421	ilk	4783	0.493907476
ensp00000324740	yes1	4784	0.494010739
ensp00000357656	fyn	4785	0.494114002
ensp00000337825	lck	4786	0.494217266
ensp00000011653	cd4	4787	0.494320529
ensp00000314067	pak2	4788	0.494423792
ensp00000351049	pak4	4789	0.494527055
ensp00000347942	ret	4790	0.494630318
ensp00000410294	fgfr2	4791	0.494733581
ensp00000317272	met	4792	0.494836844
ensp00000268035	igf1r	4793	0.494940107

ensp00000350941	src	4794	0.495043371
ensp00000284384	prkca	4795	0.495146634
ensp00000331602	prkcd	4796	0.495249897
ensp00000296414	dapp1	4797	0.49535316
ensp00000224337	blnk	4798	0.495456423
ensp00000221972	cd79a	4800	0.495662949
ensp00000391592	ptpn6	4801	0.495766212
ensp00000340944	ptpn11	4802	0.495869475
ensp00000352575	inpp5d	4803	0.495972739
ensp00000359892	slc44a5	4804	0.496076002
ensp00000351209	epha2	4805	0.496179265
ensp00000357392	efna1	4806	0.496282528
ensp00000281821	epha4	4807	0.496385791
ensp00000328777	efna5	4808	0.496489054
ensp00000358309	epha7	4809	0.496592317
ensp00000273854	epha5	4810	0.49669558
ensp00000275815	epha1	4813	0.49700537
ensp00000215368	efna2	4815	0.497211896
ensp00000361264	rapgef1	4816	0.497315159
ensp00000339826	pik3ap1	4817	0.497418422
ensp00000313419	cd19	4818	0.497521685
ensp00000256196	rras2	4820	0.497728211
ensp00000246792	rras	4821	0.497831475
ensp00000289104	mras	4822	0.497934738
ensp00000269554	pip4k2b	4825	0.498244527
ensp00000365757	pip4k2a	4826	0.49834779
ensp00000347032	pip4k2c	4827	0.498451053
ensp00000264316	txk	4828	0.498554316
ensp00000262995	gab1	4829	0.49865758
ensp00000354119	lat	4830	0.498760843
ensp00000302269	vav1	4831	0.498864106
ensp00000360916	vav2	4832	0.498967369
ensp00000289902	fcer1g	4835	0.499277158
ensp00000364898	syk	4836	0.499380421
ensp00000363115	fgr	4838	0.499586948
ensp00000264051	ngef	4841	0.499896737
ensp00000393511	ablim2	4842	0.5
ensp00000357393	efna3	4843	0.500103263
ensp00000352138	kirrel	4844	0.500206526
ensp00000368190	nphs1	4845	0.500309789
ensp00000264554	shc2	4846	0.500413052
ensp00000364995	shc3	4847	0.500516316
ensp00000329668	shc4	4848	0.500619579
ensp00000274071	pdgfc	4849	0.500722842
ensp00000346508	pdgfa	4850	0.500826105
ensp00000238607	pgf	4851	0.500929368
ensp00000330382	pdgfb	4852	0.501032631

ensp00000261799	pdgfrb	4853	0.501135894
ensp00000380280	fgfr1	4854	0.501239157
ensp00000352721	dnm2	4855	0.50134242
ensp00000173229	ntn1	4856	0.501445684
ensp00000406022	unc5c	4858	0.50165221
ensp00000311127	vegfb	4859	0.501755473
ensp00000327513	csf1	4861	0.501961999
ensp00000359073	vav3	4862	0.502065262
ensp00000354566	cd3e	4865	0.502375052
ensp00000300692	cd3d	4866	0.502478315
ensp00000283635	cd8a	4868	0.502684841
ensp00000256935	dock2	4871	0.50299463
ensp00000312185	elmo1	4872	0.503097893
ensp00000310309	ablim3	4875	0.503407683
ensp00000292408	fgfr4	4876	0.503510946
ensp00000260795	fgfr3	4877	0.503614209
ensp00000257290	pdgfra	4878	0.503717472
ensp00000261937	flt4	4879	0.503820735
ensp00000376865	pdgfd	4880	0.503923998
ensp00000269886	sh3gl1	4882	0.504130525
ensp00000361125	vegfa	4883	0.504233788
ensp00000341208	stat5a	4884	0.504337051
ensp00000315768	stat2	4885	0.504440314
ensp00000361021	pten	4888	0.504750103
ensp00000360154	ocrl	4889	0.504853366
ensp00000360777	inpp5e	4890	0.504956629
ensp00000373399	pip5kl1	4891	0.505059893
ensp00000366936	shb	4892	0.505163156
ensp00000404179	dock4	4893	0.505266419
ensp00000301843	cttn	4894	0.505369682
ensp00000353030	ptprf	4895	0.505472945
ensp00000318486	cdk5r1	4896	0.505576208
ensp00000297518	cdk5	4897	0.505679471
ensp00000370719	itsn1	4898	0.505782734
ensp00000240874	kalrn	4899	0.505885998
ensp00000405934	itpr1	4900	0.505989261
ensp00000363435	itpr3	4901	0.506092524
ensp00000370744	itpr2	4902	0.506195787
ensp00000260386	itpka	4903	0.50629905
ensp00000272117	itpkb	4904	0.506402313
ensp00000263370	itpkc	4905	0.506505576
ensp00000363046	ipmk	4906	0.506608839
ensp00000267615	itpk1	4907	0.506712102
ensp00000345988	plch1	4908	0.506815366
ensp00000348685	sh3bp2	4910	0.507021892
ensp00000220597	pag1	4911	0.507125155
ensp00000380066	map4k1	4914	0.507434944

ensp00000262866	sla2	4916	0.50764147
ensp00000321606	crmp1	4917	0.507744734
ensp00000339850	dpysl4	4918	0.507847997
ensp00000381526	dpysl3	4920	0.508054523
ensp00000357583	inpp5a	4921	0.508157786
ensp00000298229	inppl1	4922	0.508261049
ensp00000384534	inpp5j	4923	0.508364312
ensp00000254712	inpp5k	4924	0.508467575
ensp00000325423	inpp1	4925	0.508570838
ensp00000408526	impa1	4926	0.508674102
ensp00000269159	impa2	4927	0.508777365
ensp00000262848	prkx	4928	0.508880628
ensp00000359719	prkacb	4931	0.509190417
ensp00000248594	ptpn12	4932	0.50929368
ensp00000303507	bcr	4933	0.509396943
ensp00000216373	sos2	4935	0.50960347
ensp00000250617	arhgef6	4936	0.509706733
ensp00000336740	limk1	4937	0.509809996
ensp00000361824	sptan1	4938	0.509913259
ensp00000311489	sptbn2	4939	0.510016522
ensp00000265062	rab7a	4940	0.510119785
ensp00000303830	insr	4941	0.510223048
ensp00000233154	nck2	4943	0.510429575
ensp00000223023	wasl	4944	0.510532838
ensp00000365891	was	4945	0.510636101
ensp00000362924	gsn	4946	0.510739364
ensp00000338345	snca	4947	0.510842627
ensp00000356587	nphs2	4949	0.511049153
ensp00000352802	wipf1	4950	0.511152416
ensp00000085219	cd22	4952	0.511358943
ensp00000263674	arhgef17	4953	0.511462206
ensp00000253401	arhgef9	4954	0.511565469
ensp00000364277	fgd1	4955	0.511668732
ensp00000364754	mcf2l	4956	0.511771995
ensp00000286827	tiam1	4957	0.511875258
ensp00000249071	rac2	4958	0.511978521
ensp00000357177	arhgef11	4959	0.512081784
ensp00000315325	arhgef2	4960	0.512185048
ensp00000269321	arhgdia	4961	0.512288311
ensp00000228945	arhgdib	4962	0.512391574
ensp00000250559	rap1b	4963	0.512494837
ensp00000348786	rap1a	4964	0.5125981
ensp00000265036	depdc1b	4965	0.512701363
ensp00000232458	ect2	4966	0.512804626
ensp00000412292	depdc1	4967	0.512907889
ensp00000368914	pstpip1	4968	0.513011152
ensp00000367747	plch2	4969	0.513114416

ensp00000361064	minpp1	4970	0.513217679
ensp00000255882	pi4ka	4972	0.513424205
ensp00000219789	cdipt	4973	0.513527468
ensp00000320543	epn2	4974	0.513630731
ensp00000268933	epn3	4975	0.513733994
ensp00000391372	sh3gl3	4976	0.513837257
ensp00000369981	sh3gl2	4977	0.51394052
ensp00000360798	eps15	4979	0.514147047
ensp00000248070	eps15l1	4980	0.51425031
ensp00000343785	spry1	4981	0.514353573
ensp00000339299	trio	4982	0.514456836
ensp00000309539	dpysl2	4984	0.514663362
ensp00000264042	farp2	4985	0.514766625
ensp00000358696	plxna3	4986	0.514869888
ensp00000265362	sema3a	4987	0.514973152
ensp00000251772	plxna1	4988	0.515076415
ensp00000356000	plxna2	4989	0.515179678
ensp00000323194	plxna4	4990	0.515282941
ensp00000352264	cd2ap	4991	0.515386204
ensp00000304283	rac3	4992	0.515489467
ensp00000322234	synj1	4993	0.51559273
ensp00000347792	synj2	4994	0.515695993
ensp00000362115	inpp5b	4995	0.515799257
ensp00000337261	arhgef1	4996	0.51590252
ensp00000371897	arhgap5	4997	0.516005783
ensp00000310491	arhgap1	5000	0.516315572
ensp00000347710	ophn1	5001	0.516418835
ensp00000019317	ralbp1	5002	0.516522098
ensp00000394071	gdi1	5005	0.516831888
ensp00000308576	rhod	5007	0.517038414
ensp00000203786	arhgap4	5008	0.517141677
ensp00000272233	rhob	5009	0.51724494
ensp00000339467	rhog	5010	0.517348203
ensp00000303242	itgb2	5011	0.517451466
ensp00000287497	itgam	5012	0.517554729
ensp00000335333	pip5k1c	5013	0.517657993
ensp00000359665	pi4k2a	5015	0.517864519
ensp00000264864	pi4k2b	5016	0.517967782
ensp00000275493	egfr	5017	0.518071045
ensp00000265171	egf	5018	0.518174308
ensp00000311857	ptpn2	5020	0.518380834
ensp00000373301	sh3bp5	5021	0.518484097
ensp00000305721	ibtk	5022	0.518587361
ensp00000259633	cd72	5023	0.518690624
ensp00000358490	cd2	5024	0.518793887
ensp00000216338	gzmh	5025	0.51889715
ensp00000380942	arhgef12	5026	0.519000413

ensp00000308461	rnd1	5027	0.519103676
ensp00000343418	sema4d	5028	0.519206939
ensp00000365301	fgf14	5029	0.519310202
ensp00000274625	fgf18	5031	0.519516729
ensp00000180166	fgf20	5032	0.519619992
ensp00000267843	fgf7	5036	0.520033044
ensp00000371790	fgf9	5037	0.520136307
ensp00000334122	fgf3	5038	0.52023957
ensp00000381102	tmem55b	5040	0.520446097
ensp00000285419	tmem55a	5041	0.52054936
ensp00000340913	trpc6	5042	0.520652623
ensp00000322390	fgf13	5044	0.520859149
ensp00000266376	cacna1c	5045	0.520962412
ensp00000263269	grin2d	5046	0.521065675
ensp00000293190	grin2c	5047	0.521168938
ensp00000316772	hmha1	5049	0.521375465
ensp00000274498	arhgap26	5051	0.521581991
ensp00000356992	arhgap30	5052	0.521685254
ensp00000327251	nos2	5053	0.521788517
ensp00000320758	nos1	5054	0.52189178
ensp00000297494	nos3	5055	0.521995043
ensp00000318921	nostrin	5056	0.522098306
ensp00000354519	inpp5f	5057	0.52220157
ensp00000262039	pik3c3	5058	0.522304833
ensp00000337168	epb41l1	5059	0.522408096
ensp00000262138	cacng4	5060	0.522511359
ensp00000005284	cacng3	5062	0.522717885
ensp00000361120	ralgds	5064	0.522924411
ensp00000264431	rapgef2	5065	0.523027675
ensp00000383623	mllt4	5066	0.523130938
ensp00000310244	rasgrp1	5067	0.523234201
ensp00000405963	rasgrf1	5069	0.523440727
ensp00000265080	rasgrf2	5070	0.52354399
ensp00000386733	plekhg2	5071	0.523647253
ensp00000219409	arhgdig	5072	0.523750516
ensp00000220507	rhov	5073	0.523853779
ensp00000251822	rhobtb2	5074	0.523957043
ensp00000267205	rhof	5075	0.524060306
ensp00000371219	rhoh	5076	0.524163569
ensp00000338671	rhobtb1	5077	0.524266832
ensp00000355652	rhou	5078	0.524370095
ensp00000238738	rhoq	5079	0.524473358
ensp00000340594	syde2	5081	0.524679884
ensp00000290974	zfyve28	5082	0.524783147
ensp00000338548	fgf1	5084	0.524989674
ensp00000315713	ssh1	5085	0.525092937
ensp00000356346	ptprc	5086	0.5251962

ensp00000373713	sacm1l	5090	0.525609252
ensp00000262992	inpp4b	5091	0.525712515
ensp00000074304	inpp4a	5092	0.525815779
ensp00000271657	pi4kb	5093	0.525919042
ensp00000265970	pik3c2a	5094	0.526022305
ensp00000356155	pik3c2b	5095	0.526125568
ensp00000244007	plcg1	5096	0.526228831
ensp00000352336	plcg2	5097	0.526332094
ensp00000260766	plce1	5098	0.526435357
ensp00000313731	plcd3	5099	0.52653862
ensp00000388631	plcd4	5101	0.526745147
ensp00000309757	lpl	5103	0.526951673
ensp00000313681	sphk1	5104	0.527054936
ensp00000245222	sphk2	5105	0.527158199
ensp00000330276	ptpn21	5106	0.527261462
ensp00000257017	rab33a	5107	0.527364725
ensp00000249601	arhgap22	5109	0.527571252
ensp00000363727	stard8	5111	0.527777778
ensp00000203556	gmip	5112	0.527881041
ensp00000351333	arhgap19	5113	0.527984304
ensp00000239440	arap3	5115	0.52819083
ensp00000302895	arap2	5116	0.528294093
ensp00000033079	fam13b	5117	0.528397356
ensp00000260526	arhgap29	5118	0.52850062
ensp00000348349	myo9a	5119	0.528603883
ensp00000277575	usp6nl	5120	0.528707146
ensp00000347443	rassf5	5121	0.528810409
ensp00000354609	cnksr1	5122	0.528913672
ensp00000368966	trpc3	5123	0.529016935
ensp00000369003	trpc4	5124	0.529120198
ensp00000314214	vamp2	5126	0.529326724
ensp00000249647	snap23	5127	0.529429988
ensp00000317714	stx4	5128	0.529533251
ensp00000365530	stxbp4	5129	0.529636514
ensp00000011619	ranbp9	5130	0.529739777
ensp00000316589	ranbp10	5131	0.52984304
ensp00000293829	fgf11	5132	0.529946303
ensp00000413496	fgf12	5134	0.530152829
ensp00000311697	fgf5	5136	0.530359356
ensp00000264498	fgf2	5138	0.530565882
ensp00000338934	ezr	5140	0.530772408
ensp00000353408	msn	5141	0.530875671
ensp00000366977	plekhg5	5142	0.530978934
ensp00000352995	arhgef18	5143	0.531082197
ensp00000271636	cgn	5144	0.531185461
ensp00000357624	marcks	5146	0.531391987
ensp00000376024	snx9	5147	0.53149525

ensp00000228938	mgp	5149	0.531701776
ensp00000361405	mmp9	5150	0.531805039
ensp00000219070	mmp2	5151	0.531908302
ensp00000352011	cacna1g	5152	0.532011565
ensp00000377840	cacnb1	5153	0.532114829
ensp00000332549	grin2a	5156	0.532424618
ensp00000391106	myo10	5158	0.532631144
ensp00000263980	slc9a1	5159	0.532734407
ensp00000362057	nox1	5162	0.533044197
ensp00000342830	rdx	5165	0.533353986
ensp00000317985	rock2	5167	0.533560512
ensp00000237500	myl12b	5168	0.533663775
ensp00000222212	cacng7	5170	0.533870302
ensp00000349320	cacna2d1	5171	0.533973565
ensp00000252729	cacng6	5172	0.534076828
ensp00000288139	cacna1d	5174	0.534283354
ensp00000301050	cacnb3	5175	0.534386617
ensp00000374467	abcc8	5176	0.53448988
ensp00000280193	vegfc	5179	0.53479967
ensp00000315130	clu	5181	0.535006196
ensp00000348888	pigr	5184	0.535315985
ensp00000317310	cabp1	5185	0.535419248
ensp00000416463	rasgrp4	5186	0.535522511
ensp00000338864	rasgrp2	5187	0.535625774
ensp00000372169	cacna2d4	5193	0.536245353
ensp00000288197	cacna2d3	5194	0.536348616
ensp00000390329	cacna2d2	5195	0.536451879
ensp00000355192	cacna1s	5197	0.536658406
ensp00000385019	cacna1i	5198	0.536761669
ensp00000334198	cacna1h	5199	0.536864932
ensp00000356545	cacna1e	5200	0.536968195
ensp00000384651	mtmr3	5202	0.537174721
ensp00000296003	mtmr14	5203	0.537277984
ensp00000359423	mtm1	5204	0.537381247
ensp00000325285	mtmr4	5205	0.537484511
ensp00000281172	eps8	5206	0.537587774
ensp00000316338	baiap2	5208	0.5377943
ensp00000354451	iqgap3	5209	0.537897563
ensp00000274364	iqgap2	5210	0.538000826
ensp00000383178	diaph3	5211	0.538104089
ensp00000369962	igsf5	5212	0.538207352
ensp00000420419	jam2	5213	0.538310615
ensp00000299106	jam3	5214	0.538413879
ensp00000305355	prkcb	5216	0.538620405
ensp00000298316	arf6	5217	0.538723668
ensp00000408236	cyth2	5218	0.538826931
ensp00000272519	ralb	5219	0.538930194

ensp00000005257	rala	5220	0.539033457
ensp00000353564	exoc8	5221	0.53913672
ensp00000382177	myo5a	5222	0.539239983
ensp00000359025	stxbp3	5223	0.539343247
ensp00000302176	aspscr1	5224	0.53944651
ensp00000261017	abi2	5225	0.539549773
ensp00000396211	wasf2	5227	0.539756299
ensp00000352425	wasf1	5228	0.539859562
ensp00000316845	arhgef4	5229	0.539962825
ensp00000390948	cyfip2	5232	0.540272615
ensp00000343325	pkn1	5234	0.540479141
ensp00000334100	exoc7	5235	0.540582404
ensp00000389934	exoc5	5236	0.540685667
ensp00000260762	exoc6	5237	0.54078893
ensp00000253861	exoc4	5238	0.540892193
ensp00000230449	exoc2	5239	0.540995456
ensp00000228825	arpc3	5241	0.541201983
ensp00000295685	arpc2	5242	0.541305246
ensp00000380431	arpc4	5243	0.541408509
ensp00000362946	rab14	5244	0.541511772
ensp00000357564	rab13	5245	0.541615035
ensp00000300935	rab8a	5246	0.541718298
ensp00000268638	irf8	5248	0.541924824
ensp00000310127	irf3	5249	0.542028088
ensp00000329411	irf7	5250	0.542131351
ensp00000349770	irf5	5251	0.542234614
ensp00000239938	egr1	5252	0.542337877
ensp00000308208	mmp14	5253	0.54244114
ensp00000289473	ncf1	5254	0.542544403
ensp00000242465	srgn	5255	0.542647666
ensp00000218388	timp1	5256	0.542750929
ensp00000332139	cfd	5257	0.542854192
ensp00000321853	serpinf2	5259	0.543060719
ensp00000243077	lrp1	5260	0.543163982
ensp00000408958	tom1l1	5261	0.543267245
ensp00000383392	ncam2	5262	0.543370508
ensp00000303634	lrp8	5264	0.543577034
ensp00000392423	reln	5265	0.543680297
ensp00000371532	vldlr	5267	0.543886824
ensp00000360869	ifit1	5268	0.543990087
ensp00000368699	isg15	5269	0.54409335
ensp00000264350	herc5	5270	0.544196613
ensp00000287156	ube2l6	5271	0.544299876
ensp00000369217	arih1	5272	0.544403139
ensp00000342848	noxa1	5274	0.544609665
ensp00000282412	ppm1b	5275	0.544712929
ensp00000296440	plxnb1	5276	0.544816192

ensp00000352288	plxnb2	5277	0.544919455
ensp00000335055	wasf3	5278	0.545022718
ensp00000356505	ncf2	5279	0.545125981
ensp00000264832	icam1	5283	0.545539033
ensp00000294728	vcam1	5284	0.545642297
ensp00000318472	ncam1	5285	0.54574556
ensp00000252725	arpc1b	5286	0.545848823
ensp00000352918	arpc5	5287	0.545952086
ensp00000263238	actr3	5288	0.546055349
ensp00000316333	cd55	5289	0.546158612
ensp00000386187	ifitm1	5290	0.546261875
ensp00000298902	ifi27	5291	0.546365138
ensp00000355988	irf6	5292	0.546468401
ensp00000223364	myl7	5295	0.546778191
ensp00000383023	myl5	5297	0.546984717
ensp00000325239	mylpf	5298	0.54708798
ensp00000217652	myl12a	5299	0.547191243
ensp00000279022	myl9	5300	0.547294506
ensp00000307513	mrc2	5302	0.547501033
ensp00000283243	pla2r1	5303	0.547604296
ensp00000365152	mylk2	5304	0.547707559
ensp00000353452	mylk	5306	0.547914085
ensp00000339916	limk2	5307	0.548017348
ensp00000349960	actb	5309	0.548223874
ensp00000331514	actg1	5310	0.548327138
ensp00000225655	pfn1	5311	0.548430401
ensp00000348068	serpina1	5312	0.548533664
ensp00000355896	tgfb2	5313	0.548636927
ensp00000238682	tgfb3	5314	0.54874019
ensp00000231061	sparc	5315	0.548843453
ensp00000331831	gas6	5316	0.548946716
ensp00000220809	plat	5318	0.549153242
ensp00000367220	actr2	5319	0.549256506
ensp00000262942	arpc1a	5320	0.549359769
ensp00000259477	arpc5l	5321	0.549463032
ensp00000343023	sp100	5322	0.549566295
ensp00000359497	gbp2	5323	0.549669558
ensp00000316328	ciita	5324	0.549772821
ensp00000380073	irf9	5325	0.549876084
ensp00000245414	irf1	5326	0.549979347
ensp00000377218	irf2	5327	0.55008261
ensp00000293599	aqp5	5332	0.550598926
ensp00000297991	aqp3	5333	0.550702189
ensp00000346151	magi2	5334	0.550805452
ensp00000385450	magi1	5335	0.550908715
ensp00000407879	myh14	5336	0.551011979
ensp00000269243	myh10	5337	0.551115242

ensp00000216181	myh9	5338	0.551218505
ensp00000356239	ppp1r12b	5339	0.551321768
ensp00000297029	scin	5340	0.551425031
ensp00000377941	actn1	5341	0.551528294
ensp00000355537	actn2	5342	0.551631557
ensp00000252699	actn4	5343	0.55173482
ensp00000271450	fcgr2a	5344	0.551838083
ensp00000371471	rsad2	5347	0.552147873
ensp00000359783	ifi44	5348	0.552251136
ensp00000335062	pdcd1	5349	0.552354399
ensp00000353099	hla-drb1	5352	0.552664188
ensp00000364114	hla-drb5	5353	0.552767451
ensp00000384886	ifi30	5354	0.552870715
ensp00000359504	gbp1	5355	0.552973978
ensp00000357459	adar	5357	0.553180504
ensp00000342278	oas2	5358	0.553283767
ensp00000228928	oas3	5359	0.55338703
ensp00000388001	oas1	5360	0.553490293
ensp00000257570	oasl	5361	0.553593556
ensp00000360876	ifit3	5362	0.553696819
ensp00000354822	xaf1	5363	0.553800083
ensp00000360891	ifit2	5364	0.553903346
ensp00000381599	mx1	5365	0.554006609
ensp00000342513	ifi6	5366	0.554109872
ensp00000364398	habp4	5367	0.554213135
ensp00000211998	vcl	5368	0.554316398
ensp00000316029	tln1	5369	0.554419661
ensp00000358866	flna	5370	0.554522924
ensp00000359787	ifi44l	5371	0.554626188
ensp00000381565	diaph1	5372	0.554729451
ensp00000321348	diaph2	5373	0.554832714
ensp00000215539	igfals	5374	0.554935977
ensp00000324422	zyx	5376	0.555142503
ensp00000323377	exoc3	5377	0.555245766
ensp00000276202	dock11	5378	0.555349029
ensp00000294618	dock6	5379	0.555452292
ensp00000365643	dock9	5381	0.555658819
ensp00000351379	pfn3	5382	0.555762082
ensp00000322170	pfn4	5383	0.555865345
ensp00000314151	klk3	5384	0.555968608
ensp00000313581	klk2	5385	0.556071871
ensp00000236850	apoa1	5388	0.55638166
ensp00000239940	pfn2	5390	0.556588187
ensp00000293422	myl6	5392	0.556794713
ensp00000370473	igfbp3	5394	0.557001239
ensp00000236671	ctsd	5395	0.557104502
ensp00000350199	ap1b1	5396	0.557207765

ensp00000281537	tjp1	5399	0.557517555
ensp00000366453	tjp2	5400	0.557620818
ensp00000247461	canx	5401	0.557724081
ensp00000388526	hla-a	5402	0.557827344
ensp00000340858	b2m	5403	0.557930607
ensp00000400842	hla-b	5404	0.55803387
ensp00000356530	rnasel	5406	0.558240397
ensp00000306565	isg20	5407	0.55834366
ensp00000333657	mx2	5408	0.558446923
ensp00000331103	ip6k2	5409	0.558550186
ensp00000264380	pikfyve	5410	0.558653449
ensp00000230124	fig4	5411	0.558756712
ensp00000261776	vac14	5412	0.558859975
ensp00000295137	actg2	5413	0.558963238
ensp00000263621	elane	5414	0.559066501
ensp00000268150	mfge8	5417	0.559376291
ensp00000416330	tgfbi	5418	0.559479554
ensp00000306361	fga	5419	0.559582817
ensp00000306099	fgb	5420	0.55968608
ensp00000336829	fgg	5421	0.559789343
ensp00000284240	thy1	5422	0.559892606
ensp00000316779	bin1	5423	0.559995869
ensp00000278385	cd44	5424	0.560099133
ensp00000261023	itgav	5426	0.560305659
ensp00000296181	itgb5	5428	0.560512185
ensp00000222573	itgb8	5429	0.560615448
ensp00000356595	abl2	5430	0.560718711
ensp00000273739	slit2	5432	0.560925238
ensp00000347198	srgap1	5433	0.561028501
ensp00000347184	htt	5434	0.561131764
ensp00000252486	apoe	5436	0.56133829
ensp00000231751	ltf	5437	0.561441553
ensp00000367828	itm2b	5438	0.561544816
ensp00000366124	cst3	5441	0.561854606
ensp00000264691	f11	5442	0.561957869
ensp00000253496	f12	5443	0.562061132
ensp00000264690	klkb1	5444	0.562164395
ensp00000225698	c1qbp	5445	0.562267658
ensp00000359074	l1cam	5446	0.562370921
ensp00000413234	ap2a2	5447	0.562474184
ensp00000351926	ap2a1	5448	0.562577447
ensp00000329380	gp1ba	5449	0.56268071
ensp00000303942	gp9	5451	0.562887237
ensp00000299402	apbb1	5453	0.563093763
ensp00000401548	tcf19	5454	0.563197026
ensp00000372975	hla-c	5455	0.563300289
ensp00000397867	hla-c	5456	0.563403552

ensp00000377344	ddx60	5461	0.563919868
ensp00000353512	parp9	5462	0.564023131
ensp00000226218	vtn	5463	0.564126394
ensp00000228307	pxn	5464	0.564229657
ensp00000268296	itgax	5466	0.564436183
ensp00000322788	mmp1	5468	0.56464271
ensp00000361850	plau	5469	0.564745973
ensp00000339328	plaur	5470	0.564849236
ensp00000332274	ptp4a3	5471	0.564952499
ensp00000278407	serping1	5472	0.565055762
ensp00000264870	f13a1	5473	0.565159025
ensp00000353393	f8	5475	0.565365551
ensp00000295897	alb	5476	0.565468815
ensp00000346839	fn1	5477	0.565572078
ensp00000293379	itga5	5478	0.565675341
ensp00000282588	itga1	5480	0.565881867
ensp00000007722	itga3	5481	0.56598513
ensp00000294984	il24	5482	0.566088393
ensp00000250448	foxa1	5483	0.566191656
ensp00000304004	foxa3	5485	0.566398183
ensp00000314414	ap2b1	5486	0.566501446
ensp00000357668	adam12	5489	0.566811235
ensp00000273221	iqsec1	5491	0.567017761
ensp00000318982	epsti1	5492	0.567121024
ensp00000160262	icam3	5493	0.567224287
ensp00000342114	icam4	5495	0.567430814
ensp00000321334	lpa	5496	0.567534077
ensp00000366307	thbd	5497	0.56763734
ensp00000234071	proc	5498	0.567740603
ensp00000308541	f2	5499	0.567843866
ensp00000333203	serpina5	5502	0.568153656
ensp00000215743	mmp11	5503	0.568256919
ensp00000279441	mmp10	5504	0.568360182
ensp00000299855	mmp3	5505	0.568463445
ensp00000260227	mmp7	5506	0.568566708
ensp00000245932	vasp	5507	0.568669971
ensp00000355809	enah	5508	0.568773234
ensp00000307078	kif5b	5509	0.568876497
ensp00000355136	sorbs1	5510	0.56897976
ensp00000354826	cald1	5511	0.569083024
ensp00000240123	sorbs3	5512	0.569186287
ensp00000269485	tnfrsf11a	5514	0.569392813
ensp00000299502	serpinb2	5515	0.569496076
ensp00000333266	uba7	5516	0.569599339
ensp00000251642	dhx58	5517	0.569702602
ensp00000356671	serpinc1	5519	0.569909128
ensp00000355330	tgm2	5521	0.570115655

ensp00000263642	ifih1	5522	0.570218918
ensp00000369213	ddx58	5523	0.570322181
ensp00000323889	trim25	5524	0.570425444
ensp00000328340	rnf135	5525	0.570528707
ensp00000295956	flnb	5526	0.57063197
ensp00000365411	apbb1ip	5527	0.570735233
ensp00000262407	itga2b	5528	0.570838496
ensp00000257879	itga7	5529	0.57094176
ensp00000283249	itgb6	5531	0.571148286
ensp00000267082	itgb7	5532	0.571251549
ensp00000380227	itga4	5533	0.571354812
ensp00000364094	itgb1	5534	0.571458075
ensp00000264741	itga9	5535	0.571561338
ensp00000317790	sptbn5	5537	0.571767865
ensp00000361926	cnpy3	5538	0.571871128
ensp00000353874	tlr9	5540	0.572077654
ensp00000263645	cd81	5544	0.572490706
ensp00000416387	fblim1	5545	0.572593969
ensp00000364731	f7	5546	0.572697233
ensp00000289407	tspan33	5548	0.572903759
ensp00000348635	amica1	5549	0.573007022
ensp00000222693	cav2	5550	0.573110285
ensp00000341940	cav3	5551	0.573213548
ensp00000349205	pik3r4	5552	0.573316811
ensp00000244458	pacsin1	5553	0.573420074
ensp00000263246	pacsin2	5554	0.573523337
ensp00000378517	spp1	5556	0.573729864
ensp00000400365	lama2	5557	0.573833127
ensp00000230538	lama4	5558	0.57393639
ensp00000298743	gas1	5559	0.574039653
ensp00000298159	cfl2	5560	0.574142916
ensp00000309629	cfl1	5561	0.574246179
ensp00000200181	itgb4	5562	0.574349442
ensp00000386896	itga6	5563	0.574452705
ensp00000345494	plscr1	5564	0.574555969
ensp00000205386	lamb4	5565	0.574659232
ensp00000307156	lamb2	5566	0.574762495
ensp00000354360	lamc3	5567	0.574865758
ensp00000258341	lamc1	5568	0.574969021
ensp00000222399	lamb1	5569	0.575072284
ensp00000374309	lama1	5570	0.575175547
ensp00000324532	lama3	5571	0.57527881
ensp00000222725	lfng	5573	0.575485337
ensp00000268171	furin	5574	0.5755886
ensp00000233838	ggcx	5575	0.575691863
ensp00000376652	evl	5576	0.575795126
ensp00000316454	pacs1	5578	0.576001652

ensp00000296754	erap1	5580	0.576208178
ensp00000320866	calr	5581	0.576311442
ensp00000300289	pdia3	5582	0.576414705
ensp00000397705	hla-f	5583	0.576517968
ensp00000391723	pum1	5585	0.576724494
ensp00000366525	ftl	5587	0.57693102
ensp00000358310	itga10	5588	0.577034283
ensp00000327290	itga11	5589	0.577137546
ensp00000384515	parvb	5590	0.57724081
ensp00000334008	parva	5592	0.577447336
ensp00000336775	synm	5593	0.577550599
ensp00000312435	dag1	5594	0.577653862
ensp00000356991	pvrl4	5595	0.577757125
ensp00000418070	pvrl3	5596	0.577860388
ensp00000246551	hcst	5597	0.577963651
ensp00000262629	tyrobp	5598	0.578066914
ensp00000295408	mertk	5599	0.578170178
ensp00000316983	sv2c	5600	0.578273441
ensp00000332818	sv2b	5601	0.578376704
ensp00000334002	hap1	5602	0.578479967
ensp00000320081	c3orf58	5604	0.578686493
ensp00000347329	sec31a	5606	0.578893019
ensp00000362335	sar1a	5608	0.579099546
ensp00000260356	thbs1	5610	0.579306072
ensp00000339730	thbs4	5611	0.579409335
ensp00000252999	lama5	5612	0.579512598
ensp00000264144	lamc2	5613	0.579615861
ensp00000348384	lamb3	5614	0.579719124
ensp00000324101	cd151	5615	0.579822387
ensp00000359370	sec31b	5616	0.579925651
ensp00000216336	ctsg	5617	0.580028914
ensp00000354280	prss3	5619	0.58023544
ensp00000310832	ctsf	5620	0.580338703
ensp00000334052	lgmn	5623	0.580648492
ensp00000342070	ctsb	5624	0.580751755
ensp00000264025	pvrl1	5625	0.580855019
ensp00000252483	pvrl2	5626	0.580958282
ensp00000260643	preb	5627	0.581061545
ensp00000265175	sec24b	5628	0.581164808
ensp00000306881	sec23a	5629	0.581268071
ensp00000381823	sec24a	5630	0.581371334
ensp00000280551	sec24d	5631	0.581474597
ensp00000321845	sec24c	5632	0.58157786
ensp00000009530	cd74	5633	0.581681124
ensp00000336666	ap1s1	5634	0.581784387
ensp00000377148	ap1g1	5635	0.58188765
ensp00000250244	ap1m2	5636	0.581990913

ensp00000378735	dnajc6	5637	0.582094176
ensp00000220931	ncald	5638	0.582197439
ensp00000238855	aftph	5639	0.582300702
ensp00000329419	copb2	5641	0.582507228
ensp00000262061	copz1	5642	0.582610492
ensp00000328789	ap1s2	5644	0.582817018
ensp00000379891	ap1s3	5645	0.582920281
ensp00000302913	sh3d19	5646	0.583023544
ensp00000388340	clint1	5647	0.583126807
ensp00000341170	ptn	5648	0.58323007
ensp00000344468	sdc3	5649	0.583333333
ensp00000307046	sdc2	5650	0.583436596
ensp00000254351	sdc1	5651	0.58353986
ensp00000361818	sdc4	5652	0.583643123
ensp00000264039	gpc1	5653	0.583746386
ensp00000296145	tdgf1	5654	0.583849649
ensp00000281419	asap2	5657	0.584159438
ensp00000350297	asap1	5658	0.584262701
ensp00000352798	col18a1	5659	0.584365964
ensp00000304408	col3a1	5660	0.584469228
ensp00000297268	col1a2	5661	0.584572491
ensp00000225964	col1a1	5662	0.584675754
ensp00000355751	thbs2	5663	0.584779017
ensp00000357362	thbs3	5664	0.58488228
ensp00000222271	comp	5665	0.584985543
ensp00000303476	tln2	5666	0.585088806
ensp00000360882	col5a1	5669	0.585398596
ensp00000353654	col4a2	5671	0.585605122
ensp00000364000	col5a2	5672	0.585708385
ensp00000379823	col4a3	5674	0.585914911
ensp00000332371	col7a1	5675	0.586018174
ensp00000347847	mkl1	5676	0.586121437
ensp00000362650	scai	5677	0.586224701
ensp00000336747	hip1	5678	0.586327964
ensp00000309415	cltb	5679	0.586431227
ensp00000242285	clta	5680	0.58653449
ensp00000357048	copa	5681	0.586637753
ensp00000325527	fbn1	5682	0.586741016
ensp00000327145	flnc	5683	0.586844279
ensp00000239462	tnn	5684	0.586947542
ensp00000355180	col6a1	5686	0.587154069
ensp00000300527	col6a2	5687	0.587257332
ensp00000295550	col6a3	5688	0.587360595
ensp00000379866	col4a4	5689	0.587463858
ensp00000361290	col4a6	5690	0.587567121
ensp00000331902	col4a5	5691	0.587670384
ensp00000364979	col4a1	5692	0.587773647

ensp00000258969	chad	5694	0.587980173
ensp00000296591	edil3	5695	0.588083437
ensp00000340937	col17a1	5697	0.588289963
ensp00000359603	col24a1	5698	0.588393226
ensp00000349790	col9a1	5699	0.588496489
ensp00000316030	col19a1	5700	0.588599752
ensp00000303153	col22a1	5701	0.588703015
ensp00000297848	col14a1	5702	0.588806278
ensp00000305913	col8a2	5706	0.589219331
ensp00000261037	col8a1	5707	0.589322594
ensp00000348695	col13a1	5708	0.589425857
ensp00000362776	col16a1	5709	0.58952912
ensp00000348385	col27a1	5710	0.589632383
ensp00000364140	col15a1	5711	0.589735646
ensp00000375069	col23a1	5712	0.58983891
ensp00000333255	vma21	5714	0.590045436
ensp00000393912	copg2	5715	0.590148699
ensp00000006101	copz2	5716	0.590251962
ensp00000414817	hla-dmb	5718	0.590458488
ensp00000410443	hla-dra	5721	0.590768278
ensp00000339398	hla-dqa1	5722	0.590871541
ensp00000382034	hla-dqb1	5723	0.590974804
ensp00000372608	hla-dra	5724	0.591078067
ensp00000402060	pvr	5727	0.591387856
ensp00000265131	tnc	5728	0.591491119
ensp00000263525	tnr	5729	0.591594382
ensp00000361834	col9a2	5731	0.591800909
ensp00000382356	col28a1	5732	0.591904172
ensp00000351767	col20a1	5733	0.592007435
ensp00000325146	col12a1	5734	0.592110698
ensp00000341640	col9a3	5735	0.592213961
ensp00000243222	col10a1	5736	0.592317224
ensp00000360672	pard6b	5737	0.592420487
ensp00000343144	pard6g	5738	0.592523751
ensp00000376333	llgl2	5739	0.592627014
ensp00000316809	pitpna	5740	0.592730277
ensp00000261908	neo1	5741	0.59283354
ensp00000233813	igfbp5	5743	0.593040066
ensp00000330658	pappa	5744	0.593143329
ensp00000233809	igfbp2	5745	0.593246592
ensp00000257899	bloc1s1	5747	0.593453119
ensp00000341680	dtnbp1	5748	0.593556382
ensp00000369081	txndc5	5751	0.593866171
ensp00000270452	lilrb4	5755	0.594279223
ensp00000291759	lilra4	5759	0.594692276
ensp00000251372	lilra1	5761	0.594898802
ensp00000416448	hla-doa	5763	0.595105328

ensp00000410390	hla-dob	5764	0.595208591
ensp00000414196	hla-dpb1	5765	0.595311855
ensp00000332353	ptch1	5766	0.595415118
ensp00000297261	shh	5767	0.595518381
ensp00000362638	marcksl1	5768	0.595621644
ensp00000267996	tpm1	5769	0.595724907
ensp00000354219	tpm2	5770	0.59582817
ensp00000345230	tpm4	5771	0.595931433
ensp00000234313	plek	5775	0.596344486
ensp00000265517	mttp	5776	0.596447749
ensp00000327801	p4hb	5777	0.596551012
ensp00000311528	gpr162	5778	0.596654275
ensp00000316881	leprel1	5779	0.596757538
ensp00000296388	lepre1	5780	0.596860801
ensp00000414982	klc1	5781	0.596964064
ensp00000368020	kif3a	5782	0.597067328
ensp00000364864	kif3b	5783	0.597170591
ensp00000352671	oscar	5784	0.597273854
ensp00000265132	ambp	5786	0.59748038
ensp00000205948	apoh	5787	0.597583643
ensp00000261681	mpp5	5788	0.597686906
ensp00000212355	tgfbr3	5789	0.597790169
ensp00000369154	smad9	5791	0.597996696
ensp00000333769	bsg	5793	0.598203222
ensp00000355361	cd47	5794	0.598306485
ensp00000314153	zbtb33	5797	0.598616274
ensp00000363071	des	5800	0.598926064
ensp00000217381	snta1	5801	0.599029327
ensp00000311997	nefh	5803	0.599235853
ensp00000322804	mtss1	5807	0.599648905
ensp00000259365	tmod1	5808	0.599752169
ensp00000226209	myh3	5809	0.599855432
ensp00000228841	myl2	5810	0.599958695
ensp00000292327	myl3	5811	0.600061958
ensp00000346467	ltbp1	5812	0.600165221
ensp00000272134	lefty1	5813	0.600268484
ensp00000379204	bmp7	5815	0.60047501
ensp00000245451	bmp4	5816	0.600578273
ensp00000368104	bmp2	5817	0.600681537
ensp00000264568	bmpr1b	5818	0.6007848
ensp00000363708	bmpr2	5820	0.600991326
ensp00000337159	zfyve16	5821	0.601094589
ensp00000313391	dab2	5822	0.601197852
ensp00000263640	acvr1	5823	0.601301115
ensp00000243349	acvr1c	5824	0.601404378
ensp00000358142	sv2a	5825	0.601507641
ensp00000358131	otud7b	5826	0.601610905

ensp00000393887	ahsg	5827	0.601714168
ensp00000252490	apoc2	5829	0.601920694
ensp00000263816	lrp2	5830	0.602023957
ensp00000241416	acvr2a	5831	0.60212722
ensp00000340361	acvr2b	5832	0.602230483
ensp00000221496	amh	5834	0.60243701
ensp00000256366	synj2bp	5836	0.602643536
ensp00000265565	scap	5839	0.602953325
ensp00000344223	mbtps1	5840	0.603056588
ensp00000368798	mbtps2	5841	0.603159851
ensp00000233156	tfpi	5842	0.603263114
ensp00000334145	f3	5843	0.603366378
ensp00000337022	tnni1	5844	0.603469641
ensp00000291901	tnnt1	5845	0.603572904
ensp00000361636	tnnc2	5846	0.603676167
ensp00000257963	acvr1b	5848	0.603882693
ensp00000308219	rgmb	5850	0.604089219
ensp00000330005	rgma	5851	0.604192482
ensp00000295633	fstl1	5852	0.604295746
ensp00000295731	ihh	5853	0.604399009
ensp00000266991	dhh	5854	0.604502272
ensp00000315147	isyna1	5855	0.604605535
ensp00000368976	kifc3	5858	0.604915324
ensp00000358994	myo6	5859	0.605018587
ensp00000252444	ldlr	5860	0.60512185
ensp00000263036	optn	5861	0.605225114
ensp00000349298	mylip	5862	0.605328377
ensp00000260130	sdcbp	5863	0.60543164
ensp00000344741	insig1	5865	0.605638166
ensp00000245787	insig2	5866	0.605741429
ensp00000348307	sirpa	5868	0.605947955
ensp00000305529	sirpg	5870	0.606154482
ensp00000363489	gdf5	5871	0.606257745
ensp00000283147	bmp6	5872	0.606361008
ensp00000304930	sostdc1	5873	0.606464271
ensp00000204604	chrd	5874	0.606567534
ensp00000256759	fst	5875	0.606670797
ensp00000300177	grem1	5876	0.60677406
ensp00000264028	arcn1	5878	0.606980587
ensp00000249923	copb1	5879	0.60708385
ensp00000359000	gbf1	5880	0.607187113
ensp00000259371	dab2ip	5881	0.607290376
ensp00000262644	impad1	5882	0.607393639
ensp00000347507	myh7	5884	0.607600165
ensp00000233638	tlx2	5886	0.607806691
ensp00000318650	grem2	5888	0.608013218
ensp00000328181	nog	5890	0.608219744

ensp00000364683	bambi	5891	0.608323007
ensp00000363921	pard3	5892	0.60842627
ensp00000363827	hspg2	5893	0.608529533
ensp00000255040	apcs	5894	0.608632796
ensp00000220584	fdft1	5895	0.608736059
ensp00000345702	nfya	5896	0.608839323
ensp00000240055	nfyb	5897	0.608942586
ensp00000396620	nfyc	5898	0.609045849
ensp00000354476	srebf2	5899	0.609149112
ensp00000287936	hmgcr	5900	0.609252375
ensp00000301012	mvd	5901	0.609355638
ensp00000344871	myo1f	5903	0.609562164
ensp00000361266	ptch2	5905	0.609768691
ensp00000347546	boc	5906	0.609871954
ensp00000296575	hhip	5907	0.609975217
ensp00000232975	tnnc1	5908	0.61007848
ensp00000278317	tnnt3	5909	0.610181743
ensp00000252898	tnni2	5910	0.610285006
ensp00000382193	mybpc3	5912	0.610491532
ensp00000347055	myl4	5913	0.610594796
ensp00000350332	mybpc2	5914	0.610698059
ensp00000341838	tnni3	5915	0.610801322
ensp00000352834	myo1c	5919	0.611214374
ensp00000361027	amot	5920	0.611317637
ensp00000219150	coro1a	5921	0.6114209
ensp00000361915	bmp8b	5922	0.611524164
ensp00000272224	gdf7	5923	0.611627427
ensp00000327440	bmp8a	5924	0.61173069
ensp00000287020	gdf6	5925	0.611833953
ensp00000359866	bmp5	5926	0.611937216
ensp00000252456	cnn1	5927	0.612040479
ensp00000264758	add1	5928	0.612143742
ensp00000237596	pkd2	5929	0.612247005
ensp00000360860	ifit5	5930	0.612350268
ensp00000308716	inhbc	5932	0.612556795
ensp00000266646	inhbe	5933	0.612660058
ensp00000242208	inhba	5934	0.612763321
ensp00000295228	inhbb	5935	0.612866584
ensp00000243786	inha	5936	0.612969847
ensp00000312624	tcap	5938	0.613176373
ensp00000354923	dmd	5939	0.613279637
ensp00000262873	myh7b	5943	0.613692689
ensp00000258201	fhod1	5946	0.614002478
ensp00000384169	fbln2	5947	0.614105741
ensp00000278282	scgb1a1	5948	0.614209005
ensp00000269844	prdm15	5949	0.614312268
ensp00000043402	rtn4r	5954	0.614828583

ensp00000365402	hla-c	5955	0.614931846
ensp00000409132	hla-g	5956	0.615035109
ensp00000366005	hla-a	5957	0.615138373
ensp00000409910	hla-e	5958	0.615241636
ensp00000005587	skap2	5961	0.615551425
ensp00000284273	ubash3b	5963	0.615757951
ensp00000254436	trim21	5964	0.615861214
ensp00000304051	rnf139	5965	0.615964477
ensp00000294507	laptm5	5966	0.616067741
ensp00000270349	slc6a3	5967	0.616171004
ensp00000228916	scnn1a	5968	0.616274267
ensp00000265372	crem	5971	0.616584056
ensp00000345001	crtc1	5972	0.616687319
ensp00000394049	sla	5973	0.616790582
ensp00000360272	sorbs1	5974	0.616893846
ensp00000284776	sorbs2	5975	0.616997109
ensp00000303145	tmed10	5976	0.617100372
ensp00000295225	kcnip3	5977	0.617203635
ensp00000265381	apba1	5978	0.617306898
ensp00000339634	kir2dl4	5980	0.617513424
ensp00000375608	kir3dl1	5981	0.617616687
ensp00000352064	klrc1	5982	0.61771995
ensp00000338130	klrd1	5983	0.617823214
ensp00000329471	kdelr1	5984	0.617926477
ensp00000411949	trim39	5985	0.61802974
ensp00000402869	wdr46	5986	0.618133003
ensp00000372789	ppt2	5987	0.618236266
ensp00000416233	hla-a	5991	0.618649318
ensp00000410645	hla-a	5992	0.618752582
ensp00000306190	slamf1	5994	0.618959108
ensp00000357013	cd244	5995	0.619062371
ensp00000263212	ppm1f	5996	0.619165634
ensp00000234389	grin3b	5998	0.61937216
ensp00000306654	gfra2	5999	0.619475423
ensp00000262593	dok5	6000	0.619578686
ensp00000344277	dok4	6001	0.61968195
ensp00000302648	nrtn	6002	0.619785213
ensp00000245810	pspn	6003	0.619888476
ensp00000371328	klrc3	6008	0.620404791
ensp00000310216	klrc4	6010	0.620611318
ensp00000349436	adam15	6011	0.620714581
ensp00000265769	adam28	6012	0.620817844
ensp00000316664	igsf8	6013	0.620921107
ensp00000264563	il11	6014	0.62102437
ensp00000279804	ctf1	6015	0.621127633
ensp00000309338	clcf1	6016	0.621230896
ensp00000261858	glce	6018	0.621437423

ensp00000359854	gpc3	6019	0.621540686
ensp00000368678	agrn	6020	0.621643949
ensp00000366246	gpc6	6021	0.621747212
ensp00000254301	lgals3	6022	0.621850475
ensp00000258729	igf2bp3	6024	0.622057001
ensp00000350387	hyal2	6027	0.622366791
ensp00000340466	ganab	6028	0.622470054
ensp00000228506	mlec	6029	0.622573317
ensp00000266085	timp3	6030	0.62267658
ensp00000262768	timp2	6031	0.622779843
ensp00000286614	mmp16	6032	0.622883106
ensp00000005279	synrg	6033	0.622986369
ensp00000272102	arf1	6034	0.623089632
ensp00000202677	ralgapa2	6035	0.623192895
ensp00000262879	ralgapb	6036	0.623296159
ensp00000035307	chpf2	6043	0.624019
ensp00000243776	chpf	6044	0.624122264
ensp00000357452	pmvk	6045	0.624225527
ensp00000228510	mvk	6046	0.62432879
ensp00000370748	idi1	6047	0.624432053
ensp00000282841	ggps1	6048	0.624535316
ensp00000349078	fdps	6049	0.624638579
ensp00000378965	sntb1	6052	0.624948368
ensp00000338191	sntb2	6053	0.625051632
ensp00000352513	mpzl1	6054	0.625154895
ensp00000333919	btla	6055	0.625258158
ensp00000265944	myo3a	6056	0.625361421
ensp00000338967	arhgap6	6057	0.625464684
ensp00000300119	myo1a	6058	0.625567947
ensp00000380444	myo9b	6059	0.62567121
ensp00000010338	traf3ip3	6060	0.625774473
ensp00000358541	sike1	6061	0.625877736
ensp00000338481	epb41l2	6064	0.626187526
ensp00000290100	epb41	6065	0.626290789
ensp00000359581	hs2st1	6067	0.626497315
ensp00000261374	hs3st2	6068	0.626600578
ensp00000002596	hs3st1	6069	0.626703841
ensp00000284110	hs3st3a1	6070	0.626807105
ensp00000323780	ip6k1	6071	0.626910368
ensp00000293756	ip6k3	6072	0.627013631
ensp00000287996	ippk	6073	0.627116894
ensp00000290902	spon2	6076	0.627426683
ensp00000373614	selplg	6077	0.627529946
ensp00000263686	selp	6078	0.627633209
ensp00000009180	cd9	6079	0.627736473
ensp00000369323	npnt	6080	0.627839736
ensp00000370115	serpinb1	6082	0.628046262

ensp00000261405	vwf	6083	0.628149525
ensp00000269033	ssh2	6084	0.628252788
ensp00000312081	ssh3	6085	0.628356051
ensp00000331867	tas1r1	6086	0.628459314
ensp00000296088	snrk	6087	0.628562577
ensp00000352852	mdk	6089	0.628769104
ensp00000312506	cspg4	6090	0.628872367
ensp00000252575	ncan	6091	0.62897563
ensp00000353072	atp2a3	6094	0.629285419
ensp00000349595	atp2a1	6095	0.629388682
ensp00000029410	b4galt7	6097	0.629595209
ensp00000259241	hs6st1	6098	0.629698472
ensp00000261797	ndst1	6099	0.629801735
ensp00000299641	ndst2	6100	0.629904998
ensp00000292377	gpc2	6101	0.630008261
ensp00000359864	gpc4	6103	0.630214787
ensp00000367446	ext1	6104	0.63031805
ensp00000342656	ext2	6105	0.630421314
ensp00000368496	b3galt6	6106	0.630524577
ensp00000264723	cspg5	6107	0.63062784
ensp00000265077	vcan	6108	0.630731103
ensp00000327336	bgn	6109	0.630834366
ensp00000052754	dcn	6110	0.630937629
ensp00000247933	idua	6111	0.631040892
ensp00000367408	cask	6112	0.631144155
ensp00000332151	dse	6114	0.631350682
ensp00000356433	ust	6115	0.631453945
ensp00000333947	chst15	6116	0.631557208
ensp00000307297	chst14	6120	0.63197026
ensp00000350937	tes	6121	0.632073523
ensp00000334300	actl7a	6122	0.632176786
ensp00000311962	gga2	6125	0.632486576
ensp00000341344	gga1	6126	0.632589839
ensp00000362207	chst3	6127	0.632693102
ensp00000254190	chsy1	6128	0.632796365
ensp00000302629	chsy3	6129	0.632899628
ensp00000310891	csgalnact1	6130	0.633002891
ensp00000276055	chst7	6131	0.633106154
ensp00000317404	chst13	6132	0.633209418
ensp00000305725	chst11	6133	0.633312681
ensp00000258711	chst12	6134	0.633415944
ensp00000355731	cdc42bpa	6135	0.633519207
ensp00000260502	bcar3	6136	0.63362247
ensp00000373347	srgap3	6138	0.633828996
ensp00000308107	hpse	6141	0.634138786
ensp00000336923	arhgap10	6147	0.634758364
ensp00000370007	tmsb4x	6148	0.634861627

ensp00000363590	csgalnact2	6150	0.635068154
ensp00000359131	extl2	6151	0.635171417
ensp00000220562	extl3	6152	0.63527468
ensp00000363398	extl1	6153	0.635377943
ensp00000325958	ptpmt1	6154	0.635481206
ensp00000338769	asap3	6158	0.635894259
ensp00000386104	cpe	6159	0.635997522
ensp00000393379	kif5c	6160	0.636100785
ensp00000314837	klc2	6162	0.636307311
ensp00000264712	kif3c	6163	0.636410574
ensp00000354560	kifap3	6164	0.636513837
ensp00000355651	rab4a	6165	0.6366171
ensp00000353444	rab5b	6166	0.636720363
ensp00000345689	rab5c	6167	0.636823627
ensp00000368538	tnfrsf4	6168	0.63692689
ensp00000349824	reps2	6169	0.637030153
ensp00000275635	lat2	6170	0.637133416
ensp00000294129	nckipsd	6171	0.637236679
ensp00000377233	arap1	6172	0.637339942
ensp00000338171	skap1	6173	0.637443205
ensp00000360181	sh2d1a	6174	0.637546468
ensp00000357113	ifi16	6175	0.637649732
ensp00000230053	b3gat2	6176	0.637752995
ensp00000265471	b3gat3	6177	0.637856258
ensp00000307875	b3gat1	6178	0.637959521
ensp00000373114	hla-a	6179	0.638062784
ensp00000383506	hla-g	6182	0.638372573
ensp00000221466	fcgrt	6183	0.638475836
ensp00000399168	hla-b	6184	0.6385791
ensp00000221554	ccdc130	6187	0.638888889
ensp00000329468	adap2	6190	0.639198678
ensp00000262477	rabep1	6195	0.639714994
ensp00000253699	zfyve20	6196	0.639818257
ensp00000317955	eea1	6197	0.63992152
ensp00000408411	lamp2	6198	0.640024783
ensp00000401287	agpat1	6200	0.640231309
ensp00000372757	pbx2	6201	0.640334572
ensp00000240851	tfg	6203	0.640541099
ensp00000255006	rin2	6204	0.640644362
ensp00000284957	rabgef1	6205	0.640747625
ensp00000348769	arih2	6206	0.640850888
ensp00000312150	trib1	6208	0.641057414
ensp00000376899	ptgfrn	6209	0.641160677
ensp00000362244	map7d1	6211	0.641367204
ensp00000352272	myoz1	6213	0.64157373
ensp00000318089	lpp	6214	0.641676993
ensp00000409167	mdc1	6215	0.641780256

ensp00000408012	cchcr1	6216	0.641883519
ensp00000382723	agpat1	6217	0.641986782
ensp00000290363	c1orf51	6222	0.642503098
ensp00000304707	sln	6223	0.642606361
ensp00000410829	tubb	6224	0.642709624
ensp00000391742	zmynd15	6225	0.642812887
ensp00000367356	fam171a1	6226	0.64291615
ensp00000353660	pea15	6227	0.643019413
ensp00000268605	nol3	6228	0.643122677
ensp00000221166	nefm	6230	0.643329203
ensp00000300026	ppib	6233	0.643638992
ensp00000323696	crtap	6234	0.643742255
ensp00000350894	serpinh1	6235	0.643845518
ensp00000355378	plxnb3	6236	0.643948781
ensp00000353104	dhdds	6237	0.644052045
ensp00000358033	pdss2	6238	0.644155308
ensp00000365388	pdss1	6239	0.644258571
ensp00000409275	coq2	6240	0.644361834
ensp00000296785	ankra2	6241	0.644465097
ensp00000305071	rfxank	6242	0.64456836
ensp00000290524	rfx5	6243	0.644671623
ensp00000255476	rfxap	6244	0.644774886
ensp00000284000	cebpg	6245	0.64487815
ensp00000403409	c6orf136	6247	0.645084676
ensp00000342118	hhipl2	6250	0.645394465
ensp00000372793	lta	6251	0.645497728
ensp00000358043	ecm1	6252	0.645600991
ensp00000357692	s100a16	6253	0.645704254
ensp00000373090	gnl1	6255	0.645910781
ensp00000356096	fam72a	6257	0.646117307
ensp00000365943	pcsk5	6258	0.64622057
ensp00000342805	faim	6259	0.646323833
ensp00000230381	prph2	6260	0.646427096
ensp00000257860	prph	6261	0.646530359
ensp00000303212	sema3e	6263	0.646736886
ensp00000317128	plxnd1	6264	0.646840149
ensp00000304604	magi3	6265	0.646943412
ensp00000340279	ssx2ip	6266	0.647046675
ensp00000342112	lmo7	6267	0.647149938
ensp00000261509	palld	6268	0.647253201
ensp00000347117	sema4a	6269	0.647356464
ensp00000332204	sema4b	6270	0.647459727
ensp00000210633	sema4g	6271	0.64756299
ensp00000350547	sema4f	6272	0.647666254
ensp00000356037	c4bpa	6274	0.64787278
ensp00000357153	cd1d	6277	0.648182569
ensp00000413493	cpsf3l	6284	0.648905411

ensp00000335500	c7orf57	6285	0.649008674
ensp00000220325	ehd4	6286	0.649111937
ensp00000355026	arhgef15	6287	0.6492152
ensp00000290894	shf	6288	0.649318463
ensp00000406367	gpr124	6290	0.64952499
ensp00000265361	sema3c	6291	0.649628253
ensp00000368401	pax6	6292	0.649731516
ensp00000345512	sema6a	6293	0.649834779
ensp00000265371	nrp1	6294	0.649938042
ensp00000303208	pcsk9	6295	0.650041305
ensp00000262626	hpn	6296	0.650144568
ensp00000373023	cdsn	6297	0.650247831
ensp00000365504	gtf2h4	6299	0.650454358
ensp00000368030	atad3a	6300	0.650557621
ensp00000388861	flot1	6303	0.65086741
ensp00000366032	commd3	6304	0.650970673
ensp00000412283	ier3	6305	0.651073936
ensp00000398355	trim39	6306	0.6511772
ensp00000225567	gosr2	6307	0.651280463
ensp00000326022	fhl5	6310	0.651590252
ensp00000397026	grik2	6311	0.651693515
ensp00000363965	alpl	6312	0.651796778
ensp00000289893	macf1	6313	0.651900041
ensp00000363976	hla-dma	6315	0.652106568
ensp00000378723	hla-dmb	6316	0.652209831
ensp00000240662	kcnj8	6317	0.652313094
ensp00000261200	abcc9	6318	0.652416357
ensp00000391998	artn	6319	0.65251962
ensp00000361418	ipo13	6320	0.652622883
ensp00000383205	cchcr1	6321	0.652726146
ensp00000383532	or11a1	6322	0.652829409
ensp00000352673	elf3	6323	0.652932672
ensp00000318585	bace1	6324	0.653035936
ensp00000288098	il34	6325	0.653139199
ensp00000353582	nrp2	6326	0.653242462
ensp00000372224	hgfac	6327	0.653345725
ensp00000294724	agl	6332	0.65386204
ensp00000398412	micb	6333	0.653965304
ensp00000373024	c6orf15	6334	0.654068567
ensp00000392835	c2	6335	0.65417183
ensp00000306497	kcnj4	6336	0.654275093
ensp00000328150	kcnj12	6337	0.654378356
ensp00000345731	dlg1	6338	0.654481619
ensp00000363480	dlg3	6340	0.654688145
ensp00000365272	dlg2	6341	0.654791409
ensp00000221459	lin7b	6342	0.654894672
ensp00000261203	lin7a	6343	0.654997935

ensp00000349437	igf2r	6344	0.655101198
ensp00000301464	igfbp6	6345	0.655204461
ensp00000316377	dlgap1	6346	0.655307724
ensp00000363023	dlgap4	6347	0.655410987
ensp00000365290	tnf	6348	0.65551425
ensp00000416509	lta	6349	0.655617513
ensp00000342098	spint1	6350	0.655720777
ensp00000345193	shank2	6351	0.65582404
ensp00000262895	grik5	6352	0.655927303
ensp00000314709	zhx2	6354	0.656133829
ensp00000256404	pebp4	6355	0.656237092
ensp00000357927	bnipl	6357	0.656443618
ensp00000341957	tp53bp2	6358	0.656546881
ensp00000357697	s100a2	6359	0.656650145
ensp00000360195	kank4	6360	0.656753408
ensp00000359174	slc35a3	6361	0.656856671
ensp00000399388	tcf19	6362	0.656959934
ensp00000406878	psmb8	6363	0.657063197
ensp00000411038	ppp1r11	6364	0.65716646
ensp00000407674	hla-dpb1	6365	0.657269723
ensp00000372595	psmb9	6366	0.657372986
ensp00000360525	magoh	6367	0.657476249
ensp00000414980	tcf19	6369	0.657682776
ensp00000407431	hla-c	6370	0.657786039
ensp00000382281	daxx	6371	0.657889302
ensp00000401397	ddr1	6372	0.657992565
ensp00000366006	ubiad1	6373	0.658095828
ensp00000386439	sync	6374	0.658199091
ensp00000278742	st14	6379	0.658715407
ensp00000372746	hla-dra	6381	0.658921933
ensp00000379154	rasgef1a	6382	0.659025196
ensp00000372713	hla-doa	6383	0.659128459
ensp00000398852	slc44a4	6384	0.659231722
ensp00000405229	trim27	6385	0.659334986
ensp00000408335	notch4	6387	0.659541512
ensp00000361878	cap1	6388	0.659644775
ensp00000399530	skiv2l	6391	0.659954564
ensp00000394400	skiv2l	6392	0.660057827
ensp00000356357	lhx9	6393	0.66016109
ensp00000403393	brd2	6395	0.660367617
ensp00000356966	tomm40l	6396	0.66047088
ensp00000329137	fam132a	6397	0.660574143
ensp00000347948	tnfrsf14	6399	0.660780669
ensp00000366513	clstn1	6400	0.660883932
ensp00000366537	c1orf200	6402	0.661090458
ensp00000397759	bsdc1	6403	0.661193722
ensp00000373091	hla-e	6404	0.661296985

ensp00000398971	ifrd2	6405	0.661400248
ensp00000349496	adprh	6406	0.661503511
ensp00000397331	hla-g	6409	0.6618133
ensp00000378089	nmb	6410	0.661916563
ensp00000351125	echdc2	6411	0.662019827
ensp00000290122	cela3a	6412	0.66212309
ensp00000355941	tatdn3	6413	0.662226353
ensp00000338629	rere	6417	0.662639405
ensp00000263966	usp13	6418	0.662742668
ensp00000261942	faf2	6420	0.662949195
ensp00000263202	ufd1l	6421	0.663052458
ensp00000287727	zfyve9	6423	0.663258984
ensp00000360718	rab3b	6424	0.663362247
ensp00000360034	serbp1	6425	0.66346551
ensp00000409605	trim15	6426	0.663568773
ensp00000194214	hspb11	6427	0.663672036
ensp00000405614	wdr46	6431	0.664085089
ensp00000412217	clic1	6432	0.664188352
ensp00000385479	hist2h3a	6435	0.664498141
ensp00000372873	flot1	6436	0.664601404
ensp00000239849	tnfsf11	6437	0.664704667
ensp00000297350	tnfrsf11b	6439	0.664911194
ensp00000414330	rimkla	6440	0.665014457
ensp00000271651	ctsk	6443	0.665324246
ensp00000349708	zmym6	6445	0.665530772
ensp00000236137	slc19a2	6446	0.665634036
ensp00000181796	fam107b	6449	0.665943825
ensp00000358794	slc16a4	6451	0.666150351
ensp00000358799	rbm15	6452	0.666253614
ensp00000366466	ctnnbip1	6453	0.666356877
ensp00000356652	cacybp	6454	0.66646014
ensp00000373107	ppp1r11	6456	0.666666667
ensp00000229725	neu1	6458	0.666873193
ensp00000355060	arhgef10l	6459	0.666976456
ensp00000407961	c2	6461	0.667182982
ensp00000415941	c4b	6462	0.667286245
ensp00000361005	atpaf1	6464	0.667492772
ensp00000416062	ppt2	6467	0.667802561
ensp00000359978	hhla3	6468	0.667905824
ensp00000358451	gdap2	6470	0.66811235
ensp00000396486	hspa1l	6471	0.668215613
ensp00000360222	tm2d1	6472	0.668318876
ensp00000367065	hes2	6473	0.66842214
ensp00000392347	hla-g	6474	0.668525403
ensp00000328173	c1s	6476	0.668731929
ensp00000349270	hbm	6478	0.668938455
ensp00000388000	egfl8	6483	0.669454771

ensp00000388842	pou5f1	6484	0.669558034
ensp00000361311	tmem53	6485	0.669661297
ensp00000359042	prpf38b	6486	0.66976456
ensp00000235835	akr7a2	6490	0.670177613
ensp00000388191	zbtb22	6491	0.670280876
ensp00000407195	tapbp	6492	0.670384139
ensp00000292169	s100a1	6493	0.670487402
ensp00000369335	fbxo18	6494	0.670590665
ensp00000365007	dnajc16	6495	0.670693928
ensp00000364956	plekhm2	6496	0.670797191
ensp00000368623	c1orf159	6497	0.670900454
ensp00000338330	paqr6	6498	0.671003717
ensp00000360583	zyg11a	6499	0.671106981
ensp00000402951	hla-dra	6501	0.671313507
ensp00000400104	rxrb	6502	0.67141677
ensp00000367086	acot7	6503	0.671520033
ensp00000364660	c2	6504	0.671623296
ensp00000410321	ly6g5c	6509	0.672139612
ensp00000401338	tmem8a	6510	0.672242875
ensp00000290039	cachd1	6513	0.672552664
ensp00000373106	rnf39	6514	0.672655927
ensp00000358617	phtf1	6515	0.67275919
ensp00000411286	gabbr1	6516	0.672862454
ensp00000262510	nlrc5	6517	0.672965717
ensp00000292199	nlrx1	6518	0.67306898
ensp00000366593	tmem201	6521	0.673378769
ensp00000335203	atpif1	6522	0.673482032
ensp00000414360	hla-dqa1	6523	0.673585295
ensp00000337425	hyal3	6524	0.673688558
ensp00000357901	scnm1	6525	0.673791822
ensp00000358136	mtmr11	6526	0.673895085
ensp00000289749	nbl1	6527	0.673998348
ensp00000339399	cryz	6528	0.674101611
ensp00000403154	ddah2	6530	0.674308137
ensp00000409428	ly6g6c	6531	0.6744114
ensp00000271357	gpr161	6537	0.675030979
ensp00000394371	trim26	6538	0.675134242
ensp00000372752	gpsm3	6539	0.675237505
ensp00000362570	fndc5	6540	0.675340768
ensp00000295314	tmod4	6542	0.675547295
ensp00000366272	mllt10	6545	0.675857084
ensp00000367059	espn	6547	0.67606361
ensp00000356156	ppp1r15b	6550	0.676373399
ensp00000358060	tars2	6553	0.676683189
ensp00000293872	luc7l	6558	0.677199504
ensp00000346729	znf589	6559	0.677302767
ensp00000356411	trove2	6562	0.677612557

ensp00000344364	lrrc29	6563	0.67771582
ensp00000403557	ppp1r11	6564	0.677819083
ensp00000359490	gbp4	6566	0.678025609
ensp00000360642	cc2d1b	6567	0.678128872
ensp00000340396	gbp5	6568	0.678232135
ensp00000372991	lta	6569	0.678335399
ensp00000391901	phf1	6570	0.678438662
ensp00000394802	vars2	6572	0.678645188
ensp00000399604	cdsn	6573	0.678748451
ensp00000403156	gtf2h4	6574	0.678851714
ensp00000363603	fuca1	6576	0.67905824
ensp00000356515	utrn	6577	0.679161504
ensp00000356468	ivns1abp	6581	0.679574556
ensp00000414373	abcf1	6587	0.680194135
ensp00000350734	st7l	6588	0.680297398
ensp00000361245	hectd3	6589	0.680400661
ensp00000411162	gnl1	6590	0.680503924
ensp00000412553	abcf1	6591	0.680607187
ensp00000387753	hsd17b8	6594	0.680916976
ensp00000360569	scp2	6595	0.68102024
ensp00000393114	aif1	6597	0.681226766
ensp00000258229	pcnxl2	6598	0.681330029
ensp00000360676	kti12	6599	0.681433292
ensp00000329002	hax1	6601	0.681639818
ensp00000406832	ly6g6d	6603	0.681846344
ensp00000271620	prune	6604	0.681949608
ensp00000309262	usp48	6605	0.682052871
ensp00000259891	mog	6606	0.682156134
ensp00000350310	atp2b4	6607	0.682259397
ensp00000257177	zcchc11	6608	0.68236266
ensp00000371294	ctns	6610	0.682569186
ensp00000264187	nid1	6611	0.682672449
ensp00000324304	tspan4	6612	0.682775713
ensp00000370377	pitrm1	6614	0.682982239
ensp00000294664	wdr63	6616	0.683188765
ensp00000294353	zyg11b	6617	0.683292028
ensp00000367013	tnfrsf25	6618	0.683395291
ensp00000261464	traf5	6619	0.683498554
ensp00000228918	ltbr	6620	0.683601817
ensp00000379709	arhgap21	6622	0.683808344
ensp00000356363	c1orf53	6624	0.68401487
ensp00000312625	hp1bp3	6629	0.684531185
ensp00000325589	ptprcap	6630	0.684634449
ensp00000356579	cep350	6632	0.684840975
ensp00000269260	arrb2	6633	0.684944238
ensp00000317578	adrbk2	6634	0.685047501
ensp00000334051	gnal	6635	0.685150764

ensp00000319308	rgs5	6637	0.68535729
ensp00000356560	kiaa1614	6639	0.685563817
ensp00000309992	zmynd11	6640	0.68566708
ensp00000290246	elmo2	6642	0.685873606
ensp00000331775	lims1	6643	0.685976869
ensp00000326888	lims2	6644	0.686080132
ensp00000171887	tns1	6645	0.686183395
ensp00000352268	aadacl3	6646	0.686286658
ensp00000348982	megf6	6647	0.686389922
ensp00000342818	znf669	6649	0.686596448
ensp00000358146	bola1	6650	0.686699711
ensp00000354677	gpx7	6651	0.686802974
ensp00000416511	mdc1	6652	0.686906237
ensp00000411113	c6orf136	6653	0.6870095
ensp00000394619	msh5	6655	0.687216026
ensp00000357861	selenbp1	6657	0.687422553
ensp00000416561	cfb	6660	0.687732342
ensp00000272198	ppfia4	6662	0.687938868
ensp00000363638	lypla2	6663	0.688042131
ensp00000361381	slc6a9	6664	0.688145394
ensp00000406250	hla-dpa1	6665	0.688248658
ensp00000383263	cchcr1	6667	0.688455184
ensp00000408453	hla-dmb	6668	0.688558447
ensp00000350009	usp33	6669	0.68866171
ensp00000355343	ubap2l	6671	0.688868236
ensp00000407779	nrm	6674	0.689178026
ensp00000401504	hla-doa	6675	0.689281289
ensp00000359707	samd13	6676	0.689384552
ensp00000362936	trnau1ap	6677	0.689487815
ensp00000355927	rps6kc1	6678	0.689591078
ensp00000408792	sp8	6679	0.689694341
ensp00000311827	msl2	6682	0.690004131
ensp00000312244	msl3	6684	0.690210657
ensp00000382064	dtna	6685	0.69031392
ensp00000379678	pgm5	6686	0.690417183
ensp00000255108	dph2	6688	0.690623709
ensp00000347038	plscr4	6689	0.690726972
ensp00000312673	gh1	6693	0.691140025
ensp00000309524	cshl1	6695	0.691346551
ensp00000360329	ttc4	6696	0.691449814
ensp00000303148	tmem37	6699	0.691759603
ensp00000245912	tnfsf14	6700	0.691862867
ensp00000278187	gas2	6702	0.692069393
ensp00000353472	hla-g	6703	0.692172656
ensp00000388724	hla-a	6704	0.692275919
ensp00000414808	ppp1r11	6705	0.692379182
ensp00000414110	znrd1	6706	0.692482445

ensp00000264638	cntnap1	6710	0.692895498
ensp00000382697	rock1	6711	0.692998761
ensp00000356399	cfh	6712	0.693102024
ensp00000378130	cfi	6713	0.693205287
ensp00000313875	cd46	6715	0.693411813
ensp00000413228	c3orf55	6716	0.693515076
ensp00000398462	ppt2	6718	0.693721603
ensp00000286031	c1orf112	6722	0.694134655
ensp00000294360	c1orf123	6723	0.694237918
ensp00000356395	cfhr3	6725	0.694444444
ensp00000360200	inadl	6726	0.694547708
ensp00000349204	crb3	6727	0.694650971
ensp00000394155	psmb8	6729	0.694857497
ensp00000321594	scnn1d	6730	0.69496076
ensp00000406144	ttc39a	6731	0.695064023
ensp00000396656	nfkbil1	6732	0.695167286
ensp00000397177	pbx2	6735	0.695477076
ensp00000389894	nrm	6736	0.695580339
ensp00000272203	plekha6	6738	0.695786865
ensp00000361554	tie1	6740	0.695993391
ensp00000246914	wnk4	6741	0.696096654
ensp00000289431	spata2	6742	0.696199917
ensp00000265631	slc25a13	6743	0.696303181
ensp00000359013	tnfrsf6b	6744	0.696406444
ensp00000282020	grid2	6745	0.696509707
ensp00000334382	homer1	6746	0.69661297
ensp00000282499	gria4	6747	0.696716233
ensp00000268864	rasl10b	6748	0.696819496
ensp00000320924	wipf2	6749	0.696922759
ensp00000367595	tprg1l	6750	0.697026022
ensp00000391685	trim31	6751	0.697129285
ensp00000362566	hpca	6752	0.697232549
ensp00000364685	mfap2	6753	0.697335812
ensp00000352455	mfap5	6754	0.697439075
ensp00000340292	dlk1	6755	0.697542338
ensp00000263895	rnd3	6756	0.697645601
ensp00000309595	c10orf2	6757	0.697748864
ensp00000355870	hlx	6761	0.698161917
ensp00000364550	kdm5c	6762	0.69826518
ensp00000301178	axl	6763	0.698368443
ensp00000263798	tyro3	6764	0.698471706
ensp00000363157	tnfsf15	6765	0.698574969
ensp00000254654	ilkap	6766	0.698678232
ensp00000355260	ppp1r14c	6767	0.698781495
ensp00000403221	trim15	6770	0.699091285
ensp00000216968	procr	6771	0.699194548
ensp00000367104	bfsp1	6772	0.699297811

ensp00000292246	ano10	6773	0.699401074
ensp00000300658	pgap3	6774	0.699504337
ensp00000217740	rnf125	6775	0.6996076
ensp00000333938	nexn	6779	0.700020653
ensp00000262776	lgals3bp	6781	0.700227179
ensp00000373572	foxi2	6782	0.700330442
ensp00000366702	errfi1	6783	0.700433705
ensp00000204615	thpo	6786	0.700743494
ensp00000279477	sirpb1	6787	0.700846758
ensp00000329942	cd300e	6788	0.700950021
ensp00000289575	slco2b1	6789	0.701053284
ensp00000357020	ly9	6793	0.701466336
ensp00000358674	ubl4a	6794	0.701569599
ensp00000415316	vars	6796	0.701776126
ensp00000348394	ncdn	6797	0.701879389
ensp00000285039	myo5b	6798	0.701982652
ensp00000347839	rab11fip2	6799	0.702085915
ensp00000263925	lnx1	6800	0.702189178
ensp00000219782	maz	6802	0.702395704
ensp00000347301	begain	6804	0.70260223
ensp00000304250	cdk5r2	6805	0.702705494
ensp00000357206	nes	6806	0.702808757
ensp00000358120	plekho1	6807	0.70291202
ensp00000369725	or52a1	6819	0.704151177
ensp00000409493	obscn	6820	0.70425444
ensp00000380349	capn3	6821	0.704357703
ensp00000406359	hspa1a	6822	0.704460967
ensp00000355568	irf2bp2	6824	0.704667493
ensp00000246533	capns1	6825	0.704770756
ensp00000279247	capn1	6826	0.704874019
ensp00000263317	nox4	6827	0.704977282
ensp00000386175	or2a1	6830	0.705287071
ensp00000325065	or4a15	6831	0.705390335
ensp00000343656	rapgef5	6836	0.70590665
ensp00000296859	rapgef6	6837	0.706009913
ensp00000253754	pdlim4	6838	0.706113176
ensp00000353154	nfasc	6839	0.706216439
ensp00000349588	ank2	6840	0.706319703
ensp00000265709	ank1	6841	0.706422966
ensp00000305988	alcam	6842	0.706526229
ensp00000367650	or5l2	6845	0.706836018
ensp00000299454	or10a5	6850	0.707352334
ensp00000412591	or5v1	6858	0.708178439
ensp00000324557	or2m7	6861	0.708488228
ensp00000320886	mlxipl	6862	0.708591491
ensp00000367079	acacb	6863	0.708694754
ensp00000344789	acaca	6864	0.708798017

ensp00000351190	itih2	6867	0.709107807
ensp00000346550	anxa6	6868	0.70921107
ensp00000323280	cd6	6869	0.709314333
ensp00000264896	scarb2	6870	0.709417596
ensp00000272163	lbr	6871	0.709520859
ensp00000265896	sqle	6873	0.709727385
ensp00000003100	cyp51a1	6874	0.709830648
ensp00000279263	tm7sf2	6875	0.709933912
ensp00000348762	lss	6877	0.710140438
ensp00000370710	kdm4c	6878	0.710243701
ensp00000259030	rtp4	6879	0.710346964
ensp00000360302	gria3	6880	0.710450227
ensp00000381293	nsf	6882	0.710656753
ensp00000297156	camlg	6886	0.711069806
ensp00000281834	tnfsf4	6887	0.711173069
ensp00000363458	ldlrap1	6890	0.711482858
ensp00000286091	pdia4	6892	0.711689385
ensp00000360519	rbp4	6893	0.711792648
ensp00000420716	c1orf228	6898	0.712308963
ensp00000393646	hla-dmb	6901	0.712618753
ensp00000372695	wdr46	6905	0.713031805
ensp00000328698	c10orf107	6906	0.713135068
ensp00000358126	vps45	6907	0.713238331
ensp00000327116	ehd3	6908	0.713341594
ensp00000263277	ehd2	6909	0.713444857
ensp00000406219	ppt2	6910	0.713548121
ensp00000389244	slc44a4	6912	0.713754647
ensp00000360149	alg6	6920	0.714580752
ensp00000291386	ssu72	6921	0.714684015
ensp00000328494	kif21b	6922	0.714787278
ensp00000341682	slc26a9	6924	0.714993804
ensp00000379042	ero1l	6927	0.715303594
ensp00000346635	ero1lb	6928	0.715406857
ensp00000270233	bcam	6929	0.71551012
ensp00000309710	slc39a1	6930	0.715613383
ensp00000356278	phlda3	6932	0.715819909
ensp00000341479	kcnj14	6933	0.715923172
ensp00000243457	kcnj2	6934	0.716026435
ensp00000370410	mpdz	6935	0.716129698
ensp00000295522	cldn1	6936	0.716232962
ensp00000318113	cldn22	6937	0.716336225
ensp00000353475	cldn7	6939	0.716542751
ensp00000064724	cldn11	6940	0.716646014
ensp00000328674	cldn6	6942	0.71685254
ensp00000347548	rnf220	6943	0.716955803
ensp00000391438	flot1	6944	0.717059067
ensp00000418018	ifne	6945	0.71716233

ensp00000262968	tjp3	6949	0.717575382
ensp00000336571	cldn2	6950	0.717678645
ensp00000264734	cldn16	6951	0.717781908
ensp00000296387	cldn19	6952	0.717885171
ensp00000378577	cldn3	6953	0.717988435
ensp00000355470	cnst	6954	0.718091698
ensp00000355480	efcab2	6955	0.718194961
ensp00000367439	rpp38	6956	0.718298224
ensp00000299339	cldn10	6957	0.718401487
ensp00000342445	cldn4	6958	0.71850475
ensp00000339292	cldn14	6963	0.719021066
ensp00000308870	cldn15	6964	0.719124329
ensp00000347379	ocln	6965	0.719227592
ensp00000364403	ubr4	6966	0.719330855
ensp00000253408	gfap	6969	0.719640644
ensp00000296280	masp1	6971	0.719847171
ensp00000315136	apba3	6972	0.719950434
ensp00000354947	capza2	6973	0.720053697
ensp00000263168	capza1	6974	0.72015696
ensp00000287916	cldn12	6975	0.720260223
ensp00000278927	esam	6976	0.720363486
ensp00000236147	sell	6977	0.720466749
ensp00000311688	or1s1	6978	0.720570012
ensp00000414688	or2h2	6983	0.721086328
ensp00000362788	or1l6	6984	0.721189591
ensp00000366460	plxdc2	6987	0.72149938
ensp00000351832	rab3gap2	6989	0.721705907
ensp00000305613	cplx1	6990	0.72180917
ensp00000264839	rims1	6991	0.721912433
ensp00000217420	slc32a1	6992	0.722015696
ensp00000355920	slc22a2	6993	0.722118959
ensp00000355511	chml	6994	0.722222222
ensp00000320516	ehd1	6995	0.722325485
ensp00000264276	als2	6996	0.722428748
ensp00000331983	lrrc16a	6997	0.722532012
ensp00000358715	sorcs3	7000	0.722841801
ensp00000330694	ndnl2	7001	0.722945064
ensp00000258052	smpd2	7002	0.723048327
ensp00000411012	nsmaf	7003	0.72315159
ensp00000344106	rtn3	7019	0.7248038
ensp00000337541	fam21c	7020	0.724907063
ensp00000328062	kiaa1033	7021	0.725010326
ensp00000240079	ccdc53	7022	0.725113589
ensp00000318016	kiaa0196	7023	0.725216853
ensp00000309052	catsper1	7024	0.725320116
ensp00000300737	stim1	7026	0.725526642
ensp00000328216	orai1	7027	0.725629905

ensp00000155858	trpm5	7028	0.725733168
ensp00000288221	erc2	7029	0.725836431
ensp00000298472	slc18a2	7030	0.725939694
ensp00000222812	stx1a	7031	0.726042957
ensp00000396876	daxx	7032	0.726146221
ensp00000335596	or10h1	7033	0.726249484
ensp00000291231	or3a3	7038	0.726765799
ensp00000305469	or1s2	7042	0.727178852
ensp00000256592	tshb	7046	0.727591904
ensp00000221421	lhb	7048	0.72779843
ensp00000272227	pdia6	7049	0.727901694
ensp00000349954	cgb	7051	0.72810822
ensp00000360997	adamts13	7053	0.728314746
ensp00000407739	or2h1	7054	0.728418009
ensp00000318956	or52k2	7058	0.728831062
ensp00000412097	or11a1	7060	0.729037588
ensp00000420502	or2a7	7061	0.729140851
ensp00000419119	or10c1	7062	0.729244114
ensp00000267540	ston2	7066	0.729657166
ensp00000263033	sytl4	7067	0.72976043
ensp00000415660	tap1	7068	0.729863693
ensp00000372599	tap2	7069	0.729966956
ensp00000372726	tap2	7071	0.730173482
ensp00000401149	tap1	7072	0.730276745
ensp00000412027	psmb9	7073	0.730380008
ensp00000362399	stxbp1	7074	0.730483271
ensp00000367756	unc13b	7075	0.730586534
ensp00000254976	snap25	7076	0.730689798
ensp00000338562	stx3	7077	0.730793061
ensp00000342554	stx2	7078	0.730896324
ensp00000261205	syt1	7079	0.730999587
ensp00000222256	rab3a	7080	0.73110285
ensp00000363229	slc18a3	7081	0.731206113
ensp00000221485	slc17a7	7082	0.731309376
ensp00000314508	gba	7083	0.731412639
ensp00000363397	ugcg	7084	0.731515903
ensp00000354829	sgms1	7085	0.731619166
ensp00000340409	smpd1	7086	0.731722429
ensp00000219334	smpd3	7087	0.731825692
ensp00000374552	rnf216	7088	0.731928955
ensp00000411321	hla-dmb	7089	0.732032218
ensp00000390632	mog	7090	0.732135481
ensp00000372700	slc39a7	7091	0.732238744
ensp00000372703	rxrb	7092	0.732342007
ensp00000291232	tnfrsf13c	7096	0.73275506
ensp00000365048	tnfsf13b	7098	0.732961586
ensp00000413254	rph3a	7099	0.733064849

ensp00000216264	cerk	7100	0.733168112
ensp00000311648	ugt8	7102	0.733374639
ensp00000261304	galc	7103	0.733477902
ensp00000351981	sgms2	7104	0.733581165
ensp00000264436	add2	7105	0.733684428
ensp00000360366	acot11	7109	0.73409748
ensp00000343557	zcchc17	7110	0.734200743
ensp00000349415	rab18	7111	0.734304007
ensp00000345868	gjb4	7113	0.734510533
ensp00000225474	csf3	7114	0.734613796
ensp00000364219	camk2n1	7115	0.734717059
ensp00000357812	tdrkh	7116	0.734820322
ensp00000358360	pde4dip	7117	0.734923585
ensp00000364034	tap2	7118	0.735026848
ensp00000378546	tapbp	7119	0.735130112
ensp00000402316	tap1	7120	0.735233375
ensp00000346206	tap1	7121	0.735336638
ensp00000372684	tapbp	7122	0.735439901
ensp00000334941	ptpru	7123	0.735543164
ensp00000360305	pdlim1	7125	0.73574969
ensp00000286448	vamp7	7126	0.735852953
ensp00000236192	vamp4	7127	0.735956216
ensp00000054666	vamp3	7128	0.73605948
ensp00000263864	vamp8	7130	0.736266006
ensp00000215095	stx1b	7131	0.736369269
ensp00000259988	fgfbp1	7132	0.736472532
ensp00000255416	mybph	7133	0.736575795
ensp00000311165	aqp1	7134	0.736679058
ensp00000372654	aqp4	7135	0.736782321
ensp00000347134	net1	7136	0.736885584
ensp00000221980	icam5	7137	0.736988848
ensp00000253571	rlim	7138	0.737092111
ensp00000257818	lmo2	7139	0.737195374
ensp00000392466	ldb1	7140	0.737298637
ensp00000322977	ssbp2	7141	0.7374019
ensp00000360371	ssbp3	7142	0.737505163
ensp00000345752	mtmr2	7145	0.737814952
ensp00000359417	mtmr1	7147	0.738021479
ensp00000415769	itih3	7148	0.738124742
ensp00000405812	slc4a8	7149	0.738228005
ensp00000406229	fat1	7150	0.738331268
ensp00000263773	fnbp4	7151	0.738434531
ensp00000272252	galm	7153	0.738641057
ensp00000360125	pgm1	7155	0.738847584
ensp00000371393	pgm2	7156	0.738950847
ensp00000298198	pgm2l1	7157	0.73905411
ensp00000301452	acer1	7158	0.739157373

ensp00000342609	acer2	7159	0.739260636
ensp00000378897	asah2	7160	0.739363899
ensp00000371152	asah1	7161	0.739467162
ensp00000247225	sgpp1	7162	0.739570425
ensp00000315137	sgpp2	7163	0.739673689
ensp00000307126	degs2	7164	0.739776952
ensp00000316476	degs1	7165	0.739880215
ensp00000263800	ltk	7168	0.740190004
ensp00000324628	napg	7169	0.740293267
ensp00000263354	napa	7170	0.74039653
ensp00000225724	gosr1	7171	0.740499793
ensp00000360183	stx16	7172	0.740603057
ensp00000352544	cplx2	7173	0.74070632
ensp00000357789	flg	7174	0.740809583
ensp00000215730	snap29	7177	0.741119372
ensp00000242770	stx10	7178	0.741222635
ensp00000258301	stx6	7179	0.741325898
ensp00000305255	stx8	7180	0.741429162
ensp00000356918	stx7	7181	0.741532425
ensp00000376792	vti1a	7182	0.741635688
ensp00000223369	ykt6	7184	0.741842214
ensp00000319096	rap2b	7185	0.741945477
ensp00000305692	gaa	7186	0.74204874
ensp00000326227	ganc	7187	0.742152003
ensp00000340211	coro1b	7190	0.742461793
ensp00000368646	prdx4	7191	0.742565056
ensp00000341848	golgb1	7192	0.742668319
ensp00000294179	stx5	7194	0.742874845
ensp00000372210	bet1l	7195	0.742978108
ensp00000222547	bet1	7197	0.743184634
ensp00000364986	agmat	7198	0.743287898
ensp00000265022	dgkg	7199	0.743391161
ensp00000288490	dgki	7200	0.743494424
ensp00000264057	dgkd	7201	0.743597687
ensp00000337572	dgkh	7202	0.74370095
ensp00000382260	dgkb	7203	0.743804213
ensp00000320340	dgkz	7204	0.743907476
ensp00000248444	vil1	7205	0.744010739
ensp00000412897	ifna4	7208	0.744320529
ensp00000362413	pgk1	7212	0.744733581
ensp00000234590	eno1	7213	0.744836844
ensp00000336927	aldoa	7214	0.744940107
ensp00000363988	aldob	7215	0.745043371
ensp00000226253	aldoc	7216	0.745146634
ensp00000321259	taldo1	7217	0.745249897
ensp00000358931	tktl1	7218	0.74535316
ensp00000416583	dera	7220	0.745559686

ensp00000223366	gck	7221	0.745662949
ensp00000229319	ldhb	7222	0.745766212
ensp00000405455	tkt	7223	0.745869475
ensp00000348877	gpi	7224	0.745972739
ensp00000379933	tpi1	7225	0.746076002
ensp00000377192	g6pd	7226	0.746179265
ensp00000304592	fasn	7227	0.746282528
ensp00000265276	gpam	7228	0.746385791
ensp00000337889	mxd4	7229	0.746489054
ensp00000229239	gapdh	7230	0.746592317
ensp00000352842	pfkm	7231	0.74669558
ensp00000174618	mnt	7232	0.746798843
ensp00000312250	adpgk	7233	0.746902107
ensp00000246912	mlx	7234	0.74700537
ensp00000261458	hhat	7235	0.747108633
ensp00000324527	myo1d	7237	0.747315159
ensp00000265028	dnajb11	7238	0.747418422
ensp00000265195	sil1	7240	0.747624948
ensp00000264065	dnajc10	7241	0.747728211
ensp00000220616	tg	7242	0.747831475
ensp00000287814	timp4	7243	0.747934738
ensp00000347883	plekha7	7245	0.748141264
ensp00000217086	sall4	7246	0.748244527
ensp00000291842	shkbp1	7248	0.748451053
ensp00000320936	il17ra	7250	0.74865758
ensp00000369071	postn	7251	0.748760843
ensp00000414905	hla-f	7252	0.748864106
ensp00000295987	syn1	7253	0.748967369
ensp00000007735	krt33a	7255	0.749173895
ensp00000342032	bpgm	7261	0.749793474
ensp00000297283	pgam2	7262	0.749896737
ensp00000412189	pgam4	7264	0.750103263
ensp00000370517	pfkp	7265	0.750206526
ensp00000179259	c12orf5	7266	0.750309789
ensp00000352047	gulp1	7267	0.750413052
ensp00000254584	arfip2	7269	0.750619579
ensp00000284637	sh3rf1	7270	0.750722842
ensp00000397055	mcf2	7271	0.750826105
ensp00000289968	arhgap17	7272	0.750929368
ensp00000366620	h6pd	7273	0.751032631
ensp00000270776	pgd	7274	0.751135894
ensp00000283646	rpia	7275	0.751239157
ensp00000381504	prps2	7277	0.751445684
ensp00000219240	dhodh	7278	0.751548947
ensp00000364475	fbp1	7280	0.751755473
ensp00000369100	pfkfb3	7281	0.751858736
ensp00000269848	pfkl	7282	0.751961999

ensp00000216117	hmox1	7283	0.752065262
ensp00000222305	usf2	7284	0.752168525
ensp00000216410	gnpnat1	7286	0.752375052
ensp00000307481	amdhd2	7287	0.752478315
ensp00000356047	pfkfb2	7288	0.752581578
ensp00000232375	pfkfb4	7289	0.752684841
ensp00000407981	ppp1r11	7290	0.752788104
ensp00000166139	fstl3	7292	0.75299463
ensp00000278544	acer3	7294	0.753201157
ensp00000264775	ppap2a	7296	0.753407683
ensp00000329697	ppap2c	7297	0.753510946
ensp00000385083	kdsr	7298	0.753614209
ensp00000315465	dscaml1	7299	0.753717472
ensp00000295448	gnpda2	7301	0.753923998
ensp00000311876	gnpda1	7302	0.754027261
ensp00000373411	nkiras1	7303	0.754130525
ensp00000007969	lrrc23	7304	0.754233788
ensp00000374359	zxdc	7305	0.754337051
ensp00000326706	plekho2	7306	0.754440314
ensp00000291568	cstb	7307	0.754543577
ensp00000306817	rbks	7308	0.75464684
ensp00000303575	nudt9	7309	0.754750103
ensp00000419628	nudt5	7310	0.754853366
ensp00000321735	slc16a8	7312	0.755059893
ensp00000358640	slc16a1	7313	0.755163156
ensp00000343479	nbr1	7314	0.755266419
ensp00000363970	map1lc3a	7315	0.755369682
ensp00000318318	mpi	7316	0.755472945
ensp00000290573	hk2	7317	0.755576208
ensp00000346643	hkdc1	7319	0.755782734
ensp00000267814	sord	7321	0.755989261
ensp00000260598	khk	7322	0.756092524
ensp00000288078	fuk	7323	0.756195787
ensp00000306670	nudt16l1	7328	0.756712102
ensp00000259254	gypc	7330	0.756918629
ensp00000297439	defb1	7332	0.757125155
ensp00000329890	defa5	7333	0.757228418
ensp00000364217	ager	7334	0.757331681
ensp00000289429	cd1a	7337	0.75764147
ensp00000385636	obsl1	7338	0.757744734
ensp00000368517	echdc3	7339	0.757847997
ensp00000378368	flot2	7340	0.75795126
ensp00000410658	flot1	7341	0.758054523
ensp00000264346	herc6	7342	0.758157786
ensp00000215794	usp18	7343	0.758261049
ensp00000366326	nebl	7344	0.758364312
ensp00000363298	bspry	7345	0.758467575

ensp00000410481	ltb	7348	0.758777365
ensp00000304006	pafah1b2	7349	0.758880628
ensp00000262890	pafah1b3	7350	0.758983891
ensp00000359928	tnni3k	7353	0.75929368
ensp00000353165	tpk1	7355	0.759500207
ensp00000288014	thtpa	7356	0.75960347
ensp00000364145	pfkfb1	7357	0.759706733
ensp00000390849	abhd5	7358	0.759809996
ensp00000285518	agpat5	7359	0.759913259
ensp00000265965	sergef	7361	0.760119785
ensp00000372901	ddah2	7363	0.760326311
ensp00000314615	arfgap1	7364	0.760429575
ensp00000354347	gfpt1	7365	0.760532838
ensp00000253778	gfpt2	7366	0.760636101
ensp00000341843	etv7	7368	0.760842627
ensp00000371833	pla2g4f	7369	0.76094589
ensp00000290472	pla2g4d	7370	0.761049153
ensp00000262764	pgs1	7371	0.761152416
ensp00000368140	crls1	7372	0.761255679
ensp00000419879	cds2	7373	0.761358943
ensp00000295887	cds1	7374	0.761462206
ensp00000324105	eno3	7375	0.761565469
ensp00000229277	eno2	7376	0.761668732
ensp00000351206	txlnb	7377	0.761771995
ensp00000291890	ncr1	7378	0.761875258
ensp00000299663	clec4e	7379	0.761978521
ensp00000262418	slc4a1	7380	0.762081784
ensp00000342793	pld1	7381	0.762185048
ensp00000263088	pld2	7382	0.762288311
ensp00000356436	pla2g4a	7383	0.762391574
ensp00000356438	ptgs2	7386	0.762701363
ensp00000299022	lipc	7387	0.762804626
ensp00000351695	pnliprp1	7390	0.763114416
ensp00000216180	pnpla3	7391	0.763217679
ensp00000228027	dgat2	7392	0.763320942
ensp00000291572	agpat3	7393	0.763424205
ensp00000314036	agpat4	7394	0.763527468
ensp00000360761	agpat2	7395	0.763630731
ensp00000310551	lclat1	7396	0.763733994
ensp00000324944	mboat1	7397	0.763837257
ensp00000302177	mboat2	7398	0.76394052
ensp00000412805	agpat1	7399	0.764043784
ensp00000381302	ppapdc1a	7405	0.764663362
ensp00000352547	gpat2	7406	0.764766625
ensp00000268099	scamp2	7407	0.764869888
ensp00000219919	aqp9	7408	0.764973152
ensp00000163416	golga5	7409	0.765076415

ensp00000355296	ldb3	7410	0.765179678
ensp00000228606	cyp27b1	7413	0.765489467
ensp00000233954	il1rl1	7414	0.76559273
ensp00000340089	tlr5	7415	0.765695993
ensp00000329213	siva1	7416	0.765799257
ensp00000266557	cd27	7417	0.76590252
ensp00000263932	tnfrsf8	7418	0.766005783
ensp00000337701	pnpla2	7419	0.766109046
ensp00000347581	agk	7420	0.766212309
ensp00000265052	mgll	7421	0.766315572
ensp00000348429	acsl5	7423	0.766522098
ensp00000258873	acsbg1	7424	0.766625361
ensp00000395005	mog	7426	0.766831888
ensp00000355963	lpgat1	7427	0.766935151
ensp00000355607	gnpat	7429	0.767141677
ensp00000301149	gpd1	7430	0.76724494
ensp00000282541	gpd1l	7431	0.767348203
ensp00000308610	gpd2	7432	0.767451466
ensp00000264409	agpat9	7433	0.767554729
ensp00000380184	agpat6	7434	0.767657993
ensp00000256720	lpin1	7435	0.767761256
ensp00000261596	lpin2	7436	0.767864519
ensp00000342082	slpi	7437	0.767967782
ensp00000309148	klk6	7438	0.768071045
ensp00000345719	tor1a	7439	0.768174308
ensp00000332052	pcsk6	7440	0.768277571
ensp00000380855	pdcd1lg2	7443	0.768587361
ensp00000370989	cd274	7444	0.768690624
ensp00000234831	tmem59	7448	0.769103676
ensp00000363079	mbl2	7449	0.769206939
ensp00000394863	ppt1	7450	0.769310202
ensp00000372853	c2	7451	0.769413466
ensp00000364321	c4b	7453	0.769619992
ensp00000366179	dnajc1	7455	0.769826518
ensp00000397101	mog	7456	0.769929781
ensp00000405864	agpat1	7457	0.770033044
ensp00000367462	olah	7458	0.770136307
ensp00000359410	ephx4	7459	0.77023957
ensp00000254260	rhpn2	7460	0.770342834
ensp00000261833	cit	7461	0.770446097
ensp00000356959	nr1i3	7463	0.770652623
ensp00000360541	cpt2	7464	0.770755886
ensp00000281455	acsl1	7465	0.770859149
ensp00000252669	acsbg2	7466	0.770962412
ensp00000339787	acsl4	7467	0.771065675
ensp00000350012	acsl3	7468	0.771168938
ensp00000310082	setd6	7469	0.771272202

ensp00000292147	ethe1	7470	0.771375465
ensp00000273814	dgkq	7472	0.771581991
ensp00000328405	dgka	7474	0.771788517
ensp00000255389	pemt	7475	0.77189178
ensp00000320043	lypla1	7476	0.771995043
ensp00000221249	pnpla6	7477	0.772098306
ensp00000384610	pnpla7	7478	0.77220157
ensp00000346228	pla2g4c	7479	0.772304833
ensp00000240617	plbd1	7480	0.772408096
ensp00000320337	pla2g16	7481	0.772511359
ensp00000257694	pnpla8	7482	0.772614622
ensp00000317300	lpcat4	7483	0.772717885
ensp00000245615	mboat7	7484	0.772821148
ensp00000304736	elovl6	7485	0.772924411
ensp00000361536	elovl1	7486	0.773027675
ensp00000278840	fads2	7487	0.773130938
ensp00000319851	chdh	7489	0.773337464
ensp00000308258	ptdss2	7490	0.773440727
ensp00000266095	pisd	7491	0.77354399
ensp00000261407	lpcat3	7492	0.773647253
ensp00000264005	lcat	7493	0.773750516
ensp00000354376	rab25	7494	0.773853779
ensp00000261890	rab11a	7495	0.773957043
ensp00000265846	adap1	7497	0.774163569
ensp00000297044	cyth3	7499	0.774370095
ensp00000262305	rab11fip3	7501	0.774576621
ensp00000258098	rab11fip5	7502	0.774679884
ensp00000331342	rab11fip1	7503	0.774783147
ensp00000312837	rab11fip4	7504	0.774886411
ensp00000259486	enpp2	7505	0.774989674
ensp00000219345	pla2g15	7506	0.775092937
ensp00000337331	ptdss1	7507	0.7751962
ensp00000229266	chpt1	7510	0.775505989
ensp00000247992	pla2g2c	7512	0.775712515
ensp00000312286	pla2g1b	7513	0.775815779
ensp00000325941	ric8a	7515	0.776022305
ensp00000223114	mogat3	7516	0.776125568
ensp00000332258	dgat1	7517	0.776228831
ensp00000261292	lipg	7518	0.776332094
ensp00000361151	cel	7520	0.77653862
ensp00000318852	bpnt1	7521	0.776641884
ensp00000406157	papss2	7523	0.77684841
ensp00000319501	ugdh	7524	0.776951673
ensp00000259938	clps	7525	0.777054936
ensp00000402935	tbc1d5	7526	0.777158199
ensp00000338742	sult1a2	7528	0.777364725
ensp00000277480	lcn2	7529	0.777467988

ensp00000218197	slc25a14	7530	0.777571252
ensp00000384432	icoslg	7531	0.777674515
ensp00000370880	mlana	7532	0.777777778
ensp00000345826	pmepa1	7533	0.777881041
ensp00000340118	litaf	7534	0.777984304
ensp00000355721	snap47	7535	0.778087567
ensp00000265026	map3k13	7536	0.77819083
ensp00000346160	dusp19	7537	0.778294093
ensp00000358501	cd58	7540	0.778603883
ensp00000341737	necap1	7541	0.778707146
ensp00000262215	arfgef1	7542	0.778810409
ensp00000360985	arfgef2	7543	0.778913672
ensp00000347244	itsn2	7544	0.779016935
ensp00000295324	cdc42ep3	7545	0.779120198
ensp00000258062	reps1	7547	0.779326724
ensp00000350018	sh3bp1	7548	0.779429988
ensp00000348128	svil	7550	0.779636514
ensp00000296721	afap1l1	7551	0.779739777
ensp00000348215	sh3pxd2a	7552	0.77984304
ensp00000363400	pafah2	7553	0.779946303
ensp00000262134	lpcat2	7554	0.780049566
ensp00000283415	lpcat1	7555	0.780152829
ensp00000371886	jmjd7-pla2g4b	7556	0.780256093
ensp00000364249	pla2g5	7557	0.780359356
ensp00000346693	elovl2	7559	0.780565882
ensp00000306640	elovl5	7560	0.780669145
ensp00000246949	dnase1	7561	0.780772408
ensp00000262428	cotl1	7562	0.780875671
ensp00000352657	me3	7563	0.780978934
ensp00000321070	me2	7564	0.781082197
ensp00000358719	me1	7565	0.781185461
ensp00000280706	ldhal6a	7566	0.781288724
ensp00000280704	ldhc	7567	0.781391987
ensp00000394382	pdha1	7570	0.781701776
ensp00000307241	pdhb	7571	0.781805039
ensp00000363390	trim63	7572	0.781908302
ensp00000315167	alox12b	7573	0.782011565
ensp00000330442	plb1	7575	0.782218092
ensp00000333142	pla2g6	7576	0.782321355
ensp00000393847	pla2g10	7579	0.782631144
ensp00000364246	pla2g2d	7580	0.782734407
ensp00000243501	pla2g12a	7581	0.78283767
ensp00000261891	dapk2	7583	0.783044197
ensp00000261007	chrna1	7584	0.78314746
ensp00000254667	ptpre	7585	0.783250723
ensp00000390673	vars	7586	0.783353986
ensp00000229340	rab35	7587	0.783457249

ensp00000254691	card6	7588	0.783560512
ensp00000297056	daglb	7589	0.783663775
ensp00000220764	decr1	7591	0.783870302
ensp00000306888	fam151a	7592	0.783973565
ensp00000360323	ttc22	7594	0.784180091
ensp00000303147	mat2a	7596	0.784386617
ensp00000357880	amd1	7598	0.784593143
ensp00000345341	ptges2	7599	0.784696406
ensp00000265641	cpt1a	7600	0.78479967
ensp00000409612	acadm	7601	0.784902933
ensp00000322229	fads1	7602	0.785006196
ensp00000360811	lhx3	7603	0.785109459
ensp00000306772	ldb2	7604	0.785212722
ensp00000417052	ebp	7607	0.785522511
ensp00000326305	slc25a20	7608	0.785625774
ensp00000312189	cpt1b	7609	0.785729038
ensp00000319343	cpt1c	7610	0.785832301
ensp00000325395	acadvl	7611	0.785935564
ensp00000233710	acadl	7612	0.786038827
ensp00000263552	tbxas1	7614	0.786245353
ensp00000342385	ptges	7615	0.786348616
ensp00000221307	cyp4f3	7616	0.786451879
ensp00000248041	cyp4f11	7617	0.786555143
ensp00000221700	cyp4f2	7618	0.786658406
ensp00000333212	cyp2u1	7619	0.786761669
ensp00000354612	ptgs1	7620	0.786864932
ensp00000290354	cbr3	7621	0.786968195
ensp00000290349	cbr1	7622	0.787071458
ensp00000295718	ptprn	7623	0.787174721
ensp00000252603	pgls	7624	0.787277984
ensp00000333019	adssl1	7626	0.787484511
ensp00000355493	adss	7627	0.787587774
ensp00000377527	pc	7628	0.787691037
ensp00000300051	ldhd	7629	0.7877943
ensp00000383611	ccnl2	7630	0.787897563
ensp00000271843	jtb	7631	0.788000826
ensp00000298032	armc3	7632	0.788104089
ensp00000257868	gdf11	7633	0.788207352
ensp00000376188	crlf1	7634	0.788310615
ensp00000271643	adamtsl4	7635	0.788413879
ensp00000377083	aldh1l1	7636	0.788517142
ensp00000371236	gart	7637	0.788620405
ensp00000264220	ppat	7638	0.788723668
ensp00000313490	pfas	7639	0.788826931
ensp00000360316	dhcr24	7640	0.788930194
ensp00000347717	dhcr7	7641	0.789033457
ensp00000419923	klf6	7642	0.78913672

ensp00000244043	ptgis	7643	0.789239983
ensp00000360687	ptgds	7644	0.789343247
ensp00000228740	lta4h	7646	0.789549773
ensp00000369858	alox5ap	7647	0.789653036
ensp00000292596	ltc4s	7648	0.789756299
ensp00000370571	th	7649	0.789859562
ensp00000250615	aanat	7650	0.789962825
ensp00000359297	nsdhl	7651	0.790066088
ensp00000000233	arf5	7652	0.790169352
ensp00000256682	arf3	7653	0.790272615
ensp00000370658	ccdc91	7654	0.790375878
ensp00000000412	m6pr	7655	0.790479141
ensp00000221957	plin3	7656	0.790582404
ensp00000382342	abcc1	7659	0.790892193
ensp00000307218	nat1	7660	0.790995456
ensp00000286479	nat2	7661	0.79109872
ensp00000368727	xdh	7662	0.791201983
ensp00000285949	cyp26c1	7664	0.791408509
ensp00000332679	cyp2a13	7666	0.791615035
ensp00000276651	dpys	7667	0.791718298
ensp00000324343	upb1	7668	0.791821561
ensp00000295755	retnlb	7671	0.792131351
ensp00000293761	alox15	7672	0.792234614
ensp00000360247	cyp2j2	7675	0.792544403
ensp00000369530	alox15b	7676	0.792647666
ensp00000363512	alox5	7677	0.792750929
ensp00000387261	pstpip2	7678	0.792854192
ensp00000356898	ddr2	7680	0.793060719
ensp00000296129	cdcp1	7681	0.793163982
ensp00000369843	ephx2	7683	0.793370508
ensp00000361507	hyi	7688	0.793886824
ensp00000274192	srd5a1	7691	0.794196613
ensp00000264228	srd5a3	7692	0.794299876
ensp00000358903	cyp17a1	7693	0.794403139
ensp00000240285	rdh10	7696	0.794712929
ensp00000316670	dhrs9	7697	0.794816192
ensp00000365397	dhrs3	7698	0.794919455
ensp00000326219	dhrs4	7699	0.795022718
ensp00000370750	rdh11	7700	0.795125981
ensp00000257895	rdh5	7701	0.795229244
ensp00000316786	hsd11b2	7702	0.795332507
ensp00000261465	hsd11b1	7703	0.79543577
ensp00000337224	lrat	7707	0.795848823
ensp00000341045	ugt2b15	7709	0.796055349
ensp00000369927	akr1c3	7711	0.796261875
ensp00000216286	nid2	7712	0.796365138
ensp00000416598	cyp21a2	7713	0.796468401

ensp00000260433	cyp19a1	7716	0.796778191
ensp00000318631	hsd17b6	7717	0.796881454
ensp00000369050	cyp1a1	7719	0.79708798
ensp00000201586	sult2b1	7721	0.797294506
ensp00000267502	rdh12	7723	0.797501033
ensp00000295802	retsat	7725	0.797707559
ensp00000370430	pnpla4	7726	0.797810822
ensp00000258168	bcmo1	7727	0.797914085
ensp00000224356	cyp26a1	7728	0.798017348
ensp00000249750	aldh1a2	7729	0.798120611
ensp00000297785	aldh1a1	7730	0.798223874
ensp00000304845	ugt1a8	7734	0.798636927
ensp00000303174	ugt1a6	7735	0.79874019
ensp00000304811	ugt2b7	7739	0.799153242
ensp00000407375	gpx1	7743	0.799566295
ensp00000241052	cat	7745	0.799772821
ensp00000387019	uxs1	7746	0.799876084
ensp00000369442	kl	7747	0.799979347
ensp00000252945	cyp2e1	7749	0.800185874
ensp00000324648	cyp2b6	7750	0.800289137
ensp00000260682	cyp2c9	7752	0.800495663
ensp00000311095	cyp4a11	7753	0.800598926
ensp00000353820	cyp2d6	7755	0.800805452
ensp00000263126	akr1c4	7756	0.800908715
ensp00000370254	akr1c1	7757	0.801011979
ensp00000221403	dhdh	7758	0.801115242
ensp00000346901	fmo1	7759	0.801218505
ensp00000341422	p4htm	7760	0.801321768
ensp00000216075	miox	7761	0.801425031
ensp00000334592	cyp2r1	7762	0.801528294
ensp00000265403	ugt2b10	7765	0.801838083
ensp00000199936	hsd17b2	7767	0.80204461
ensp00000413950	hsd17b8	7768	0.802147873
ensp00000225929	hsd17b1	7769	0.802251136
ensp00000278353	hsd17b12	7770	0.802354399
ensp00000263368	blvrb	7772	0.802560925
ensp00000260630	cyp1b1	7773	0.802664188
ensp00000337915	cyp3a4	7774	0.802767451
ensp00000337450	cyp3a7	7775	0.802870715
ensp00000222382	cyp3a43	7776	0.802973978
ensp00000222982	cyp3a5	7777	0.803077241
ensp00000272167	ephx1	7778	0.803180504
ensp00000378359	adh6	7781	0.803490293
ensp00000381206	rdh16	7783	0.803696819
ensp00000373477	gpx3	7785	0.803903346
ensp00000374265	gpx2	7786	0.804006609
ensp00000381607	gstp1	7787	0.804109872

ensp00000359998	gsta4	7788	0.804213135
ensp00000256594	gstm3	7789	0.804316398
ensp00000256593	gstm5	7790	0.804419661
ensp00000254090	fmo5	7792	0.804626188
ensp00000317842	ces2	7793	0.804729451
ensp00000302728	gusb	7794	0.804832714
ensp00000354511	comt	7795	0.804935977
ensp00000265512	adh4	7798	0.805245766
ensp00000296412	adh5	7799	0.805349029
ensp00000308032	cyp2s1	7800	0.805452292
ensp00000306522	camta1	7801	0.805555556
ensp00000361331	eri3	7802	0.805658819
ensp00000322159	atg4c	7803	0.805762082
ensp00000389591	apom	7804	0.805865345
ensp00000420168	gsta2	7805	0.805968608
ensp00000290765	gstt2b	7806	0.806071871
ensp00000284562	gsta5	7807	0.806175134
ensp00000401632	gstt1	7808	0.806278397
ensp00000211122	gsta3	7809	0.80638166
ensp00000241337	gstm2	7812	0.80669145
ensp00000345023	gsto2	7813	0.806794713
ensp00000265498	mgst2	7814	0.806897976
ensp00000275016	cyp39a1	7815	0.807001239
ensp00000312834	mlxip	7816	0.807104502
ensp00000360918	ch25h	7817	0.807207765
ensp00000258415	cyp27a1	7818	0.807311029
ensp00000337354	lipa	7819	0.807414292
ensp00000356591	soat1	7820	0.807517555
ensp00000301466	soat2	7821	0.807620818
ensp00000297679	hsd3b7	7823	0.807827344
ensp00000318867	cyp8b1	7824	0.807930607
ensp00000356723	fmo4	7825	0.80803387
ensp00000415786	serpine2	7827	0.808240397
ensp00000215727	serpind1	7828	0.80834366
ensp00000310036	cd34	7830	0.808550186
ensp00000300060	anpep	7831	0.808653449
ensp00000311469	gstm1	7832	0.808756712
ensp00000358851	gstm4	7833	0.808859975
ensp00000010404	mgst1	7834	0.808963238
ensp00000216465	gstz1	7836	0.809169765
ensp00000358727	gsto1	7837	0.809273028
ensp00000221130	gsr	7838	0.809376291
ensp00000266031	hyal1	7839	0.809479554
ensp00000217961	sts	7841	0.80968608
ensp00000268053	cyp11a1	7842	0.809789343
ensp00000310721	cyp7b1	7843	0.809892606
ensp00000416505	ppt2	7844	0.809995869

ensp00000293217	acox1	7845	0.810099133
ensp00000348775	acox3	7846	0.810202396
ensp00000255084	aldh3b2	7848	0.810408922
ensp00000225740	aldh3a1	7849	0.810512185
ensp00000332256	aldh1a3	7850	0.810615448
ensp00000296734	gpx8	7852	0.810821974
ensp00000238892	cript	7853	0.810925238
ensp00000300231	map1a	7854	0.811028501
ensp00000363054	stx12	7855	0.811131764
ensp00000333193	gjc1	7856	0.811235027
ensp00000317817	sh2d3c	7857	0.81133829
ensp00000313169	nphp1	7858	0.811441553
ensp00000216780	pck2	7859	0.811544816
ensp00000340510	ppl	7860	0.811648079
ensp00000219700	hmox2	7861	0.811751342
ensp00000395653	slc46a1	7862	0.811854606
ensp00000368226	gk	7863	0.811957869
ensp00000312134	prg2	7864	0.812061132
ensp00000395701	tapbp	7866	0.812267658
ensp00000361508	pltp	7867	0.812370921
ensp00000263094	abca7	7868	0.812474184
ensp00000262903	hace1	7869	0.812577447
ensp00000340454	rap1gds1	7870	0.81268071
ensp00000288368	prex2	7871	0.812783974
ensp00000222792	chn2	7872	0.812887237
ensp00000303909	abr	7873	0.8129905
ensp00000377380	arhgap9	7874	0.813093763
ensp00000386741	chn1	7875	0.813197026
ensp00000345436	caskin1	7877	0.813403552
ensp00000312606	akr1a1	7880	0.813713342
ensp00000285930	akr1b1	7881	0.813816605
ensp00000340684	maoa	7882	0.813919868
ensp00000367309	maob	7883	0.814023131
ensp00000253799	aoc2	7884	0.814126394
ensp00000312326	aoc3	7885	0.814229657
ensp00000377783	pros1	7890	0.814745973
ensp00000409489	neu1	7892	0.814952499
ensp00000385746	sms	7893	0.815055762
ensp00000325425	mat2b	7894	0.815159025
ensp00000315931	ahcyl2	7895	0.815262288
ensp00000353804	acss2	7896	0.815365551
ensp00000316924	acss1	7897	0.815468815
ensp00000261206	acss3	7898	0.815572078
ensp00000372191	sirt3	7899	0.815675341
ensp00000255192	bhmt2	7900	0.815778604
ensp00000274353	bhmt	7901	0.815881867
ensp00000367030	trdmt1	7902	0.81598513

ensp00000366156	srm	7903	0.816088393
ensp00000368572	sat1	7904	0.816191656
ensp00000269298	sat2	7905	0.816294919
ensp00000234111	odc1	7906	0.816398183
ensp00000350928	gad1	7909	0.816707972
ensp00000338964	ggt7	7911	0.816914498
ensp00000370962	ggt6	7912	0.817017761
ensp00000216951	gss	7913	0.817121024
ensp00000262430	mlycd	7916	0.817430814
ensp00000280346	dlat	7917	0.817534077
ensp00000253792	acly	7918	0.81763734
ensp00000290429	mcat	7919	0.817740603
ensp00000280701	oxsm	7920	0.817843866
ensp00000215882	slc25a1	7921	0.817947129
ensp00000362888	ado	7922	0.818050392
ensp00000280097	hnmt	7923	0.818153656
ensp00000256216	hsd17b4	7924	0.818256919
ensp00000248923	ggt1	7927	0.818566708
ensp00000381340	ggt5	7928	0.818669971
ensp00000260118	ggh	7929	0.818773234
ensp00000267085	csad	7930	0.818876497
ensp00000348234	tat	7932	0.819083024
ensp00000387536	pdk3	7934	0.81928955
ensp00000005178	pdk4	7935	0.819392813
ensp00000238618	acyp1	7936	0.819496076
ensp00000378161	acyp2	7937	0.819599339
ensp00000356015	acat2	7939	0.819805865
ensp00000265838	acat1	7940	0.819909128
ensp00000285093	acaa2	7942	0.820115655
ensp00000333664	acaa1	7943	0.820218918
ensp00000253513	ido1	7944	0.820322181
ensp00000350616	ddc	7945	0.820425444
ensp00000366927	aldh1b1	7947	0.82063197
ensp00000387123	aldh7a1	7948	0.820735233
ensp00000261733	aldh2	7949	0.820838496
ensp00000345774	aldh3a2	7950	0.82094176
ensp00000342564	aldh6a1	7951	0.821045023
ensp00000251654	pccb	7952	0.821148286
ensp00000365462	pcca	7953	0.821251549
ensp00000263702	mecr	7954	0.821354812
ensp00000264705	cad	7955	0.821458075
ensp00000255082	acy3	7956	0.821561338
ensp00000263080	aspa	7957	0.821664601
ensp00000175506	asns	7958	0.821767865
ensp00000265395	hibadh	7959	0.821871128
ensp00000293404	nags	7961	0.822077654
ensp00000360268	aldh18a1	7962	0.822180917

ensp00000290597	aldh4a1	7963	0.82228418
ensp00000277865	glud1	7964	0.822387443
ensp00000327589	glud2	7965	0.822490706
ensp00000314649	aldh5a1	7966	0.822593969
ensp00000268251	abat	7967	0.822697233
ensp00000351682	cndp1	7968	0.822800496
ensp00000267845	hdc	7969	0.822903759
ensp00000266971	suox	7970	0.823007022
ensp00000260324	sqrdl	7971	0.823110285
ensp00000222214	gcdh	7972	0.823213548
ensp00000231887	ehhadh	7973	0.823316811
ensp00000370023	hadha	7974	0.823420074
ensp00000265594	mccc1	7976	0.823626601
ensp00000343657	mccc2	7977	0.823729864
ensp00000253083	hip1r	7978	0.823833127
ensp00000329869	tpo	7979	0.82393639
ensp00000265758	wbscr22	7983	0.824349442
ensp00000390665	rims2	7984	0.824452705
ensp00000373006	micb	7985	0.824555969
ensp00000352706	hibch	7986	0.824659232
ensp00000363832	aox1	7987	0.824762495
ensp00000358814	ahcyl1	7989	0.824969021
ensp00000220966	pycrl	7990	0.825072284
ensp00000349577	prodh	7991	0.825175547
ensp00000342557	il4i1	7992	0.82527881
ensp00000245206	got2	7993	0.825382074
ensp00000359539	got1	7994	0.825485337
ensp00000249042	tst	7995	0.8255886
ensp00000377055	prcp	7997	0.825795126
ensp00000168216	hsd17b10	7999	0.826001652
ensp00000346378	apbb3	8000	0.826104915
ensp00000332170	p4ha3	8001	0.826208178
ensp00000263556	p4ha1	8002	0.826311442
ensp00000166534	p4ha2	8003	0.826414705
ensp00000226299	lap3	8004	0.826517968
ensp00000275428	ggct	8005	0.826621231
ensp00000359258	gclm	8006	0.826724494
ensp00000229416	gclc	8007	0.826827757
ensp00000250535	cdo1	8008	0.82693102
ensp00000342502	pycr2	8009	0.827034283
ensp00000328858	pycr1	8010	0.827137546
ensp00000263574	aplp2	8011	0.82724081
ensp00000221891	aplp1	8012	0.827344073
ensp00000377696	nit2	8013	0.827447336
ensp00000357920	ddo	8014	0.827550599
ensp00000335304	dlst	8015	0.827653862
ensp00000367923	sucla2	8016	0.827757125

ensp00000307432	suclg2	8017	0.827860388
ensp00000363614	hmgcl	8018	0.827963651
ensp00000312288	hadh	8019	0.828066914
ensp00000350914	bdh1	8020	0.828170178
ensp00000309259	alas1	8021	0.828273441
ensp00000322706	hmgcs1	8022	0.828376704
ensp00000346827	aldh9a1	8024	0.82858323
ensp00000361914	oxct2	8025	0.828686493
ensp00000196371	oxct1	8026	0.828789756
ensp00000324842	aacs	8027	0.828893019
ensp00000364883	auh	8028	0.828996283
ensp00000296424	bdh2	8029	0.829099546
ensp00000395102	ltb	8030	0.829202809
ensp00000264717	gckr	8031	0.829306072
ensp00000005260	baiap2l1	8036	0.829822387
ensp00000305956	sepsecs	8038	0.830028914
ensp00000359976	cth	8039	0.830132177
ensp00000367893	sephs1	8040	0.83023544
ensp00000254663	scly	8042	0.830441966
ensp00000307214	lcmt2	8044	0.830648492
ensp00000373300	mettl6	8045	0.830751755
ensp00000262432	mettl2b	8046	0.830855019
ensp00000333934	trmt11	8047	0.830958282
ensp00000379895	gatm	8049	0.831164808
ensp00000253004	ass1	8050	0.831268071
ensp00000307188	asl	8051	0.831371334
ensp00000342056	cs	8052	0.831474597
ensp00000233114	mdh1	8053	0.83157786
ensp00000327070	mdh2	8054	0.831681124
ensp00000328938	afmid	8055	0.831784387
ensp00000274813	mut	8056	0.83188765
ensp00000367992	esd	8057	0.831990913
ensp00000345580	slc25a10	8058	0.832094176
ensp00000281317	mmaa	8059	0.832197439
ensp00000244217	mcee	8060	0.832300702
ensp00000269980	bckdha	8061	0.832403965
ensp00000359151	dbt	8062	0.832507228
ensp00000318351	bckdhb	8063	0.832610492
ensp00000205402	dld	8064	0.832713755
ensp00000227868	pdhx	8065	0.832817018
ensp00000007708	pdk2	8066	0.832920281
ensp00000266736	amdhd1	8069	0.83323007
ensp00000294517	adc	8070	0.833333333
ensp00000261783	arg2	8071	0.833436596
ensp00000308351	mlkl	8076	0.833952912
ensp00000371634	igf2bp2	8077	0.834056175
ensp00000289004	hpd	8078	0.834159438

ensp00000331897	idh2	8079	0.834262701
ensp00000363216	ogdhl	8080	0.834365964
ensp00000299518	idh3a	8081	0.834469228
ensp00000217901	idh3g	8082	0.834572491
ensp00000370223	idh3b	8083	0.834675754
ensp00000260985	idh1	8084	0.834779017
ensp00000216254	aco2	8085	0.83488228
ensp00000175756	ptpn18	8087	0.835088806
ensp00000362711	txlna	8088	0.835192069
ensp00000339801	ids	8091	0.835501859
ensp00000314606	sgsh	8092	0.835605122
ensp00000225927	naglu	8093	0.835708385
ensp00000368965	hgsnat	8094	0.835811648
ensp00000266556	tapbpl	8096	0.836018174
ensp00000321184	igsf3	8097	0.836121437
ensp00000242592	acads	8101	0.83653449
ensp00000357873	acadsb	8102	0.836637753
ensp00000249760	ivd	8103	0.836741016
ensp00000281182	acad8	8104	0.836844279
ensp00000222673	ogdh	8106	0.837050805
ensp00000263035	dhtkd1	8107	0.837154069
ensp00000261434	lias	8108	0.837257332
ensp00000267436	l2hgdh	8110	0.837463858
ensp00000307900	glul	8111	0.837567121
ensp00000310447	gls2	8112	0.837670384
ensp00000317379	gls	8113	0.837773647
ensp00000325548	cndp2	8114	0.83787691
ensp00000295156	vsnl1	8115	0.837980173
ensp00000329452	slc25a21	8116	0.838083437
ensp00000264932	sdha	8117	0.8381867
ensp00000377446	suclg1	8118	0.838289963
ensp00000355518	fh	8120	0.838496489
ensp00000261755	fah	8121	0.838599752
ensp00000283871	hgd	8122	0.838703015
ensp00000226840	aadat	8123	0.838806278
ensp00000263182	bbox1	8124	0.838909542
ensp00000389175	glyctk	8125	0.839012805
ensp00000235521	wars2	8126	0.839116068
ensp00000347495	wars	8127	0.839219331
ensp00000248935	gstt1	8130	0.83952912
ensp00000322991	bcat2	8134	0.839942173
ensp00000261192	bcat1	8135	0.840045436
ensp00000318868	shmt1	8136	0.840148699
ensp00000385149	neu4	8139	0.840458488
ensp00000294064	neu3	8140	0.840561751
ensp00000343234	gal3st1	8141	0.840665014
ensp00000218516	gla	8142	0.840768278

ensp00000216124	arsa	8143	0.840871541
ensp00000333395	arsi	8144	0.840974804
ensp00000320219	arsj	8145	0.841078067
ensp00000370522	arsh	8147	0.841284593
ensp00000344460	cbs	8149	0.841491119
ensp00000257549	sds	8150	0.841594382
ensp00000342267	slc25a15	8152	0.841800909
ensp00000402608	cps1	8153	0.841904172
ensp00000309477	aco1	8154	0.842007435
ensp00000365773	psat1	8156	0.842213961
ensp00000358417	phgdh	8157	0.842317224
ensp00000299198	ckb	8158	0.842420487
ensp00000300176	agfg2	8160	0.842627014
ensp00000393776	fcho2	8161	0.842730277
ensp00000255189	dmgdh	8162	0.84283354
ensp00000360938	sardh	8163	0.842936803
ensp00000317721	pipox	8164	0.843040066
ensp00000361894	gnmt	8165	0.843143329
ensp00000231420	agxt2	8166	0.843246592
ensp00000302620	agxt	8168	0.843453119
ensp00000275605	psph	8169	0.843556382
ensp00000370546	arsd	8170	0.843659645
ensp00000369346	arsk	8171	0.843762908
ensp00000370526	arse	8172	0.843866171
ensp00000272902	sumf1	8173	0.843969434
ensp00000313432	grhpr	8174	0.844072697
ensp00000362463	glo1	8175	0.84417596
ensp00000331544	fbln1	8177	0.844382487
ensp00000408094	clic1	8178	0.84448575
ensp00000316339	hao2	8181	0.844795539
ensp00000330918	pgp	8182	0.844898802
ensp00000403536	gamt	8183	0.845002065
ensp00000283752	serpinb3	8184	0.845105328
ensp00000370074	serpinb9	8185	0.845208591
ensp00000341584	serpinb13	8186	0.845311855
ensp00000341952	haghl	8188	0.845518381
ensp00000380514	hagh	8189	0.845621644
ensp00000370737	gldc	8191	0.84582817
ensp00000332369	alas2	8192	0.845931433
ensp00000248924	gcat	8193	0.846034696
ensp00000381634	slc38a1	8194	0.84613796
ensp00000355890	eprs	8195	0.846241223
ensp00000223029	aimp2	8196	0.846344486
ensp00000369038	eef1e1	8197	0.846447749
ensp00000307567	qars	8198	0.846551012
ensp00000359804	fubp1	8199	0.846654275
ensp00000251507	rabgap1l	8200	0.846757538

ensp00000306906	rnf181	8202	0.846964064
ensp00000225665	slc25a11	8203	0.847067328
ensp00000381654	hmgcll1	8205	0.847273854
ensp00000017003	xylt2	8206	0.847377117
ensp00000303356	dcxr	8208	0.847583643
ensp00000225614	galk1	8209	0.847686906
ensp00000379766	galk2	8210	0.847790169
ensp00000302227	ccbl1	8212	0.847996696
ensp00000355517	kmo	8213	0.848099959
ensp00000264170	kynu	8214	0.848203222
ensp00000294973	haao	8215	0.848306485
ensp00000371554	sod3	8216	0.848409748
ensp00000392762	dct	8217	0.848513011
ensp00000358896	as3mt	8218	0.848616274
ensp00000348459	acmsd	8219	0.848719537
ensp00000205061	glg1	8221	0.848926064
ensp00000380903	robo3	8222	0.849029327
ensp00000204637	flt3lg	8223	0.84913259
ensp00000368119	galt	8226	0.849442379
ensp00000235345	slc35d1	8227	0.849545642
ensp00000356236	syt2	8228	0.849648905
ensp00000337103	chat	8229	0.849752169
ensp00000303211	ache	8230	0.849855432
ensp00000293780	chrne	8231	0.849958695
ensp00000372751	notch4	8232	0.850061958
ensp00000265537	lars2	8233	0.850165221
ensp00000377954	lars	8234	0.850268484
ensp00000364794	iars	8235	0.850371747
ensp00000327821	xpot	8236	0.85047501
ensp00000281828	farsb	8237	0.850578273
ensp00000373918	gars	8238	0.850681537
ensp00000282276	mars2	8239	0.8507848
ensp00000262027	mars	8240	0.850888063
ensp00000231572	rars	8241	0.850991326
ensp00000378191	aimp1	8243	0.851197852
ensp00000263897	use1	8244	0.851301115
ensp00000305810	stx18	8245	0.851404378
ensp00000231668	bnip1	8246	0.851507641
ensp00000220058	mtfmt	8247	0.851610905
ensp00000258494	aldh1l2	8248	0.851714168
ensp00000356290	mthfd1l	8249	0.851817431
ensp00000377094	rufy1	8250	0.851920694
ensp00000276297	dlc1	8251	0.852023957
ensp00000403397	zdhhc17	8253	0.852230483
ensp00000325448	kars	8254	0.852333746
ensp00000396308	dhfr	8255	0.85243701
ensp00000216605	mthfd1	8256	0.852540273

ensp00000377617	mthfd2	8257	0.852643536
ensp00000379108	mthfd2l	8258	0.852746799
ensp00000355536	mtr	8259	0.852850062
ensp00000291670	ftcd	8260	0.852953325
ensp00000273588	amt	8261	0.853056588
ensp00000365775	mthfr	8262	0.853159851
ensp00000258874	mthfs	8263	0.853263114
ensp00000281938	hspb8	8265	0.853469641
ensp00000227251	cryab	8266	0.853572904
ensp00000288048	c1orf158	8268	0.85377943
ensp00000260197	sorl1	8269	0.853882693
ensp00000374135	lrp1b	8270	0.853985956
ensp00000336552	pkia	8271	0.854089219
ensp00000262461	slc12a2	8272	0.854192482
ensp00000155840	kcnq1	8273	0.854295746
ensp00000262186	kcnh2	8274	0.854399009
ensp00000371936	sema5a	8275	0.854502272
ensp00000340017	doc2a	8276	0.854605535
ensp00000259526	nov	8277	0.854708798
ensp00000311984	carkd	8279	0.854915324
ensp00000357218	apoa1bp	8280	0.855018587
ensp00000361759	bex2	8281	0.85512185
ensp00000349727	dok3	8283	0.855328377
ensp00000347532	kiaa1598	8284	0.85543164
ensp00000307265	irf2bp1	8286	0.855638166
ensp00000317177	pld6	8288	0.855844692
ensp00000348901	pld3	8289	0.855947955
ensp00000323837	slc4a1ap	8290	0.856051219
ensp00000285379	ca2	8291	0.856154482
ensp00000319531	gcsh	8292	0.856257745
ensp00000342087	fhit	8293	0.856361008
ensp00000384979	lingo3	8294	0.856464271
ensp00000262395	traf4	8295	0.856567534
ensp00000292823	pcyt1a	8297	0.85677406
ensp00000331719	pcyt2	8298	0.856877323
ensp00000406909	phospho1	8299	0.856980587
ensp00000265689	chka	8300	0.85708385
ensp00000384400	chkb	8301	0.857187113
ensp00000271227	slc44a3	8302	0.857290376
ensp00000342071	lipt1	8303	0.857393639
ensp00000309463	lipt2	8304	0.857496902
ensp00000311713	oxsr1	8305	0.857600165
ensp00000254878	hrsp12	8306	0.857703428
ensp00000274721	gfra3	8307	0.857806691
ensp00000258385	chrnd	8315	0.858632796
ensp00000298854	rapsn	8316	0.858736059
ensp00000262018	sgca	8317	0.858839323

ensp00000225573	pnpo	8318	0.858942586
ensp00000215904	pdxp	8319	0.859045849
ensp00000291565	pdxk	8320	0.859149112
ensp00000352782	phospho2	8321	0.859252375
ensp00000266517	etnk1	8322	0.859355638
ensp00000356170	etnk2	8323	0.859458901
ensp00000341117	sdsl	8324	0.859562164
ensp00000339435	srr	8325	0.859665428
ensp00000216484	sptlc2	8326	0.859768691
ensp00000262554	sptlc1	8327	0.859871954
ensp00000381968	sptlc3	8328	0.859975217
ensp00000335261	tmlhe	8329	0.86007848
ensp00000366902	zbtb48	8330	0.860181743
ensp00000245304	rap2a	8331	0.860285006
ensp00000388658	slc25a12	8332	0.860388269
ensp00000361642	ppcs	8333	0.860491532
ensp00000268164	st8sia2	8334	0.860594796
ensp00000231461	st8sia4	8335	0.860698059
ensp00000304858	ormdl3	8336	0.860801322
ensp00000363296	grm4	8339	0.861111111
ensp00000344173	grm8	8340	0.861214374
ensp00000245903	cd70	8342	0.8614209
ensp00000293677	raver1	8343	0.861524164
ensp00000301607	evpl	8344	0.861627427
ensp00000357986	plekha1	8346	0.861833953
ensp00000393860	plekha2	8347	0.861937216
ensp00000298923	slc6a5	8348	0.862040479
ensp00000321826	stxbp5	8350	0.862247005
ensp00000278618	aasdhppt	8351	0.862350268
ensp00000343190	ppcdc	8353	0.862556795
ensp00000239231	pank3	8354	0.862660058
ensp00000367727	pank4	8355	0.862763321
ensp00000345997	dmpk	8358	0.86307311
ensp00000261772	aars	8359	0.863176373
ensp00000364699	sdhd	8361	0.8633829
ensp00000356953	sdhc	8362	0.863486163
ensp00000364649	sdhb	8363	0.863589426
ensp00000303727	chrna7	8366	0.863899215
ensp00000356202	mtrf1l	8367	0.864002478
ensp00000302935	il16	8369	0.864209005
ensp00000353462	cass4	8371	0.864415531
ensp00000297273	casd1	8372	0.864518794
ensp00000357461	chrnb2	8374	0.86472532
ensp00000276410	chrna6	8376	0.864931846
ensp00000262052	slc11a2	8378	0.865138373
ensp00000261024	slc40a1	8379	0.865241636
ensp00000356905	vnn1	8380	0.865344899

ensp00000322276	vnn2	8381	0.865448162
ensp00000315013	crat	8382	0.865551425
ensp00000344903	mafk	8384	0.865757951
ensp00000350369	mafg	8385	0.865861214
ensp00000250378	cma1	8386	0.865964477
ensp00000201647	eps8l1	8387	0.866067741
ensp00000363649	akap2	8388	0.866171004
ensp00000360806	kcnb1	8389	0.866274267
ensp00000305824	kcns3	8390	0.86637753
ensp00000319141	cybrd1	8392	0.866584056
ensp00000385142	nrxn1	8394	0.866790582
ensp00000265459	nrxn2	8395	0.866893846
ensp00000372326	fech	8397	0.867100372
ensp00000264613	cp	8398	0.867203635
ensp00000264998	tf	8399	0.867306898
ensp00000353224	tfrc	8400	0.867410161
ensp00000417404	hfe	8401	0.867513424
ensp00000305288	nlgn2	8403	0.86771995
ensp00000223051	tfr2	8404	0.867823214
ensp00000307697	acox2	8405	0.867926477
ensp00000398890	hla-dmb	8406	0.86802974
ensp00000372718	hla-dmb	8407	0.868133003
ensp00000334424	amacr	8408	0.868236266
ensp00000325663	nfkbiz	8410	0.868442792
ensp00000239882	elf1	8411	0.868546055
ensp00000258403	slc19a3	8412	0.868649318
ensp00000245312	slc10a2	8413	0.868752582
ensp00000285238	abcc3	8414	0.868855845
ensp00000377549	fabp6	8415	0.868959108
ensp00000414024	gpsm3	8416	0.869062371
ensp00000230236	hsd17b8	8417	0.869165634
ensp00000257749	bach2	8418	0.869268897
ensp00000345393	maff	8419	0.86937216
ensp00000312436	nfe2	8420	0.869475423
ensp00000002829	sema3f	8424	0.869888476
ensp00000305974	slco1a2	8427	0.870198265
ensp00000259396	orm1	8431	0.870611318
ensp00000296557	arfip1	8432	0.870714581
ensp00000271751	kcnh1	8434	0.870921107
ensp00000221770	pop4	8438	0.871334159
ensp00000339529	pop1	8439	0.871437423
ensp00000264649	atp6v0a1	8440	0.871540686
ensp00000253856	atp6v0a4	8441	0.871643949
ensp00000265093	atp6v0e1	8442	0.871747212
ensp00000358802	kcnc4	8444	0.871953738
ensp00000218176	kcnd1	8445	0.872057001
ensp00000312129	kcng4	8446	0.872160264

ensp00000307694	kcns1	8449	0.872470054
ensp00000371514	kcnv2	8451	0.87267658
ensp00000221444	kcna7	8453	0.872883106
ensp00000318212	kcnh6	8456	0.873192895
ensp00000302719	kcnab3	8457	0.873296159
ensp00000262916	kcnq4	8458	0.873399422
ensp00000374125	siglec15	8460	0.873605948
ensp00000317337	cd300lb	8462	0.873812474
ensp00000354541	nlgn1	8463	0.873915737
ensp00000358784	kcna3	8468	0.874432053
ensp00000359425	kcnq5	8469	0.874535316
ensp00000295082	kcnf1	8470	0.874638579
ensp00000360626	kcng1	8471	0.874741842
ensp00000297404	kcnv1	8472	0.874845105
ensp00000257981	kcnh3	8473	0.874948368
ensp00000352035	kcnq2	8474	0.875051632
ensp00000373648	kcnq3	8475	0.875154895
ensp00000164247	kcnab2	8481	0.875774473
ensp00000319591	kcnd3	8482	0.875877736
ensp00000333496	kcnd2	8483	0.875981
ensp00000337255	kcne1	8484	0.876084263
ensp00000313377	pank2	8485	0.876187526
ensp00000395323	kcnip1	8486	0.876290789
ensp00000222726	hoxa5	8488	0.876497315
ensp00000323568	slc2a2	8489	0.876600578
ensp00000265162	enpep	8491	0.876807105
ensp00000343445	serpinb4	8492	0.876910368
ensp00000367697	atp6ap2	8493	0.877013631
ensp00000252519	ace2	8494	0.877116894
ensp00000369519	mtap	8495	0.877220157
ensp00000284719	ola1	8496	0.87732342
ensp00000040663	mri1	8497	0.877426683
ensp00000333666	adi1	8498	0.877529946
ensp00000350098	pop5	8499	0.877633209
ensp00000207870	xylb	8501	0.877839736
ensp00000329312	igll1	8504	0.878149525
ensp00000278359	apip	8505	0.878252788
ensp00000273920	enoph1	8506	0.878356051
ensp00000245551	mif4gd	8507	0.878459314
ensp00000327453	acsm2b	8508	0.878562577
ensp00000289416	acsm3	8510	0.878769104
ensp00000327916	acsm5	8511	0.878872367
ensp00000336888	slc44a2	8513	0.879078893
ensp00000363852	slc44a1	8514	0.879182156
ensp00000353679	mme	8516	0.879388682
ensp00000217131	ctsz	8517	0.879491945
ensp00000290866	ace	8518	0.879595209

ensp00000236067	atp6v0b	8520	0.879801735
ensp00000329757	atp6v0c	8521	0.879904998
ensp00000265686	tcirg1	8523	0.880111524
ensp00000304891	atp6v1e2	8525	0.88031805
ensp00000276390	atp6v1b2	8526	0.880421314
ensp00000363162	atp6v1g1	8527	0.880524577
ensp00000379203	atp6v1c1	8528	0.88062784
ensp00000272238	atp6v1c2	8529	0.880731103
ensp00000411672	atp6v0e2	8530	0.880834366
ensp00000296350	mfi2	8531	0.880937629
ensp00000346874	far1	8532	0.881040892
ensp00000264167	agps	8533	0.881144155
ensp00000182377	far2	8534	0.881247418
ensp00000313691	ftmt	8535	0.881350682
ensp00000394936	orm2	8537	0.881557208
ensp00000369820	piga	8538	0.881660471
ensp00000216452	pigh	8539	0.881763734
ensp00000315659	dnmbp	8540	0.881866997
ensp00000258390	dock10	8541	0.88197026
ensp00000301200	cdc42ep5	8542	0.882073523
ensp00000279249	cdc42ep2	8543	0.882176786
ensp00000376822	steap3	8544	0.88228005
ensp00000293195	fdxr	8545	0.882383313
ensp00000225688	rasd1	8546	0.882486576
ensp00000368790	mtrf1	8547	0.882589839
ensp00000357973	lace1	8548	0.882693102
ensp00000295440	nppc	8549	0.882796365
ensp00000365651	nppb	8550	0.882899628
ensp00000398028	npr3	8551	0.883002891
ensp00000338927	znf385a	8552	0.883106154
ensp00000344909	ptp4a2	8553	0.883209418
ensp00000358777	atp6ap1	8555	0.883415944
ensp00000352522	atp6v1h	8556	0.883519207
ensp00000290949	atp6v0d1	8557	0.88362247
ensp00000285393	atp6v0d2	8558	0.883725733
ensp00000249289	atp6v1f	8559	0.883828996
ensp00000216442	atp6v1d	8560	0.883932259
ensp00000273398	atp6v1a	8561	0.884035523
ensp00000253413	atp6v1e1	8562	0.884138786
ensp00000317473	rabggtb	8563	0.884242049
ensp00000319210	rph3al	8564	0.884345312
ensp00000264079	mcoln1	8565	0.884448575
ensp00000266088	slc5a1	8566	0.884551838
ensp00000366641	slc2a5	8567	0.884655101
ensp00000268695	galns	8568	0.884758364
ensp00000265112	tars	8572	0.885171417
ensp00000256854	nars	8573	0.88527468

ensp00000343313	atg5	8574	0.885377943
ensp00000274459	atg12	8575	0.885481206
ensp00000375872	atg16l1	8576	0.885584469
ensp00000366662	ca6	8577	0.885687732
ensp00000334415	fam183a	8579	0.885894259
ensp00000357835	lhpp	8580	0.885997522
ensp00000343885	ppa2	8582	0.886204048
ensp00000265447	anxa11	8583	0.886307311
ensp00000268261	pmm2	8584	0.886410574
ensp00000216259	pmm1	8585	0.886513837
ensp00000306920	glb1	8587	0.886720363
ensp00000306459	b4galt6	8588	0.886823627
ensp00000248929	sgsm3	8589	0.88692689
ensp00000323816	ahrr	8590	0.887030153
ensp00000417764	alg2	8591	0.887133416
ensp00000348753	spred2	8592	0.887236679
ensp00000407088	rgl2	8593	0.887339942
ensp00000292475	atp5j2	8595	0.887546468
ensp00000389649	atp5j	8596	0.887649732
ensp00000300688	atp5l	8597	0.887752995
ensp00000301587	atp5h	8598	0.887856258
ensp00000284727	atp5g3	8600	0.888062784
ensp00000377878	atp5g2	8601	0.888166047
ensp00000295566	yy1ap1	8605	0.8885791
ensp00000406872	hla-dqb2	8607	0.888785626
ensp00000375622	lair1	8609	0.888992152
ensp00000198536	pilra	8610	0.889095415
ensp00000161559	ceacam1	8612	0.889301941
ensp00000348205	atp5g1	8613	0.889405204
ensp00000215375	atp5d	8614	0.889508468
ensp00000262030	atp5b	8615	0.889611731
ensp00000349142	atp5c1	8616	0.889714994
ensp00000282050	atp5a1	8617	0.889818257
ensp00000355190	nfe2l1	8618	0.88992152
ensp00000367124	slc3a2	8619	0.890024783
ensp00000271638	s100a11	8620	0.890128046
ensp00000401770	c14orf2	8621	0.890231309
ensp00000243997	atp5e	8622	0.890334572
ensp00000306003	atp5i	8623	0.890437836
ensp00000290299	atp5o	8624	0.890541099
ensp00000358737	atp5f1	8625	0.890644362
ensp00000228318	slc25a3	8626	0.890747625
ensp00000362010	anxa7	8627	0.890850888
ensp00000299565	chrna5	8628	0.890954151
ensp00000308334	atp5s	8629	0.891057414
ensp00000418661	ptrh1	8630	0.891160677
ensp00000347504	itgb1bp1	8632	0.891367204

ensp00000261918	sema7a	8633	0.891470467
ensp00000258526	plxnc1	8634	0.89157373
ensp00000409159	hla-dqb2	8635	0.891676993
ensp00000269701	akap8	8636	0.891780256
ensp00000356465	rab32	8637	0.891883519
ensp00000228850	akap3	8638	0.891986782
ensp00000384084	dtnb	8639	0.892090045
ensp00000298352	ngb	8643	0.892503098
ensp00000252951	hbz	8644	0.892606361
ensp00000251595	hba2	8645	0.892709624
ensp00000369654	hbd	8647	0.89291615
ensp00000199708	hbq1	8650	0.89322594
ensp00000348170	hp	8651	0.893329203
ensp00000293230	cygb	8652	0.893432466
ensp00000372995	atp6v1g2	8653	0.893535729
ensp00000406389	atp6v1g2	8654	0.893638992
ensp00000371372	atp12a	8656	0.893845518
ensp00000352835	mb	8658	0.894052045
ensp00000261622	slc7a5	8660	0.894258571
ensp00000285850	slc7a7	8661	0.894361834
ensp00000219343	slc7a6	8662	0.894465097
ensp00000295888	wdfy3	8663	0.89456836
ensp00000266458	gabarapl1	8664	0.894671623
ensp00000037243	gabarapl2	8665	0.894774886
ensp00000268607	map1lc3b	8666	0.89487815
ensp00000306866	gabarap	8667	0.894981413
ensp00000320378	slc7a8	8668	0.895084676
ensp00000280612	slc7a11	8669	0.895187939
ensp00000283290	atg3	8670	0.895291202
ensp00000346437	atg7	8671	0.895394465
ensp00000384259	atg4b	8672	0.895497728
ensp00000262394	wsb1	8673	0.895600991
ensp00000253814	ndfip1	8675	0.895807518
ensp00000264954	grpel1	8677	0.896014044
ensp00000318115	timm50	8678	0.896117307
ensp00000248114	gfer	8679	0.89622057
ensp00000387578	hla-dqb2	8680	0.896323833
ensp00000370839	sgcb	8682	0.896530359
ensp00000249269	pmpcb	8685	0.896840149
ensp00000270538	timm44	8687	0.897046675
ensp00000252487	tomm40	8689	0.897253201
ensp00000329558	grpel2	8690	0.897356464
ensp00000260867	timm23	8692	0.89756299
ensp00000257245	timm10	8694	0.897769517
ensp00000320236	timm22	8695	0.89787278
ensp00000355077	nsmce1	8696	0.897976043
ensp00000358019	nsmce4a	8697	0.898079306

ensp00000323439	smc6	8698	0.898182569
ensp00000354957	smc5	8699	0.898285832
ensp00000287437	nsmce2	8700	0.898389095
ensp00000357360	mtx1	8701	0.898492359
ensp00000345445	samm50	8702	0.898595622
ensp00000249442	mtx2	8703	0.898698885
ensp00000372005	dnajc19	8705	0.898905411
ensp00000356256	timm17a	8706	0.899008674
ensp00000284320	tomm70a	8707	0.899111937
ensp00000384411	tomm5	8708	0.8992152
ensp00000355566	tomm20	8709	0.899318463
ensp00000216034	tomm22	8710	0.899421727
ensp00000381856	tomm6	8711	0.89952499
ensp00000295767	chchd4	8712	0.899628253
ensp00000262262	cd33	8714	0.899834779
ensp00000312370	aifm2	8715	0.899938042
ensp00000297185	hspa9	8716	0.900041305
ensp00000329715	drg1	8717	0.900144568
ensp00000282185	atg10	8721	0.900557621
ensp00000311502	heg1	8722	0.900660884
ensp00000382025	hla-dqb1	8723	0.900764147
ensp00000321810	trit1	8724	0.90086741
ensp00000391735	slc39a7	8725	0.900970673
ensp00000397139	hla-dpa1	8726	0.901073936
ensp00000389288	hla-dpb1	8727	0.9011772
ensp00000260270	fdx1	8728	0.901280463
ensp00000244051	mocs3	8729	0.901383726
ensp00000363205	nfs1	8730	0.901486989
ensp00000216027	hscb	8732	0.901693515
ensp00000365159	isca1	8733	0.901796778
ensp00000261070	cox17	8734	0.901900041
ensp00000377311	fdx1l	8735	0.902003304
ensp00000412922	urm1	8737	0.902209831
ensp00000338788	zc3h15	8738	0.902313094
ensp00000375093	tas2r31	8740	0.90251962
ensp00000364697	proz	8744	0.902932672
ensp00000387892	hla-dqa1	8745	0.903035936
ensp00000409127	hla-dqa1	8746	0.903139199
ensp00000229708	ulbp1	8749	0.903448988
ensp00000344218	c1orf162	8753	0.90386204
ensp00000244709	trem1	8754	0.903965304
ensp00000259206	il1rn	8756	0.90417183
ensp00000264257	il1rl2	8757	0.904275093
ensp00000407561	micb	8758	0.904378356
ensp00000402134	mica	8760	0.904584882
ensp00000356320	ulbp2	8763	0.904894672
ensp00000311427	snx33	8765	0.905101198

ensp00000363349	snx30	8766	0.905204461
ensp00000348455	uvrag	8767	0.905307724
ensp00000220509	vps18	8772	0.90582404
ensp00000326534	vps39	8773	0.905927303
ensp00000369810	vps16	8775	0.906133829
ensp00000309457	vps41	8776	0.906237092
ensp00000353701	dpp3	8777	0.906340355
ensp00000246062	mkks	8779	0.906546881
ensp00000319062	bbs12	8780	0.906650145
ensp00000376946	bbs10	8781	0.906753408
ensp00000245157	bbs2	8782	0.906856671
ensp00000281243	qdpr	8783	0.906959934
ensp00000254908	pcbd2	8784	0.907063197
ensp00000295240	bbs5	8785	0.90716646
ensp00000242067	bbs9	8786	0.907269723
ensp00000370031	ttc8	8787	0.907372986
ensp00000268057	bbs4	8788	0.907476249
ensp00000356903	uap1	8790	0.907682776
ensp00000260257	fdxacb1	8791	0.907786039
ensp00000239891	alg5	8792	0.907889302
ensp00000361625	dolpp1	8793	0.907992565
ensp00000370194	gmds	8794	0.908095828
ensp00000262374	alg1	8795	0.908199091
ensp00000333813	alg12	8797	0.908405618
ensp00000258324	pigc	8799	0.908612144
ensp00000420037	pigp	8800	0.908715407
ensp00000322181	dpm2	8801	0.90881867
ensp00000225609	pigl	8802	0.908921933
ensp00000315925	gmppa	8803	0.909025196
ensp00000309092	gmppb	8804	0.909128459
ensp00000357384	dpm3	8806	0.909334986
ensp00000380793	alg3	8807	0.909438249
ensp00000296292	rft1	8808	0.909541512
ensp00000327077	pcm1	8809	0.909644775
ensp00000361047	alg13	8810	0.909748038
ensp00000359224	alg14	8811	0.909851301
ensp00000346142	dpagt1	8812	0.909954564
ensp00000361667	dolk	8813	0.910057827
ensp00000215570	timm13	8815	0.910264354
ensp00000361993	timm8a	8816	0.910367617
ensp00000357069	pigm	8817	0.91047088
ensp00000317301	pigx	8818	0.910574143
ensp00000078527	pigv	8819	0.910677406
ensp00000350263	pign	8820	0.910780669
ensp00000267199	vps33a	8821	0.910883932
ensp00000234454	spr	8822	0.910987195
ensp00000361852	tmco2	8824	0.911193722

ensp00000321674		4-Sep	8825	0.911296985
ensp00000332313	pigw		8829	0.911710037
ensp00000346809	pgap1		8831	0.911916563
ensp00000359848	pigk		8832	0.912019827
ensp00000164305	pigb		8833	0.91212309
ensp00000283977	pgm3		8834	0.912226353
ensp00000244204	nagk		8835	0.912329616
ensp00000341828	chia		8836	0.912432879
ensp00000337722	arl6		8838	0.912639405
ensp00000386284	alad		8839	0.912742668
ensp00000246337	urod		8840	0.912845931
ensp00000343943	ppox		8841	0.912949195
ensp00000326579	hccs		8842	0.913052458
ensp00000261643	cox10		8843	0.913155721
ensp00000278715	hmbs		8844	0.913258984
ensp00000264193	cpox		8845	0.913362247
ensp00000357775	uros		8846	0.91346551
ensp00000016171	cox15		8847	0.913568773
ensp00000355627	agt		8848	0.913672036
ensp00000272190	ren		8849	0.913775299
ensp00000393355	micb		8850	0.913878563
ensp00000391681	pou5f1		8851	0.913981826
ensp00000386456	aak1		8852	0.914085089
ensp00000366603	tgoln2		8853	0.914188352
ensp00000386935	uap1l1		8854	0.914291615
ensp00000379839	gne		8855	0.914394878
ensp00000377303	renbp		8856	0.914498141
ensp00000357209	ptprk		8857	0.914601404
ensp00000359512	gbp3		8858	0.914704667
ensp00000386200	foxp2		8859	0.914807931
ensp00000318902	foxp1		8860	0.914911194
ensp00000261416	hexb		8862	0.91511772
ensp00000268097	hexa		8863	0.915220983
ensp00000366549	st3gal5		8864	0.915324246
ensp00000414880	b3galt4		8865	0.915427509
ensp00000349687	gm2a		8866	0.915530772
ensp00000249005	a4galt		8867	0.915634036
ensp00000255427	chit1		8868	0.915737299
ensp00000255409	chi3l1		8869	0.915840562
ensp00000362873	ndufa8		8870	0.915943825
ensp00000362058	ndufs5		8871	0.916047088
ensp00000357507	c1orf43		8872	0.916150351
ensp00000309823	foxp4		8874	0.916356877
ensp00000258317	npl		8875	0.91646014
ensp00000210444	nans		8876	0.916563404
ensp00000229329	cmas		8877	0.916666667
ensp00000302441	nanp		8878	0.91676993

ensp00000352603	ap4m1	8880	0.916976456
ensp00000347408	ap3m1	8881	0.917079719
ensp00000252825	hrc	8883	0.917286245
ensp00000372734	hla-dqb1	8884	0.917389508
ensp00000372738	hla-dqa1	8885	0.917492772
ensp00000304229	hint1	8886	0.917596035
ensp00000267842	slc27a2	8887	0.917699298
ensp00000407401	pex5	8888	0.917802561
ensp00000315680	pex7	8889	0.917905824
ensp00000225873	pex12	8890	0.918009087
ensp00000390722	slc25a17	8892	0.918215613
ensp00000218104	abcd1	8893	0.918318876
ensp00000356563	pex3	8894	0.91842214
ensp00000295030	pex13	8895	0.918525403
ensp00000357051	pex19	8897	0.918731929
ensp00000299335	cox11	8899	0.918938455
ensp00000359233	abcd3	8900	0.919041718
ensp00000247655	cox7c	8901	0.919144981
ensp00000417656	cox7b	8905	0.919558034
ensp00000297564	cox6c	8906	0.919661297
ensp00000317780	cox5a	8907	0.91976456
ensp00000263774	ndufs3	8908	0.919867823
ensp00000360492	ndufa1	8909	0.919971086
ensp00000346196	ndufv3	8911	0.920177613
ensp00000215565	ndufb7	8912	0.920280876
ensp00000233627	ndufs7	8913	0.920384139
ensp00000322450	ndufv1	8914	0.920487402
ensp00000276062	ndufb11	8916	0.920693928
ensp00000007516	ndufab1	8917	0.920797191
ensp00000259037	ndufb5	8918	0.920900454
ensp00000330737	ndufa12	8919	0.921003717
ensp00000311740	ndufa11	8920	0.921106981
ensp00000230459	cox7a2	8922	0.921313507
ensp00000362576	yars	8923	0.92141677
ensp00000356789	atp1b1	8924	0.921520033
ensp00000268379	uqcrc2	8925	0.921623296
ensp00000306397	uqcrfs1	8926	0.921726559
ensp00000317159	cyc1	8927	0.921829822
ensp00000233190	ndufs1	8928	0.921933086
ensp00000356972	ndufs2	8929	0.922036349
ensp00000266544	ndufa9	8930	0.922139612
ensp00000296684	ndufs4	8931	0.922242875
ensp00000252102	ndufa2	8932	0.922346138
ensp00000276689	ndufb9	8933	0.922449401
ensp00000398290	ndufa3	8934	0.922552664
ensp00000339720	ndufa4	8935	0.922655927
ensp00000315774	ndufs8	8939	0.92306898

ensp00000327268	ndufv2	8940	0.923172243
ensp00000267950	etfa	8941	0.923275506
ensp00000346173	etfb	8942	0.923378769
ensp00000303552	etfdh	8943	0.923482032
ensp00000217446	pigu	8944	0.923585295
ensp00000347206	gpaa1	8945	0.923688558
ensp00000367934	uqcrq	8946	0.923791822
ensp00000287022	uqcrb	8947	0.923895085
ensp00000203407	uqcrc1	8948	0.923998348
ensp00000309565	uqcrh	8951	0.924308137
ensp00000347988	ndufa5	8953	0.924514663
ensp00000184266	ndufb4	8954	0.924617926
ensp00000330787	ndufb1	8956	0.924824453
ensp00000237889	ndufb3	8958	0.925030979
ensp00000274137	ndufs6	8960	0.925237505
ensp00000268668	ndufb10	8961	0.925340768
ensp00000281031	ndufc2	8963	0.925547295
ensp00000299166	ndufb8	8965	0.925753821
ensp00000252711	ndufa10	8966	0.925857084
ensp00000377770	ndufc1	8967	0.925960347
ensp00000260361	ndufaf1	8968	0.92606361
ensp00000261729	rasal1	8970	0.926270136
ensp00000299084	spred1	8971	0.926373399
ensp00000369897	cars	8972	0.926476663
ensp00000263038	phyh	8974	0.926683189
ensp00000352219	bcs1l	8975	0.926786452
ensp00000258424	cox5b	8976	0.926889715
ensp00000321260	cox8a	8977	0.926992978
ensp00000246554	cox6b1	8978	0.927096241
ensp00000253452	cox4i1	8979	0.927199504
ensp00000234301	cox7a2l	8980	0.927302767
ensp00000369176	ndufb6	8981	0.927406031
ensp00000247866	ndufb2	8982	0.927509294
ensp00000265500	ndufc1	8983	0.927612557
ensp00000323811	hacl1	8984	0.92771582
ensp00000333122	nr4a3	8987	0.928025609
ensp00000353238	spn	8988	0.928128872
ensp00000323071	acot4	8990	0.928335399
ensp00000316329	scd5	8991	0.928438662
ensp00000222120	rab3d	8993	0.928645188
ensp00000263150	wdr37	8994	0.928748451
ensp00000373420	rufy2	8995	0.928851714
ensp00000389516	agpat1	8996	0.928954977
ensp00000405502	egfl8	8997	0.92905824
ensp00000361110	gbgt1	8998	0.929161504
ensp00000323479	b3galnt1	8999	0.929264767
ensp00000281382	pigf	9000	0.92936803

ensp00000339382	pigo	9001	0.929471293
ensp00000415203	pigg	9002	0.929574556
ensp00000338127	tesk1	9003	0.929677819
ensp00000344967	spry4	9004	0.929781082
ensp00000264605	mlph	9005	0.929884345
ensp00000337761	rab27a	9006	0.929987608
ensp00000262094	rab27b	9007	0.930090872
ensp00000265843	exph5	9008	0.930194135
ensp00000353631	sytl3	9009	0.930297398
ensp00000346576	sytl2	9011	0.930503924
ensp00000199764	ceacam6	9012	0.930607187
ensp00000316464	sytl1	9015	0.930916976
ensp00000242786	cd97	9016	0.93102024
ensp00000319883	emr2	9017	0.931123503
ensp00000361646	zmynd12	9018	0.931226766
ensp00000354620	foxj3	9020	0.931433292
ensp00000352920	zmym1	9021	0.931536555
ensp00000361895	mfsd2a	9022	0.931639818
ensp00000356791	dpt	9023	0.931743081
ensp00000360065	wdr78	9024	0.931846344
ensp00000390282	hla-c	9025	0.931949608
ensp00000365817	hla-e	9026	0.932052871
ensp00000265316	abcb6	9028	0.932259397
ensp00000344155	abca2	9029	0.93236266
ensp00000349396	abcd4	9030	0.932465923
ensp00000344055	ap3d1	9031	0.932569186
ensp00000338777	ap3s2	9032	0.932672449
ensp00000327431	hbg1	9034	0.932878976
ensp00000301021	trappc2l	9035	0.932982239
ensp00000351896	trappc4	9036	0.933085502
ensp00000362261	trappc3	9037	0.933188765
ensp00000006275	trappc6a	9039	0.933395291
ensp00000352708	trappc2	9040	0.933498554
ensp00000302783	trappc1	9042	0.933705081
ensp00000377040	aass	9044	0.933911607
ensp00000306477	btd	9045	0.93401487
ensp00000338387	hlcs	9046	0.934118133
ensp00000295598	atp1a1	9047	0.934221396
ensp00000357060	atp1a4	9048	0.934324659
ensp00000286371	atp1b3	9049	0.934427922
ensp00000302397	atp1a3	9050	0.934531185
ensp00000354490	atp1a2	9053	0.934840975
ensp00000362751	rabgap1	9054	0.934944238
ensp00000311449	rab6a	9055	0.935047501
ensp00000307853	mus81	9056	0.935150764
ensp00000339897	eme1	9057	0.935254027
ensp00000307939	gcc2	9058	0.93535729

ensp00000354486	golga4	9060	0.935563817
ensp00000354398	cyth1	9061	0.93566708
ensp00000264718	gpn1	9062	0.935770343
ensp00000228827	gpn3	9063	0.935873606
ensp00000264192	cytip	9064	0.935976869
ensp00000349351	bicd2	9065	0.936080132
ensp00000281474	bicd1	9066	0.936183395
ensp00000302936	dynlrb2	9069	0.936493185
ensp00000241041	pex16	9070	0.936596448
ensp00000386385	pxmp4	9071	0.936699711
ensp00000296218	dnali1	9072	0.936802974
ensp00000336762	ang	9073	0.936906237
ensp00000344193	rnase1	9076	0.937216026
ensp00000355086	dars2	9077	0.93731929
ensp00000258955	rsad1	9078	0.937422553
ensp00000281543	guf1	9079	0.937525816
ensp00000290846	trmu	9080	0.937629079
ensp00000402038	mto1	9081	0.937732342
ensp00000351644	gtpbp3	9082	0.937835605
ensp00000301956	acsm1	9083	0.937938868
ensp00000340200	glyat	9084	0.938042131
ensp00000306185	antxr2	9085	0.938145394
ensp00000288532	coq5	9086	0.938248658
ensp00000301945	antxr1	9087	0.938351921
ensp00000420298	cd200	9088	0.938455184
ensp00000311035	cd200r1	9089	0.938558447
ensp00000333946	coq6	9090	0.93866171
ensp00000322316	coq7	9091	0.938764973
ensp00000368858	tmem14b	9092	0.938868236
ensp00000229563	tmem14c	9093	0.938971499
ensp00000254759	coq3	9094	0.939074762
ensp00000356076	dyrk3	9095	0.939178026
ensp00000381333	crhr1	9098	0.939487815
ensp00000331106	pex26	9099	0.939591078
ensp00000303511	pex6	9100	0.939694341
ensp00000248633	pex1	9101	0.939797604
ensp00000372295	gjb2	9102	0.939900867
ensp00000354900	gjb1	9103	0.940004131
ensp00000413845	brd2	9107	0.940417183
ensp00000358272	ndufaf4	9109	0.940623709
ensp00000391200	znf692	9110	0.940726972
ensp00000296099	ucn	9112	0.940933499
ensp00000221086	mtmr9	9113	0.941036762
ensp00000363985	mtmr8	9114	0.941140025
ensp00000227135	spa17	9117	0.941449814
ensp00000184183	ropn1	9118	0.941553077
ensp00000376921	ntng2	9119	0.94165634

ensp00000279036	pigt	9121	0.941862867
ensp00000309430	pigs	9122	0.94196613
ensp00000365116	ctrc	9123	0.942069393
ensp00000252034	eln	9125	0.942275919
ensp00000305714	bmp1	9126	0.942379182
ensp00000223061	pcolce	9127	0.942482445
ensp00000061240	tll1	9128	0.942585708
ensp00000295992	pcolce2	9129	0.942688971
ensp00000251582	adamts2	9130	0.942792235
ensp00000362304	adamts14	9131	0.942895498
ensp00000286657	adamts3	9132	0.942998761
ensp00000231004	lox	9133	0.943102024
ensp00000309953	efemp2	9134	0.943205287
ensp00000369292	samd9	9135	0.94330855
ensp00000326247	samd9l	9136	0.943411813
ensp00000363452	man1c1	9137	0.943515076
ensp00000348959	man1a2	9138	0.94361834
ensp00000357453	man1a1	9139	0.943721603
ensp00000360645	man1b1	9140	0.943824866
ensp00000318147	edem3	9141	0.943928129
ensp00000311888	mgat1	9142	0.944031392
ensp00000256497	edem1	9143	0.944134655
ensp00000265978	fam160a2	9144	0.944237918
ensp00000300245	aktip	9145	0.944341181
ensp00000360252	hook1	9147	0.944547708
ensp00000305699	hook3	9148	0.944650971
ensp00000363616	edem2	9149	0.944754234
ensp00000291554	cryaa	9150	0.944857497
ensp00000280904	dsc2	9151	0.94496076
ensp00000261590	dsg2	9152	0.945064023
ensp00000303423	fnta	9153	0.945167286
ensp00000246166	fntb	9155	0.945373812
ensp00000404676	pggt1b	9156	0.945477076
ensp00000330587	mt1h	9158	0.945683602
ensp00000274565	spink7	9159	0.945786865
ensp00000391397	mt1g	9163	0.946199917
ensp00000260191	htr3b	9166	0.946509707
ensp00000347754	htr3a	9168	0.946716233
ensp00000414624	prpsap1	9173	0.947232549
ensp00000361512	prps1	9174	0.947335812
ensp00000405295	hla-dra	9175	0.947439075
ensp00000407233	psmb9	9176	0.947542338
ensp00000345008	fbln5	9178	0.947748864
ensp00000261483	man2a1	9179	0.947852127
ensp00000353655	man2a2	9180	0.94795539
ensp00000345270	mgat3	9181	0.948058653
ensp00000338983	muc1	9182	0.948161917

ensp00000361214	nrg3		9183	0.94826518
ensp00000261921	loxl1		9184	0.948368443
ensp00000292401	azgp1		9185	0.948471706
ensp00000291009	pip		9186	0.948574969
ensp00000347140	asgr2		9187	0.948678232
ensp00000269299	asgr1		9188	0.948781495
ensp00000222248	slc5a5		9189	0.948884758
ensp00000278198	lrrc4c		9191	0.949091285
ensp00000359085	ntng1		9192	0.949194548
ensp00000288167	il17rb		9194	0.949401074
ensp00000353157		2-Sep	9196	0.9496076
ensp00000341524		6-Sep	9197	0.949710863
ensp00000381992		7-Sep	9198	0.949814126
ensp00000348565	uba5		9199	0.94991739
ensp00000391249		9-Sep	9200	0.950020653
ensp00000239878	ufm1		9201	0.950123916
ensp00000320076	ush1g		9204	0.950433705
ensp00000005226	ush1c		9205	0.950536968
ensp00000386331	myo7a		9206	0.950640231
ensp00000299314	gnptab		9207	0.950743494
ensp00000352665	atp2c1		9210	0.951053284
ensp00000264968	mgat4a		9212	0.95125981
ensp00000338487	mgat4b		9213	0.951363073
ensp00000307423	mgat2		9214	0.951466336
ensp00000353910	fut8		9215	0.951569599
ensp00000391227	mgat5b		9216	0.951672862
ensp00000281923	mgat5		9217	0.951776126
ensp00000345728	atp7a		9218	0.951879389
ensp00000242839	atp7b		9219	0.951982652
ensp00000342626	eya1		9220	0.952085915
ensp00000316854	atox1		9221	0.952189178
ensp00000247182	six1		9222	0.952292441
ensp00000379680	naga		9224	0.952498967
ensp00000343318	b3galt5		9225	0.95260223
ensp00000379353	st8sia1		9226	0.952705494
ensp00000318142	fut7		9227	0.952808757
ensp00000352456	slc33a1		9229	0.953015283
ensp00000341562	b4galnt1		9230	0.953118546
ensp00000318445	st3gal1		9231	0.953221809
ensp00000345477	st3gal2		9232	0.953325072
ensp00000321343	st8sia5		9233	0.953428335
ensp00000417583	st6galnac5		9234	0.953531599
ensp00000291839	st6galnac6		9235	0.953634862
ensp00000329214	st6galnac3		9236	0.953738125
ensp00000366800	dnajc11		9237	0.953841388
ensp00000355299	tsen15		9238	0.953944651
ensp00000353331	kirrel2		9239	0.954047914

ensp00000312021	fut1	9240	0.954151177
ensp00000375748	fut2	9241	0.95425444
ensp00000305603	fut3	9242	0.954357703
ensp00000286955	fut6	9243	0.954460967
ensp00000351602	fut4	9244	0.95456423
ensp00000302599	fut9	9246	0.954770756
ensp00000309096	b3gnt1	9247	0.954874019
ensp00000369055	b4galt1	9248	0.954977282
ensp00000349293	b4galt2	9249	0.955080545
ensp00000320965	b4galt3	9250	0.955183808
ensp00000265012	gcnt2	9252	0.955390335
ensp00000356404	b3galt2	9254	0.955596861
ensp00000303740	b3galt1	9255	0.955700124
ensp00000355273	st6gal2	9256	0.955803387
ensp00000316173	b3gnt5	9257	0.95590665
ensp00000321874	b3gnt3	9258	0.956009913
ensp00000377717	st3gal6	9259	0.956113176
ensp00000266718	lum	9260	0.956216439
ensp00000328983	chst6	9262	0.956422966
ensp00000307911	chst2	9263	0.956526229
ensp00000338783	chst5	9264	0.956629492
ensp00000227495	st3gal4	9265	0.956732755
ensp00000262915	st3gal3	9266	0.956836018
ensp00000336733	st6galnac4	9267	0.956939281
ensp00000396774	muc20	9268	0.957042544
ensp00000304207	muc4	9269	0.957145808
ensp00000406861	muc6	9270	0.957249071
ensp00000415183	muc2	9273	0.95755886
ensp00000302021	muc7	9276	0.957868649
ensp00000305595	b3gnt2	9277	0.957971912
ensp00000319636	b3gnt4	9278	0.958075176
ensp00000287590	b3gnt7	9279	0.958178439
ensp00000417175	vapb	9280	0.958281702
ensp00000345656	vapa	9281	0.958384965
ensp00000259056	galnt5	9282	0.958488228
ensp00000288988	galnt14	9284	0.958694754
ensp00000269195	galnt1	9285	0.958798017
ensp00000309270	chst1	9289	0.95921107
ensp00000365920	gcnt1	9292	0.959520859
ensp00000347041	fmod	9297	0.960037175
ensp00000317027	gcnt4	9299	0.960243701
ensp00000169298	st6gal1	9300	0.960346964
ensp00000225276	st6galnac2	9301	0.960450227
ensp00000360776	b4galt5	9302	0.96055349
ensp00000297107	galnt10	9303	0.960656753
ensp00000376465	galnt3	9305	0.96086328
ensp00000348668	galnt6	9307	0.961069806

ensp00000315835	galnt11	9308	0.961173069
ensp00000355632	galnt2	9309	0.961276332
ensp00000380488	galnt9	9310	0.961379595
ensp00000265000	galnt7	9311	0.961482858
ensp00000364150	galnt12	9316	0.961999174
ensp00000223122	c1galt1	9319	0.962308963
ensp00000156626	st6galnac1	9320	0.962412226
ensp00000238633	npc2	9324	0.962825279
ensp00000269228	npc1	9325	0.962928542
ensp00000264773	kcnn2	9327	0.963135068
ensp00000369965	btf3	9329	0.963341594
ensp00000403817	naca	9330	0.963444857
ensp00000315757	lcp1	9331	0.963548121
ensp00000394842	gca	9332	0.963651384
ensp00000280772	ank3	9333	0.963754647
ensp00000283256	scn2a	9334	0.96385791
ensp00000374332	tsn	9338	0.964270962
ensp00000355599	tsnax	9339	0.964374226
ensp00000298841	serpina4	9340	0.964477489
ensp00000301420	klk1	9341	0.964580752
ensp00000363941	hla-dpa1	9342	0.964684015
ensp00000399298	hla-dpb1	9343	0.964787278
ensp00000311402	slc4a2	9344	0.964890541
ensp00000406706	c6orf25	9346	0.965097067
ensp00000283254	scn3a	9348	0.965303594
ensp00000322460	scn4b	9349	0.965406857
ensp00000278947	scn2b	9350	0.96551012
ensp00000251287	hcn2	9352	0.965716646
ensp00000261917	hcn4	9353	0.965819909
ensp00000257312	dzip1	9355	0.966026435
ensp00000394033	kcnk2	9358	0.966336225
ensp00000310568	kcnk10	9359	0.966439488
ensp00000378033	kcnk4	9360	0.966542751
ensp00000246801	tsks	9361	0.966646014
ensp00000367766	rpgr	9364	0.966955803
ensp00000188312	actr6	9365	0.967059067
ensp00000307071	c15orf40	9367	0.967265593
ensp00000333551	prosc	9368	0.967368856
ensp00000410732	gabrg2	9370	0.967575382
ensp00000299267	gabrb3	9372	0.967781908
ensp00000328875	trak2	9380	0.968608013
ensp00000252971	mnx1	9381	0.968711276
ensp00000282728	hhex	9383	0.968917803
ensp00000328364	mafa	9384	0.969021066
ensp00000282549	otx1	9385	0.969124329
ensp00000260649	slc3a1	9386	0.969227592
ensp00000300896	usp32	9388	0.969434118

ensp00000263559	vps26a	9391	0.969743907
ensp00000299138	vps35	9393	0.969950434
ensp00000357106	cadm3	9394	0.970053697
ensp00000329797	cadm1	9395	0.97015696
ensp00000251047	lman1	9396	0.970260223
ensp00000317271	mcfd2	9397	0.970363486
ensp00000165524	prlh	9398	0.970466749
ensp00000239032	prlhr	9399	0.970570012
ensp00000387266	spire1	9400	0.970673276
ensp00000318884	fmn2	9401	0.970776539
ensp00000031135	bclaf1	9404	0.971086328
ensp00000261842	ap4e1	9405	0.971189591
ensp00000256658	ap4b1	9406	0.971292854
ensp00000284049	chd1	9407	0.971396117
ensp00000216513	six4	9408	0.97149938
ensp00000260645	abcg5	9410	0.971705907
ensp00000241527	mterfd2	9412	0.971912433
ensp00000419740	nsun4	9413	0.972015696
ensp00000278409	or5f1	9434	0.974184221
ensp00000279791	or5m9	9442	0.975010326
ensp00000306095	or10h2	9443	0.975113589
ensp00000373194	or5k3	9452	0.976042957
ensp00000309673	or52w1	9459	0.976765799
ensp00000323595	or8h1	9465	0.977385378
ensp00000352626	or6k2	9466	0.977488641
ensp00000325076	or10g4	9467	0.977591904
ensp00000342697	or2ag2	9488	0.97976043
ensp00000343521	or1j4	9494	0.980380008
ensp00000342836	or6c74	9495	0.980483271
ensp00000325203	or6t1	9505	0.981515903
ensp00000313110	or6s1	9508	0.981825692
ensp00000362784	or5c1	9514	0.982445271
ensp00000353343	or52m1	9517	0.98275506
ensp00000322593	or51g2	9522	0.983271375
ensp00000324958	or4c15	9526	0.983684428
ensp00000342008	or2t27	9529	0.983994217
ensp00000334441	or10j5	9544	0.985543164
ensp00000322866	or52n5	9546	0.98574969
ensp00000311038	or6m1	9548	0.985956216
ensp00000293614	or7g1	9550	0.986162743
ensp00000335535	or6n1	9551	0.986266006
ensp00000318834	or10h4	9552	0.986369269
ensp00000386209	or2a2	9556	0.986782321
ensp00000387523	or7e24	9557	0.986885584
ensp00000303096	or5a1	9562	0.9874019
ensp00000334456	or5d14	9573	0.988537794
ensp00000307751	or4x2	9574	0.988641057

ensp00000344040	or2s2	9582	0.989467162
ensp00000353516	or2w3	9590	0.990293267
ensp00000329210	or2t10	9592	0.990499793
ensp00000327585	or1d2	9601	0.991429162
ensp00000248072	or7c2	9619	0.993287898
ensp00000369742	or52r1	9638	0.995249897
ensp00000304419	or2b2	9647	0.996179265
ensp00000330280	or8b8	9649	0.996385791
ensp00000345163	or51f1	9665	0.998038001
ensp00000305424	or1f1	9666	0.998141264
ensp00000308082	or10p1	9669	0.998451053
ensp00000300127	or4d6	9673	0.998864106
ensp00000333184	or4f15	9674	0.998967369
ensp00000378516	or10w1	9683	0.999896737
