# Supplementary material for: Transcriptome analysis reveals manifold mechanisms of cyst development in ADPKD
Source: Hum Genomics. 2016 Nov 21;10:37. doi: 10.1186/s40246-016-0095-x (PMC5117508; doi:10.1186/s40246-016-0095-x)
Supplement: Additional file 3: — Microarray validation by qRT-PCR. (PDF 235 kb) [file 40246_2016_95_MOESM3_ESM.pdf]

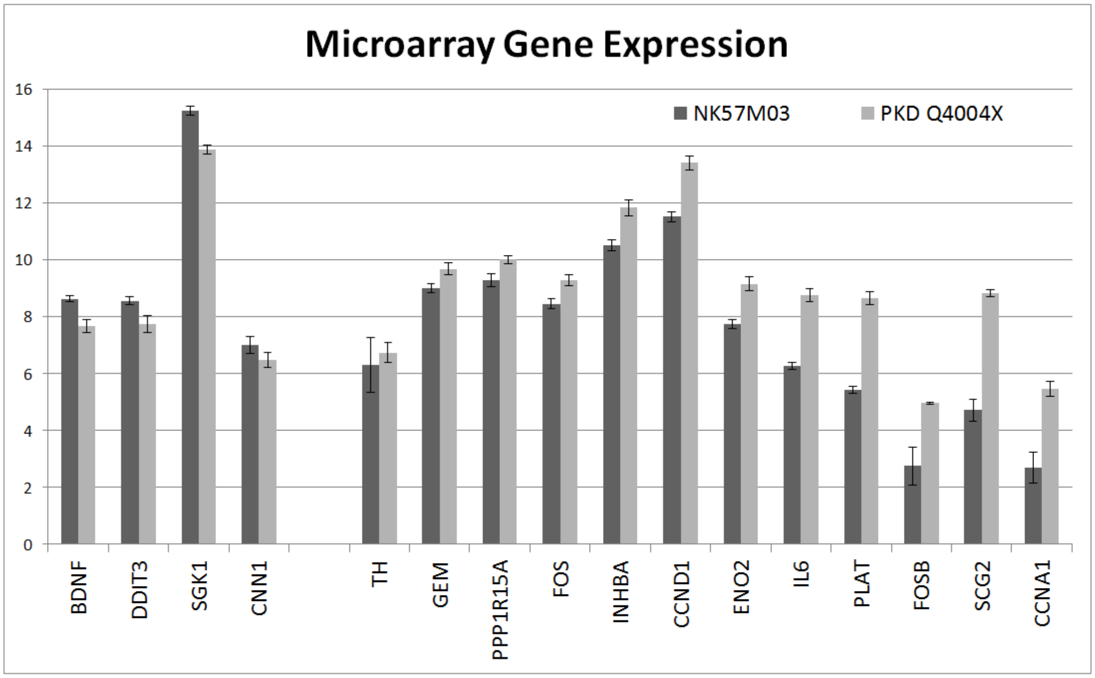

PCR Gene Expression		
BDNF	-6.29	0.008558
DDIT3	-1.94	0.001954
SGK1	-2.49	0.019200
CNN1	-2.32	0.012049
GEM	1.85	0.009975
PPP1R15A	2.71	0.002131
FOS	8.50	0.000123
INHBA	10.95	0.000026
CCND1	42.40	0.000123
ENO2	4.46	0.001085
IL6	18.63	0.000033
PLAT	29.98	0.003126
FOSB	10.82	0.001328
SCG2	47.11	0.000044
CCNA1	7.48	0.004563

Additional File 3. Microarray Validation by PCR. A sampling of microarray data was validated using PCR. Trends in up or down regulation of expression were preserved.
