# Supplementary material for: Transcriptome analysis reveals manifold mechanisms of cyst development in ADPKD
Source: Hum Genomics. 2016 Nov 21;10:37. doi: 10.1186/s40246-016-0095-x (PMC5117508; doi:10.1186/s40246-016-0095-x)
Supplement: Additional file 4: — Protein expression information on NOD-like receptor signaling, potassium ion transport, and oxidative phosphorylation gene sets from the human protein atlas. (PDF 94.6 kb) [file 40246_2016_95_MOESM4_ESM.pdf]

POTASSIUM CHANNELS					
symbol	gene description	tissue localization (RNA)	kidney localization	cellular localization	Human Prot Atlas
**Down in NC-ADPKD and further down in C-ADPKD**					
**ATP1A4**	ATPase Na+/K+ transporting subunit alpha 4	lymph node, placenta, testis, urinary bladder, tonsil, spleen, gallbladder. Sperm-specific protein essential for sperm motility and fertility (http://www.ncbi.nlm.nih.gov/pubmed/26343794)		predicted membranous	not shown
**Up in NC-ADPKD and further up in C-ADPKD**					
**ATP12A** - K absorption in various tissues	ATPase, H+/K+ transporting, nongastric, alpha polypeptide	esophagus, rectum, kidney, placenta, skin, tonsil, lung, high in testis	tubules, strong apical in DT	cytoplasmic and membranous	http://www.proteinatlas.org/ENSG00000075673-ATP12A/tissue/kidney#imid_10106048
**KCNJ12**	K inwardly-rectifying channel, subfamily J, member 12	esophagus, colon, kidney, testis, prostate, fallopian tube, skin, adipose, skeletal muscle (hi), tonsil, spleen, cerebral cortex, thyroid, adrenal, lung, heart	glomerulus (med), tubules (hi)	membranous/apical	http://www.proteinatlas.org/ENSG00000184185-KCNJ12/tissue/kidney#imid_7285448
**KCNK3** (aka OAT1)	K outwardly rectifying channel	pancreas, placenta, cerebral cortex, adrenal (hi), lung, heart		predicted membranous	
**KCNMB4** - slows activation kinetics, leads to steeper Ca sensitivity	large conductance, voltage and Ca-sensitive K channels	widespread, highest in cerebral cortex and adrenal, high in kidney and lung		predicted membranous	not shown
**KCNC4** – - mediates voltage-dependent K ion permeability of excitable membranes	K voltage-gated channel, Shaw-related subfamily, member 4	widespread, including kidney	tubules, strongest in DT	cytoplasmic and membranous	http://www.proteinatlas.org/ENSG00000116396-KCNC4/tissue/kidney#imid_3844024
**KCNQ1**	K voltage-gated channel, KQT-like subfamily, member 1	widespread, high in digestrive tract and urinary tract including kidney, highest in thyroid and adrenal	glomerulus (low), PT (medium)	cytoplasmic and basolateral	http://www.proteinatlas.org/ENSG00000053918-KCNQ1/tissue/kidney#imid_15227078
KCNS1	K voltage-gated channel, channel modifier subfamily S member	gallbladder, salivary, testis, skin, cerebral cortex,		predicted membranous	not shown
**Higher in NC-ADPKD then lower in C-ADPKD**					
KCNN4 – implicated in cyst fluid secretion, inhibition may delay cyst progression	voltage independent, activated by intracellular Ca, promotes Ca influx	gallbladder, digestive tract, kidney and bladder, testis and prostate, breast and female reproductive system, bone marrow, lymph node, tonsil, spleen	glomerulus, tubules, greater in PTs, greater basolateral	predicted intracellular, membrane, secreted	http://www.proteinatlas.org/ENSG00000104783-KCNN4/tissue/kidney#imid_16483971
KCNV1	K voltage gated – might inhibit outward rectifying	cerebral cortex			
KCNS3	K voltage-gated channel, delayed-rectifier, subfamily S, member 3	widespread including kidney, highest in placenta, skeletal muscle, lung	all tubules, stronger in DT	cytoplasmic and membranous	http://www.proteinatlas.org/ENSG00000170745-KCNS3/tissue/kidney#imid_3801928
**NOD-like signaling**					
symbol		tissue localization (RNA)	kidney localization	cellular localization	Human Prot Atlas
**Up in NC-ADPKD and further up in C-ADPKD**					
BIRC3	Baculoviral IAP repeat containing 3-member of IAP family of proteins that inhibit apoptosis by binding to TNF receptor-associated factors TRAF1 and TRAF2, interfering with activation of ICE-like proteases	widespread, including kidney	all tubules, higher in DT	membranous	http://www.proteinatlas.org/ENSG00000023445-BIRC3/tissue/kidney#imid_1042428
CCL2	Chemokine (C-C motif) ligand 2 - chemotactic for monocytes and basophils but not for neutrophils or eosinophils	widespread, including kidney	all tubules, higher in DT	secreted, cytoplasmic and membranous	http://www.proteinatlas.org/ENSG00000108691-CCL2/tissue/kidney#imid_5814476
CXCL1	Chemokine (C-X-C motif) ligand 1	liver and pancreas, GI tract, kidney and bladder, prostate, adipose, blood and immune system		secreted	not shown
IKBKB	Inhibitor of kappa light polypeptide gene enhancer in B-cells, kinase beta	widespread, including kidney	glom and tubules, higher in DT	cytoplasmic and membranous	http://www.proteinatlas.org/ENSG00000104365-IKBKB/tissue/kidney#img
IL18	Interleukin 18	widespread, including kidney, not in CNS or cardiovascular	tubules, very high in DTs	cytoplasmic and membranous	http://www.proteinatlas.org/ENSG00000150782-IL18/tissue/kidney#img
IL1B	Interleukin 1, beta	widespread, including kidney, highest in bone marrow		Intracellular	not shown
IL6	Interleukin 6	widespread, present in bladder protein not confirmed in kidney	not	Intracellular, Secreted	http://www.proteinatlas.org/ENSG00000136244-IL6/tissue/kidney#imid_6471288
MAPK11	Mitogen-activated protein kinase 11	widespread, including kidney	tubules	Intracellular, cytoplasmic granular	http://www.proteinatlas.org/ENSG00000185386-MAPK11/tissue/kidney#img
MAPK13	Mitogen-activated protein kinase 13	widespread, including kidney	glom and tubules, slightly higher DTs	Intracellular	http://www.proteinatlas.org/ENSG00000156711-MAPK13/tissue/kidney#imid_6843584
NFKBIA	Nuclear factor of kappa light polypeptide gene enhancer in B-cells inhibitor, alpha	widespread, including kidney	glom and tubules, slightly higher DT	Intracellular/apical	http://www.proteinatlas.org/ENSG00000100906-NFKBIA/tissue/kidney#img
PYCARD	PYD and CARD domain containing	widespread, low in kidney	tubules, level varies	cytoplasmic and membranous	http://www.proteinatlas.org/ENSG00000103490-PYCARD/tissue/kidney#imid_16851519
TNF	Tumor necrosis factor	widespread, highest in blood and immune system		Intracellular	not shown
TNFAIP3	Tumor necrosis factor, alpha-induced protein 3	widespread, including kidney	glom and tubules, higher glom. DTs	Intracellular	http://www.proteinatlas.org/ENSG00000118503-TNFAIP3/tissue/kidney#img
**Up in NC-ADPKD and further down in C-ADPKD**					
CARD8	Caspase recruitment domain family, member 8	widespread, including kidney	glom and tubules	cytoplasmic, membranous, nuclear	http://www.proteinatlas.org/ENSG00000105483-CARD8/tissue/kidney#img
CASP1	Caspase 1, apoptosis-related cysteine peptidase	widespread, including kidney	glom and tubules, higher in DT	Intracellular	http://www.proteinatlas.org/ENSG00000137752-CASP1/tissue/kidney#img
HSP90AA1	Heat shock protein 90kDa alpha (cytosolic), class A member 1	RNA widespread including kidney	protein not detected		http://www.proteinatlas.org/ENSG00000080824-HSP90AA1/tissue/kidney#imid_701408
MAPK9	Mitogen-activated protein kinase 9	widespread, including kidney	glom and tubules, slightly higher DTs	cytoplasmic and membranous	http://www.proteinatlas.org/ENSG00000050748-MAPK9/tissue/kidney#imid_2449392
NFKB1	Nuclear factor of kappa light polypeptide gene enhancer in B-cells 1	widespread, including kidney	glom and tubules, low in both	Intracellular	http://www.proteinatlas.org/ENSG00000109320-NFKB1/tissue/kidney#img
**Up in NC-ADPKD then back to normal in C-ADPKD**					
HSP90B1	Heat shock protein 90kDa beta (Grp94), member 1	widespread, including kidney	glom and tubules	cytoplasmic and membranous, secreted	http://www.proteinatlas.org/ENSG00000166598-HSP90B1/tissue/kidney#img
**Down in NC-ADPKD then badk to normal in C-ADPKD**					
CARD9	Caspase recruitment domain family, member 9	widespread, highest in bone marrow and spleen	protein not detected	intracellular	http://www.proteinatlas.org/ENSG00000187796-CARD9/tissue/kidney#imid_16427147
**Oxydative phosphorylation - all are decreased in ADPKD**					
symbol		tissue localization (RNA)	kidney localization	cellular localization	Human Prot Atlas
ATP5C1	ATP synthase, H+ transporting, mitochondrial F1 complex, gamma polypeptide 1	widespread, including kidney	tubules	cytoplasmic and membranous	http://www.proteinatlas.org/ENSG00000165629-ATP5C1/tissue/kidney#imid_16721755
ATP5F1	ATP synthase, H+ transporting, mitochondrial Fo complex, subunit B1	widespread, including kidney	tubules	cytoplasmic	http://www.proteinatlas.org/ENSG00000116459-ATP5F1/tissue/kidney#imid_18209039
ATP5G1	ATP synthase, H+ transporting, mitochondrial Fo complex, subunit C1 (subunit 9)	widespread, including kidney		cytoplasmic granular	not shown
COX5B	Cytochrome c oxidase subunit Vb	widespread, including kidney	tubules	cytoplasmic	http://www.proteinatlas.org/ENSG00000135940-COX5B/tissue/kidney#imid_8306440
COX6C	Cytochrome c oxidase subunit VIc	widespread, including kidney	some glom, mostly tubules	cytoplasmic, granular	http://www.proteinatlas.org/ENSG00000164919-COX6C/tissue/kidney#imid_4077900
COX7A2	Cytochrome c oxidase subunit VIIa polypeptide 2 (liver)	widespread, including kidney		intracellular, membrane	not shown
COX7B	Cytochrome c oxidase subunit VIIb	widespread, including kidney	tubules	cytoplasmic	http://www.proteinatlas.org/ENSG00000131174-COX7B/tissue/kidney#img
CYC1	Cytochrome c-1	widespread, including kidney	tubules, higher in DT	cytoplasmic, granular	
NDUFA5	NADH dehydrogenase (ubiquinone) 1 alpha subcomplex, 5	widespread, including kidney	tubules, higher in DT	cytoplasmic, granular	http://www.proteinatlas.org/ENSG00000128609-NDUFA5/tissue/kidney#imid_11387659
NDUFA8	NADH dehydrogenase (ubiquinone) 1 alpha subcomplex, 8, 19kDa	widespread, including kidney	glom and tubules	cytoplasmic, granular	http://www.proteinatlas.org/ENSG00000119421-NDUFA8/tissue/kidney#imid_11107967
NDUFB1	NADH dehydrogenase (ubiquinone) 1 beta subcomplex, 1, 7kDa	widespread, including kidney	glom and tubules	nuclear	http://www.proteinatlas.org/ENSG00000183648-NDUFB1/tissue/kidney#imid_13585315
NDUFB2	NADH dehydrogenase (ubiquinone) 1 beta subcomplex, 2, 8kDa	widespread, including kidney	glom and tubules, higher in DTs	cytoplasmic	http://www.proteinatlas.org/ENSG00000090266-NDUFB2/tissue/kidney#imid_15122634
NDUFB6	NADH dehydrogenase (ubiquinone) 1 beta subcomplex, 6, 17kDa	widespread, including kidney	glom and tubules	cytoplasmic	http://www.proteinatlas.org/ENSG00000165264-NDUFB6/tissue/kidney#imid_11755515
NDUFB9	NADH dehydrogenase (ubiquinone) 1 beta subcomplex, 9, 22kDa	widespread, including kidney	tubules	cytoplasmic, granular	http://www.proteinatlas.org/ENSG00000147684-NDUFB9/tissue/kidney#imid_11196215
SDHB	Succinate dehydrogenase complex, subunit B, iron sulfur (Ip)	widespread, including kidney	glom and tubules	cytoplasmic and membranous	http://www.proteinatlas.org/ENSG00000117118-SDHB/tissue/kidney#imid_18632340
UQCRB	Ubiquinol-cytochrome c reductase binding protein	widespread, including kidney	glom and tubules, higher in DTs	cytoplasmic and membranous	http://www.proteinatlas.org/ENSG00000156467-UQCRB/tissue/kidney#imid_11300991
UQCRFS1	Ubiquinol-cytochrome c reductase, Rieske iron-sulfur polypeptide 1	widespread, including kidney	glom and tubules, higher in DTs	cytoplasmic, granular	http://www.proteinatlas.org/ENSG00000169021-UQCRFS1/tissue/kidney#imid_10938087
UQCRH	Ubiquinol-cytochrome c reductase hinge protein	widespread, including kidney	tubules, higher in DTs	cytoplasmic, granular	http://www.proteinatlas.org/ENSG00000173660-UQCRH/tissue/kidney#imid_12620453
