# Supplementary material for: Transcriptome analysis reveals manifold mechanisms of cyst development in ADPKD
Source: Hum Genomics. 2016 Nov 21;10:37. doi: 10.1186/s40246-016-0095-x (PMC5117508; doi:10.1186/s40246-016-0095-x)
Supplement: Additional file 5: — Relative transcriptogram for ADPKD cells. (PDF 330 kb) [file 40246_2016_95_MOESM5_ESM.pdf]

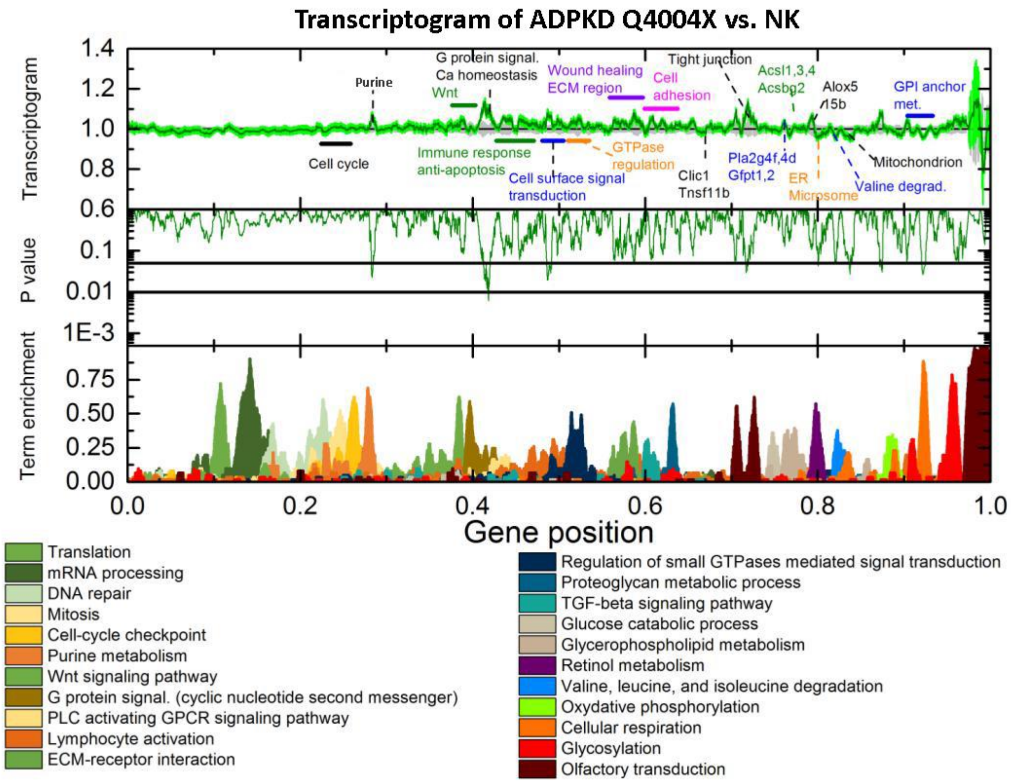

Additional Figure 5. Transcriptogram analysis for ADPKD Q4004X vs. NK. For all three subpanels (Transcriptogram, P-value, Term enrichment), the x-axis represents the ordered gene list. The upper panel shows the relative average (± S.E.) trasncriptogram for ADPKD Q4004X (green line) relative to NK (black line). The light green and grey regions around the lines are the standard errors.

The central panel presents the P value obtained from a two tail Weyl's t-test for each point of the transcriptograms and the two horizontal lines mark P =0.05 and P=0.01. The lower panel projects on the ordered list selected GOBP terms and KEGG pathways found differentially expressed in the comparison of ADPKD Q4004X to NK samples. The transcriptograms show many regions of the ordered list with differential expression exceptions, marked by inverted peaks in P values.

The ADPKD Q4004X cell line was derived from an end stage polycystic kidney of a 57 year old male and have been previously described (Herbert et al., 2013). The cells have a single detectable truncating mutation in PC1.
